# Copper-catalyzed atroposelective synthesis of C–O axially chiral compounds enabled by chiral 1,8-naphthyridine based ligands†

**DOI:** 10.1039/d4sc01074d

**Published:** 2024-03-22

**Authors:** Lei Dai, Xueting Zhou, Jiami Guo, Qingqin Huang, Yixin Lu

**Affiliations:** a Department of Chemistry, National University of Singapore 117543 Singapore chmlyx@nus.edu.sg; b Joint School of National University of Singapore and Tianjin University, International Campus of Tianjin University Binhai New City Fuzhou 350207 China; c Chongqing Key Laboratory of Natural Product Synthesis and Drug Research, School of Pharmaceutical Sciences, Chongqing University Chongqing 401331 China

## Abstract

Axially chiral molecular scaffolds are widely present in therapeutic agents, natural products, catalysts, and advanced materials. The construction of such molecules has garnered significant attention from academia and industry. The catalytic asymmetric synthesis of axially chiral biaryls, along with other non-biaryl axially chiral molecules, has been extensively explored in the past decade. However, the atroposelective synthesis of C–O axial chirality remains largely underdeveloped. Herein, we document a copper-catalyzed atroposelective construction of C–O axially chiral compounds using novel 1,8-naphthyridine-based chiral ligands. Mechanistic investigations have provided good evidence in support of a mechanism involving synergistic interplay between a desymmetrization reaction and kinetic resolution process. The method described in this study holds great significance for the atroposelective synthesis of C–O axially chiral compounds, with promising applications in organic chemistry. The utilization of 1,8-naphthyridine-based ligands in copper catalysis is anticipated to find broad applications in asymmetric copper(i)-catalyzed azide–alkyne cycloadditions (CuAACs) and beyond.

## Abstract 2

A copper-catalyzed atroposelective construction of C–O axially chiral compounds using novel 1,8-naphthyridine-based chiral ligands was reported.

## Graphical abstract

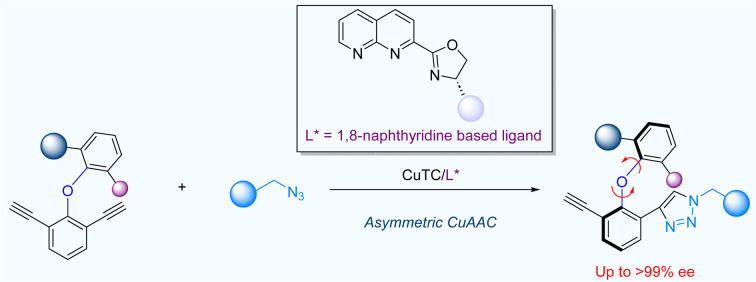

## Introduction

**Scheme 1:**
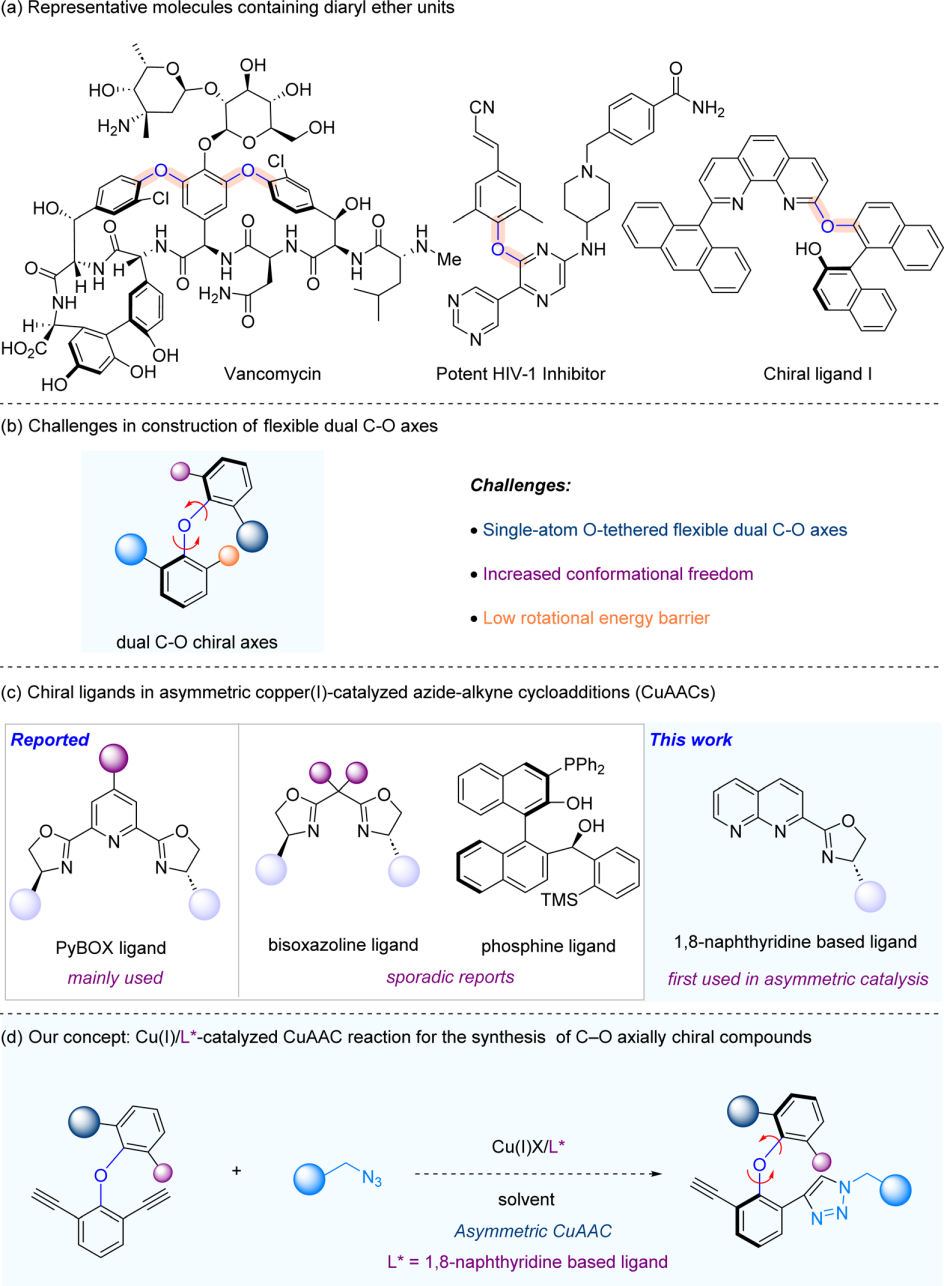
Research background. (a) Representative molecules containing diaryl ether units. (b) Challenges in construction of a flexible dual C–O axis. (c) Chiral ligands in asymmetric CuAACs. (d) Our concept: copper/1,8-naphthyridine based ligands in asymmetric CuAACs for the synthesis of C–O axially chiral compounds.

Axially chiral molecular scaffolds widely exist in bioactive molecules and pharmaceutical agents, as well as privileged chiral catalysts and ligands in asymmetric catalysis.^1^ Consequently, their atroposelective synthesis is an area that attracts much attention from chemists in both academia and industry.^2^ In the past decade, the catalytic asymmetric synthesis of axially chiral biaryls, as well as other non-biaryl axially chiral molecules including axially chiral amides, anilines, styrenes and boranes bearing C–C, C–N, C–B and N–N axes, has been extensively studied.^3^ However, atroposelective synthesis of C–O axial chirality is a very much underdeveloped area. Axially chiral diaryl ethers bearing C–O axial chirality are privileged substructures that are present in numerous biologically active molecules, natural products, and chiral ligands (Scheme 1a).^4^ Current atroposelective synthetic methods make use of di-aldehydes as substrates through either enzymatic or organocatalytic approaches.^5^ In 2010, Clayden and coworkers reported the first catalytic atroposelective synthesis of axially chiral diaryl ethers *via* enzyme catalysis.^5*a*^ In 2018, Gustafson and co-workers described a C(sp^2^)–H alkylation with nitroalkanes to access enantioenriched diaryl ethers *via* phase-transfer catalysis.^5*b*^ Recently, Zeng and Zhong,^5*c*^ and Yang^5*d*^ groups documented the atroposelective synthesis of axially diaryl ethers *via* chiral phosphoric acid catalysis. Very recently, the atroposelective synthesis of axially diaryl ethers was independently reported by Biju,^5*e*^ Ye^5*f*^ and Gao^5*g*^ groups enabled by *N*-heterocyclic carbene-catalysed desymmetrization of dialdehydes. The challenges in atroposelective synthesis of C–O axially chiral diaryl ethers lie in the following: (1) single-atom O-tethered flexible dual C–O axes, (2) increased conformational freedom, and (3) low rotational energy barrier (Scheme 1b).

To develop a viable atroposelective synthesis method of diaryl ethers, we envision that asymmetric copper(i)-catalyzed azide–alkyne cycloadditions (CuAACs) may be utilized, thus providing a straightforward approach to the asymmetric synthesis of C–O axially chiral compounds. The CuAAC, first disclosed by Meldal^6^ and Sharpless^7^ independently in the 2000s, is the key transformation in the “click chemistry”^8^ arsenal. Owing to its operational simplicity, excellent functional group tolerance and generally high yields, CuAAC has found wide application across different fields of chemistry, spanning from materials science to life sciences.^9^ Nonetheless, the investigation of asymmetric CuAAC has remained rather limited.^10^ The primary hurdle in developing highly efficient asymmetric CuAAC processes lies in the limited availability of suitable chiral ligands. The chiral ligand family commonly employed in asymmetric CuAAC is Pybox ligands,^11^ and the use of other ligands, *e.g.* bisoxazoline ligands^12^ and phosphine ligands,^13^ is very rare (Scheme 1c). At the outset, we envision that a catalytic system consisting of copper and chiral 1,8-naphthyridine-based ligands,^14^ which were not used in asymmetric catalysis to the best of our knowledge (Scheme 1c), may yield an efficient catalytic system that is broadly applicable to asymmetric CuAAC reactions. As part of our continuous interest in axial chirality,^15^ herein we report a copper-catalyzed asymmetric CuAAC reaction for the atroposelective synthesis of C–O axially chiral diaryl ethers through the employment of chiral 1,8-naphthyridine based ligands (Scheme 1d).

## Results and discussion

We started our investigation by choosing diaryl ether 1a and benzyl azide 2a as the model substrates, and CuTC as the metal catalyst (Table 1). When chiral 1,8-naphthyridine based ligand L1 was applied to the reaction, 76% yield and 96% ee were obtained, showing the power of this type of ligand (entry 1). Substituents on the oxazoline ring were next examined (entries 2–4). Ligand L2 with a bulky *tert*-butyl group and ligand L3 with an isopropyl group both led to much-decreased enantioselectivities (entries 2 and 3). Interestingly, when a phenyl substituent was introduced, the yield of reaction was improved to 81% with the formation of the opposite enantiomeric isomer (entry 4). The employment of L5 which bears a larger ring system did not lead to any improvement (entry 5). For comparison, the catalytic effects of various commercially available ligands were next studied. PyBox ligand L6, Box ligand L7, and unsymmetrical Pybox ligands L8 and L9 were all found to be ineffective, forming products with very poor enantioselectivities (entries 6–9). The fact that chiral 1,8-naphthyridine based ligands turned out to be more efficient than the privileged ligands for our reaction suggests that unique binding mode provided by the 1,8-naphthyridine moiety may be crucial for asymmetric induction. Notably, we did not observe product inhibition during the reaction process. The *N*,*P*-ligand L10 led to the formation of the opposite enantiomer in very low enantioselectivity (entry 10). Moreover, diamine ligand L11 yielded a racemic product, and diphosphine ligand L12 was virtually unable to promote the reaction (entries 11 and 12). Last, solvent screening was carried out (entries 13–15), while PhCF_3_ and 1,2-dichloroethane (DCE) were found to be good solvents, and CH_3_CN remains to be the solvent of choice for overall effectiveness of the reaction.

**Table 1:** Optimization of reaction conditions^a^

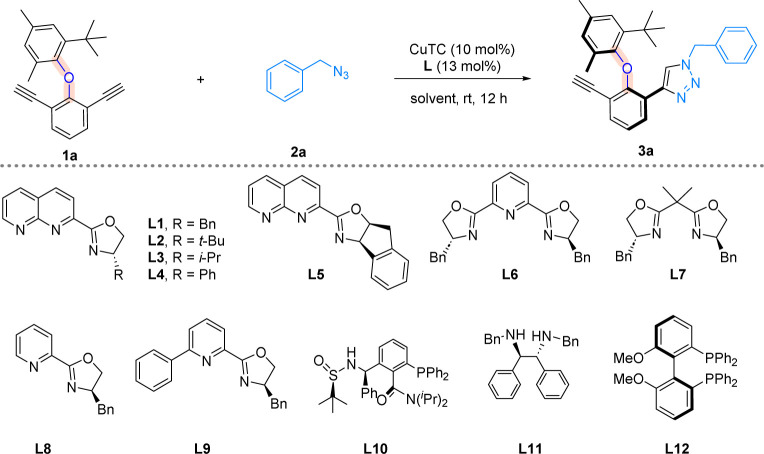			
Entry	Ligand	Yield^b^	ee^c^
1	L1	76%	96%
2	L2	70%	24%
3	L3	78%	12%
4	L4	81%	−55%
5	L5	75%	66%
6	L6	90%	11%
7	L7	60%	9%
8	L8	63%	6%
9	L9	72%	20%
10	L10	32%	−13%
11	L11	40%	0%
12	L12	Trace	n.d.
13	PhCF_3_	85%	93%
14	MeOH	60%	75%
15	DCE	82%	91%

a Reaction conditions: 1a (0.05 mmol), 2a (0.09 mmol), CuTC (10 mol%), and L (13 mol%) in solvent (2.0 ml) at room temperature for 12 h.

b Isolated yields.

c Determined by HPLC analysis on a chiral-stationary-phase. TC, thiophene-2-carboxylate; n.d., not determined; DCE, 1,2-dichloroethane.

**Scheme 2:**
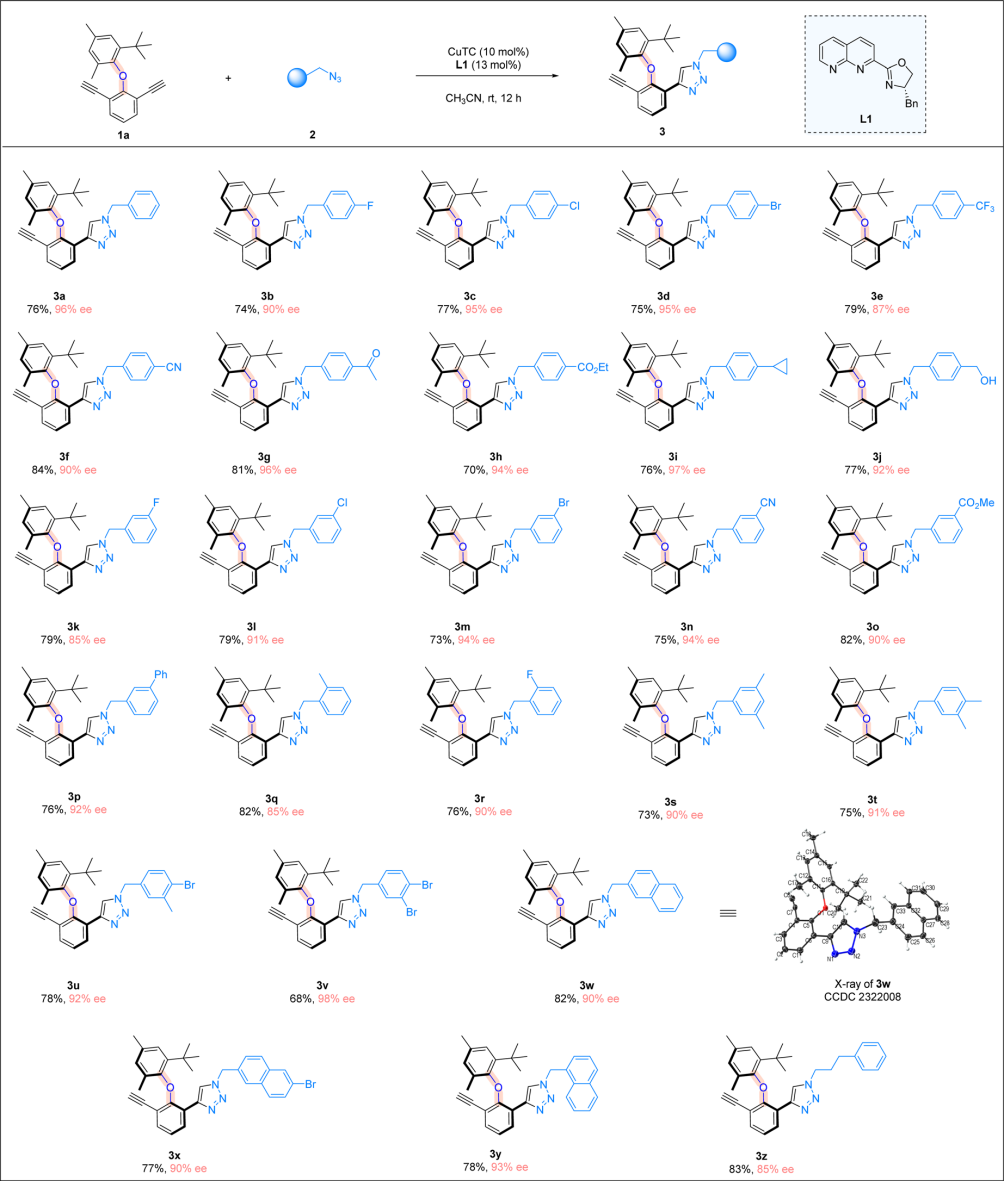
Reaction scope. Reaction conditions: 1a (0.05 mmol), 2 (0.09 mmol), CuTC (10 mol%), and L1 (13 mol%) in CH_3_CN (2.0 ml) at room temperature for 12 h; yields refer to isolated yields.

With the optimal reaction conditions in hand, we explored the scope of azides (Scheme 2). A wide range of benzyl azides bearing different aryl rings were found to be suitable, regardless of the electronic nature and substitution patterns of the substituents on the aromatic ring, and good chemical yields and excellent enantioselectivities were attainable. Benzyl azides containing a *para*-substituted phenyl ring were first investigated (3b–3j). Various halogen atoms on the phenyl ring were all well tolerated, including fluoro, chloro and bromo substituents, and good yields and excellent ee values were attainable (3b–3d). The trifluoromethyl group was also found to be suitable, although the enantioselectivity of the reaction was slightly lower (3e). The reaction tolerated well different functional groups on the *para*-position of the phenyl ring, including a nitrile (3f), ketone(3g), or ester (3h) moiety, and consistent high yields and ee values were obtained. The substrate bearing a cyclopropyl group (3i) was well-tolerated, so was the azide containing a free hydroxyl group (3j). Benzyl azides bearing a *meta*-substituted phenyl ring were next examined, and all the substituents were found to be compatible, including halogen atoms (3k–3m), a cyano group (3n), an ester function (3o), and a simple phenyl group (3p). Benzyl azides with an *ortho*-substituted phenyl ring were also tested, and the corresponding products were obtained in excellent yields with high ee values (3q and 3r). Moreover, benzyl azides bearing a disubstituted phenyl ring were evaluated under the reaction conditions. Benzyl azides with a 3,5-dimethyl phenyl group and a 3,4-dimethyl phenyl group were excellent substrates, forming the desired products in high yields with excellent enantioselectivities (3s and 3t). Azides containing a mono-bromo or di-bromo-substituted phenyl moiety were found to be suitable, and the cycloaddition products were formed in good yields and excellent ee values (3u and 3v), with bromo atoms serving as an excellent synthetic handle for further synthetic transformations. When the azides containing a fused ring system were subjected to the reaction conditions, products bearing bulky naphthyl groups were prepared in high yields and over 90% ee values (3w–3y). Moreover, azides with a simple alkyl substituent also underwent the CuAAC reaction smoothly, furnishing the triazole product in 83% yield with 85% ee (3z). It is noteworthy that a triazole ring is formed in the above atroposelective synthesis, which represents an interesting entry of triazole-containing atropisomers in biological sciences, medicinal chemistry, and drug discovery. The absolute configurations of the triazole products were assigned on the basis of the X-ray crystallographic analysis of 3w.

**Scheme 3:**
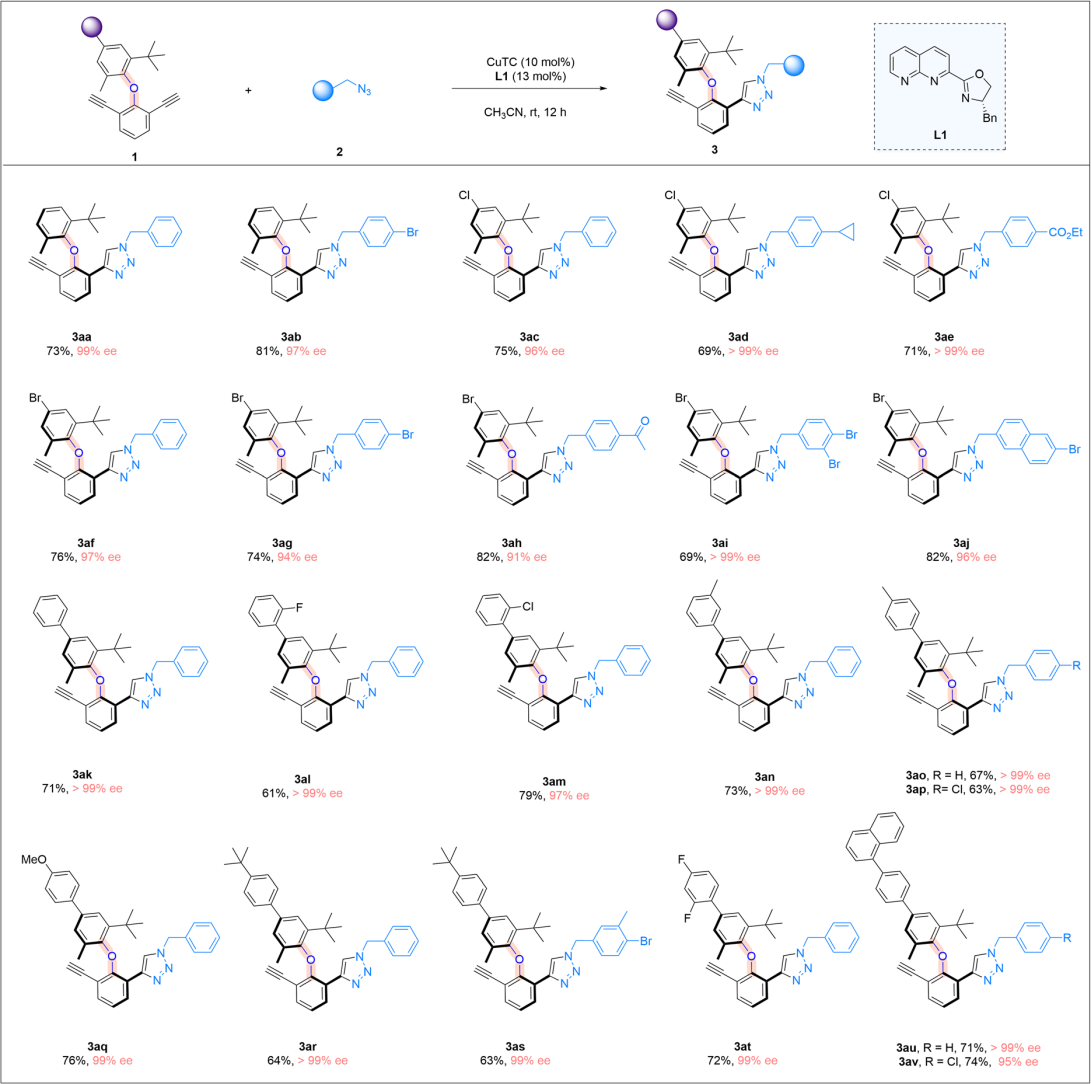
Further reaction scope. Reaction conditions: 1 (0.05 mmol), 2 (0.09 mmol), CuTC (10 mol%), and L1 (13 mol%) in CH_3_CN (2.0 ml) at room temperature for 12 h; yields refer to isolated yields.

We further examine the generality of the reaction by exploring the utilization of different diaryl ethers, focusing on varying the structure of the non-alkynyl-substituted aryl moiety (Scheme 3). Simple diaryl ethers with a phenyl ring bearing a methyl and a *tert*-butyl group at the *ortho*-positions were good substrates (3aa and 3ab). The presence of a chlorine or a bromine atom in the aryl moiety was well-tolerated, and the corresponding cycloaddition products were consistently obtained in high yields and with excellent enantiomeric excesses (3ac–3aj). We next introduced an aryl substituent into the 2-methyl-6-*tert*-butyl aryl moiety of the diaryl ethers, and different aryl moieties bearing different substituents, ranging from halogens to a methyl or a methoxyl group, with different substitution patterns, were all found to be suitable substrates, and the corresponding triazole products were obtained in good yields and with very high enantiomeric excesses (3ak–3av). The limitation of the current study lies in the necessity of having an *ortho-t*-butyl group in the C–O axially molecular structures, which is required for the introduction of a sufficient rotational barrier.

**Scheme 4:**
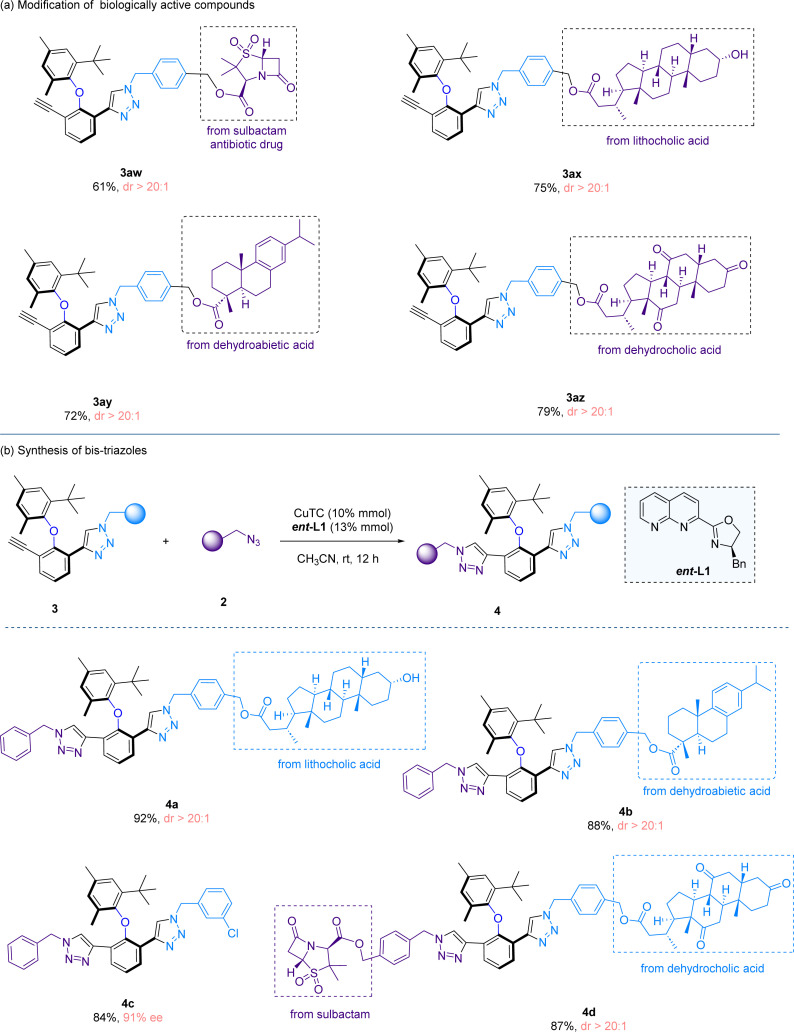
Synthetic applications. (a) Modification of biologically active compounds. (b) Synthesis of bis-triazoles.

To demonstrate the utility of our method, we performed the late-stage functionalization of several natural products and drugs (Scheme 4a), which include sulbactam, lithocholic acid, dehydroabietic acid and dehydrocholic acid, and all the reactions proceeded with high yields and excellent diastereoselectivities (3aw–3az). After the first CuAAC reaction, a triple bond remains in the cycloaddition products. Therefore, we sought to perform a second CuAAC reaction to form bis-triazole scaffolds, which represent an interesting molecular architecture (Scheme 4b). Towards this end, the first CuAAC reaction product 3 and benzyl azide 2 were subjected to the second CuAAC reaction, under the catalysis of CuTC and the *ent*-L1 ligand. The reason of utilizing another enantio-isomer *ent*-L1 to promote the second cycloaddition is due to the matching of chirality, as L1 only promotes the reaction on the first triple bond while *ent*-L1 is effective in catalyzing the cycloaddition reaction at the second triple bond site. The second CuAAC reaction would not proceed in the presence of L1 or without a ligand. The second CuAAC reaction is compatible with the late-stage functionalization reactions (4a and 4b). Notably, a bis-triazole compound bearing two complex natural product and drug moieties was smoothly synthesized (4d), showcasing that our synthetic protocols are mild and highly suitable for the late-stage derivatization of complex natural products and drug-like molecules.

**Scheme 5:**
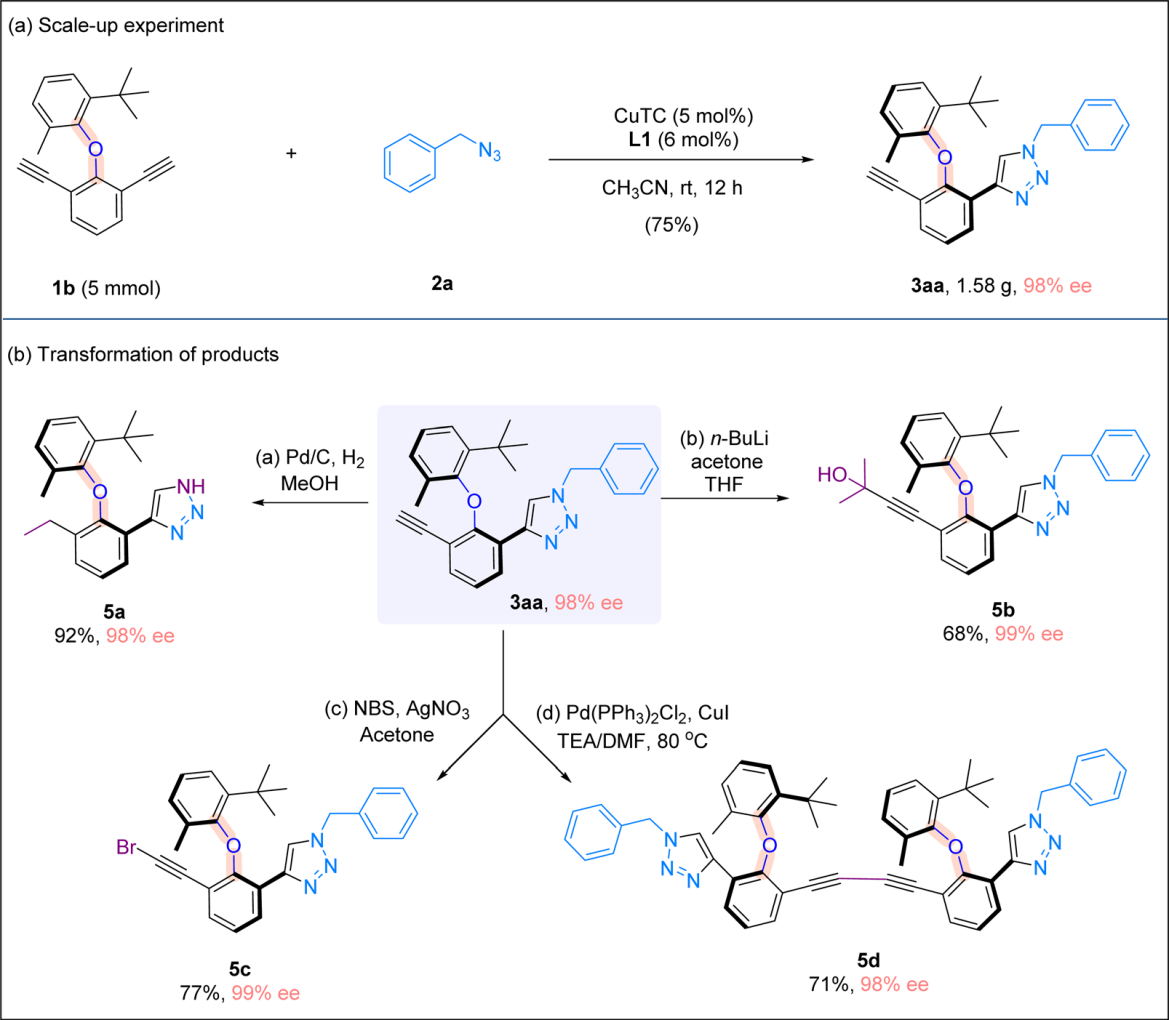
Further synthetic applications. (a) Scale-up experiment. (b) Transformation of products.

To illustrate the practicality of our method, a scale-up experiment on a 5 mmol scale was carried out, and the desired product 3aa was obtained in 75% yield and with 98% ee (Scheme 5a). When product 3aa was treated with hydrogen under Pd/C, free triazole 5a bearing a saturated ethyl group was obtained in 92% yield. The terminal alkynyl moiety in 3aa could be easily manipulated, and the treatment with NBS or *n*-BuLi/acetone formed bromide 5c or propargylic alcohol 5b, respectively. Interestingly, when alkynyl triazole 3aa was subjected to a homocoupling reaction in the presence of a Pd/Cu catalyst, a dimeric product bearing bis-alkynyl moieties and two triazole rings was formed in good yield without any erosion of enantiomeric excess (Scheme 5b).

**Scheme 6:**
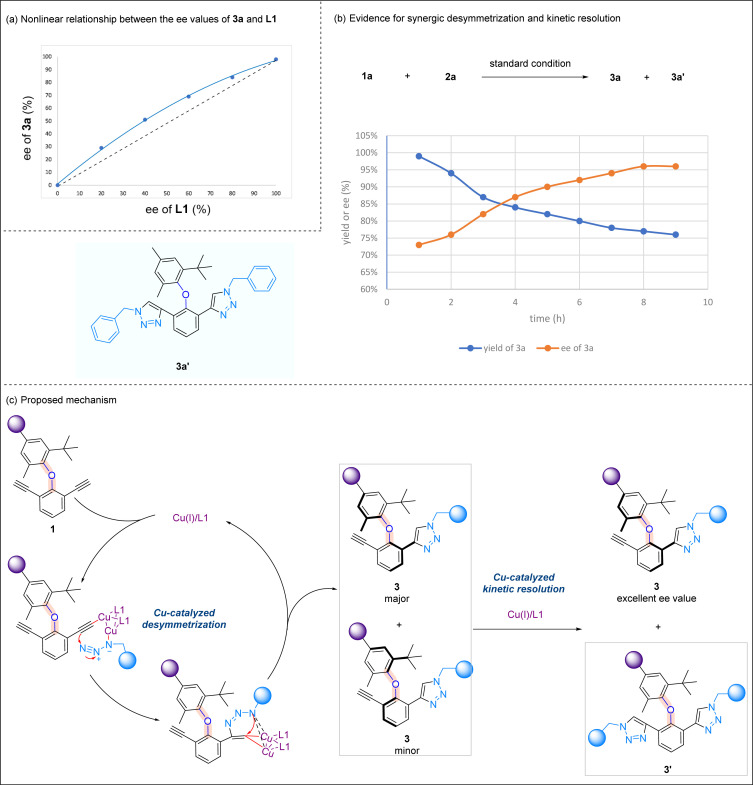
Mechanistic studies. (a) Nonlinear relationship between the ee values of 3a and L1; (b) evidence for synergic desymmetrization and kinetic resolution. (c) Proposed mechanism.

Preliminary mechanistic investigations were performed to gain some insights into the reaction mechanism. A positive nonlinear effect^16^ between the ee values of optically active ligand L1 and product 3a was observed, suggesting that the mononuclear copper catalyst may not be active catalytic species in this asymmetric CuAAC reaction (Scheme 6a). The yields and ee values of product 3a were plotted over the time, and it was noted that bis-triazole product 3a′ was accumulated during the reaction process. The initial high yield of 3a is correlated to a low ee value, and the yield of 3a is then decreased, which is coupled with an increase in the ee value. At the end of the reaction, the relatively low yield of 3a is associated with the highest ee value of 3a (Scheme 6b). Based on known literature studies on the mechanism of CuAAC reactions,^17^ and in light of the above mechanistic experiments we performed, a plausible reaction mechanism is proposed (Scheme 6c). In the presence of a Cu/1,8-naphthyridine-based ligand, diaryl ether undergoes desymmetrization, forming Cu(i) acetylide at one alkynyl site. The subsequent cycloaddition reaction yields triazole 3, and a key kinetic resolution process takes place here. While the minor isomer of 3 undergoes another CuAAC to yield bis-triazole product 3′, the enantiomeric excess of the major isomer of 3 increases during the reaction progress and reaches the highest when the minor isomer of 3 fully converts to a bis-triazole product. This proposal is consistent with our earlier experiment whereby *ent*-L1 promoted the second CuAAC reaction of the major isomer of 3 to form bis-triazole 4 (Scheme 4b).

## Conclusions

In summary, through the introduction of a novel chiral 1,8-naphthyridine based ligand, we developed a copper-catalyzed CuAAC reaction for the atroposelective synthesis of C–O axially chiral compounds. Utilizing readily available dialkynyl diaryl ethers and benzyl azides, this operationally simple reaction proceeded under mild reaction conditions, leading to the formation of a wide range of chiral diaryl ethers possessing two C–O axes in a highly enantioselective manner. In our mechanistic proposal, the synergistic interplay of desymmetrization and kinetic resolution is uncommon for an asymmetric CuAAC reaction. We are confident that the mechanistic insights we gained in this study, together with the introduction of the copper/1,8-naphthyridine based ligand catalytic system, may pave the way to the discovery of more asymmetric CuAAC reactions in the future.

## Data availability

All the data supporting this study are included in the main text and the ESI.†

## Author contributions

L. D. designed and carried out the experiments. X. Z., J. G. and Q. H. participated in the synthesis of substrates. L. D. and Y. L. conceived the project and wrote the manuscript. Y. L. supervised the project.

## Conflicts of interest

There are no conflicts to declare.

## Supplementary Material

SC-015-D4SC01074D-s001

SC-015-D4SC01074D-s002
