# Supplementary material for: Copper-catalyzed atroposelective synthesis of C–O axially chiral compounds enabled by chiral 1,8-naphthyridine based ligands
Source: Chem Sci. 2024 Mar 22;15(16):5993–6001. doi: 10.1039/d4sc01074d (PMC11040657; doi:10.1039/d4sc01074d)
Supplement: SC-015-D4SC01074D-s001 [file SC-015-D4SC01074D-s001.pdf]

Electronic Supplementary Material (ESI) for Chemical Science.
This journal is © The Royal Society of Chemistry 2024

Electronic Supplementary Information

Copper-catalyzed atroposelective synthesis of C–O axially chiral compounds enabled by chiral 1,8-naphthyridine based ligands

Lei Dai,[ac] Xueting Zhou,[ab] Jiami Guo,[ab] Qingqin Huang[ab] and Yixin Lu*[ab]

[a]Department of Chemistry, National University of Singapore, 3 Science Drive 3, Singapore, 117543, Singapore.

[b]Joint School of National University of Singapore and Tianjin University, International Campus of Tianjin University, Binhai New City, Fuzhou, Fujian, 350207, China.

[c]Chongqing Key Laboratory of Natural Product Synthesis and Drug Research, School of Pharmaceutical Sciences, Chongqing University, Chongqing, 401331, China.

*Corresponding author: Y. Lu, Email: chmlyx@nus.edu.sg

Contents

1. Materials and Methods 3

2. Synthesis of Chiral 1,8-Naphthyridine Based Ligands 3

3. Synthesis of Substrates 6

4. General Procedure for the Asymmetric CuAACs 12

5. Analytical Data and HPLC Chromatograms of the Products **3**. 13

6. Synthetic Applications 61

7. Mechanistic Studies 72

8. Single Crystal Structure X-ray Analysis of **3w** 75

9. NMR Spectra 92

10. References 168

1. Materials and Methods

All starting materials were obtained from commercial suppliers (Sigma Aldrich and TCI) and directly used without further purification unless otherwise stated. All reactions were carried out under argon atmosphere with magnetic stirring. Substrates were synthesized according to literatures.[1-6] CuTC was purchased from TCI.

Analytical thin layer chromatography was carried out with silica gel pre-coated glass plates (TLC-Silica gel GF254, coating thickness: 0.25 mm) purchased from Merck. Visualization was accomplished with short wave UV light (254nm, 365nm) and/or 10% phosphomolybdic acid in ethanol or KMnO4 staining solutions followed by heating. Column chromatography was performed on silica gel 200~300 mesh. ^{1}H NMR and ^{13}C NMR spectra were recorded on a Bruker AV-III400 (400 MHZ) spectrometer. Chemical shifts were calibrated using residual solvent as an internal reference ($CDCl_3$: 7.18 ppm ^{1}H NMR, 77.00 ppm ^{13}C NMR). ^{1}H NMR Spectroscopy splitting patterns were designated as singlet (s), doublet (d), triplet (t), quartet (q). Splitting patterns that could not be interpreted or easily visualized were designated as multiplet (m) or broad (br). All high-resolution mass spectra (HRMS) were obtained on a Finnigan/MAT 95XL-T spectrometer, the calculated values are based on the most abundant isotope. Chiral HPLC analyses were performed on an Agilent 1100 Series using a Daicel Chiralpak column (IC and IE) with hexanes/iPrOH as the eluent.

2. Synthesis of Chiral 1,8-Naphthyridine Based Ligands

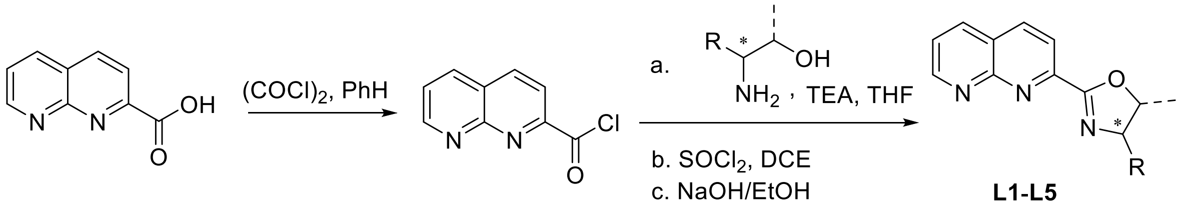

To a suspension of 1,8-naphthyridine-2-carboxylic acid (2 g, 11 mmol) in dry benzene (60 mL), was added oxalyl chloride (1.4 mL, 17 mmol) drop wisely at 0 °C. Then three drops of DMF were added. It was then slowly brought to room temperature and heated at 65 °C for 3 h until the gas evolution subsided. The solvent and excess oxalyl chloride was removed under reduced pressure to afford the derided products, which was directly used for the next step without further purification.

Chiral amino alcohol (11 mmol) was dissolved in dry THF (120 mL) and the solution was chilled to 0 °C. Then, triethylamine (3.9 mL, 0.028 mol) was added to the solution. Subsequently, a suspension of 1,8-naphthyridine-2-carbonyl chloride in dry THF was added portion wise during 30 min. Then the mixture was slowly brought to room temperature and stirred at room temperature for additional 24 h. After that it was evaporated, taken in dichloromethane and extracted with saturated aqueous sodium bicarbonate solution. After further extraction of the aqueous phase with dichloromethane, the combined organic phases were dried over anhydrous Na_2SO_4 and evaporated again to get a brown residue. The residue was then purified by silica gel column chromatography using ethyl acetate/hexane as eluent to obtain the corresponding products. To a solution of above-mentioned product (6.8 mmol) in dry DCE (60 mL), $SOCl_2$ (5 mL, 68.2 mmol) was added drop wise at room temperature and the resulting mixture was refluxed for 3 h until the gas evolution subsided. Then, it was cooled to room temperature and solvent and excess $SOCl_2$ were removed under reduced pressure. The residue was taken in dichloromethane and extracted cautiously with saturated aqueous solution of Na_2CO_3. The combined organic phase was dried over anhydrous Na2SO4 and evaporated to obtain a brown residue which was purified by silica gel column chromatography using 50% ethyl acetate/petroleum ether as eluent. To a solution of above-mentioned product (5.1 mmol) in dry ethanol (50 mL), 5.6 mL of 1N ethanolic NaOH (5.6 mmol) solution was added drop wise and the solution was refluxed for 3 h under N_2 atmosphere. Then the solvent was removed by rotary evaporation and the residue was passed through a column packed with silica gel using 5% MeOH/DCM as eluent. Chiral ligands (**L1**-**L5**) were obtained.

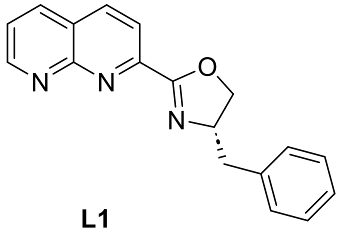

L1

(*S*)-4-Benzyl-2-(1,8-naphthyridin-2-yl)-4,5-dihydrooxazole (L1)

79% yield; $[\alpha]_D^{25}$ = -12.4 (c 0.5, $CHCl_3$), a white solid. ^{1}H NMR (400 MHz, $CDCl_3$) δ 9.20 (d, *J* = 2.4 Hz, 1H), 8.48 – 8.18 (m, 3H), 7.55 (dd, *J* = 8.1, 4.2 Hz, 1H), 7.41 – 7.18 (m, 5H), 4.83 – 4.67 (m, 1H), 4.55 (t, *J* = 9.1 Hz, 1H), 4.33 (t, *J* = 8.2 Hz, 1H), 3.30 (dd, J = 13.8, 5.4 Hz, 1H), 2.84 (dd, J = 13.8, 8.6 Hz, 1H). ^{13}C NMR (101 MHz, $CDCl_3$) δ 163.4, 155.4, 154.5, 149.9, 137.9, 137.7,

136.7, 129.3, 128.6, 126.7, 123.6, 123.2, 121.9, 72.7, 68.3, 41.7. HRMS (ESI) m/z calcd for $C_{18}H_{16}N_3O$ $[M+H]^+$ = 290.1288, found = 290.1290.

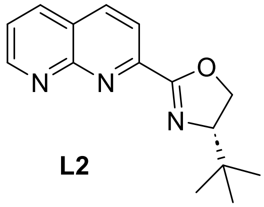

L2

(*S*)-4-(*tert*-Butyl)-2-(1,8-naphthyridin-2-yl)-4,5-dihydrooxazole (L2)

74% yield; $[\alpha]_D^{25}$ = -119.2 (c 0.5, $CHCl_3$), a white solid. ^{1}H NMR (400 MHz, $CDCl_3$) δ 9.19 (dd, *J* = 4.2, 2.0 Hz, 1H), 8.35 (d, *J* = 8.3 Hz, 1H), 8.27 (d, *J* = 8.4 Hz, 1H), 8.23 (dd, *J* = 8.2, 2.0 Hz, 1H), 7.54 (dd, *J* = 8.1, 4.2 Hz, 1H), 4.54 (dd, *J* = 10.3, 8.8 Hz, 1H), 4.40 (t, *J* = 8.6 Hz, 1H), 4.19 (dd, *J* = 10.3, 8.4 Hz, 1H), 1.01 (s, 9H). ^{13}C NMR (101 MHz, $CDCl_3$) δ 162.8, 155.4, 154.4, 150.1, 137.7, 136.6, 123.6, 123.0, 122.0, 76.7, 69.6, 34.1, 26.0. HRMS (ESI) m/z calcd for $C_{15}H_{18}N_3O$ $[M+H]^+$ = 256.1444, found = 256.1448.

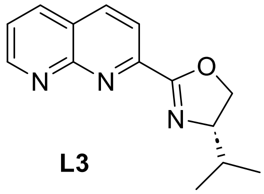

L3

(*S*)-4-Isopropyl-2-(1,8-naphthyridin-2-yl)-4,5-dihydrooxazole (L3)

84% yield; $[\alpha]_D^{25}$ = -92.6 (c 0.5, $CHCl_3$), a white solid. ^{1}H NMR (400 MHz, $CDCl_3$) δ 9.11 (dd, *J* = 4.2, 2.0 Hz, 1H), 8.48 – 8.13 (m, 3H), 7.49 (ddd, *J* = 18.6, 8.1, 4.2 Hz, 1H), 4.61 – 3.75 (m, 3H), 2.12 – 1.76 (m, 1H), 1.06 – 0.87 (m, 6H). ^{13}C NMR (101 MHz, $CDCl_3$) δ 162.9, 155.4, 154.5, 154.4, 150.1, 138.9, 137.8, 137.3, 136.7, 123.6, 123.2, 123.1, 122.0, 120.3, 71.0, 58.1, 32.9, 19.0, 18.3. HRMS (ESI) m/z calcd for $C_{14}H_{16}N_3O$ $[M+H]^+$ = 242.1288, found = 242.1289.

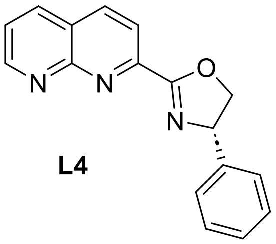

L4

(*S*)-2-(1,8-Naphthyridin-2-yl)-4-phenyl-4,5-dihydrooxazole (L4)

65% yield; $[\alpha]_D^{25}$ = -151.0 (c 0.5, $CHCl_3$), a white solid. ^{1}H NMR (400 MHz, $CDCl_3$) δ 9.14 (dd, *J* = 4.2, 2.0 Hz, 1H), 8.33 (d, *J* = 8.4 Hz, 1H), 8.23 (d, *J* = 8.4 Hz, 1H), 8.17 (dd, *J* = 8.2, 2.0 Hz, 1H), 7.49 (dd, *J* = 8.2, 4.2 Hz, 1H), 7.29 (s, 5H), 5.45 (dd, *J* = 10.4, 8.6 Hz, 1H), 4.91 (dd, *J* = 10.4, 8.6 Hz, 1H), 4.40 (t, *J* = 8.6 Hz, 1H). ^{13}C NMR (101 MHz, $CDCl_3$) δ 164.2, 155.4, 154.5, 149.8, 141.7, 138.0, 136.7, 128.9, 128.9, 127.9, 127.1, 126.9, 123.7, 123.2, 122.1, 75.6, 70.6. HRMS (ESI) m/z calcd for $C_{17}H_{14}N_3O$ $[M+H]^+$ = 276.1131, found = 276.1132.

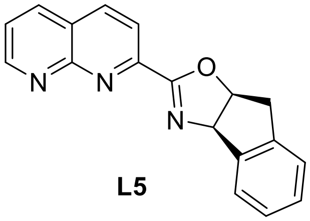

L5

(*3aR*, *8aS*)-2-(1,8-Naphthyridin-2-yl)-3a,8a-dihydro-8*H*-indeno[1,2-d]oxazole (L5)

68% yield; $[\alpha]_D^{25}$ = +395.0 (c 0.5, $CHCl_3$), a white solid. ^{1}H NMR (400 MHz, $CDCl_3$) δ 9.08 (dd, *J* = 4.2, 2.0 Hz, 1H), 8.25 – 8.07 (m, 3H), 7.55 – 7.48 (m, 1H), 7.44 (dd, *J* = 8.1, 4.2 Hz, 1H), 7.24 – 7.12 (m, 4H), 5.79 (d, *J* = 8.0 Hz, 1H), 5.59 (dt, *J* = 8.2, 4.3 Hz, 1H), 3.47 (d, *J* = 4.3 Hz, 2H). ^{13}C NMR (101 MHz, $CDCl_3$) δ 163.5, 155.4, 154.4, 150.0, 141.4, 140.1, 137.7, 136.6, 128.7, 127.5, 125.6, 125.5, 123.5, 123.1, 122.1, 84.2, 77.3, 39.8. HRMS (ESI) m/z calcd for $C_{18}H_{14}N_3O$ $[M+H]^+$ = 288.1131, found = 288.1131.

3. Synthesis of Substrates

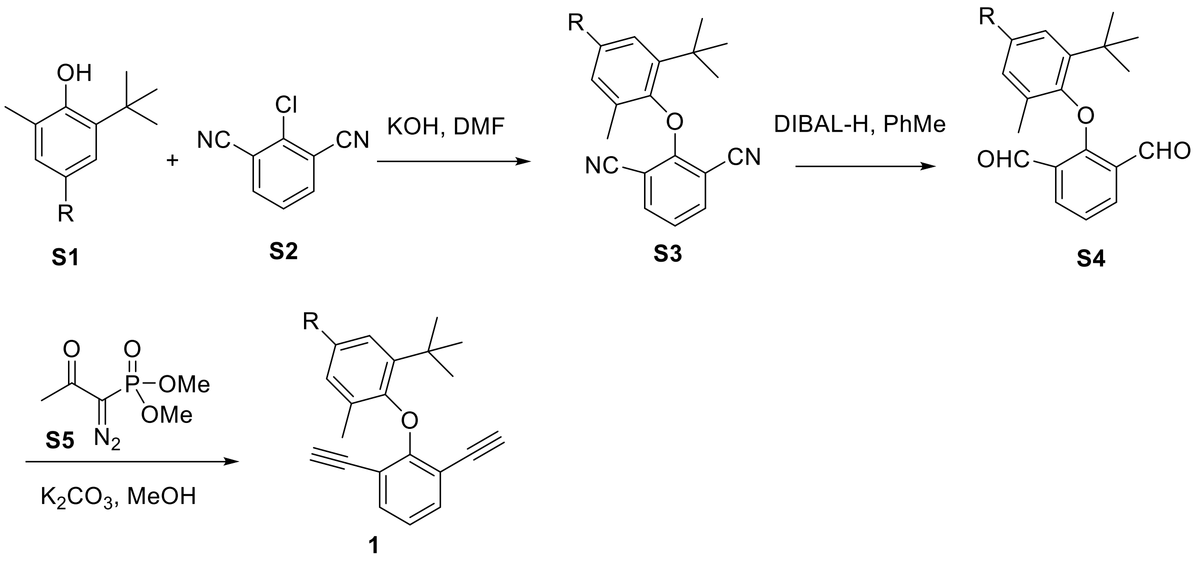

The mixture of phenol **S1** (10 mmol, 1 equiv) and KOH (10 mmol, 1 equiv) in toluene (30 mL) in egg-plant bottle installed with water separator was stirred at 130 °C for 4 h. After removal of the solvent of toluene under reduced pressure, it was added 2-chloro-1,3-dicyanobenzene **S2** (10vmmol, 1 equiv) and anhydrous DMF (60 mL). The reaction mixture was stirred under N_2 at 150 °C for 16 h. Solvent were removed under reduced pressure, and the residue was extracted with EA for 3 times. The combined organics was washed with water for 3 times, dried over Na_2SO_4, and solvent removed under reduced pressure. The residue was purified by flash column chromatography (PE/EA = 10:1) to yield 2-aryloxy-1,3-dicyanobenzene **S3**.

To the solution of 2-aryloxy-1,3-dicyanobenzene **S3** was slowly added DIBAL-H (1.5M solution in toluene, 2.5 equiv) in anhydrous toluene (30 mL) under N_2 at -78 °C and then stirred for 1h at this temperature. The reaction mixture was allowed to warm to room temperature and stirred 16 h. The reaction mixture was cooled to 0 °C and added slowly 5M HCl. After stirring for 2h, the mixture was extracted 3 times with EA and the combined organics was washed with brine. The organics was dried over Na_2SO_4, and solvent was removed under reduced pressure. The residue was purified by flash column chromatography (PE/EA = 50:1) to yield 2-aryloxyisophthalaldehydes **S4**.

To a stirred solution of **S4** (5 mmol, 1.0 equiv.) and K_2CO_3 (20 mmol, 4.0 equiv.) in MeOH (20 mL), **S5** (15 mmol, 3.0 equiv.) was added at room temperature. After the completion of the reaction, the reaction mixture was quenched with water. The reaction mixture was then diluted with EA. The separated organic layer was washed with brine, dried over Na_2SO_4, filtered and concentrated under reduced pressure. The residue was purified by column chromatography to obtain **1**.

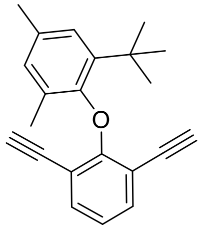

1a

1-(*tert*-Butyl)-2-(2,6-diethynylphenoxy)-3,5-dimethylbenzene (1a)

58% yield; a white solid. ^{1}H NMR (400 MHz, $CDCl_3$) δ 7.34 (d, *J* = 7.7 Hz, 2H), 6.88 (d, *J* = 2.0 Hz, 1H), 6.81 (t, *J* = 7.7 Hz, 1H), 6.70 – 6.64 (m, 1H), 2.90 (s, 2H), 2.23 (s, 3H), 1.85 (s, 3H), 1.32 (s, 9H). ^{13}C NMR (101 MHz, $CDCl_3$) δ 159.4, 151.1, 141.8, 135.7, 133.8, 130.5, 128.8, 125.0, 120.9, 111.9, 82.5, 78.7, 35.0, 30.7, 21.1, 17.2. HRMS (ESI) m/z calcd for $C_{22}H_{23}O$ $[M+H]^+$ = 303.1743, found = 303.1747.

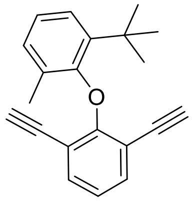

1b

1-(*tert*-Butyl)-2-(2,6-diethynylphenoxy)-3-methylbenzene (1b)

64% yield; a white solid. ^{1}H NMR (400 MHz, $CDCl_3$) δ 7.35 (d, *J* = 7.7 Hz, 2H), 7.08 (dd, *J* = 7.9, 1.7 Hz, 1H), 6.95 (t, *J* = 7.6 Hz, 1H), 6.89 – 6.79 (m, 2H), 2.90 (s, 2H), 1.88 (s, 3H), 1.33 (s, 9H). ^{13}C NMR (101 MHz, $CDCl_3$) δ 159.2, 153.4, 142.2, 135.7, 130.9, 128.5, 124.6, 124.3, 121.1, 111.9, 82.5, 78.6, 35.1, 30.6, 17.3. HRMS (ESI) m/z calcd for $C_{21}H_{21}O$ $[M+H]^+$ = 289.1587, found = 289.1585.

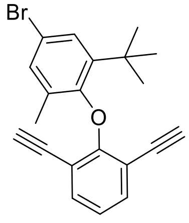

1c

5-Bromo-1-(*tert*-butyl)-2-(2,6-diethynylphenoxy)-3-methylbenzene (1c)

73% yield; a white solid. ^{1}H NMR (400 MHz, $CDCl_3$) δ 7.35 (d, J = 7.7 Hz, 2H), 7.20 (d, J = 2.4 Hz, 1H), 7.02 (d, J = 2.2 Hz, 1H), 6.84 (t, J = 7.7 Hz, 1H), 2.96 (s, 2H), 1.85 (s, 3H), 1.31 (s, 9H). ^{13}C NMR (101 MHz, $CDCl_3$) δ 158.5, 152.4, 144.5, 135.7, 133.1, 130.9, 127.5, 121.5, 117.4, 111.9, 83.0, 78.4, 35.4, 30.4, 17.2. HRMS (ESI) m/z calcd for $C_{21}H_{20}BrO$ $[M+H]^+$ = 367.0692, found = 367.0697.

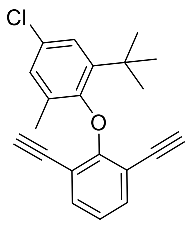

1d

1-(*tert*-Butyl)-5-chloro-2-(2,6-diethynylphenoxy)-3-methylbenzene (1d)

76% yield; a white solid. ^{1}H NMR (400 MHz, $CDCl_3$) δ 7.36 (d, J = 7.7 Hz, 2H), 7.06 (d, J = 2.6 Hz, 1H), 6.89 – 6.85 (m, 1H), 6.83 (d, J = 7.7 Hz, 1H), 2.95 (s, 2H), 1.85 (s, 3H), 1.31 (s, 9H). ^{13}C NMR (101 MHz, $CDCl_3$) δ 158.6, 151.8, 144.1, 135.7, 132.6, 129.5, 127.9, 124.6, 121.5, 111.9, 83.0, 78.5, 35.4, 30.4, 17.3. HRMS (ESI) m/z calcd for $C_{21}H_{20}ClO$ $[M+H]^+$ = 323.1197, found = 323.1194.

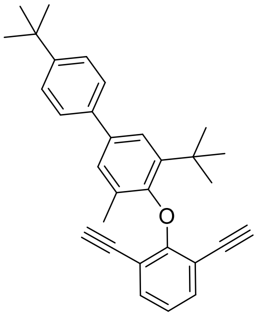

1e

3,4'-Di-*tert*-butyl-4-(2,6-diethynylphenoxy)-5-methyl-1,1'-biphenyl (1e)

54% yield; a white solid. ^{1}H NMR (400 MHz, $CDCl_3$) δ 7.47 (d, J = 8.4 Hz, 2H), 7.42 – 7.31 (m, 5H), 7.12 – 7.09 (m, 1H), 6.83 (t, J = 7.7 Hz, 1H), 2.90 (s, 2H), 1.94 (s, 3H), 1.37 (s, 9H), 1.29 (s, 9H). ^{13}C NMR (101 MHz, $CDCl_3$) δ 159.2, 152.8, 149.9, 142.4, 137.3, 135.7, 131.1, 126.9, 126.66, 125.7, 123.3, 121.2, 112.0, 82.7, 78.7, 35.3, 34.5, 31.4, 30.7, 17.5. HRMS (ESI) m/z calcd for $C_{31}H_{33}O$ $[M+H]^+$ = 421.2526, found = 421.2526.

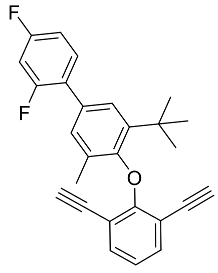

1f

3'-(*tert*-Butyl)-4'-(2,6-diethynylphenoxy)-2,4-difluoro-5'-methyl-1,1'-biphenyl (1f)

46% yield; a white solid. ^{1}H NMR (400 MHz, $CDCl_3$) δ 7.37 (d, *J* = 7.7 Hz, 2H), 7.32 (td, *J* = 8.6, 6.4 Hz, 1H), 7.20 (d, *J* = 2.0 Hz, 1H), 6.99 (t, *J* = 1.8 Hz, 1H), 6.91 – 6.78 (m, 3H), 2.94 (s, 2H), 1.93 (s, 3H), 1.37 (s, 9H). ^{13}C NMR (101 MHz, $CDCl_3$) δ 159.0, 142.3, 135.7, 131.2, 131.0, 128.9, 125.2, 121.3, 112.0, 111.4, 82.7, 78.6, 35.2, 30.6, 17.4. HRMS (ESI) m/z calcd for $C_{27}H_{23}F_2O$ $[M+H]^+$ = 401.1711, found = 401.1716.

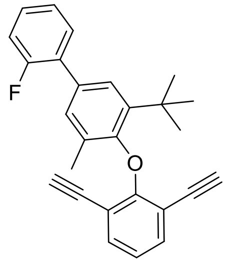

1g

3'-(*tert*-Butyl)-4'-(2,6-diethynylphenoxy)-2-fluoro-5'-methyl-1,1'-biphenyl (1g)

57% yield; a white solid. ^{1}H NMR (400 MHz, $CDCl_3$) δ 7.37 (d, *J* = 7.7 Hz, 2H), 7.26 (s, 1H), 7.16 – 7.03 (m, 3H), 6.84 (t, *J* = 7.7 Hz, 1H), 2.94 (s, 2H), 1.94 (s, 3H), 1.37 (s, 9H). ^{13}C NMR (101 MHz, $CDCl_3$) δ 153.0, 142.2, 135.7, 132.0, 130.9, 130.7, 128.6, 125.4, 124.23, 124.19, 121.3, 115.9, 112.0, 82.8, 78.6, 35.2, 30.6, 17.4. HRMS (ESI) m/z calcd for $C_{27}H_{24}FO$ $[M+H]^+$ = 383.1806, found = 383.1809.

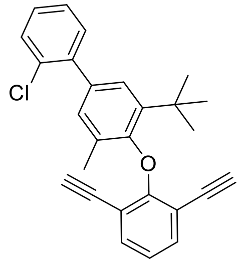

1h

3'-(*tert*-Butyl)-2-chloro-4'-(2,6-diethynylphenoxy)-5'-methyl-1,1'-biphenyl (1h)

57% yield; a white solid. ^{1}H NMR (400 MHz, $CDCl_3$) δ 7.39 (dd, *J* = 10.1, 7.7 Hz, 3H), 7.32 – 7.20 (m, 3H), 7.17 (d, *J* = 2.2 Hz, 1H), 6.97 – 6.92 (m, 1H), 6.84 (t, *J* = 7.7 Hz, 1H), 2.98 (s, 2H), 1.94 (s, 3H), 1.37 (s, 9H). ^{13}C NMR (101 MHz, $CDCl_3$) δ 152.8, 141.8, 140.8, 135.6, 132.6, 131.4, 130.6, 129.9, 129.3, 128.3, 126.8, 125.9, 121.2, 112.0, 82.9, 78.6, 35.2, 30.6, 17.4. HRMS (ESI) m/z calcd for $C_{27}H_{24}ClO$ $[M+H]^+$ = 399.1510, found = 399.1510.

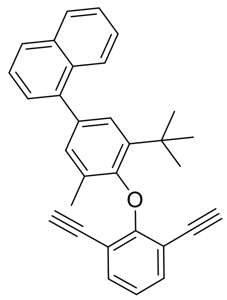

1i

1-(3-(*tert*-Butyl)-4-(2,6-diethynylphenoxy)-5-methylphenyl)naphthalene (1i)

47% yield; a white solid. ^{1}H NMR (400 MHz, $CDCl_3$) δ 7.90 (d, *J* = 8.3 Hz, 1H), 7.84 (d, *J* = 7.6 Hz, 1H), 7.78 (d, *J* = 8.2 Hz, 1H), 7.49 – 7.35 (m, 6H), 7.23 (d, *J* = 2.0 Hz, 1H), 7.03 (s, 1H), 6.87 (t, *J* = 7.7 Hz, 1H), 3.01 (s, 2H), 1.97 (s, 3H), 1.39 (s, 9H). ^{13}C NMR (101 MHz, $CDCl_3$) δ 159.1, 140.5, 136.9, 135.8, 133.9, 131.7, 130.6, 130.1, 128.4, 127.4, 126.9, 126.4, 126.0, 125.9, 125.7, 125.4, 121.3, 82.8, 78.8, 35.3, 30.7, 17.4. HRMS (ESI) m/z calcd for $C_{31}H_{27}O$ $[M+H]^+$ = 415.2056, found = 415.2059.

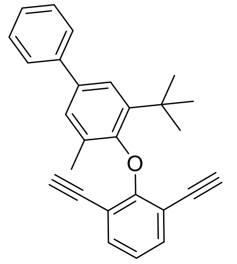

1j

3-(*tert*-Butyl)-4-(2,6-diethynylphenoxy)-5-methyl-1,1'-biphenyl (1j)

78% yield; a white solid. ^{1}H NMR (400 MHz, $CDCl_3$) δ 7.52 (d, *J* = 7.9 Hz, 2H), 7.41 – 7.31 (m, 5H), 7.29 – 7.21 (m, 1H), 7.11 (d, *J* = 1.8 Hz, 1H), 6.84 (t, *J* = 7.7 Hz, 1H), 2.91 (s, 2H), 1.95 (s, 3H), 1.38 (s, 9H). ^{13}C NMR (101 MHz, $CDCl_3$) δ 152.9, 142.4, 141.5, 137.4, 135.7, 131.2, 128.7, 127.1, 127.0, 126.9, 123.4, 121.3, 112.0, 82.8, 78.6, 35.3, 30.6, 17.5. HRMS (ESI) m/z calcd for $C_{27}H_{25}O$ $[M+H]^+$ = 365.1900, found = 365.1904.

4. General Procedure for the asymmetric CuAACs

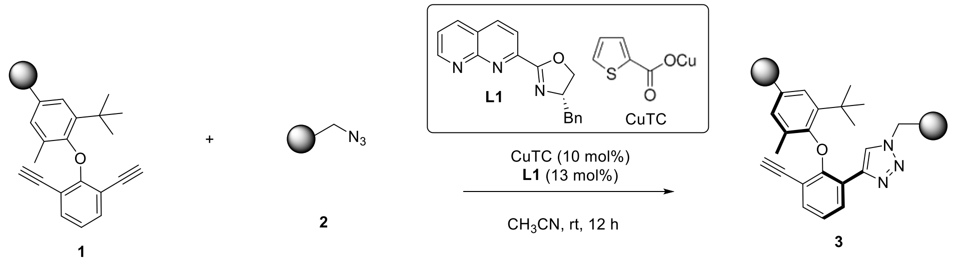

To a dried and argon-filled 10 mL Schlenk tube equipped with a magnetic stir bar was added CuTC (10 mol%) and **L1** (13 mol%) in CH_3CN (1.5 ml). The reaction mixture was stirred under at room temperature for 1 h. Then the solution of **1** (0.05 mmol, 1.0 equiv.) and **2** (0.09 mmol, 1.8 equiv.) in CH_3CN (0.5 mL) was added. The reaction mixture was stirred at room temperature for 12 h. Then the reaction mixture was concentrated under reduced pressure and the residue was purified by column chromatography on silica gel to furnish the product.

5. Analytical Data and HPLC Chromatograms of the Products 3.

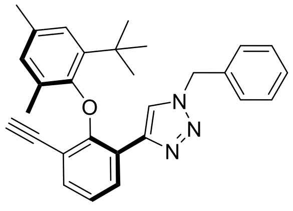

3a

1-Benzyl-4-(2-(2-(tert-butyl)-4,6-dimethylphenoxy)-3-ethynylphenyl)-1*H*-1,2,3-triazole (3a)

76% yield; $[\alpha]_D^{25}$ = -62.0 (c 0.25, $CHCl_3$), a white foam, R_f = 0.32 (hexane/ethyl acetate 7:1). ^1H NMR (400 MHz, $CDCl_3$) δ 8.41 (d, *J* = 9.4 Hz, 1H), 7.97 (s, 1H), 7.30 – 7.21 (m, 4H), 7.18 – 7.11 (m, 2H), 6.99 (t, *J* = 7.7 Hz, 1H), 6.89 (d, *J* = 2.1 Hz, 1H), 6.69 (d, *J* = 2.1 Hz, 1H), 5.59 (d, *J* = 15.0 Hz, 1H), 5.35 (d, *J* = 15.0 Hz, 1H), 2.57 (s, 1H), 2.24 (s, 3H), 1.72 (s, 3H), 1.18 (s, 9H). ^{13}C NMR (101 MHz, $CDCl_3$) δ 154.1, 151.0, 141.9, 135.8, 134.8, 133.9, 130.5, 129.2, 129.1, 129.0, 128.6, 127.8, 125.3, 121.9, 121.6, 110.4, 82.8, 78.5, 54.2, 34.9, 31.0, 21.1, 17.2. HRMS (ESI) m/z calcd for $C_{29}H_{30}N_3O$ $[M+H]^+$ = 436.2383, found = 436.2389; the ee value was 96%, t_R (minor) = 12.5 min, t_R (major) = 16.5 min (Chiralpak IC, λ = 254 nm, 10% *i*-PrOH/Hexane, flow rate = 1.0 mL/min).

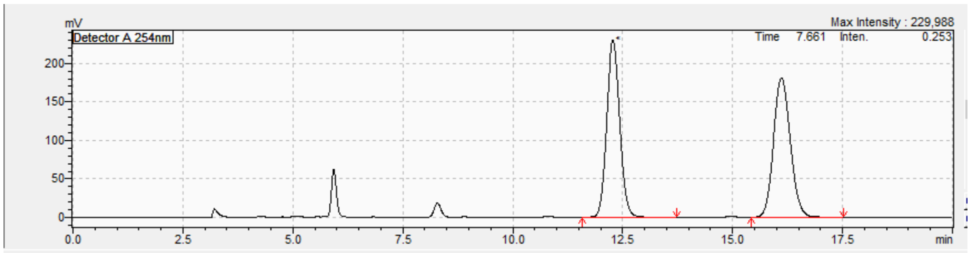

Results View - Peak Table

Peak Table | Compound | Group | Calibration Curve

Peak#	Ret. Time	Area	Height	Mark	Conc.	Unit	ID#	Name
1	12.281	4742165	229690		49.712			
2	16.113	4797122	180935		50.288			
Total		9539287	410625		100.000			

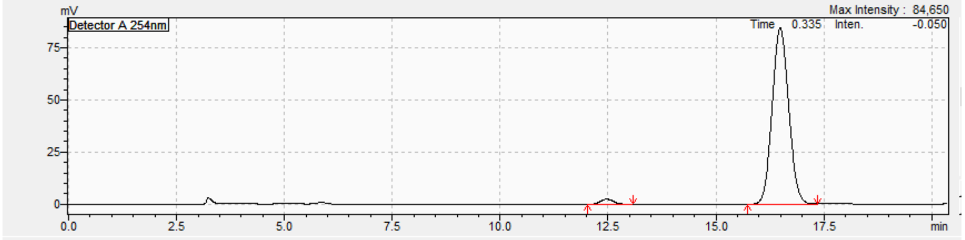

Results View - Peak Table

Peak Table | Compound | Group | Calibration Curve

Peak#	Ret. Time	Area	Height	Mark	Conc.	Unit	ID#	Name
1	12.471	49288	2433		2.141			
2	16.489	2252520	84619		97.859			
Total		2301807	87052		100.000			

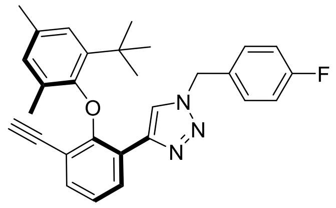

3b

4-(2-(2-(*tert*-Butyl)-4,6-dimethylphenoxy)-3-ethynylphenyl)-1-(4-fluorobenzyl)-1*H*-1,2,3-triazole (3b)

74% yield; $[\alpha]_D^{25}$ = -42.2 (c 0.5, $CHCl_3$), a white foam, R_f = 0.35 (hexane/ethyl acetate 7:1). ^{1}H NMR (400 MHz, $CDCl_3$) δ 8.41 (d, *J* = 7.1 Hz, 1H), 7.96 (s, 1H), 7.25 (d, *J* = 8.4 Hz, 1H), 7.17 – 7.11 (m, 2H), 7.06 – 6.87 (m, 4H), 6.69 (s, 1H), 5.56 (d, *J* = 15.0 Hz, 1H), 5.32 (d, *J* = 15.0 Hz, 1H), 2.58 (s, 1H), 2.24 (s, 3H), 1.73 (s, 3H), 1.19 (s, 9H). ^{13}C NMR (101 MHz, $CDCl_3$) δ 150.9, 141.9, 135.9, 134.0, 130.4, 129.8, 129.7, 129.2, 129.1, 125.4, 122.0, 121.5, 116.1, 115.9, 110.5, 82.9, 78.4, 53.4, 34.9, 31.0, 21.1, 17.2. HRMS (ESI) m/z calcd for $C_{29}H_{29}FN_3O$ $[M+H]^+$ = 454.2289, found = 454.2294; the ee value was 90%, t_R (minor) = 10.8 min, t_R (major) = 14.6 min (Chiralpak IC, λ = 254 nm, 10% *i*-PrOH/Hexane, flow rate = 1.0 mL/min).

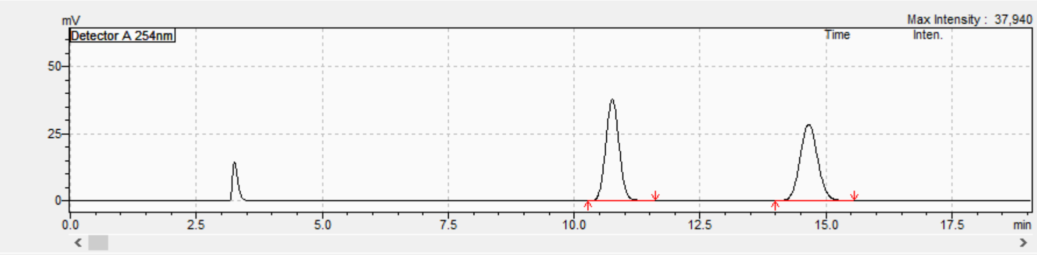

Results View - Peak Table

Peak Table | Compound | Group | Calibration Curve

Peak#	Ret. Time	Area	Height	Mark	Conc.	Unit	ID#	Name	Area%
1	10.757	693794	37855		50.033				50.033
2	14.655	692881	28489		49.967				49.967
Total		1386675	66344		100.000				100.000

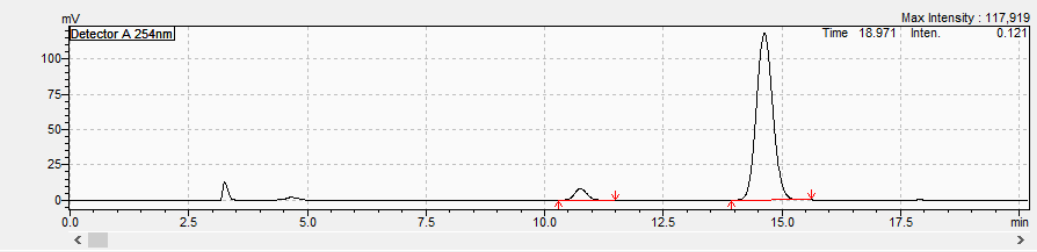

Results View - Peak Table

Peak Table | Compound | Group | Calibration Curve

Peak#	Ret. Time	Area	Height	Mark	Conc.	Unit	ID#	Name	Area%
1	10.756	145348	8034		4.856				4.856
2	14.630	2847814	117774		95.144				95.144
Total		2993161	125808		100.000				100.000

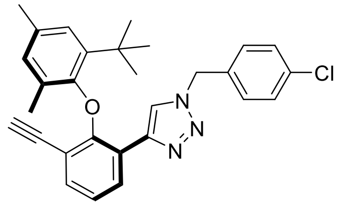

3c

4-(2-(2-(*tert*-Butyl)-4,6-dimethylphenoxy)-3-ethynylphenyl)-1-(4-chlorobenzyl)-1*H*-1,2,3-triazole (3c)

77% yield; $[\alpha]_D^{25}$ = -53.8 (c 0.5, $CHCl_3$), a white foam, R_f = 0.33 (hexane/ethyl acetate 8:1). ^{1}H NMR (400 MHz, $CDCl_3$) δ 8.41 (d, *J* = 7.6 Hz, 1H), 7.96 (s, 1H), 7.31 – 7.18 (m, 3H), 7.09 (d, *J* = 8.5 Hz, 2H), 7.00 (t, *J* = 7.7 Hz, 1H), 6.91 (s, 1H), 6.69 (s, 1H), 5.55 (d, *J* = 15.1 Hz, 1H), 5.32 (d, *J* = 15.1 Hz, 1H), 2.58 (s, 1H), 2.24 (s, 3H), 1.73 (s, 3H), 1.19 (s, 9H). ^{13}C NMR (101 MHz, $CDCl_3$) δ 154.1, 150.9, 141.9, 135.9, 134.7, 134.0, 133.3, 130.4, 129.2, 129.2, 129.2, 129.1, 125.4, 122.0, 121.4, 82.9, 78.4, 53.4, 34.9, 31.0, 21.1, 17.3. HRMS (ESI) m/z calcd for $C_{29}H_{29}ClN_3O$ $[M+H]^+$ = 470.1994, found = 470.1998; the ee value was 95%, t_R (minor) = 11.3 min, t_R (major) = 15.1 min (Chiralpak IC, λ = 254 nm, 10% *i*-PrOH/Hexane, flow rate = 1.0 mL/min).

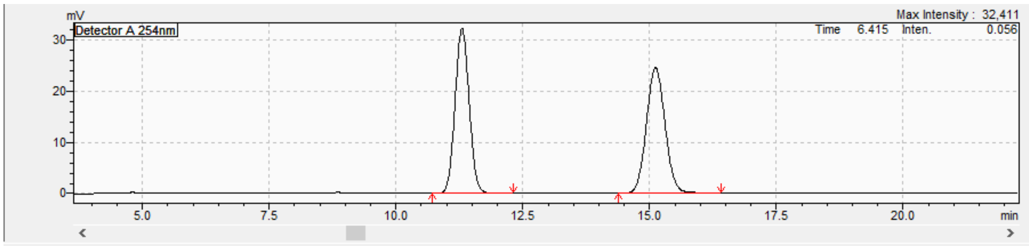

Results View - Peak Table

Peak Table | Compound | Group | Calibration Curve

Peak#	Ret. Time	Area	Height	Mark	Conc.	Unit	ID#	Name	Area%
1	11.302	625103	32379		49.876				49.876
2	15.118	628218	24573		50.124				50.124
Total		1253321	56952		100.000				100.000

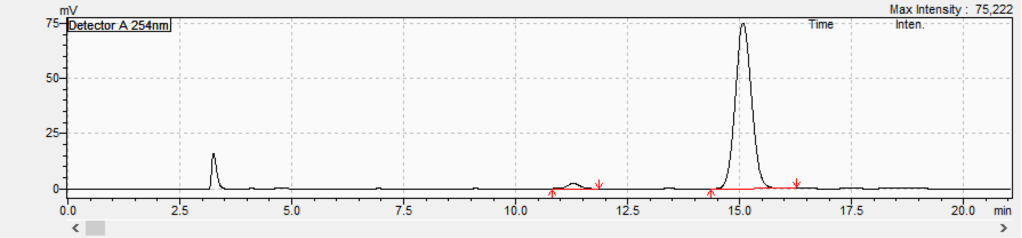

Results View - Peak Table

Peak Table | Compound | Group | Calibration Curve

Peak#	Ret. Time	Area	Height	Mark	Conc.	Unit	ID#	Name	Area%
1	11.290	45781	2411		2.360				2.360
2	15.079	1894351	75164		97.640				97.640
Total		1940131	77574		100.000				100.000

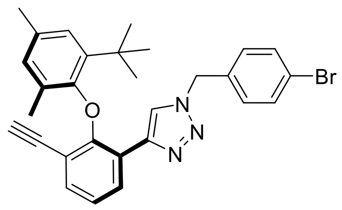

3d

1-(4-Bromobenzyl)-4-(2-(2-(*tert*-butyl)-4,6-dimethylphenoxy)-3-ethynylphenyl)-1*H*-1,2,3-triazole (3d)

75% yield; $[\alpha]_D^{25}$ = -66.9 (c 0.5, $CHCl_3$), a white foam, R_f = 0.30 (hexane/ethyl acetate 8:1). ^{1}H NMR (400 MHz, $CDCl_3$) δ 8.41 (d, J = 9.6 Hz, 1H), 7.96 (s, 1H), 7.37 (d, J = 8.5 Hz, 2H), 7.26 (dd, J = 7.7, 1.8 Hz, 1H), 7.07 – 6.97 (m, 3H), 6.91 (s, 1H), 6.69 (s, 1H), 5.53 (d, J = 15.1 Hz, 1H), 5.31 (d, J = 15.1 Hz, 1H), 2.88 (s, 1H), 2.24 (s, 3H), 1.73 (s, 3H), 1.19 (s, 9H). ^{13}C NMR (101 MHz, $CDCl_3$) δ 154.1, 150.9, 143.5, 141.9, 136.0, 134.0, 133.8, 132.2, 130.4, 129.5, 129.2, 129.16, 125.4, 123.2, 122.8, 122.0, 121.3, 110.5, 82.9, 78.4, 53.5, 34.9, 31.0, 21.1, 17.3. HRMS (ESI) m/z calcd for $C_{29}H_{29}BrN_3O$ $[M+H]^+$ = 514.1489, found = 514.1494; the ee value was 95%, t_R (minor) = 12.0 min, t_R (major) = 15.8 min (Chiralpak IC, λ = 254 nm, 10% *i*-PrOH/Hexane, flow rate = 1.0 mL/min).

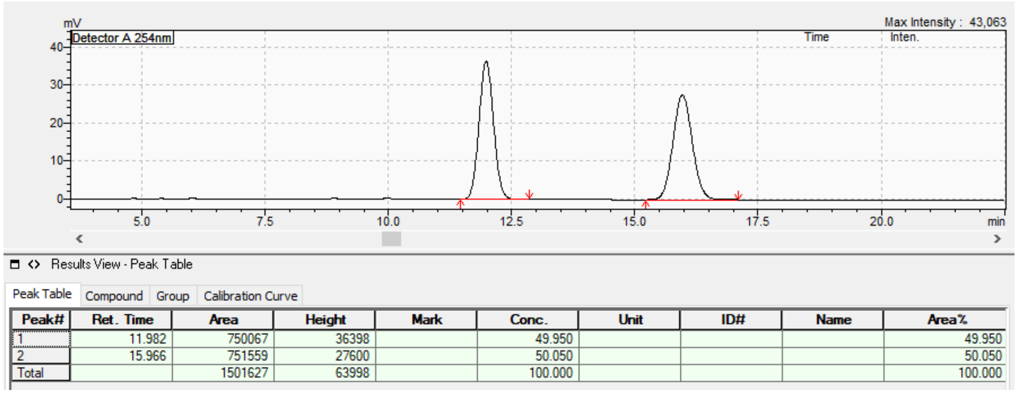

Peak#	Ret. Time	Area	Height	Mark	Conc.	Unit	ID#	Name	Area%
1	11.982	750067	36398		49.950				49.950
2	15.966	751559	27600		50.050				50.050
Total		1501627	63998		100.000				100.000

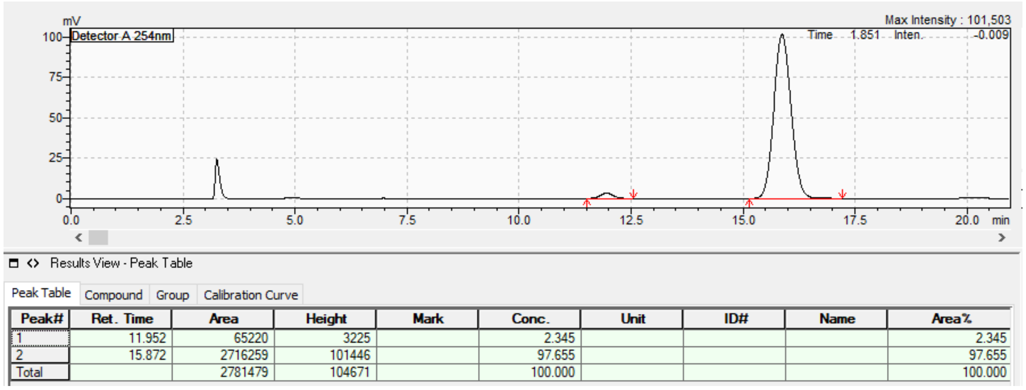

Peak#	Ret. Time	Area	Height	Mark	Conc.	Unit	ID#	Name	Area%
1	11.952	65220	3225		2.345				2.345
2	15.872	2716259	101446		97.655				97.655
Total		2781479	104671		100.000				100.000

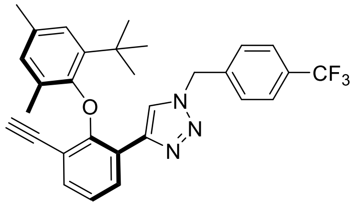

3e

4-(2-(2-(*tert*-Butyl)-4,6-dimethylphenoxy)-3-ethynylphenyl)-1-(4-(trifluoromethyl)benzyl)-1*H*-1,2,3-triazole (3e)

79% yield; $[\alpha]_D^{25}$ = -70.8 (c 0.5, $CHCl_3$), a white foam, R_f = 0.35 (hexane/ethyl acetate 8:1). ^1H NMR (400 MHz, $CDCl_3$) δ 8.41 (dd, *J* = 7.8, 1.8 Hz, 1H), 7.97 (s, 1H), 7.51 (d, *J* = 8.1 Hz, 2H), 7.26 (d, *J* = 7.7 Hz, 3H), 7.00 (t, *J* = 7.7 Hz, 1H), 6.90 (s, 1H), 6.70 (s, 1H), 5.66 (d, *J* = 15.3 Hz, 1H), 5.41 (d, *J* = 15.3 Hz, 1H), 2.58 (s, 1H), 2.24 (s, 3H), 1.73 (s, 3H), 1.17 (s, 9H). ^{13}C NMR (101 MHz, $CDCl_3$) δ 154.1, 150.9, 141.9, 138.8, 136.0, 134.1, 130.4, 129.2, 129.1, 128.0, 126.1, 126.0, 125.4, 123.3, 122.0, 121.3, 110.5, 82.9, 78.4, 53.5, 34.9, 30.9, 21.1, 17.2. HRMS (ESI) m/z calcd for $C_{30}H_{29}F_3N_3O$ $[M+H]^+$ = 504.2257, found = 504.2259; the ee value was 87%, t_R (minor) = 8.7 min, t_R (major) = 11.3 min (Chiralpak IC, λ = 254 nm, 10% *i*-PrOH/Hexane, flow rate = 1.0 mL/min).

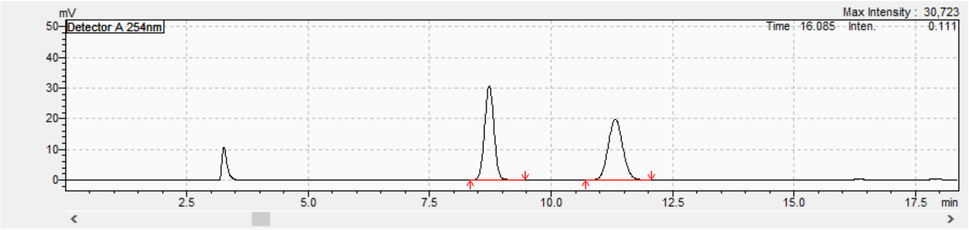

Results View - Peak Table

Peak Table | Compound | Group | Calibration Curve

Peak#	Ret. Time	Area	Height	Mark	Conc.	Unit	ID#	Name	Area%
1	8.729	412411	30689		50.008				50.008
2	11.322	412276	19821		49.992				49.992
Total		824687	50510		100.000				100.000

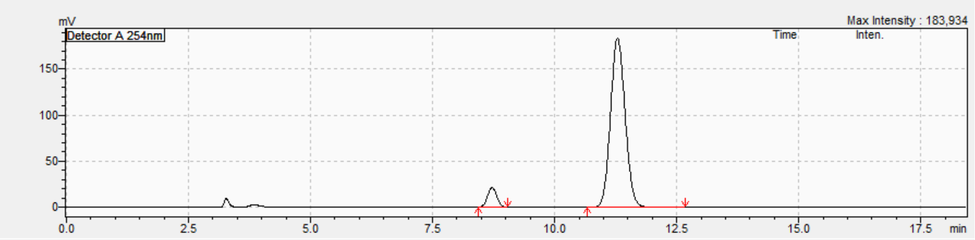

Results View - Peak Table

Peak Table | Compound | Group | Calibration Curve

Peak#	Ret. Time	Area	Height	Mark	Conc.	Unit	ID#	Name	Area%
1	8.721	276723	21260	M	6.728				6.728
2	11.289	3836142	183938		93.272				93.272
Total		4112864	205198		100.000				100.000

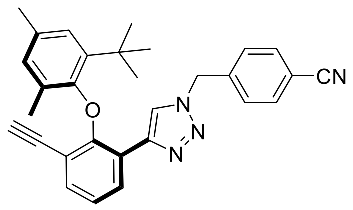

3f

4-((4-(2-(2-(*tert*-Butyl)-4,6-dimethylphenoxy)-3-ethynylphenyl)-1*H*-1,2,3-triazol-1-yl)methyl)benzonitrile (3f)

84% yield; $[\alpha]_D^{25}$ = -32.0 (c 0.5, $CHCl_3$), a white foam, R_f = 0.30 (hexane/ethyl acetate 4:1). ^{1}H NMR (400 MHz, $CDCl_3$) δ 8.41 (dd, *J* = 7.8, 1.8 Hz, 1H), 7.99 (s, 1H), 7.55 (d, *J* = 8.4 Hz, 2H), 7.32 – 7.21 (m, 3H), 7.01 (t, *J* = 7.7 Hz, 1H), 6.91 (d, *J* = 2.2 Hz, 1H), 6.70 (dd, *J* = 1.4, 0.7 Hz, 1H), 5.65 (d, *J* = 15.6 Hz, 1H), 5.43 (d, *J* = 15.6 Hz, 1H), 2.59 (s, 1H), 2.24 (s, 3H), 1.74 (s, 3H), 1.19 (s, 9H). ^{13}C NMR (101 MHz, $CDCl_3$) δ 154.1, 150.8, 143.8, 141.9, 140.0, 136.1, 134.1, 132.8, 130.4, 129.3, 129.1, 128.2, 125.4, 123.3, 122.0, 121.1, 118.2, 112.7, 110.5, 83.0, 78.3, 53.4, 35.0, 31.0, 21.1, 17.2. HRMS (ESI) m/z calcd for $C_{30}H_{29}N_4O$ $[M+H]^+$ = 461.2336, found = 461.2338; the ee value was 90%, t_R (minor) = 10.6 min, t_R (major) = 12.4 min (Chiralpak IC, λ = 254 nm, 30% *i*-PrOH/Hexane, flow rate = 1.0 mL/min).

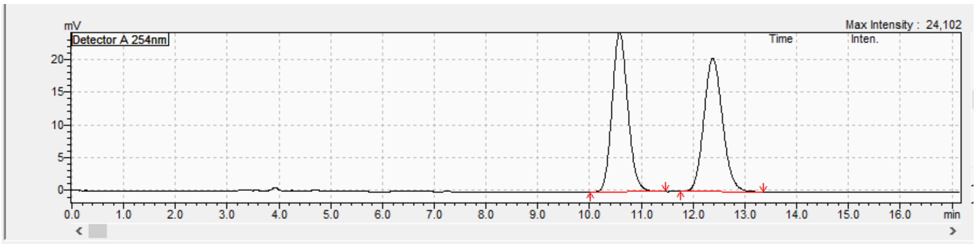

Results View - Peak Table

Peak Table | Compound | Group | Calibration Curve

Peak#	Ret. Time	Area	Height	Mark	Conc.	Unit	ID#	Name	Area%
1	10.579	513509	24310		50.116				50.116
2	12.380	511128	20382		49.884				49.884
Total		1024638	44692		100.000				100.000

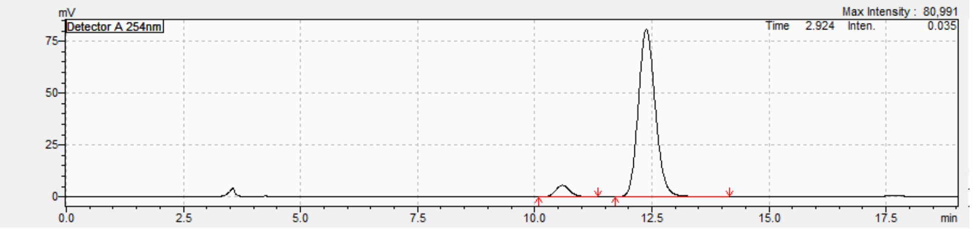

Results View - Peak Table

Peak Table | Compound | Group | Calibration Curve

Peak#	Ret. Time	Area	Height	Mark	Conc.	Unit	ID#	Name	Area%
1	10.589	109990	5449	M	5.111				5.111
2	12.384	2042019	80894		94.889				94.889
Total		2152010	86343		100.000				100.000

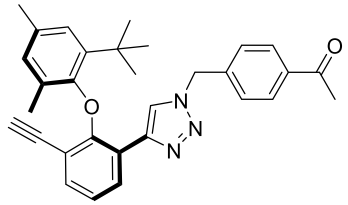

3g

1-(4-((4-(2-(2-(*tert*-Butyl)-4,6-dimethylphenoxy)-3-ethynylphenyl)-1*H*-1,2,3-triazol-1-yl)methyl)phenyl)ethan-1-one (3g)

81% yield; $[\alpha]_D^{25}$ = -89.0 (c 0.5, $CHCl_3$), a white foam, R_f = 0.32 (hexane/ethyl acetate 5:1). ^1H NMR (400 MHz, $CDCl_3$) δ 8.42 (dd, *J* = 7.8, 1.8 Hz, 1H), 7.99 (s, 1H), 7.83 (d, *J* = 8.4 Hz, 2H), 7.29 – 7.21 (m, 3H), 7.00 (t, *J* = 7.7 Hz, 1H), 6.90 (s, 1H), 6.69 (s, 1H), 5.64 (d, *J* = 15.4 Hz, 1H), 5.43 (d, *J* = 15.4 Hz, 1H), 2.58 (s, 1H), 2.50 (s, 3H), 2.24 (s, 3H), 1.73 (s, 3H), 1.18 (s, 9H). ^{13}C NMR (101 MHz, $CDCl_3$) δ 197.4, 154.1, 143.7, 141.9, 139.8, 137.2, 136.0, 134.1, 130.4, 129.2, 129.1, 129.0, 127.8, 125.4, 123.4, 122.0, 121.3, 110.5, 82.9, 78.4, 53.6, 34.9, 31.0, 26.7, 21.1, 17.2. HRMS (ESI) m/z calcd for $C_{31}H_{32}N_3O_2$ $[M+H]^+$ = 478.2489, found = 478.2500; the ee value was 96%, t_R (minor) = 15.1 min, t_R (major) = 16.8 min (Chiralpak IC, λ = 254 nm, 30% *i*-PrOH/Hexane, flow rate = 1.0 mL/min).

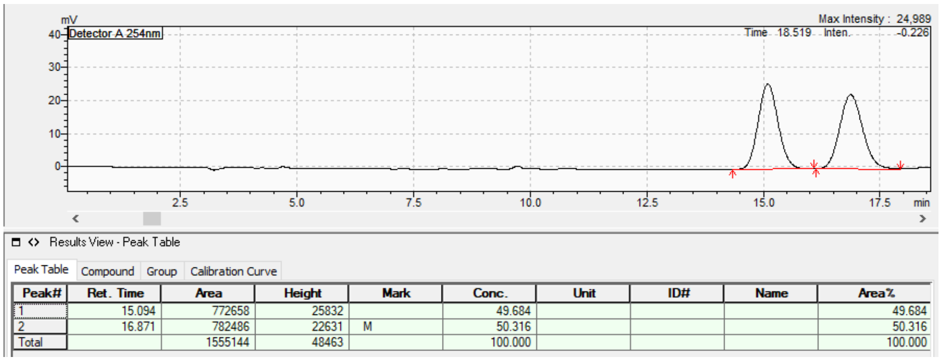

Peak#	Ret. Time	Area	Height	Mark	Conc.	Unit	ID#	Name	Area%
1	15.094	772658	25832		49.684				49.684
2	16.871	782486	22631	M	50.316				50.316
Total		1555144	48463		100.000				100.000

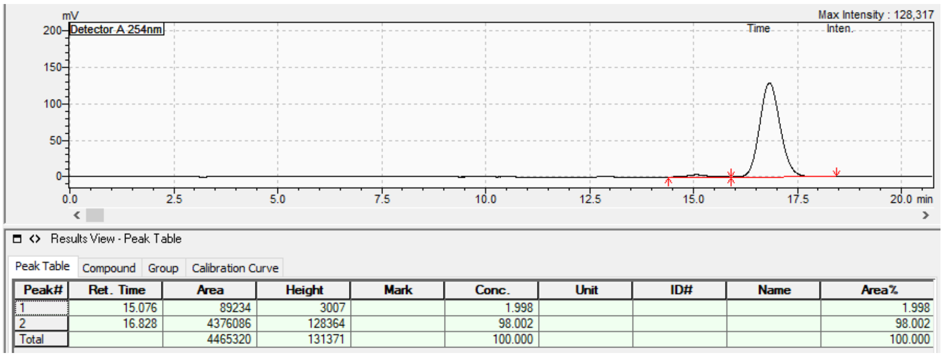

Peak#	Ret. Time	Area	Height	Mark	Conc.	Unit	ID#	Name	Area%
1	15.076	89234	3007		1.998				1.998
2	16.828	4376086	128364		98.002				98.002
Total		4465320	131371		100.000				100.000

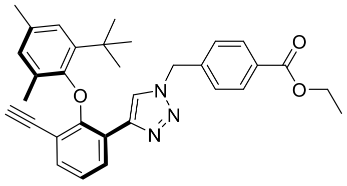

3h

Ethyl 4-((4-(2-(2-(tert-butyl)-4,6-dimethylphenoxy)-3-ethynylphenyl)-1*H*-1,2,3-triazol-1-yl)methyl)benzoate (3h)

70% yield; $[\alpha]_D^{25}$ = -59.2 (c 0.5, $CHCl_3$), a white foam, R_f = 0.34 (hexane/ethyl acetate 5:1). ^{1}H NMR (400 MHz, $CDCl_3$) δ 8.42 (dd, *J* = 7.8, 1.8 Hz, 1H), 7.99 (s, 1H), 7.92 (d, *J* = 8.4 Hz, 2H), 7.26 (dd, *J* = 7.7, 1.8 Hz, 1H), 7.21 (s, 1H), 7.00 (t, *J* = 7.7 Hz, 1H), 6.90 (s, 1H), 6.69 (s, 1H), 5.63 (d, *J* = 15.4 Hz, 1H), 5.42 (d, *J* = 15.4 Hz, 1H), 4.30 (q, *J* = 7.1 Hz, 2H), 2.58 (s, 1H), 2.24 (s, 3H), 1.73 (s, 3H), 1.31 (t, *J* = 7.1 Hz, 3H), 1.19 (s, 9H). ^{13}C NMR (101 MHz, $CDCl_3$) δ 166.0, 154.1, 150.9, 141.9, 139.6, 136.0, 134.0, 130.8, 130.4, 130.3, 129.2, 129.1, 127.5, 125.4, 123.4, 122.0, 121.4, 110.5, 82.9, 78.4, 61.2, 53.7, 34.9, 31.0, 21.1, 17.2, 14.3. HRMS (ESI) m/z calcd for $C_{32}H_{34}N_3O_3$ $[M+H]^+$ = 508.2595, found = 508.2608; the ee value was 94%, t_R (minor) = 26.7 min, t_R (major) = 34.7 min (Chiralpak IC, λ = 254 nm, 10% *i*-PrOH/Hexane, flow rate = 1.0 mL/min).

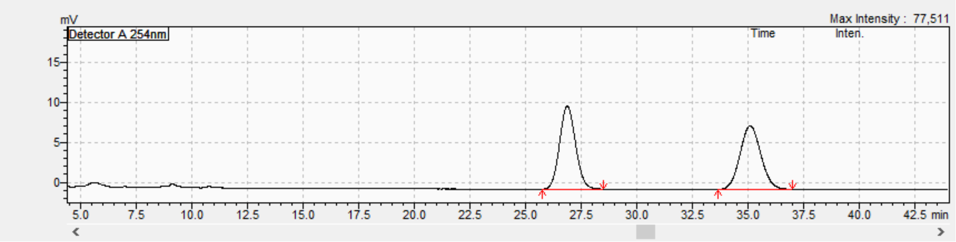

Results View - Peak Table

Peak Table | Compound | Group | Calibration Curve

Peak#	Ret. Time	Area	Height	Mark	Conc.	Unit	ID#	Name
1	26.856	518320	10376		49.984			
2	35.073	518648	7960		50.016			
Total		1036968	18335		100.000			

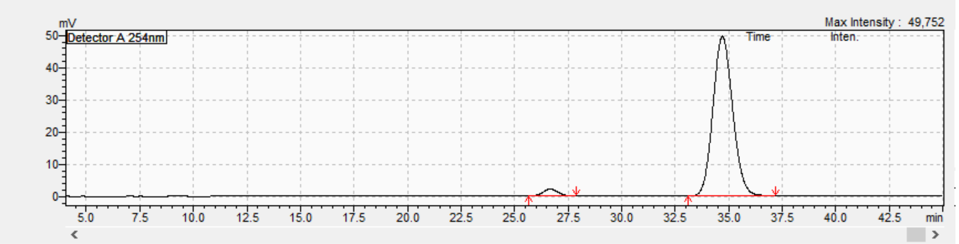

Results View - Peak Table

Peak Table | Compound | Group | Calibration Curve

Peak#	Ret. Time	Area	Height	Mark	Conc.	Unit	ID#	Name
1	26.675	103293	2114		3.159			
2	34.707	3166879	49538		96.841			
Total		3270171	51652		100.000			

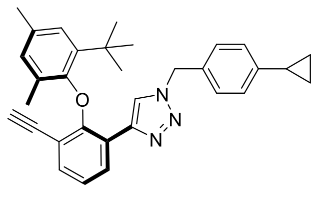

3i

4-(2-(2-(*tert*-Butyl)-4,6-dimethylphenoxy)-3-ethynylphenyl)-1-(4-cyclopropylbenzyl)-1*H*-1,2,3-triazole (3i)

76% yield; $[\alpha]_D^{25}$ = -71.2 (c 0.5, $CHCl_3$), a white foam, R_f = 0.32 (hexane/ethyl acetate 8:1). ^{1}H NMR (400 MHz, $CDCl_3$) δ 8.40 (dd, *J* = 7.8, 1.8 Hz, 1H), 7.93 (s, 1H), 7.24 (dd, *J* = 7.7, 1.8 Hz, 1H), 7.05 (d, *J* = 8.2 Hz, 2H), 6.98 (t, *J* = 7.7 Hz, 1H), 6.95 – 6.86 (m, 3H), 6.73 – 6.58 (m, 1H), 5.52 (d, *J* = 14.8 Hz, 1H), 5.29 (d, *J* = 14.8 Hz, 1H), 2.57 (s, 1H), 2.24 (s, 3H), 1.78 (tt, *J* = 8.4, 5.1 Hz, 1H), 1.72 (s, 3H), 1.18 (s, 9H), 0.91 – 0.83 (m, 2H), 0.62 – 0.52 (m, 2H). ^{13}C NMR (101 MHz, $CDCl_3$) δ 154.1, 151.0, 144.7, 141.93,135.8, 133.9, 131.7, 130.4, 129.2, 129.1, 127.9, 126.29, 125.3, 123.2, 121.9, 121.6, 110.4, 82.8, 78.5, 54.0, 34.9, 31.0, 21.1, 17.2, 15.1, 9.3. HRMS (ESI) m/z calcd for $C_{32}H_{34}N_3O$ $[M+H]^+$ = 476.2696, found = 476.2705; the ee value was 97%, t_R (minor) = 15.3 min, t_R (major) = 20.3 min (Chiralpak IC, λ = 254 nm, 10% *i*-PrOH/Hexane, flow rate = 1.0 mL/min).

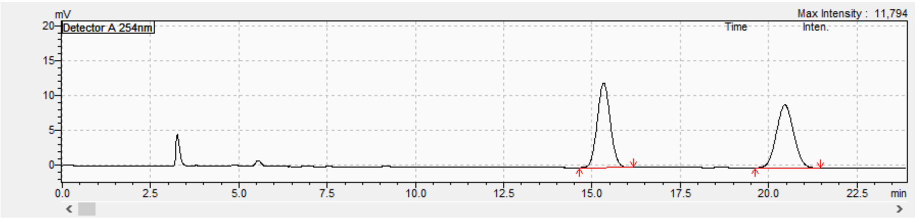

Results View - Peak Table

Peak Table | Compound | Group | Calibration Curve

Peak#	Ret. Time	Area	Height	Mark	Conc.	Unit	ID#	Name	Area%
1	15.330	309518	12100		50.102				50.102
2	20.461	308261	9015		49.898				49.898
Total		617779	21114		100.000				100.000

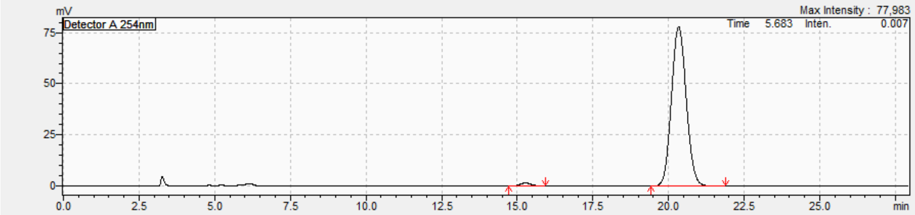

Results View - Peak Table

Peak Table | Compound | Group | Calibration Curve

Peak#	Ret. Time	Area	Height	Mark	Conc.	Unit	ID#	Name	Area%
1	15.275	36766	1456		1.366				1.366
2	20.333	2655454	78113		98.634				98.634
Total		2692220	79569		100.000				100.000

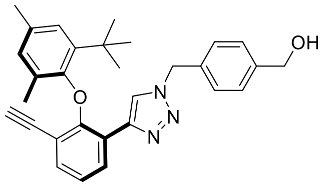

3j

(4-((4-(2-(2-(*tert*-Butyl)-4,6-dimethylphenoxy)-3-ethynylphenyl)-1*H*-1,2,3-triazol-1-yl)methyl)phenyl)methanol (3j)

77% yield; $[\alpha]_D^{25}$ = -53.0 (c 0.5, $CHCl_3$), a white foam, R_f = 0.28 (hexane/ethyl acetate 2:1). ^1H NMR (400 MHz, $CDCl_3$) δ 8.40 (d, *J* = 7.8 Hz, 1H), 7.97 (s, 1H), 7.24 (d, *J* = 7.9 Hz, 2H), 7.15 (d, *J* = 7.7 Hz, 2H), 6.99 (t, *J* = 7.7 Hz, 1H), 6.90 (s, 1H), 6.69 (s, 1H), 5.56 (d, *J* = 14.9 Hz, 1H), 5.36 (d, *J* = 14.9 Hz, 1H), 4.60 (s, 2H), 2.58 (s, 1H), 2.24 (s, 3H), 1.72 (s, 3H), 1.19 (s, 9H). ^{13}C NMR (101 MHz, $CDCl_3$) δ 154.1, 151.0, 141.9, 141.4, 135.9, 134.1, 134.0, 130.5, 129.2, 129.1, 128.0, 127.5, 125.3, 122.0, 121.5, 110.5, 82.9, 64.8, 53.9, 34.9, 31.0, 21.1, 17.2. HRMS (ESI) m/z calcd for $C_{30}H_{32}N_3O_2$ $[M+H]^+$ = 466.2489, found = 466.2495; the ee value was 92%, t_R (minor) = 8.2 min, t_R (major) = 8.7 min (Chiralpak IC, λ = 254 nm, 30% *i*-PrOH/Hexane, flow rate = 1.0 mL/min).

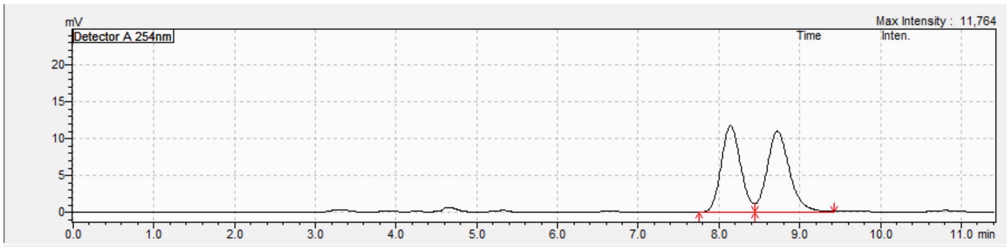

Results View - Peak Table

Peak Table | Compound | Group | Calibration Curve

Peak#	Ret. Time	Area	Height	Mark	Conc.	Unit	ID#	Name	Area%
1	8.143	194147	11699		48.018				48.018
2	8.727	210173	10940	V	51.982				51.982
Total		404319	22640		100.000				100.000

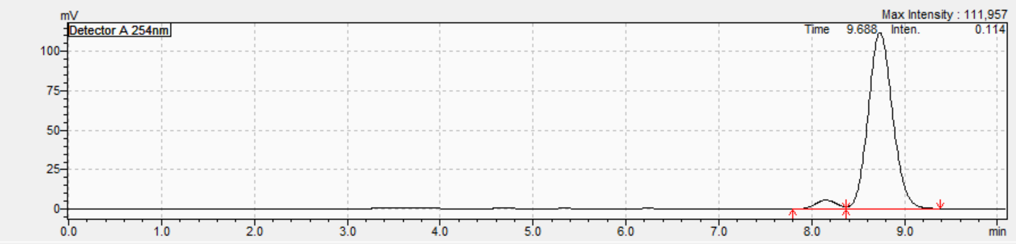

Results View - Peak Table

Peak Table | Compound | Group | Calibration Curve

Peak#	Ret. Time	Area	Height	Mark	Conc.	Unit	ID#	Name	Area%
1	8.152	90784	5632		4.247				4.247
2	8.733	2047019	111876	V M	95.753				95.753
Total		2137803	117509		100.000				100.000

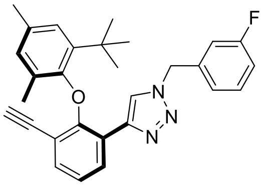

3k

4-(2-(2-(*tert*-Butyl)-4,6-dimethylphenoxy)-3-ethynylphenyl)-1-(3-fluorobenzyl)-1*H*-1,2,3-triazole (3k)

79% yield; $[\alpha]_D^{25}$ = -57.0 (c 0.5, $CHCl_3$), a white foam, R_f = 0.29 (hexane/ethyl acetate 8:1). ^{1}H NMR (400 MHz, $CDCl_3$) δ 8.41 (d, *J* = 7.9 Hz, 1H), 7.98 (s, 1H), 7.30 – 7.20 (m, 2H), 7.06 – 6.81 (m, 5H), 6.69 (s, 1H), 5.59 (d, *J* = 15.2 Hz, 1H), 5.34 (d, *J* = 15.2 Hz, 1H), 2.58 (s, 1H), 2.24 (s, 3H), 1.74 (s, 3H), 1.19 (s, 9H). ^{13}C NMR (101 MHz, $CDCl_3$) δ 150.9, 143.6, 141.9, 135.9, 134.0, 130.7, 130.7, 130.4, 129.2, 129.1, 125.4, 123.3, 123.3, 122.0, 121.4, 115.7, 115.5, 114.9, 114.7, 110.5, 82.9, 78.4, 53.5, 53.5, 34.9, 31.0, 21.1, 17.2. HRMS (ESI) m/z calcd for $C_{29}H_{29}FN_3O$ $[M+H]^+$ = 454.2289, found = 454.2291; the ee value was 85%, t_R (minor) = 10.9 min, t_R (major) = 15.0 min (Chiralpak IC, λ = 254 nm, 10% *i*-PrOH/Hexane, flow rate = 1.0 mL/min).

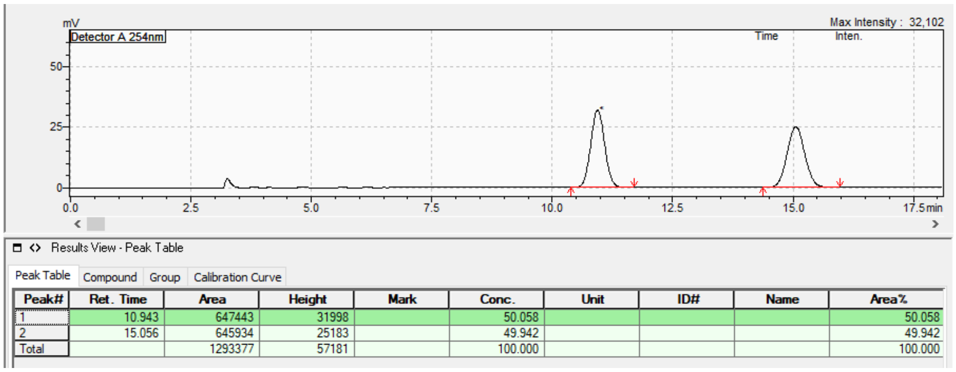

Peak#	Ret. Time	Area	Height	Mark	Conc.	Unit	ID#	Name	Area%
1	10.943	647443	31998		50.058				50.058
2	15.056	645934	25183		49.942				49.942
Total		1293377	57181		100.000				100.000

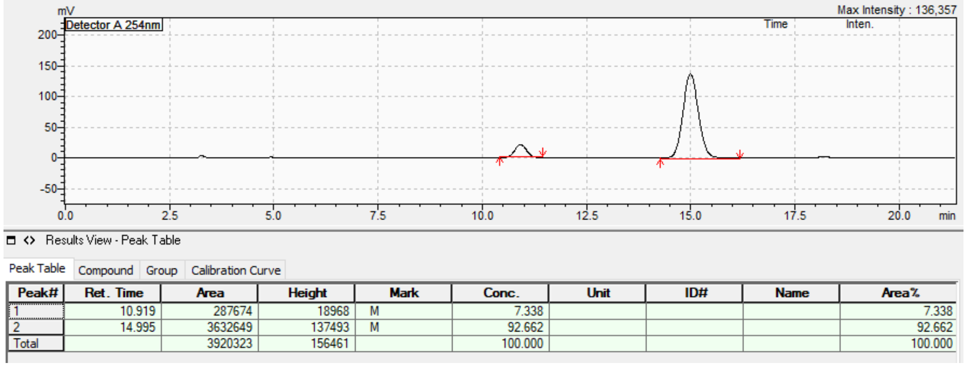

Peak#	Ret. Time	Area	Height	Mark	Conc.	Unit	ID#	Name	Area%
1	10.919	287674	18968	M	7.338				7.338
2	14.995	3632649	137493	M	92.662				92.662
Total		3920323	156461		100.000				100.000

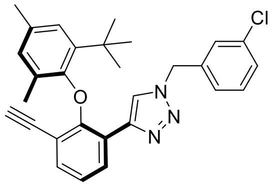

3l

4-(2-(2-(*tert*-Butyl)-4,6-dimethylphenoxy)-3-ethynylphenyl)-1-(3-chlorobenzyl)-1*H*-1,2,3-triazole (3l)

79% yield; $[\alpha]_D^{25}$ = -76.0 (c 0.5, $CHCl_3$), a white foam, R_f = 0.35 (hexane/ethyl acetate 7:1). ^1H NMR (400 MHz, $CDCl_3$) δ 8.42 (dd, *J* = 7.8, 1.8 Hz, 1H), 7.98 (s, 1H), 7.29 – 7.14 (m, 4H), 7.07 – 6.97 (m, 2H), 6.91 (s, 1H), 6.69 (s, 1H), 5.56 (d, *J* = 15.2 Hz, 1H), 5.32 (d, *J* = 15.2 Hz, 1H), 2.58 (s, 1H), 2.24 (s, 3H), 1.74 (s, 3H), 1.20 (s, 9H). ^{13}C NMR (101 MHz, $CDCl_3$) δ 154.1, 150.9, 143.6, 141.9, 136.8, 136.0, 1345.0, 134.0, 130.4, 130.3, 129.2, 129.1, 128.8, 127.9, 125.8, 125.4, 123.3, 122.0, 121.4, 110.5, 82.9, 78.4, 53.4, 35.0, 31.0, 21.1, 17.2. HRMS (ESI) m/z calcd for $C_{29}H_{29}ClN_3O$ $[M+H]^+$ = 470.1994, found = 470.1998; the ee value was 91%, t_R (minor) = 11.5 min, t_R (major) = 17.5 min (Chiralpak IC, λ = 254 nm, 10% *i*-PrOH/Hexane, flow rate = 1.0 mL/min).

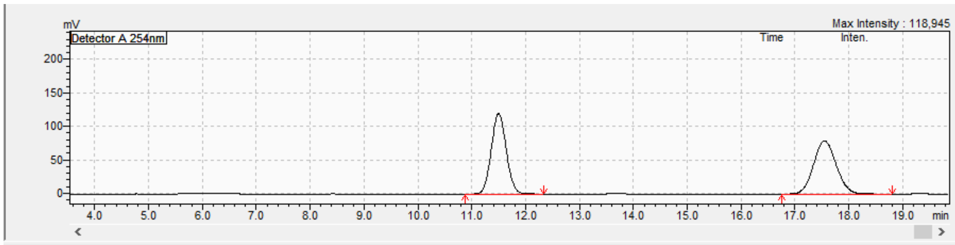

Results View - Peak Table

Peak Table | Compound | Group | Calibration Curve

Peak#	Ret. Time	Area	Height	Mark	Conc.	Unit	ID#	Name	Area%
1	11.498	2320651	119942		50.016				50.016
2	17.547	2319182	79385		49.984				49.984
Total		4639832	199326		100.000				100.000

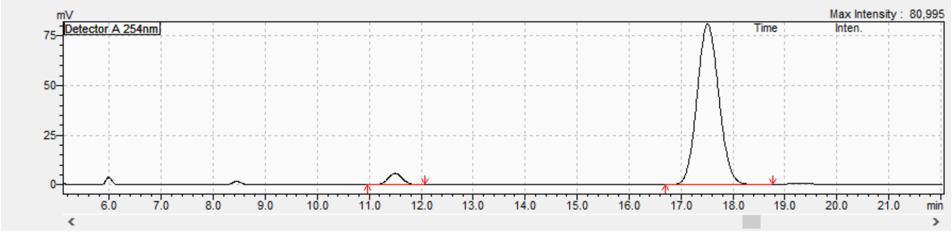

Results View - Peak Table

Peak Table | Compound | Group | Calibration Curve

Peak#	Ret. Time	Area	Height	Mark	Conc.	Unit	ID#	Name	Area%
1	11.498	111178	5740		4.506				4.506
2	17.512	2356243	80898		95.494				95.494
Total		2467421	86638		100.000				100.000

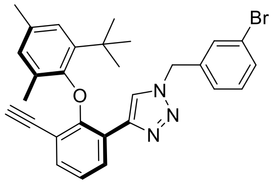

3m

4-(2-(2-(*tert*-Butyl)-4,6-dimethylphenoxy)-3-ethynylphenyl)-1-(3-bormobenzyl)-1*H*-1,2,3-triazole (3m)

73% yield; $[\alpha]_D^{25}$ = -48.3 (c 0.5, $CHCl_3$), a white foam, R_f = 0.32 (hexane/ethyl acetate 7:1). ^1H NMR (400 MHz, $CDCl_3$) δ 8.42 (dd, *J* = 7.8, 1.8 Hz, 1H), 7.98 (s, 1H), 7.36 (d, *J* = 9.2 Hz, 1H), 7.33 – 7.30 (m, 1H), 7.26 (dd, *J* = 7.6, 1.8 Hz, 1H), 7.16 – 7.06 (m, 2H), 7.00 (t, *J* = 7.7 Hz, 1H), 6.91 (s, 1H), 6.70 (s, 1H), 5.56 (d, *J* = 15.2 Hz, 1H), 5.31 (d, *J* = 15.2 Hz, 1H), 2.58 (s, 1H), 2.24 (s, 3H), 1.74 (s, 3H), 1.20 (s, 9H). ^{13}C NMR (101 MHz, $CDCl_3$) δ 154.1, 150.9, 143.6, 141.9, 137.0, 135.9, 134.0, 131.8, 130.7, 130.6, 130.4, 129.2, 129.1, 126.3, 125.4, 123.3, 123.06, 122.0, 121.4, 110.5, 82.9, 78.4, 53.4, 35.0, 31.0, 21.1, 17.3. HRMS (ESI) m/z calcd for $C_{29}H_{29}BrN_3O$ $[M+H]^+$ = 514.1489, found = 514.1495; the ee value was 94%, t_R (minor) = 11.8 min, t_R (major) = 18.5 min (Chiralpak IC, λ = 254 nm, 10% *i*-PrOH/Hexane, flow rate = 1.0 mL/min).

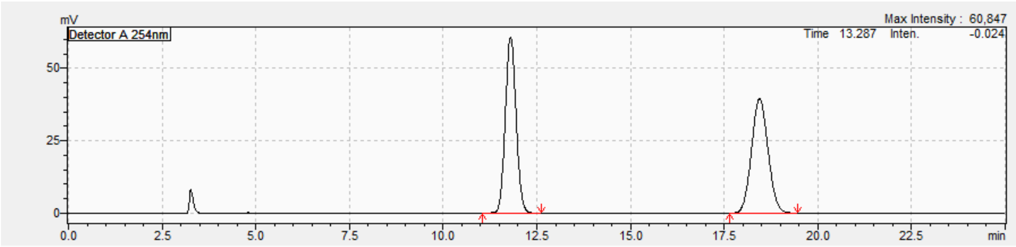

Results View - Peak Table

Peak Table | Compound | Group | Calibration Curve

Peak#	Ret. Time	Area	Height	Mark	Conc.	Unit	ID#	Name	Area%
1	11.800	1220969	60844	V	50.081				50.081
2	18.445	1217007	39404		49.919				49.919
Total		2437976	100248		100.000				100.000

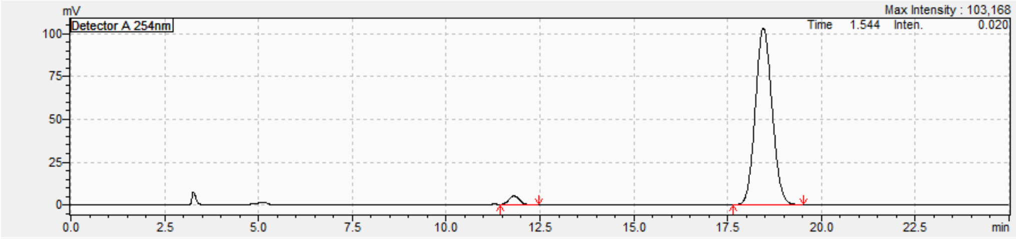

Results View - Peak Table

Peak Table | Compound | Group | Calibration Curve

Peak#	Ret. Time	Area	Height	Mark	Conc.	Unit	ID#	Name	Area%
1	11.807	105064	5179		3.178				3.178
2	18.454	3200761	103034		96.822				96.822
Total		3305825	108213		100.000				100.000

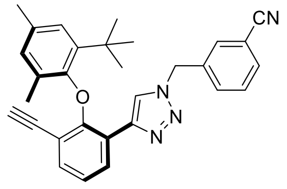

3n

3-((4-(2-(2-(*tert*-Butyl)-4,6-dimethylphenoxy)-3-ethynylphenyl)-1*H*-1,2,3-triazol-1-yl)methyl)benzonitrile (3n)

75% yield; $[\alpha]_D^{25}$ = -78.2 (c 0.5, $CHCl_3$), a white foam, R_f = 0.22 (hexane/ethyl acetate 4:1). ^{1}H NMR (400 MHz, $CDCl_3$) δ 8.41 (d, *J* = 7.8 Hz, 1H), 8.00 (d, *J* = 1.5 Hz, 1H), 7.54 (t, *J* = 4.6 Hz, 1H), 7.45 (s, 1H), 7.38 (d, *J* = 4.7 Hz, 2H), 7.27 (d, *J* = 7.7 Hz, 1H), 7.01 (t, *J* = 7.8 Hz, 1H), 6.91 (s, 1H), 6.70 (s, 1H), 5.63 (d, *J* = 15.4 Hz, 1H), 5.40 (d, *J* = 15.4 Hz, 1H), 2.59 (s, 1H), 2.24 (s, 3H), 1.74 (s, 3H), 1.20 (s, 9H). ^{13}C NMR (101 MHz, $CDCl_3$) δ 154.1, 150.8, 143.9, 141.9, 136.5, 136.1, 134.1, 132.3, 132.0, 131.0, 130.4, 130.0, 129.3, 129.1, 125.4, 123.3, 122.0, 121.2, 118.0, 113.4, 110.5, 83.0, 78.3, 53.1, 35.0, 31.0, 21.1, 17.3. HRMS (ESI) m/z calcd for $C_{30}H_{29}N_4O$ $[M+H]^+$ = 461.2336, found = 461.2338; the ee value was 94%, t_R (minor) = 16.5 min, t_R (major) = 28.6 min (Chiralpak IC, λ = 254 nm, 30% *i*-PrOH/Hexane, flow rate = 1.0 mL/min).

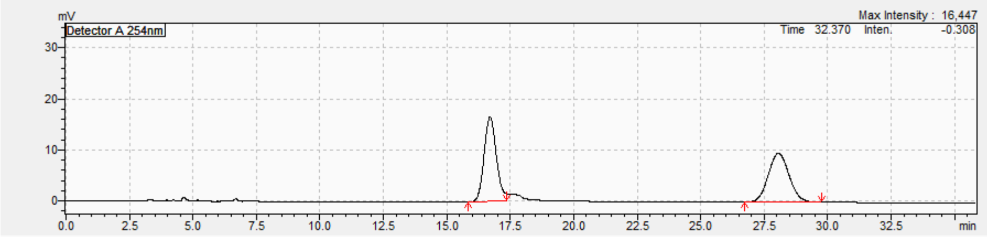

Results View - Peak Table

Peak Table | Compound | Group | Calibration Curve

Peak#	Ret. Time	Area	Height	Mark	Conc.	Unit	ID#	Name	Area%
1	16.701	559106	16560	M	50.590				50.590
2	28.062	546062	9569		49.410				49.410
Total		1105168	26129		100.000				100.000

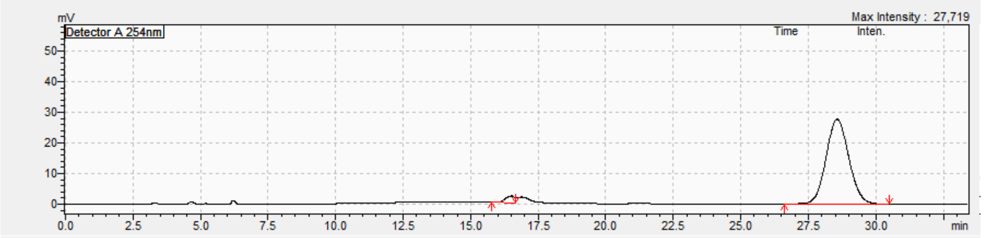

Results View - Peak Table

Peak Table | Compound | Group | Calibration Curve

Peak#	Ret. Time	Area	Height	Mark	Conc.	Unit	ID#	Name	Area%
1	16.530	52691	2169		3.140				3.140
2	28.565	1625370	27680		96.860				96.860
Total		1678061	29849		100.000				100.000

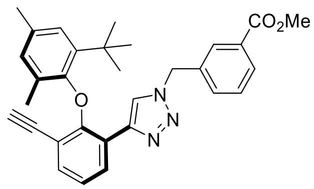

3o

Methyl 3-((4-(2-(2-(*tert*-butyl)-4,6-dimethylphenoxy)-3-ethynylphenyl)-1*H*-1,2,3-triazol-1-yl)methyl)benzoate (3o)

82% yield; $[\alpha]_D^{25}$ = -91.6 (c 0.5, $CHCl_3$), a white foam, R_f = 0.32 (hexane/ethyl acetate 6:1). ^1H NMR (400 MHz, $CDCl_3$) δ 8.42 (d, *J* = 7.3 Hz, 1H), 7.98 (s, 1H), 7.95 – 7.90 (m, 1H), 7.87 (p, *J* = 1.0 Hz, 1H), 7.37 – 7.30 (m, 2H), 7.25 (dd, *J* = 7.6, 1.2 Hz, 1H), 6.99 (t, *J* = 7.6 Hz, 1H), 6.89 (d, *J* = 2.2 Hz, 1H), 6.69 (dt, *J* = 2.3, 0.7 Hz, 1H), 5.63 (d, *J* = 15.0 Hz, 1H), 5.40 (d, *J* = 15.0 Hz, 1H), 3.82 (s, 3H), 2.58 (s, 1H), 2.23 (s, 3H), 1.73 (s, 3H), 1.17 (s, 9H). ^{13}C NMR (101 MHz, $CDCl_3$) δ 166.4, 154.1, 141.9, 135.9, 135.3, 134.0, 132.2, 131.0, 130.4, 129.9, 129.3, 129.2, 129.1, 128.9, 125.3, 121.9, 121.4, 110.5, 82.9, 78.4, 53.7, 52.3, 34.9, 31.0, 21.1, 17.2. HRMS (ESI) m/z calcd for $C_{31}H_{32}N_3O_3$ $[M+H]^+$ = 494.2438, found = 494.2440; the ee value was 90%, t_R (minor) = 11.7 min, t_R (major) = 15.4 min (Chiralpak IC, λ = 254 nm, 30% *i*-PrOH/Hexane, flow rate = 1.0 mL/min).

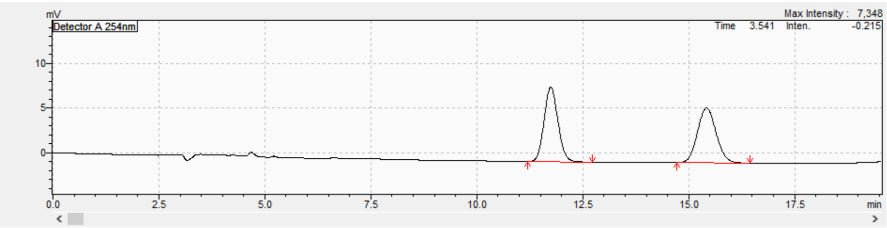

Results View - Peak Table

Peak Table | Compound | Group | Calibration Curve

Peak#	Ret. Time	Area	Height	Mark	Conc.	Unit	ID#	Name	Area%
1	11.746	189954	8346		50.202				50.202
2	15.420	188427	6132		49.798				49.798
Total		378381	14478		100.000				100.000

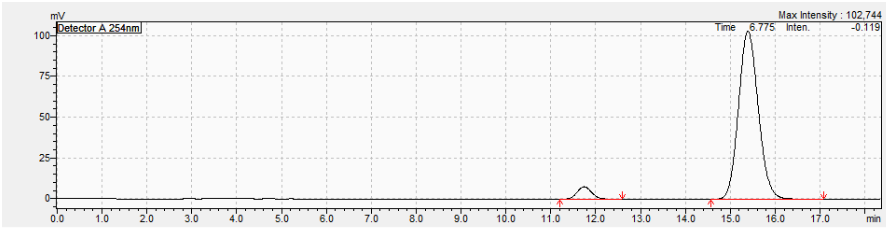

Results View - Peak Table

Peak Table | Compound | Group | Calibration Curve

Peak#	Ret. Time	Area	Height	Mark	Conc.	Unit	ID#	Name	Area%
1	11.742	175040	7680		5.247				5.247
2	15.395	3160860	103059		94.753				94.753
Total		3335899	110739		100.000				100.000

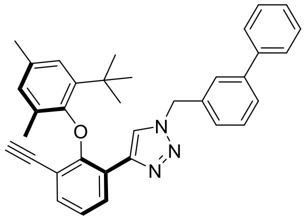

3p

1-([1,1'-Biphenyl]-3-ylmethyl)-4-(2-(2-(*tert*-butyl)-4,6-dimethylphenoxy)-3-ethynylphenyl)-1*H*-1,2,3-triazole (3p)

76% yield; $[\alpha]_D^{25}$ = -62.2 (c 0.5, $CHCl_3$), a white foam, R_f = 0.32 (hexane/ethyl acetate 8:1). ^1H NMR (400 MHz, $CDCl_3$) δ 8.42 (dd, *J* = 7.8, 1.8 Hz, 1H), 8.02 (s, 1H), 7.49 – 7.23 (m, 9H), 7.13 (dt, *J* = 7.8, 1.3 Hz, 1H), 6.88 (d, *J* = 2.2 Hz, 1H), 6.71 – 6.64 (m, 1H), 5.64 (d, *J* = 15.0 Hz, 1H), 5.41 (d, *J* = 15.0 Hz, 1H), 2.57 (s, 1H), 2.23 (s, 3H), 1.72 (s, 3H), 1.14 (s, 9H). ^{13}C NMR (101 MHz, $CDCl_3$) δ 154.1, 151.0, 143.5, 142.2, 141.9, 135.8, 135.4, 133.9, 130.4, 129.5, 129.2, 129.1, 128.8, 127.6, 127.4, 127.2, 126.7, 126.6, 125.3, 123.3, 121.9, 121.6, 110.5, 82.8, 54.2, 34.9, 31.0, 21.1, 17.2. HRMS (ESI) m/z calcd for $C_{35}H_{34}N_3O$ $[M+H]^+$ = 512.2696, found = 512.2704; the ee value was 92%, t_R (minor) = 14.5 min, t_R (major) = 18.6 min (Chiralpak IC, λ = 254 nm, 10% *i*-PrOH/Hexane, flow rate = 1.0 mL/min).

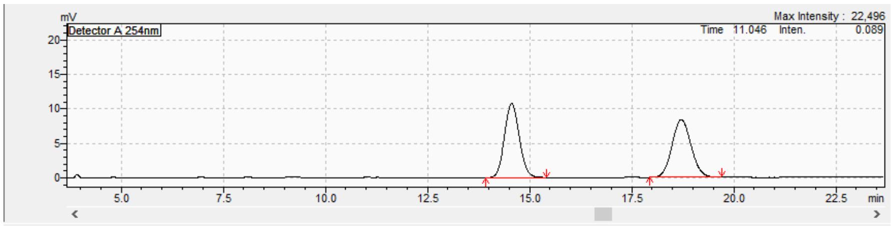

Results View - Peak Table

Peak Table | Compound | Group | Calibration Curve

Peak#	Ret. Time	Area	Height	Mark	Conc.	Unit	ID#	Name
1	14.565	270749	10710		49.552			
2	18.717	270113	8333		49.435			
3	24.305	5537	189		1.013			
Total		546400	19231		100.000			

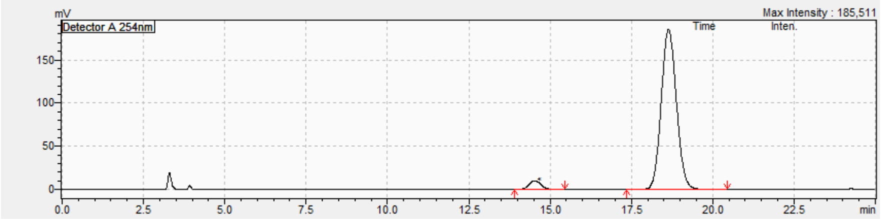

Results View - Peak Table

Peak Table | Compound | Group | Calibration Curve

Peak#	Ret. Time	Area	Height	Mark	Conc.	Unit	ID#	Name
1	14.534	254634	10085		4.071			
2	18.643	5999639	185271		95.929			
Total		6254273	195356		100.000			

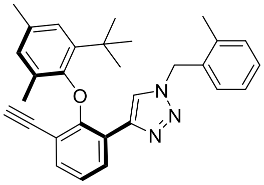

3q

4-(2-(2-(*tert*-Butyl)-4,6-dimethylphenoxy)-3-ethynylphenyl)-1-(2-methylbenzyl)-1*H*-1,2,3-triazole (3q)

82% yield; $[\alpha]_D^{25}$ = -52.9 (c 0.5, $CHCl_3$), a white foam, R_f = 0.34 (hexane/ethyl acetate 8:1). ^1H NMR (400 MHz, $CDCl_3$) δ 8.41 (dd, *J* = 7.8, 1.8 Hz, 1H), 7.88 (s, 1H), 7.24 (dd, *J* = 7.6, 1.8 Hz, 1H), 7.14 (td, *J* = 7.4, 1.4 Hz, 1H), 7.11 – 7.03 (m, 2H), 6.99 (t, *J* = 7.7 Hz, 2H), 6.88 (d, *J* = 2.0 Hz, 1H), 6.67 (d, *J* = 2.1 Hz, 1H), 5.61 (d, *J* = 14.9 Hz, 1H), 5.35 (d, *J* = 15.0 Hz, 1H), 2.57 (s, 1H), 2.23 (s, 3H), 2.21 (s, 3H), 1.70 (s, 3H), 1.17 (s, 9H). ^{13}C NMR (101 MHz, $CDCl_3$) δ 154.2, 151.1, 143.1, 141.9, 136.6, 135.9, 133.9, 132.7, 130.9, 130.4, 129.2, 129.1, 129.0, 128.9, 126.6, 125.3, 123.2, 121.9, 121.6, 110.5, 82.8, 78.5, 52.4, 34.9, 31.0, 21.1, 19.0, 17.2. HRMS (ESI) m/z calcd for $C_{30}H_{32}N_3O$ $[M+H]^+$ = 450.2540, found = 450.2541; the ee value was 85%, t_R (minor) = 11.4 min, t_R (major) = 17.9 min (Chiralpak IC, λ = 254 nm, 10% *i*-PrOH/Hexane, flow rate = 1.0 mL/min).

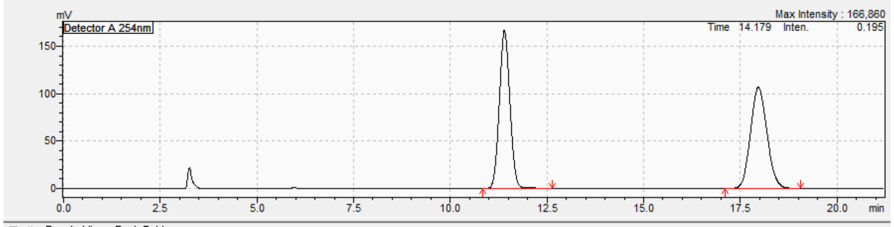

Results View - Peak Table

Peak Table | Compound | Group | Calibration Curve

Peak#	Ret. Time	Area	Height	Mark	Conc.	Unit	ID#	Name	Area%
1	11.402	3168411	166713		50.083				50.083
2	17.968	3157955	106555		49.917				49.917
Total		6326365	273268		100.000				100.000

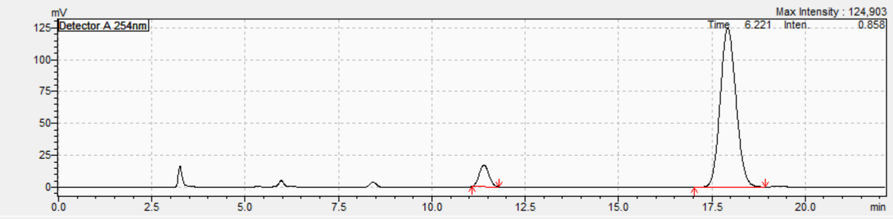

Results View - Peak Table

Peak Table | Compound | Group | Calibration Curve

Peak#	Ret. Time	Area	Height	Mark	Conc.	Unit	ID#	Name	Area%
1	11.394	305828	17002	M	7.668				7.668
2	17.925	3682536	124837		92.332				92.332
Total		3988363	141839		100.000				100.000

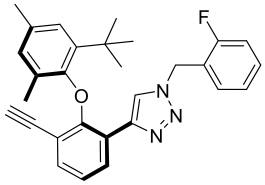

3r

4-(2-(2-(*tert*-Butyl)-4,6-dimethylphenoxy)-3-ethynylphenyl)-1-(2-fluorobenzyl)-1*H*-1,2,3-triazole (3r)

76% yield; $[\alpha]_D^{25}$ = -71.7 (c 0.5, $CHCl_3$), a white foam, R_f = 0.32 (hexane/ethyl acetate 8:1). ^{1}H NMR (400 MHz, $CDCl_3$) δ 8.40 (d, *J* = 7.9 Hz, 1H), 8.05 (s, 1H), 7.32 – 7.13 (m, 3H), 7.07 – 6.96 (m, 3H), 6.90 (s, 1H), 6.69 (s, 1H), 5.59 (d, *J* = 15.1 Hz, 1H), 5.47 (d, *J* = 15.1 Hz, 1H), 2.58 (s, 1H), 2.24 (s, 3H), 1.73 (s, 3H), 1.22 (s, 9H). ^{13}C NMR (101 MHz, $CDCl_3$) δ 161.6, 151.0, 143.4, 142.0, 135.9, 133.9, 130.7, 130.7, 130.5, 130.24, 130.21, 129.2, 129.1, 125.3, 124.8, 124.7, 123.5, 121.9, 121.5, 115.8, 115.6, 110.5, 82.8, 78.5, 47.7, 47.6, 34.9, 31.0, 21.1, 17.2. HRMS (ESI) m/z calcd for $C_{29}H_{29}FN_3O$ $[M+H]^+$ = 454.2289, found = 454.2291; the ee value was 90%, t_R (minor) = 11.6 min, t_R (major) = 16.5 min (Chiralpak IC, λ = 254 nm, 10% *i*-PrOH/Hexane, flow rate = 1.0 mL/min).

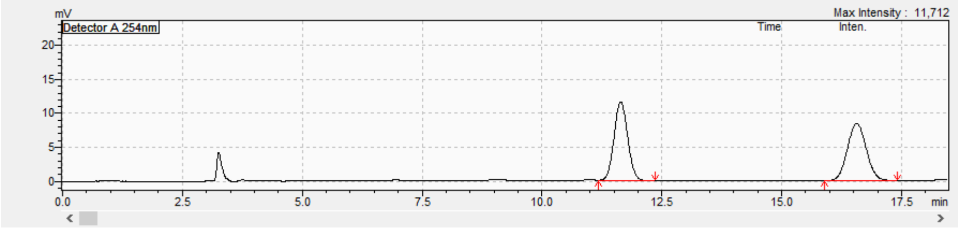

Results View - Peak Table

Peak Table | Compound | Group | Calibration Curve

Peak#	Ret. Time	Area	Height	Mark	Conc.	Unit	ID#	Name	Area%
1	11.643	231262	11625		50.117				50.117
2	16.562	230186	8449		49.883				49.883
Total		461448	20074		100.000				100.000

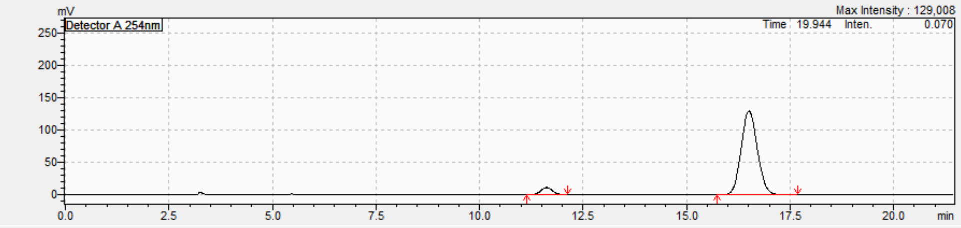

Results View - Peak Table

Peak Table | Compound | Group | Calibration Curve

Peak#	Ret. Time	Area	Height	Mark	Conc.	Unit	ID#	Name	Area%
1	11.623	184987	10458	M	5.005				5.005
2	16.507	3511127	128917		94.995				94.995
Total		3696114	139375		100.000				100.000

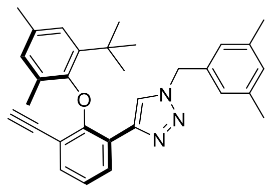

3s

4-(2-(2-(*tert*-Butyl)-4,6-dimethylphenoxy)-3-ethynylphenyl)-1-(3,5-dimethylbenzyl)-1H-1,2,3-triazole (3s)

73% yield; $[\alpha]_D^{25}$ = -53.9 (c 0.5, $CHCl_3$), a white foam, R_f = 0.36 (hexane/ethyl acetate 8:1). ^1H NMR (400 MHz, $CDCl_3$) δ 8.42 (dd, *J* = 7.8, 1.8 Hz, 1H), 7.97 (s, 1H), 7.25 (dd, *J* = 7.7, 1.8 Hz, 1H), 7.05 – 6.67 (m, 6H), 5.49 (d, *J* = 14.8 Hz, 1H), 5.26 (d, *J* = 14.9 Hz, 1H), 2.58 (s, 1H), 2.24 (s, 3H), 2.17 (s, 6H), 1.73 (s, 3H), 1.20 (s, 9H). ^{13}C NMR (101 MHz, $CDCl_3$) δ 154.1, 151.0, 143.3, 141.9, 138.7, 135.8, 134.6, 133.9, 130.5, 130.2, 129.2, 129.1, 125.6, 125.3, 123.3, 121.9, 121.6, 110.5, 82.8, 78.5, 54.2, 34.9, 31.0, 21.2, 21.1, 17.2. HRMS (ESI) m/z calcd for $C_{31}H_{34}N_3O$ $[M+H]^+$ = 464.2696, found = 464.2709; the ee value was 90%, t_R (minor) = 12.6 min, t_R (major) = 17.1 min (Chiralpak IC, λ = 254 nm, 10% *i*-PrOH/Hexane, flow rate = 1.0 mL/min).

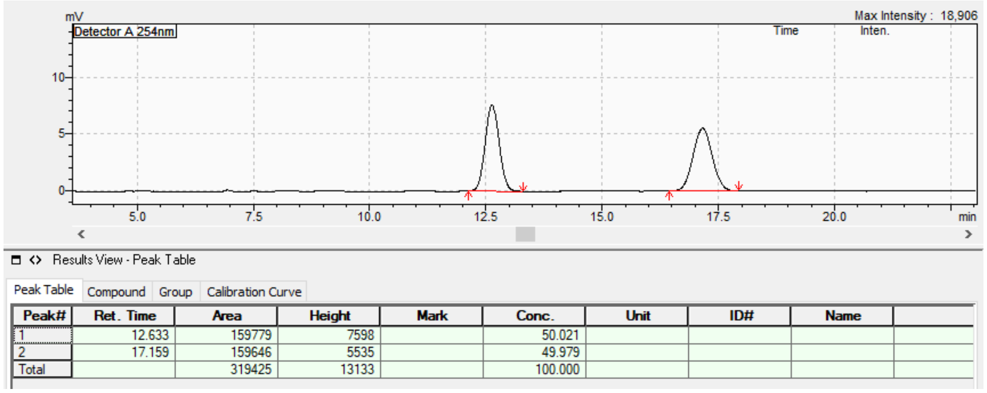

Peak#	Ret. Time	Area	Height	Mark	Conc.	Unit	ID#	Name
1	12.633	159779	7598		50.021			
2	17.159	159646	5535		49.979			
Total		319425	13133		100.000			

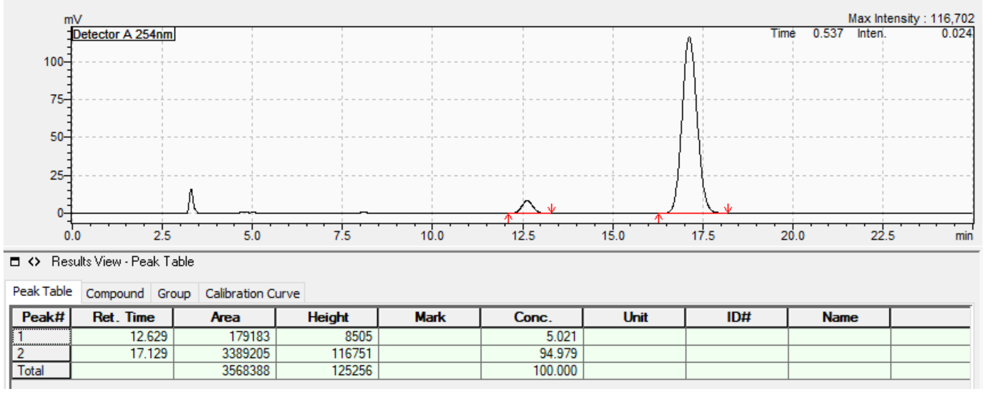

Peak#	Ret. Time	Area	Height	Mark	Conc.	Unit	ID#	Name
1	12.629	179183	8505		5.021			
2	17.129	3389205	116751		94.979			
Total		3568388	125256		100.000			

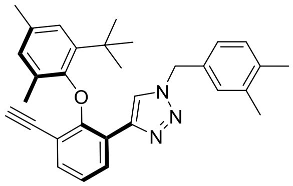

3t

4-(2-(2-(*tert*-Butyl)-4,6-dimethylphenoxy)-3-ethynylphenyl)-1-(3,4-dimethylbenzyl)-1*H*-1,2,3-triazole (3t)

75% yield; $[\alpha]_D^{25}$ = -91.0 (c 0.5, $CHCl_3$), a white foam, R_f = 0.33 (hexane/ethyl acetate 8:1). ^1H NMR (400 MHz, $CDCl_3$) δ 8.41 (dd, *J* = 7.8, 1.8 Hz, 1H), 7.94 (s, 1H), 7.24 (dd, *J* = 7.7, 1.8 Hz, 1H), 7.02 – 6.85 (m, 5H), 6.69 (dt, *J* = 2.4, 0.8 Hz, 1H), 5.49 (d, *J* = 14.8 Hz, 1H), 5.28 (d, *J* = 14.8 Hz, 1H), 2.57 (s, 1H), 2.24 (s, 3H), 2.14 (s, 3H), 2.12 (s, 3H), 1.72 (s, 3H), 1.19 (s, 9H). ^{13}C NMR (101 MHz, $CDCl_3$) δ 154.1, 151.0, 143.2, 141.9, 137.3, 137.1, 135.8, 133.9, 132.1, 130.5, 130.2, 129.20, 129.15, 125.4, 125.3, 123.2, 121.9, 121.6, 110.5, 82.8, 78.5, 54.1, 34.9, 31.0, 21.1, 19.7, 19.4, 17.2. HRMS (ESI) m/z calcd for $C_{31}H_{34}N_3O$ $[M+H]^+$ = 464.2696, found = 464.2708; the ee value was 91%, t_R (minor) = 14.4 min, t_R (major) = 19.4 min (Chiralpak IC, λ = 254 nm, 10% *i*-PrOH/Hexane, flow rate = 1.0 mL/min).

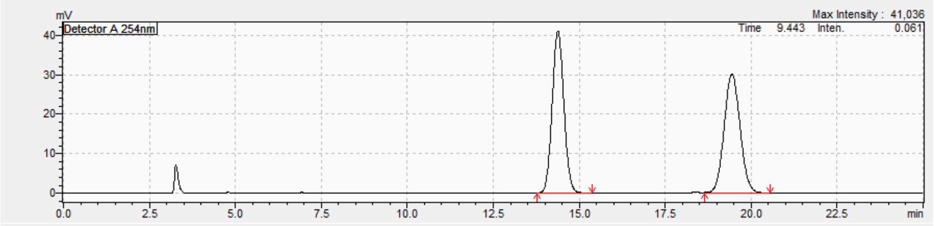

Results View - Peak Table

Peak Table | Compound | Group | Calibration Curve

Peak#	Ret. Time	Area	Height	Mark	Conc.	Unit	ID#	Name	Area%
1	14.380	968200	40931	V	50.116				50.116
2	19.441	963736	30040		49.884				49.884
Total		1931936	70971		100.000				100.000

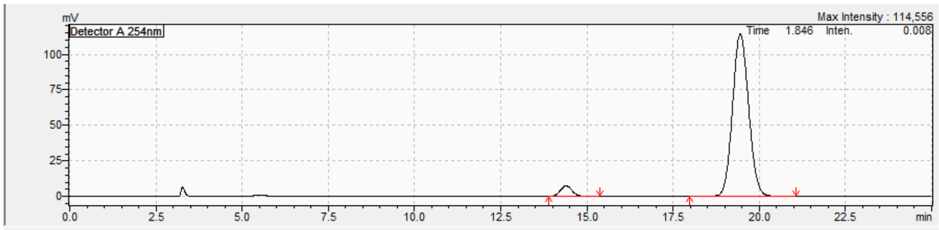

Results View - Peak Table

Peak Table | Compound | Group | Calibration Curve

Peak#	Ret. Time	Area	Height	Mark	Conc.	Unit	ID#	Name	Area%
1	14.396	176214	7389		4.538				4.538
2	19.449	3707164	114412		95.462				95.462
Total		3883378	121801		100.000				100.000

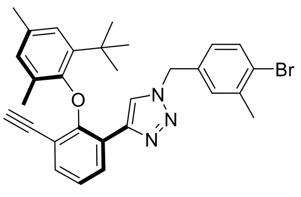

3u

1-(4-Bromo-3-methylbenzyl)-4-(2-(2-(*tert*-butyl)-4,6-dimethylphenoxy)-3-ethynylphenyl)-1*H*-1,2,3-triazole (3u)

78% yield; $[\alpha]_D^{25}$ = -71.4 (c 0.5, $CHCl_3$), a white foam, R_f = 0.35 (hexane/ethyl acetate 8:1). ^1H NMR (400 MHz, $CDCl_3$) δ 8.41 (dd, *J* = 7.8, 1.8 Hz, 1H), 7.96 (s, 1H), 7.38 (d, *J* = 8.1 Hz, 1H), 7.25 (dd, *J* = 7.6, 1.5 Hz, 1H), 7.05 – 6.96 (m, 2H), 6.91 (d, *J* = 2.2 Hz, 1H), 6.84 (dd, *J* = 8.2, 2.3 Hz, 1H), 6.69 (d, *J* = 2.2 Hz, 1H), 5.49 (d, *J* = 15.0 Hz, 1H), 5.27 (d, *J* = 15.0 Hz, 1H), 2.58 (s, 1H), 2.26 (s, 3H), 2.24 (s, 3H), 1.73 (s, 3H), 1.19 (s, 9H). ^{13}C NMR (101 MHz, $CDCl_3$) δ 154.1, 151.0, 141.9, 138.8, 135.9, 134.0, 134.0, 133.0, 130.4, 130.2, 129.2, 129.1, 126.7, 125.4, 122.0, 121.5, 110.5, 82.9, 78.4, 53.5, 35.0, 31.0, 22.9, 21.1, 17.2. HRMS (ESI) m/z calcd for $C_{30}H_{31}BrN_3O$ $[M+H]^+$ = 528.1645, found = 528.1647; the ee value was 92%, t_R (minor) = 12.0 min, t_R (major) = 16.4 min (Chiralpak IC, λ = 254 nm, 10% *i*-PrOH/Hexane, flow rate = 1.0 mL/min).

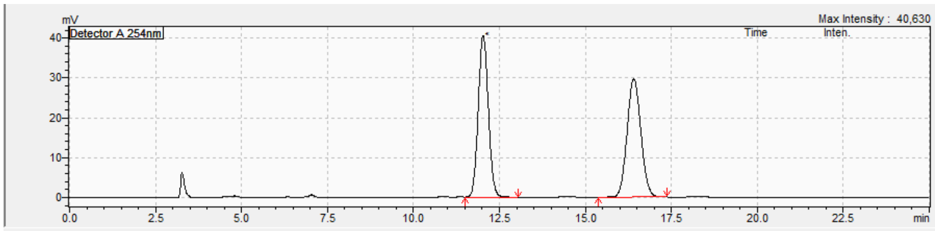

Results View - Peak Table

Peak Table | Compound | Group | Calibration Curve

Peak#	Ret. Time	Area	Height	Mark	Conc.	Unit	ID#	Name	Area%
1	12.028	830630	40530		50.014				50.014
2	16.409	830155	29710		49.986				49.986
Total		1660786	70240		100.000				100.000

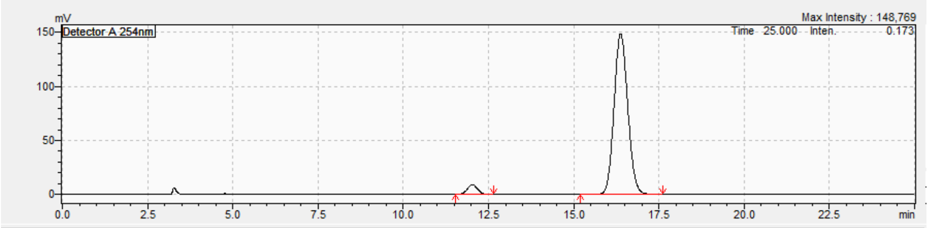

Results View - Peak Table

Peak Table | Compound | Group | Calibration Curve

Peak#	Ret. Time	Area	Height	Mark	Conc.	Unit	ID#	Name	Area%
1	12.026	184736	9046		4.258				4.258
2	16.375	4154313	148608		95.742				95.742
Total		4339049	157654		100.000				100.000

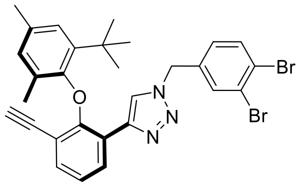

3v

4-(2-(2-(*tert*-Butyl)-4,6-dimethylphenoxy)-3-ethynylphenyl)-1-(3,4-dibromobenzyl)-1*H*-1,2,3-triazole (3v)

68% yield; $[\alpha]_D^{25}$ = -32.1 (c 0.5, $CHCl_3$), a white foam, R_f = 0.31 (hexane/ethyl acetate 8:1). ^1H NMR (400 MHz, $CDCl_3$) δ 8.41 (dd, *J* = 7.8, 1.8 Hz, 1H), 7.98 (s, 1H), 7.49 (d, *J* = 8.3 Hz, 1H), 7.42 (d, *J* = 2.2 Hz, 1H), 7.27 (dd, *J* = 7.7, 1.8 Hz, 1H), 7.09 – 6.90 (m, 3H), 6.77 – 6.65 (m, 1H), 5.51 (d, *J* = 15.3 Hz, 1H), 5.29 (d, *J* = 15.3 Hz, 1H), 2.59 (s, 1H), 2.25 (s, 3H), 1.74 (s, 3H), 1.21 (s, 9H). ^{13}C NMR (101 MHz, $CDCl_3$) δ 154.1, 150.9, 143.8, 141.9, 136.0, 135.7, 134.2, 134.1, 132.8, 130.4, 129.2, 129.1, 127.8, 125.4, 123.2, 122.0, 121.3, 110.5, 83.0, 78.4, 52.8, 35.0, 31.0, 21.1, 17.3. HRMS (ESI) m/z calcd for $C_{29}H_{28}Br_2N_3O$ $[M+H]^+$ = 592.0594, found = 592.0597; the ee value was 98%, t_R (minor) = 12.5 min, t_R (major) = 18.2 min (Chiralpak IC, λ = 254 nm, 10% *i*-PrOH/Hexane, flow rate = 1.0 mL/min).

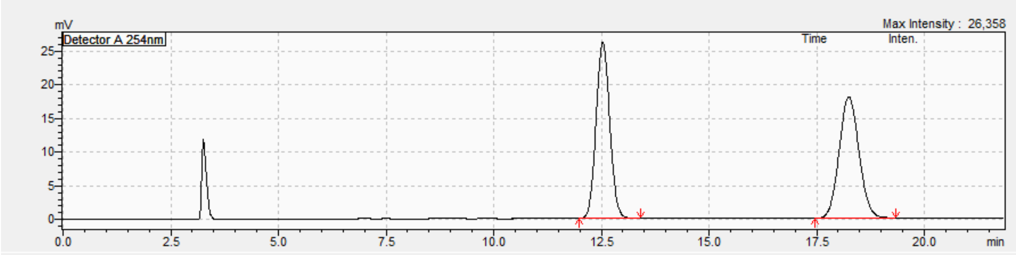

Results View - Peak Table

Peak Table | Compound | Group | Calibration Curve

Peak#	Ret. Time	Area	Height	Mark	Conc.	Unit	ID#	Name	Area%
1	12.533	576367	26181	V	50.017				50.017
2	18.248	575981	18012		49.983				49.983
Total		1152348	44193		100.000				100.000

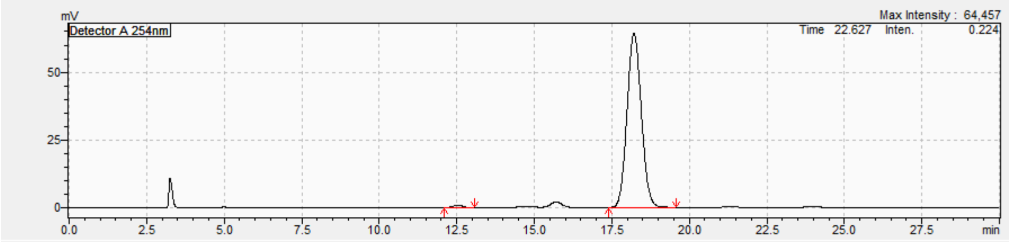

Results View - Peak Table

Peak Table | Compound | Group | Calibration Curve

Peak#	Ret. Time	Area	Height	Mark	Conc.	Unit	ID#	Name	Area%
1	12.529	18484	846		0.893				0.893
2	18.215	2050763	64227		99.107				99.107
Total		2069247	65074		100.000				100.000

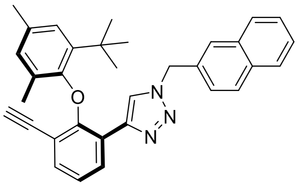

3w

4-(2-(2-(*tert*-Butyl)-4,6-dimethylphenoxy)-3-ethynylphenyl)-1-(naphthalen-2-ylmethyl)-1*H*-1,2,3-triazole (3w)

82% yield; $[\alpha]_D^{25}$ = -91.1 (c 0.5, $CHCl_3$), a white foam, R_f = 0.33 (hexane/ethyl acetate 8:1). ^{1}H NMR (400 MHz, $CDCl_3$) δ 8.42 (dd, *J* = 7.8, 1.8 Hz, 1H), 8.02 (s, 1H), 7.72 (dd, *J* = 7.4, 4.4 Hz, 3H), 7.64 – 7.57 (m, 1H), 7.47 – 7.37 (m, 2H), 7.30 – 7.22 (m, 2H), 6.99 (t, *J* = 7.7 Hz, 1H), 6.85 (d, *J* = 2.2 Hz, 1H), 6.66 (d, *J* = 2.2 Hz, 1H), 5.73 (d, *J* = 15.0 Hz, 1H), 5.52 (d, *J* = 15.0 Hz, 1H), 2.56 (s, 1H), 2.22 (s, 3H), 1.71 (s, 3H), 1.11 (s, 9H). ^{13}C NMR (101 MHz, $CDCl_3$) δ 154.1, 151.1, 143.5, 141.9, 135.9, 133.9, 133.2, 133.1, 132.2, 130.4, 129.2, 129.1, 129.1, 128.0, 127.7, 127.0, 126.6, 126.6, 125.3, 125.1, 123.4, 121.9, 121.5, 110.5, 82.8, 78.5, 54.4, 34.9, 30.9, 21.1, 17.3. HRMS (ESI) m/z calcd for $C_{33}H_{32}N_3O$ $[M+H]^+$ = 486.2540, found = 486.2542; the ee value was 90%, t_R (minor) = 16.0 min, t_R (major) = 20.8 min (Chiralpak IC, λ = 254 nm, 10% *i*-PrOH/Hexane, flow rate = 1.0 mL/min).

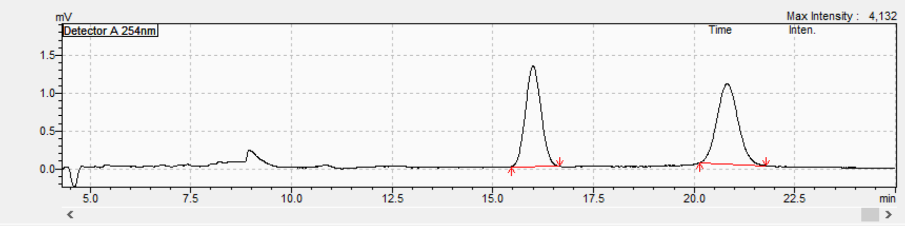

Results View - Peak Table

Peak Table | Compound | Group | Calibration Curve

Peak#	Ret. Time	Area	Height	Conc.	Unit	ID#	Name	Area%
1	15.999	35897	1328	48.297				48.297
2	20.822	38428	1059	51.703				51.703
Total		74325	2386	100.000				100.000

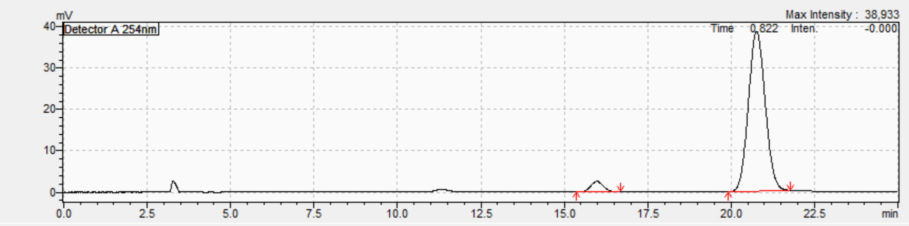

Results View - Peak Table

Peak Table | Compound | Group | Calibration Curve

Peak#	Ret. Time	Area	Height	Conc.	Unit	ID#	Name	Area%
1	15.978	69069	2566	4.846				4.846
2	20.765	1356142	38695	95.154				95.154
Total		1425212	41261	100.000				100.000

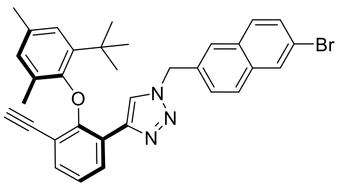

3x

1-((6-Bromonaphthalen-2-yl)methyl)-4-(2-(2-(*tert*-butyl)-4,6-dimethylphenoxy)-3-ethynylphenyl)-1*H*-1,2,3-triazole (3x)

77% yield; $[\alpha]_D^{25}$ = -86.3 (c 0.5, $CHCl_3$), a white foam, R_f = 0.33 (hexane/ethyl acetate 8:1). ^1H NMR (400 MHz, $CDCl_3$) δ 8.42 (d, *J* = 7.8 Hz, 1H), 8.01 (d, *J* = 1.7 Hz, 1H), 7.90 (s, 1H), 7.62 (d, *J* = 8.5 Hz, 1H), 7.55 (d, *J* = 9.6 Hz, 2H), 7.48 (dd, *J* = 8.7, 1.8 Hz, 1H), 7.26 (t, *J* = 8.6 Hz, 2H), 7.04 – 6.95 (m, 1H), 6.87 (s, 1H), 6.67 (s, 1H), 5.72 (d, *J* = 15.1 Hz, 1H), 5.50 (d, *J* = 15.1 Hz, 1H), 2.57 (s, 1H), 2.22 (s, 3H), 1.72 (s, 3H), 1.12 (s, 9H). ^{13}C NMR (101 MHz, $CDCl_3$) δ 154.1, 150.1, 141.9, 135.9, 134.1, 134.0, 132.8, 131.6, 130.4, 130.0, 129.8, 129.6, 129.2, 129.1, 128.2, 126.9, 126.2, 125.4, 123.4, 122.0, 121.5, 110.5, 82.9, 78.4, 54.1, 34.9, 30.9, 21.1, 17.3. HRMS (ESI) m/z calcd for $C_{33}H_{31}BrN_3O$ $[M+H]^+$ = 564.1645, found = 564.1647; the ee value was 90%, t_R (minor) = 16.4 min, t_R (major) = 21.5 min (Chiralpak IC, λ = 254 nm, 10% *i*-PrOH/Hexane, flow rate = 1.0 mL/min).

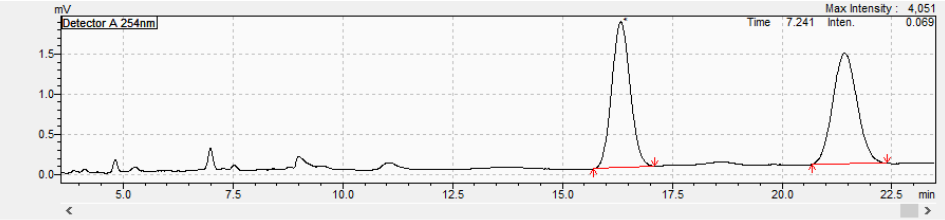

Results View - Peak Table

Peak Table | Compound | Group | Calibration Curve

Peak#	Ret. Time	Area	Height	Mark	Conc.	Unit	ID#	Name	Area%
1	16.327	53156	1819		50.024				50.024
2	21.418	53105	1380		49.976				49.976
Total		106261	3198		100.000				100.000

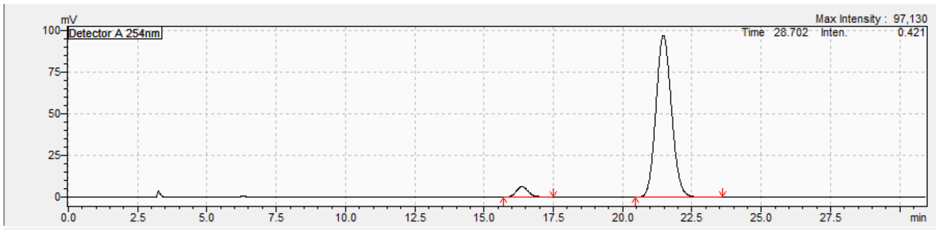

Results View - Peak Table

Peak Table | Compound | Group | Calibration Curve

Peak#	Ret. Time	Area	Height	Mark	Conc.	Unit	ID#	Name	Area%
1	16.367	195886	6328		4.901				4.901
2	21.470	3800929	97088		95.099				95.099
Total		3996815	103416		100.000				100.000

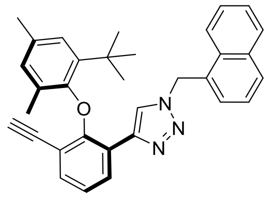

3y

4-(2-(2-(*tert*-Butyl)-4,6-dimethylphenoxy)-3-ethynylphenyl)-1-(naphthalen-1-ylmethyl)-1H-1,2,3-triazole (3y)

78% yield; $[\alpha]_D^{25}$ = -61.8 (c 0.5, $CHCl_3$), a white foam, R_f = 0.30 (hexane/ethyl acetate 8:1). ^1H NMR (400 MHz, $CDCl_3$) δ 8.40 (dd, *J* = 7.9, 1.8 Hz, 1H), 7.99 – 7.86 (m, 2H), 7.82 – 7.72 (m, 2H), 7.47 – 7.38 (m, 2H), 7.37 – 7.20 (m, 3H), 6.97 (t, *J* = 7.7 Hz, 1H), 6.84 (d, *J* = 2.2 Hz, 1H), 6.64 (d, *J* = 2.2 Hz, 1H), 6.12 (d, *J* = 15.0 Hz, 1H), 5.74 (d, *J* = 15.0 Hz, 1H), 2.54 (s, 1H), 2.21 (s, 3H), 1.65 (s, 3H), 1.03 (s, 9H). ^{13}C NMR (101 MHz, $CDCl_3$) δ 154.1, 151.0, 141.9, 135.8, 133.9, 133.8, 131.0, 130.3, 130.0, 129.8, 129.1, 129.1, 128.95, 127.4, 127.2, 126.3, 125.3, 125.2, 122.8, 121.9, 121.5, 110.5, 82.8, 78.4, 52.4, 34.8, 30.9, 21.1, 17.2. HRMS (ESI) m/z calcd for $C_{33}H_{32}N_3O$ $[M+H]^+$ = 486.2540, found = 486.2543; the ee value was 93%, t_R (minor) = 14.3 min, t_R (major) = 23.1 min (Chiralpak IC, λ = 254 nm, 10% *i*-PrOH/Hexane, flow rate = 1.0 mL/min).

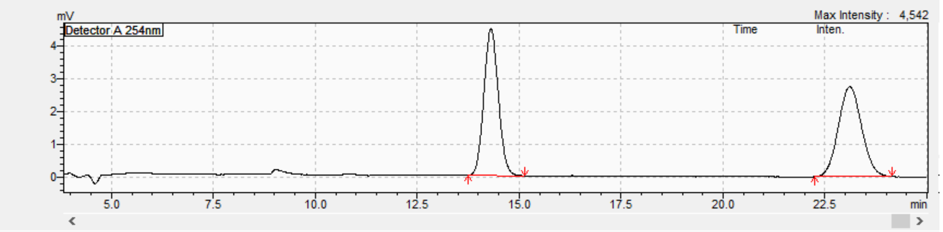

Results View - Peak Table

Peak Table | Compound | Group | Calibration Curve

Peak#	Ret. Time	Area	Height	Conc.	Unit	ID#	Name	Area%
1	14.307	110805	4498	50.251				50.251
2	23.108	109697	2761	49.749				49.749
Total		220502	7259	100.000				100.000

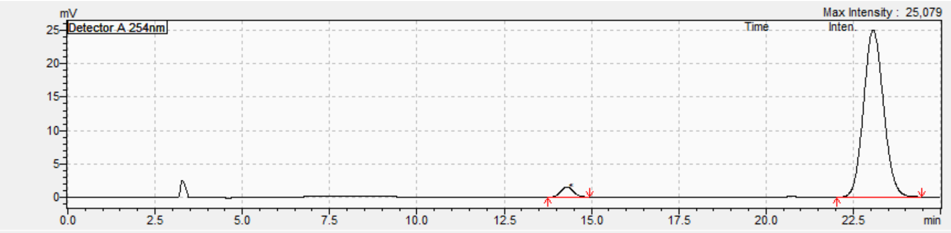

Results View - Peak Table

Peak Table | Compound | Group | Calibration Curve

Peak#	Ret. Time	Area	Height	Conc.	Unit	ID#	Name	Area%
1	14.300	36961	1520	3.576				3.576
2	23.071	996579	25045	96.424				96.424
Total		1033541	26565	100.000				100.000

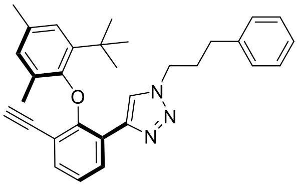

3z

4-(2-(2-(*tert*-Butyl)-4,6-dimethylphenoxy)-3-ethynylphenyl)-1-(3-phenylpropyl)-1*H*-1,2,3-triazole (3z)

83% yield; $[\alpha]_D^{25}$ = -55.8 (c 0.5, $CHCl_3$), a white foam, R_f = 0.32 (hexane/ethyl acetate 8:1). ^1H NMR (400 MHz, $CDCl_3$) δ 8.42 (d, *J* = 7.7 Hz, 1H), 8.02 (s, 1H), 7.31 – 6.91 (m, 9H), 6.72 (s, 1H), 4.39 – 4.18 (m, 2H), 2.64 – 2.48 (m, 3H), 2.26 (s, 3H), 2.22 – 2.09 (m, 2H), 1.77 (s, 3H), 1.30 (s, 9H). ^{13}C NMR (101 MHz, $CDCl_3$) δ 154.1, 151.0, 141.9, 140.1, 135.8, 134.0, 130.4, 129.3, 129.1, 128.6, 128.4, 126.3, 125.4, 122.0, 121.7, 110.5, 82.9, 49.5, 35.1, 32.5, 31.8, 31.2, 21.1, 17.3. HRMS (ESI) m/z calcd for $C_{31}H_{34}N_3O$ $[M+H]^+$ = 464.2696, found = 464.2696; the ee value was 85%, t_R (minor) = 10.1 min, t_R (major) = 13.6 min (Chiralpak IC, λ = 254 nm, 10% *i*-PrOH/Hexane, flow rate = 1.0 mL/min).

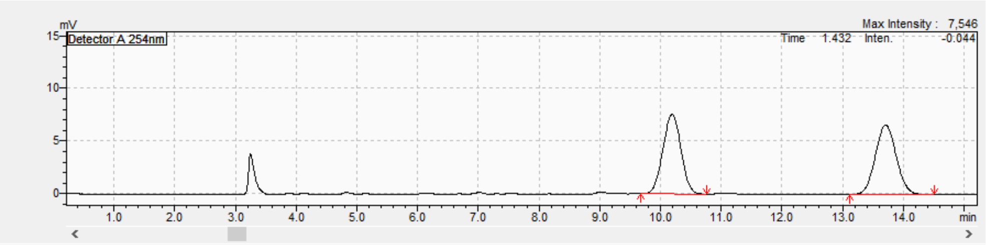

Results View - Peak Table

Peak Table | Compound | Group | Calibration Curve

Peak#	Ret. Time	Area	Height	Mark	Conc.	Unit	ID#	Name	Area%
1	10.192	157050	7580		49.844				49.844
2	13.710	158031	6589		50.156				50.156
Total		315081	14169		100.000				100.000

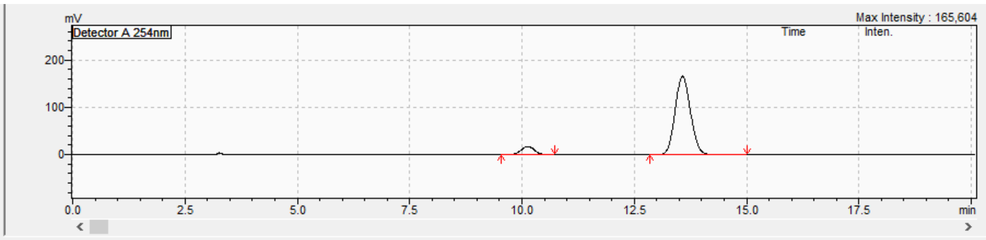

Results View - Peak Table

Peak Table | Compound | Group | Calibration Curve

Peak#	Ret. Time	Area	Height	Mark	Conc.	Unit	ID#	Name	Area%
1	10.131	323437	16711	M	7.509				7.509
2	13.574	3984164	166013	M	92.491				92.491
Total		4307601	182724		100.000				100.000

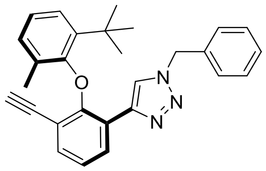

3aa

1-Benzyl-4-(2-(2-(*tert*-butyl)-6-methylphenoxy)-3-ethynylphenyl)-1*H*-1,2,3-triazole (3aa)

73% yield; $[\alpha]_D^{25}$ = -65.1 (c 0.5, $CHCl_3$), a white foam, R_f = 0.30 (hexane/ethyl acetate 8:1). ^{1}H NMR (400 MHz, $CDCl_3$) δ 8.42 (dd, *J* = 7.8, 1.8 Hz, 1H), 7.97 (s, 1H), 7.29 – 7.14 (m, 6H), 7.09 (dd, *J* = 7.9, 1.8 Hz, 1H), 6.99 (dt, *J* = 12.4, 7.8 Hz, 2H), 6.91 – 6.81 (m, 1H), 5.60 (d, *J* = 14.9 Hz, 1H), 5.36 (d, *J* = 14.9 Hz, 1H), 2.56 (s, 1H), 1.76 (s, 3H), 1.19 (s, 9H). ^{13}C NMR (101 MHz, $CDCl_3$) δ 153.9, 153.3, 143.4, 142.3, 135.8, 134.8, 130.8, 129.2, 129.1, 128.8, 128.61, 127.8, 124.6, 123.3, 122.1, 121.6, 110.5, 82.7, 78.3, 54.2, 35.1, 31.0, 17.3. HRMS (ESI) m/z calcd for $C_{28}H_{28}N_3O$ $[M+H]^+$ = 422.2227, found = 422.2227; the ee value was 99%, t_R (minor) = 12.2 min, t_R (major) = 13.8 min (Chiralpak IC, λ = 254 nm, 10% *i*-PrOH/Hexane, flow rate = 1.0 mL/min).

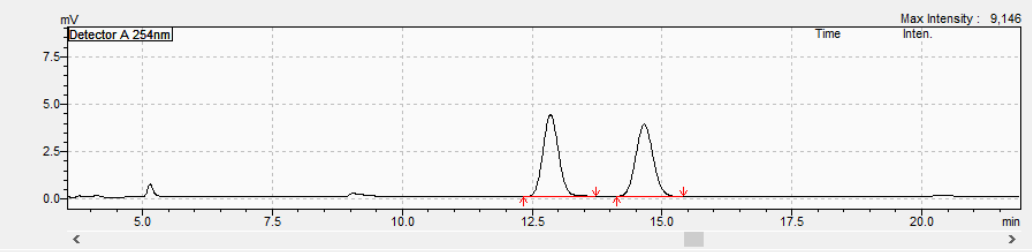

Results View - Peak Table

Peak Table | Compound | Group | Calibration Curve

Peak#	Ret. Time	Area	Height	Mark	Conc.	Unit	ID#	Name	Area%
1	12.854	90024	4321		50.334				50.334
2	14.658	88830	3806		49.666				49.666
Total		178854	8127		100.000				100.000

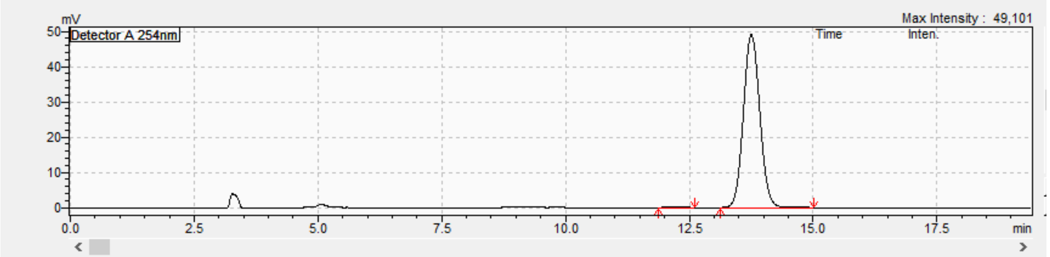

Results View - Peak Table

Peak Table | Compound | Group | Calibration Curve

Peak#	Ret. Time	Area	Height	Conc.	Unit	ID#	Name	Area%
1	12.222	4465	229	0.394				0.394
2	13.754	1127659	49049	99.606				99.606
Total		1132123	49278	100.000				100.000

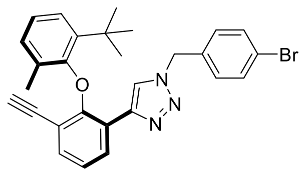

3ab

1-(4-Bromobenzyl)-4-(2-(2-(*tert*-butyl)-6-methylphenoxy)-3-ethynylphenyl)-1*H*-1,2,3-triazole (3ab)

81% yield; $[\alpha]_D^{25}$ = -67.3 (c 0.5, $CHCl_3$), a white foam, R_f = 0.33 (hexane/ethyl acetate 8:1). ^1H NMR (400 MHz, $CDCl_3$) δ 8.41 (dd, *J* = 7.9, 1.7 Hz, 1H), 7.96 (d, *J* = 0.9 Hz, 1H), 7.43 – 7.35 (m, 2H), 7.26 (dt, *J* = 7.6, 1.4 Hz, 1H), 7.11 (dd, *J* = 7.9, 1.7 Hz, 1H), 7.08 – 6.95 (m, 4H), 6.87 (dd, *J* = 7.4, 1.7 Hz, 1H), 5.54 (d, *J* = 15.1 Hz, 1H), 5.32 (d, *J* = 15.1 Hz, 1H), 2.56 (s, 1H), 1.77 (s, 3H), 1.21 (s, 9H). ^{13}C NMR (101 MHz, $CDCl_3$) δ 153.9, 153.2, 143.5, 142.3, 135.9, 133.8, 132.2, 130.8, 129.5, 129.2, 128.9, 124.7, 123.2, 122.8, 122.1, 121.4, 110.6, 82.8, 78.3, 53.5, 35.1, 31.0, 17.4. HRMS (ESI) m/z calcd for $C_{28}H_{27}BrN_3O$ $[M+H]^+$ = 500.1332, found = 500.1335; the ee value was 97%, t_R (minor) = 12.7 min, t_R (major) = 14.5 min (Chiralpak IC, λ = 254 nm, 10% *i*-PrOH/Hexane, flow rate = 1.0 mL/min).

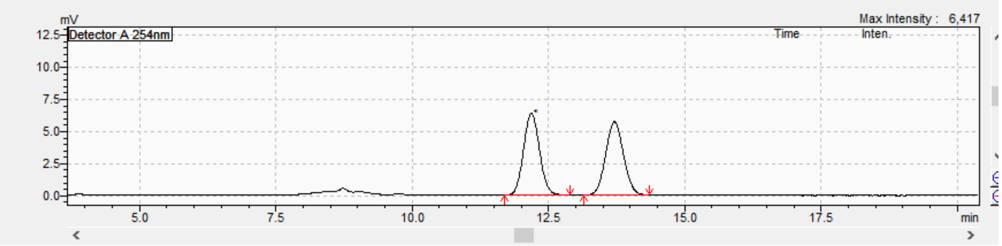

Results View - Peak Table

Peak Table | Compound | Group | Calibration Curve

Peak#	Ret. Time	Area	Height	Conc.	Unit	ID#	Name	Area%
1	12.187	130418	6370	50.045				50.045
2	13.712	130186	5702	49.955				49.955
Total		260604	12072	100.000				100.000

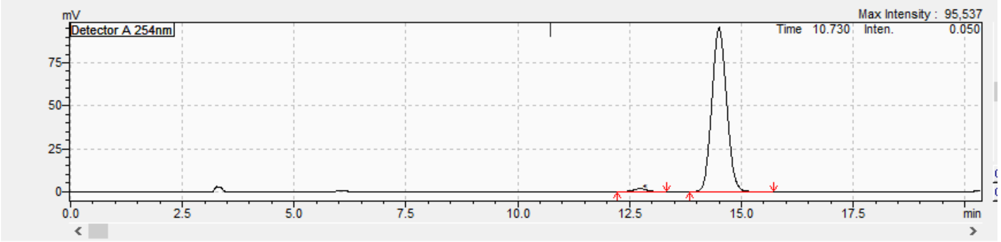

Results View - Peak Table

Peak Table | Compound | Group | Calibration Curve

Peak#	Ret. Time	Area	Height	Conc.	Unit	ID#	Name	Area%
1	12.735	36234	1755	1.628				1.628
2	14.501	2189159	95505	98.372				98.372
Total		2225393	97260	100.000				100.000

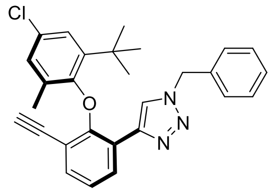

3ac

1-Benzyl-4-(2-(2-(*tert*-butyl)-4-chloro-6-methylphenoxy)-3-ethynylphenyl)-1*H*-1,2,3-triazole (3ac)

75% yield; $[\alpha]_D^{25}$ = -76.0 (c 0.5, $CHCl_3$), a white foam, R_f = 0.30 (hexane/ethyl acetate 8:1). ^1H NMR (400 MHz, $CDCl_3$) δ 8.41 (d, *J* = 7.9 Hz, 1H), 7.89 (s, 1H), 7.29 – 7.22 (m, 4H), 7.22 – 7.15 (m, 2H), 7.12 – 7.01 (m, 2H), 6.87 (d, *J* = 2.6 Hz, 1H), 5.60 (d, *J* = 14.9 Hz, 1H), 5.37 (d, *J* = 14.9 Hz, 1H), 2.66 (s, 1H), 1.72 (s, 3H), 1.17 (s, 9H). ^{13}C NMR (101 MHz, $CDCl_3$) δ 153.4, 151.7, 144.1, 135.9, 134.6, 132.4, 129.6, 129.4, 129.1, 128.7, 128.3, 127.9, 124.8, 122.4, 121.6, 110.4, 83.5, 78.2, 54.3, 35.3, 30.7, 17.3. HRMS (ESI) m/z calcd for $C_{28}H_{27}ClN_3O$ $[M+H]^+$ = 456.1837, found = 456.1843; the ee value was 96%, t_R (minor) = 12.4 min, t_R (major) = 14.9 min (Chiralpak IC, λ = 254 nm, 10% *i*-PrOH/Hexane, flow rate = 1.0 mL/min).

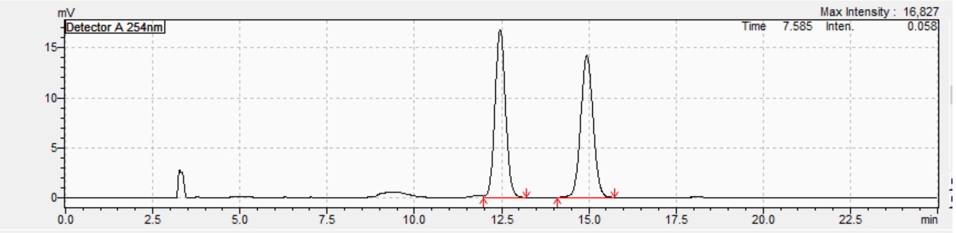

Results View - Peak Table

Peak Table | Compound | Group | Calibration Curve

Peak#	Ret. Time	Area	Height	Conc.	Unit	ID#	Name	Area%
1	12.458	351083	16764	50.173				50.173
2	14.945	348660	14137	49.827				49.827
Total		699742	30901	100.000				100.000

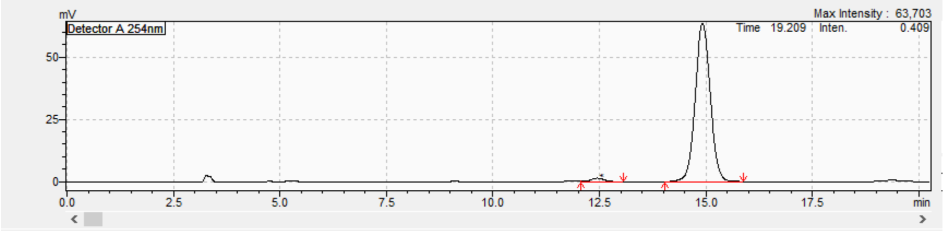

Results View - Peak Table

Peak Table | Compound | Group | Calibration Curve

Peak#	Ret. Time	Area	Height	Conc.	Unit	ID#	Name	Area%
1	12.449	30032	1410	1.884				1.884
2	14.922	1563811	63700	98.116				98.116
Total		1593843	65110	100.000				100.000

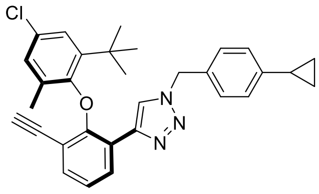

3ad

4-(2-(2-(*tert*-Butyl)-4-chloro-6-methylphenoxy)-3-ethynylphenyl)-1-(4-cyclopropylbenzyl)-1*H*-1,2,3-triazole (3ad)

69% yield; $[\alpha]_D^{25}$ = -86.1 (c 0.5, $CHCl_3$), a white foam, R_f = 0.33 (hexane/ethyl acetate 8:1). ^{1}H NMR (400 MHz, $CDCl_3$) δ 8.39 (dd, *J* = 7.8, 1.8 Hz, 1H), 7.84 (s, 1H), 7.25 (dd, *J* = 7.7, 1.8 Hz, 1H), 7.14 – 6.69 (m, 7H), 5.54 (d, *J* = 14.8 Hz, 1H), 5.31 (d, *J* = 14.8 Hz, 1H), 2.66 (s, 1H), 1.79 (td, *J* = 8.5, 4.2 Hz, 1H), 1.72 (s, 3H), 1.17 (s, 9H), 0.94 – 0.86 (m, 2H), 0.57 (dt, *J* = 6.6, 4.7 Hz, 2H). ^{13}C NMR (101 MHz, $CDCl_3$) δ 153.4, 151.7, 144.8, 144.1, 143.0, 135.8, 132.41 131.5, 129.5, 129.4, 128.3, 128.0, 126.3, 124.8, 123.0, 122.4, 121.7, 110.5, 83.5, 78.3, 54.0, 35.3, 30.7, 17.3, 15.1, 9.4. HRMS (ESI) m/z calcd for $C_{31}H_{31}ClN_3O$ $[M+H]^+$ = 496.2150, found = 496.2155; the ee value was > 99%, t_R (minor) = 14.9 min, t_R (major) = 17.8 min (Chiralpak IC, λ = 254 nm, 10% *i*-PrOH/Hexane, flow rate = 1.0 mL/min).

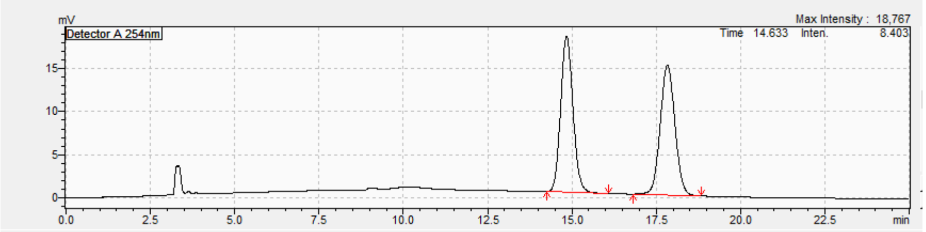

Results View - Peak Table

Peak Table | Compound | Group | Calibration Curve

Peak#	Ret. Time	Area	Height	Conc.	Unit	ID#	Name	Area%
1	14.844	461276	18111	50.193				50.193
2	17.832	457733	15070	49.807				49.807
Total		919009	33181	100.000				100.000

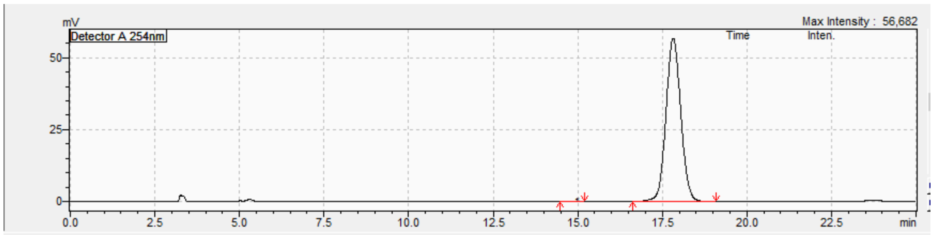

Results View - Peak Table

Peak Table | Compound | Group | Calibration Curve

Peak#	Ret. Time	Area	Height	Conc.	Unit	ID#	Name	Area%
1	14.867	3590	173	0.207				0.207
2	17.821	1727945	56687	99.793				99.793
Total		1731536	56860	100.000				100.000

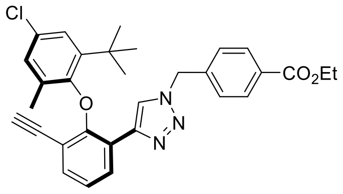

3ae

Ethyl 4-((4-(2-(2-(*tert*-butyl)-4-chloro-6-methylphenoxy)-3-ethynylphenyl)-1*H*-1,2,3-triazol-1-yl)methyl)benzoate (3ae)

71% yield; $[\alpha]_D^{25}$ = -81.9 (c 0.5, $CHCl_3$), a white foam, R_f = 0.23 (hexane/ethyl acetate 8:1). ^1H NMR (400 MHz, $CDCl_3$) δ 8.41 (dd, *J* = 7.8, 1.8 Hz, 1H), 8.05 – 7.87 (m, 3H), 7.34 – 7.18 (m, 4H), 7.13 – 6.95 (m, 2H), 6.87 (d, *J* = 2.6 Hz, 1H), 5.65 (d, *J* = 15.3 Hz, 1H), 5.45 (d, *J* = 15.3 Hz, 1H), 4.30 (q, *J* = 7.1 Hz, 2H), 2.67 (s, 1H), 1.73 (s, 3H), 1.32 (d, *J* = 7.2 Hz, 3H), 1.18 (s, 9H). ^{13}C NMR (101 MHz, $CDCl_3$) δ 165.9, 153.4, 151.6, 144.1, 139.2, 136.0, 132.4, 130.9, 130.3, 129.7, 129.4, 128.3, 127.6, 124.9, 122.5, 121.4, 110.5, 83.6, 78.2, 61.2, 53.7, 35.3, 30.7, 17.3, 14.3. HRMS (ESI) m/z calcd for $C_{31}H_{31}ClN_3O_3$ $[M+H]^+$ = 528.2048, found = 528.2053; the ee value was > 99%, t_R (minor) = 25.1 min, t_R (major) = 31.4 min (Chiralpak IC, λ = 254 nm, 10% *i*-PrOH/Hexane, flow rate = 1.0 mL/min).

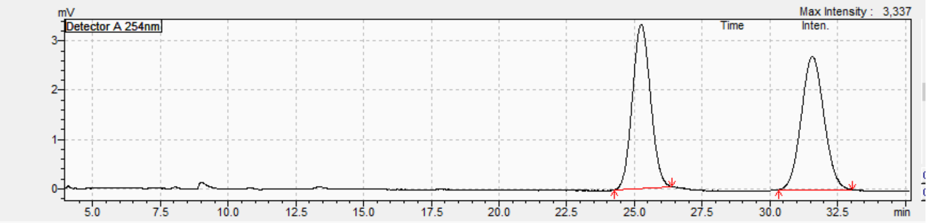

Results View - Peak Table

Peak Table | Compound | Group | Calibration Curve

Peak#	Ret. Time	Area	Height	Conc.	Unit	ID#	Name	Area%
1	25.255	154827	3328	49.538				49.538
2	31.579	157717	2707	50.462				50.462
Total		312544	6035	100.000				100.000

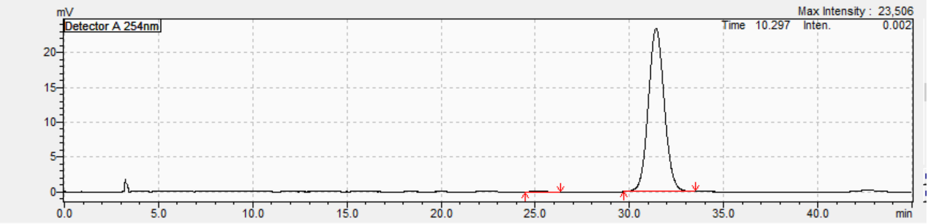

Results View - Peak Table

Peak Table | Compound | Group | Calibration Curve

Peak#	Ret. Time	Area	Height	Conc.	Unit	ID#	Name	Area%
1	25.143	3044	65	0.220				0.220
2	31.415	1378625	23486	99.780				99.780
Total		1381669	23551	100.000				100.000

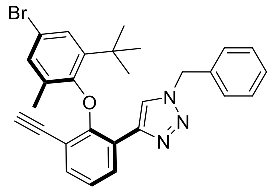

3af

1-Benzyl-4-(2-(4-bromo-2-(*tert*-butyl)-6-methylphenoxy)-3-ethynylphenyl)-1*H*-1,2,3-triazole (3af)

76% yield; $[\alpha]_D^{25}$ = -64.0 (c 0.5, $CHCl_3$), a white foam, R_f = 0.32 (hexane/ethyl acetate 8:1). ^{1}H NMR (400 MHz, $CDCl_3$) δ 8.41 (dd, *J* = 7.8, 1.8 Hz, 1H), 7.87 (s, 1H), 7.32 – 7.15 (m, 7H), 7.09 – 6.92 (m, 2H), 5.60 (d, *J* = 14.9 Hz, 1H), 5.36 (d, *J* = 14.8 Hz, 1H), 2.67 (s, 1H), 1.72 (s, 3H), 1.17 (s, 9H). ^{13}C NMR (101 MHz, $CDCl_3$) δ 153.3, 152.3, 144.5, 143.1, 135.8, 134.7, 132.9, 131.3, 129.4, 129.1, 128.7, 127.9, 127.8, 123.1, 122.5, 121.7, 117.5, 110.5, 83.5, 78.2, 54.2, 35.3, 30.7, 17.2. HRMS (ESI) m/z calcd for $C_{28}H_{27}BrN_3O$ $[M+H]^+$ = 500.1332, found = 500.1336; the ee value was 97%, t_R (minor) = 12.3 min, t_R (major) = 15.2 min (Chiralpak IC, λ = 254 nm, 10% *i*-PrOH/Hexane, flow rate = 1.0 mL/min).

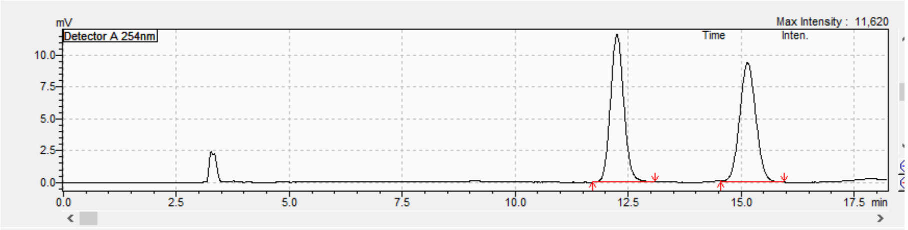

Peak#	Ret. Time	Area	Height	Conc.	Unit	ID#	Name	Area%
1	12.252	238295	11590	50.344				50.344
2	15.144	235040	9392	49.656				49.656
Total		473335	20983	100.000				100.000

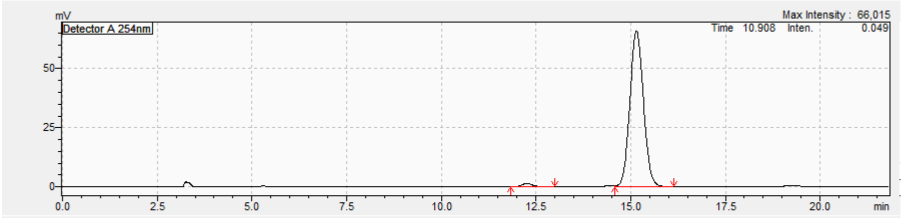

Peak#	Ret. Time	Area	Height	Mark	Conc.	Unit	ID#	Name	Area%
1	12.260	27306	1316		1.633				1.633
2	15.153	1645013	65959		98.367				98.367
Total		1672319	67275		100.000				100.000

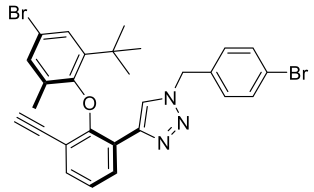

3ag

4-(2-(4-Bromo-2-(*tert*-butyl)-6-methylphenoxy)-3-ethynylphenyl)-1-(4-bromobenzyl)-1*H*-1,2,3-triazole (3ag)

74% yield; $[\alpha]_D^{25}$ = -94.5 (c 0.5, $CHCl_3$), a white foam, R_f = 0.34 (hexane/ethyl acetate 8:1). ^1H NMR (400 MHz, $CDCl_3$) δ 8.40 (dd, *J* = 7.8, 1.8 Hz, 1H), 7.87 (s, 1H), 7.44 – 7.34 (m, 2H), 7.32 – 7.26 (m, 1H), 7.22 (s, 1H), 7.03 (td, *J* = 8.0, 5.0 Hz, 4H), 5.55 (d, *J* = 15.0 Hz, 1H), 5.32 (d, *J* = 15.1 Hz, 1H), 2.67 (s, 1H), 1.73 (s, 3H), 1.18 (s, 9H). ^{13}C NMR (101 MHz, $CDCl_3$) δ 153.3, 152.2, 144.4, 143.32 135.9, 133.7, 132.8, 132.3, 131.4, 129.5, 129.4, 127.9, 123.0, 122.9, 122.5, 121.5, 117.6, 110.5, 83.6, 78.2, 53.5, 35.3, 30.7, 17.2. HRMS (ESI) m/z calcd for $C_{28}H_{26}Br_2N_3O$ $[M+H]^+$ = 578.0437, found = 578.0438; the ee value was 94%, t_R (minor) = 11.8 min, t_R (major) = 14.8 min (Chiralpak IC, λ = 254 nm, 10% *i*-PrOH/Hexane, flow rate = 1.0 mL/min).

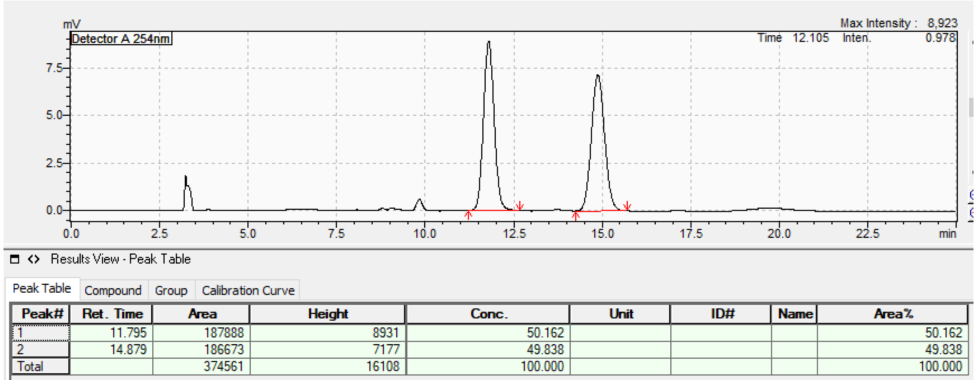

Peak#	Ret. Time	Area	Height	Conc.	Unit	ID#	Name	Area%
1	11.795	187888	8931	50.162				50.162
2	14.879	186673	7177	49.838				49.838
Total		374561	16108	100.000				100.000

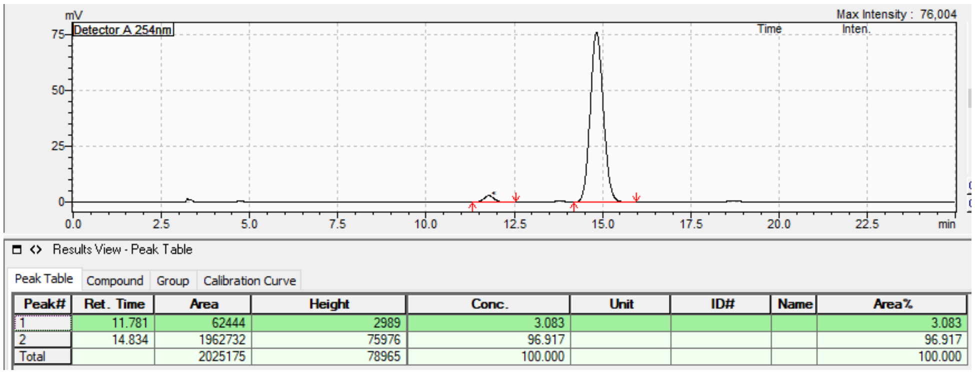

Peak#	Ret. Time	Area	Height	Conc.	Unit	ID#	Name	Area%
1	11.781	62444	2989	3.083				3.083
2	14.834	1962732	75976	96.917				96.917
Total		2025175	78965	100.000				100.000

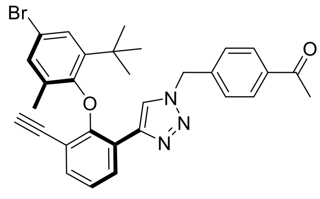

3ah

1-(4-((4-(2-(4-Bromo-2-(*tert*-butyl)-6-methylphenoxy)-3-ethynylphenyl)-1*H*-1,2,3-triazol-1-yl)methyl)phenyl)ethan-1-one (3ah)

82% yield; $[\alpha]_D^{25}$ = -82.5 (c 0.5, $CHCl_3$), a white foam, R_f = 0.30 (hexane/ethyl acetate 8:1). ^{1}H NMR (400 MHz, $CDCl_3$) δ 8.41 (d, *J* = 7.8 Hz, 1H), 7.91 (s, 1H), 7.84 (d, *J* = 8.3 Hz, 2H), 7.33 – 7.18 (m, 4H), 7.11 – 6.96 (m, 2H), 5.65 (d, *J* = 15.3 Hz, 1H), 5.45 (d, *J* = 15.3 Hz, 1H), 2.68 (s, 1H), 2.51 (s, 3H), 1.73 (s, 3H), 1.17 (s, 9H). ^{13}C NMR (101 MHz, $CDCl_3$) δ 197.3, 153.3, 152.2, 144.4, 143.4, 139.7, 137.2, 136.0, 132.9, 131.4, 129.4, 129.1, 127.9, 123.2, 122.5, 121.4, 117.6, 83.7, 78.1, 53.7, 35.3, 30.7, 26.7, 17.2. HRMS (ESI) m/z calcd for $C_{30}H_{29}BrN_3O_2$ $[M+H]^+$ = 542.1438, found = 542.1440; the ee value was 91%, t_R (minor) = 14.2 min, t_R (major) = 15.9 min (Chiralpak IC, λ = 254 nm, 30% *i*-PrOH/Hexane, flow rate = 1.0 mL/min).

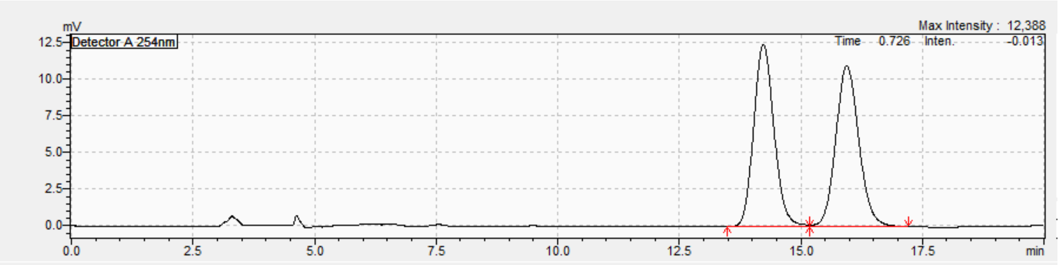

Results View - Peak Table

Peak Table | Compound | Group | Calibration Curve

Peak#	Ret. Time	Area	Height	Mark	Conc.	Unit	ID#	Name	Area%
1	14.231	364575	12460		49.899				49.899
2	15.947	366048	11008	V	50.101				50.101
Total		730623	23468		100.000				100.000

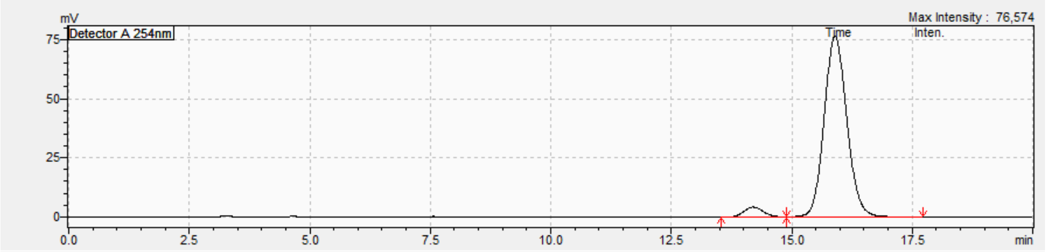

Results View - Peak Table

Peak Table | Compound | Group | Calibration Curve

Peak#	Ret. Time	Area	Height	Mark	Conc.	Unit	ID#	Name	Area%
1	14.203	119648	4220	M	4.547				4.547
2	15.898	2511845	76612	M	95.453				95.453
Total		2631494	80832		100.000				100.000

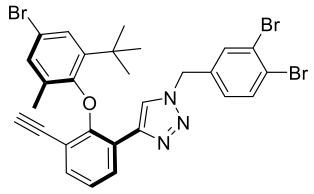

3ai

4-(2-(4-Bromo-2-(*tert*-butyl)-6-methylphenoxy)-3-ethynylphenyl)-1-(3,4-dibromobenzyl)-1*H*-1,2,3-triazole (3ai)

69% yield; $[\alpha]_D^{25}$ = -80.1 (c 0.5, $CHCl_3$), a white foam, R_f = 0.36 (hexane/ethyl acetate 8:1). ^1H NMR (400 MHz, $CDCl_3$) δ 8.40 (dd, *J* = 7.8, 1.8 Hz, 1H), 7.90 (s, 1H), 7.50 (d, *J* = 8.2 Hz, 1H), 7.44 (d, *J* = 2.1 Hz, 1H), 7.28 (dd, *J* = 7.7, 1.7 Hz, 1H), 7.23 (d, *J* = 2.5 Hz, 1H), 7.09 – 7.01 (m, 2H), 6.97 (dd, *J* = 8.2, 2.1 Hz, 1H), 5.53 (d, *J* = 15.2 Hz, 1H), 5.31 (d, *J* = 15.2 Hz, 1H), 2.68 (s, 1H), 1.74 (s, 3H), 1.20 (s, 9H). ^{13}C NMR (101 MHz, $CDCl_3$) δ 153.3, 152.2, 144.4, 136.0, 135.6, 134.3, 132.9, 132.9, 131.4, 129.4, 127.9, 127.8, 125.6, 125.4, 123.0, 122.5, 121.3, 117.6, 110.5, 83.7, 78.1, 52.8, 35.3, 30.7, 17.2. HRMS (ESI) m/z calcd for $C_{28}H_{25}Br_3N_3O$ $[M+H]^+$ = 655.9542, found = 655.9541; the ee value was > 99%, t_R (minor) = 12.4 min, t_R (major) = 17.2 min (Chiralpak IC, λ = 254 nm, 10% *i*-PrOH/Hexane, flow rate = 1.0 mL/min).

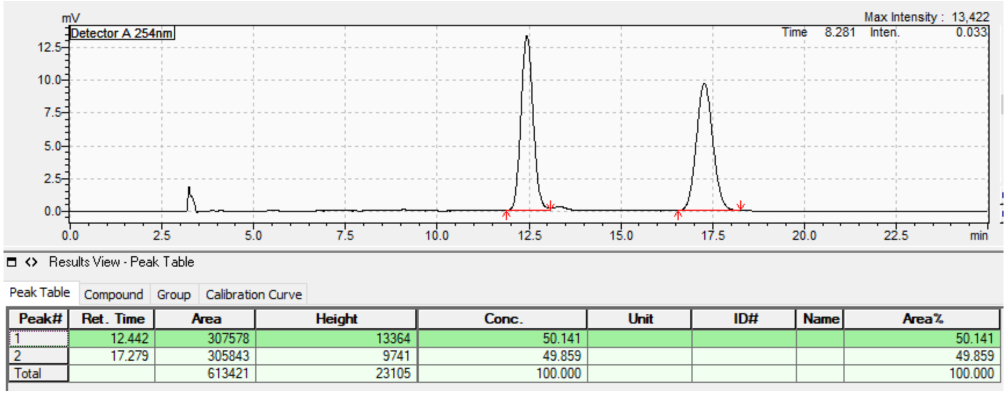

Peak#	Ret. Time	Area	Height	Conc.	Unit	ID#	Name	Area%
1	12.442	307578	13364	50.141				50.141
2	17.279	305843	9741	49.859				49.859
Total		613421	23105	100.000				100.000

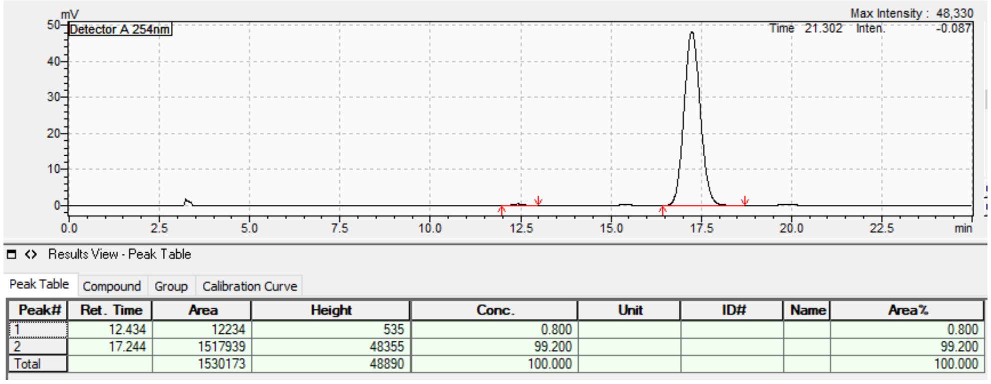

Peak#	Ret. Time	Area	Height	Conc.	Unit	ID#	Name	Area%
1	12.434	12234	535	0.800				0.800
2	17.244	1517939	48355	99.200				99.200
Total		1530173	48890	100.000				100.000

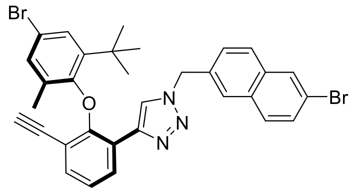

3aj

4-(2-(4-Bromo-2-(*tert*-butyl)-6-methylphenoxy)-3-ethynylphenyl)-1-((6-bromonaphthalen-2-yl)methyl)-1*H*-1,2,3-triazole (3aj)

82% yield; $[\alpha]_D^{25}$ = -60.4 (c 0.5, $CHCl_3$), a white foam, R_f = 0.31 (hexane/ethyl acetate 8:1). ^1H NMR (400 MHz, $CDCl_3$) δ 8.41 (dd, *J* = 7.8, 1.8 Hz, 1H), 7.97 – 7.86 (m, 2H), 7.68 – 7.47 (m, 4H), 7.27 (td, *J* = 8.1, 7.6, 1.8 Hz, 2H), 7.23 – 7.13 (m, 2H), 7.10 – 6.94 (m, 2H), 5.73 (d, *J* = 15.1 Hz, 1H), 5.52 (d, *J* = 15.0 Hz, 1H), 2.66 (s, 1H), 1.71 (s, 3H), 1.09 (s, 9H). ^{13}C NMR (101 MHz, $CDCl_3$) δ 153.3, 152.2, 144.4, 143.3, 135.9, 134.2, 132.8, 132.7, 131.6, 131.3, 130.1, 129.8, 129.5, 129.4, 128.2, 127.8, 127.0, 126.2, 123.2, 122.5, 121.6, 120.7, 117.5, 110.5, 83.6, 78.2, 54.2, 35.2, 30.7, 17.2. HRMS (ESI) m/z calcd for $C_{32}H_{28}Br_2N_3O$ $[M+H]^+$ = 628.0594, found = 628.0593; the ee value was 96%, t_R (minor) = 16.0 min, t_R (major) = 19.7 min (Chiralpak IC, λ = 254 nm, 10% *i*-PrOH/Hexane, flow rate = 1.0 mL/min).

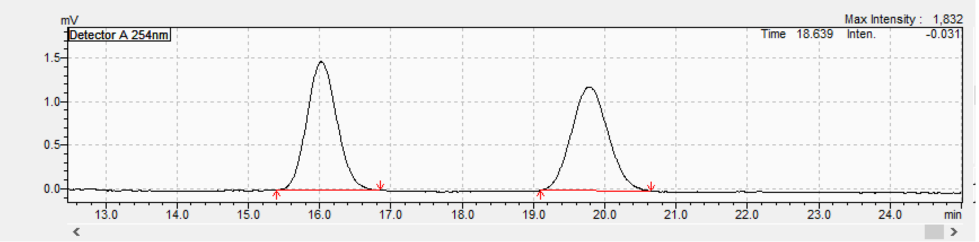

Results View - Peak Table

Peak Table | Compound | Group | Calibration Curve

Peak#	Ret. Time	Area	Height	Conc.	Unit	ID#	Name	Area%
1	16.023	43374	1478	50.487				50.487
2	19.787	42537	1191	49.513				49.513
Total		85911	2669	100.000				100.000

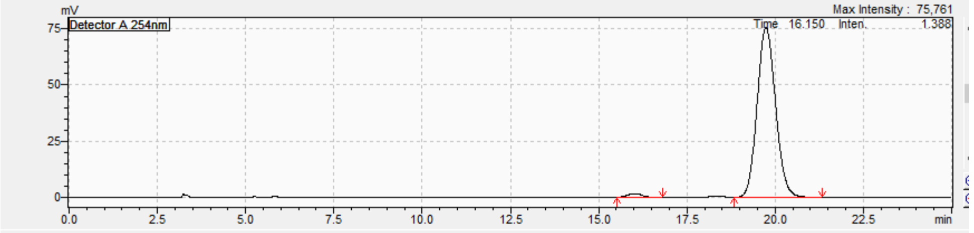

Results View - Peak Table

Peak Table | Compound | Group | Calibration Curve

Peak#	Ret. Time	Area	Height	Conc.	Unit	ID#	Name	Area%
1	16.010	51903	1763	1.850				1.850
2	19.740	2753266	75724	98.150				98.150
Total		2805168	77487	100.000				100.000

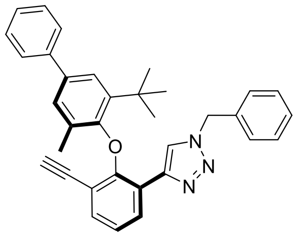

3ak

1-Benzyl-4-(2-((3-(*tert*-butyl)-5-methyl-[1,1'-biphenyl]-4-yl)oxy)-3-ethynylphenyl)-1*H*-1,2,3-triazole (3ak)

71% yield; $[\alpha]_D^{25}$ = -80.6 (c 0.5, $CHCl_3$), a white foam, R_f = 0.34 (hexane/ethyl acetate 8:1). ^1H NMR (400 MHz, $CDCl_3$) δ 8.44 (dd, *J* = 7.8, 1.8 Hz, 1H), 8.00 (s, 1H), 7.56 – 7.46 (m, 2H), 7.41 – 7.33 (m, 3H), 7.32 – 7.24 (m, 4H), 7.14 – 6.95 (m, 3H), 5.62 (d, *J* = 14.9 Hz, 1H), 5.37 (d, *J* = 15.0 Hz, 1H), 2.57 (s, 1H), 1.83 (s, 3H), 1.24 (s, 9H). ^{13}C NMR (101 MHz, $CDCl_3$) δ 152.8, 142.6, 137.6, 136.0, 134.6, 131.1, 129.3, 129.1, 128.8, 128.7, 127.9, 127.5, 127.1, 127.0, 123.7, 123.4, 122.2, 83.2, 78.3, 54.4, 35.2, 31.0, 17.6. HRMS (ESI) m/z calcd for $C_{34}H_{32}N_3O$ $[M+H]^+$ = 498.2540, found = 498.2541; the ee value was > 99%, t_R (minor) = 12.6 min, t_R (major) = 17.8 min (Chiralpak IC, λ = 254 nm, 10% *i*-PrOH/Hexane, flow rate = 1.0 mL/min).

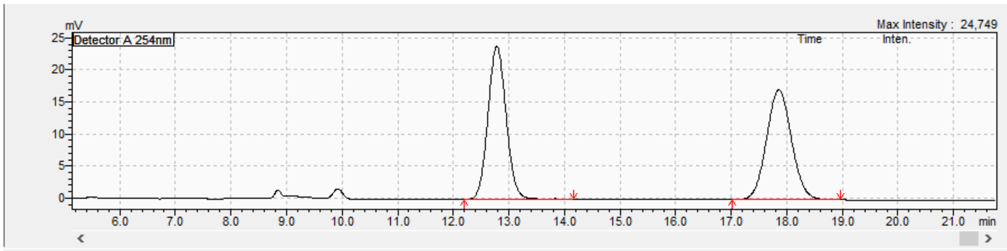

Peak#	Ret. Time	Area	Height	Mark	Conc.	Unit	ID#	Name	Area%
1	12.783	545116	23925	S	50.250				50.250
2	17.856	539689	17187		49.750				49.750
Total		1084805	41112		100.000				100.000

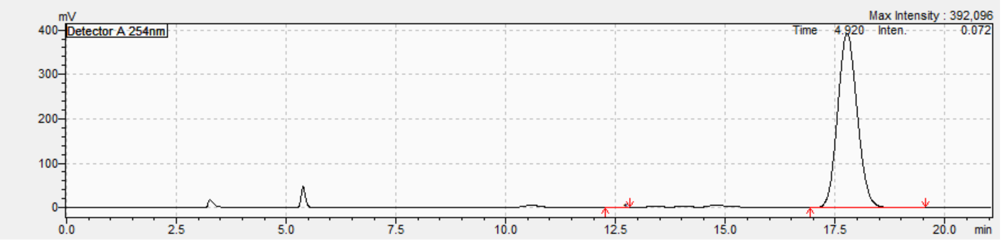

Peak#	Ret. Time	Area	Height	Mark	Conc.	Unit	ID#	Name	Area%
1	12.617	8850	293		0.073				0.073
2	17.768	12154190	392048		99.927				99.927
Total		12163040	392341		100.000				100.000

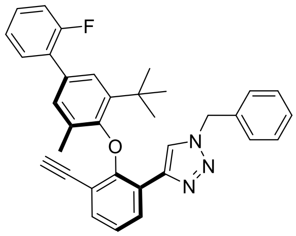

3al

1-Benzyl-4-(2-((3-(*tert*-butyl)-2'-fluoro-5-methyl-[1,1'-biphenyl]-4-yl)oxy)-3-ethynylphenyl)-1*H*-1,2,3-triazole (3al)

61% yield; $[\alpha]_D^{25}$ = -73.6 (c 0.5, $CHCl_3$), a white foam, R_f = 0.35 (hexane/ethyl acetate 8:1). ^{1}H NMR (400 MHz, $CDCl_3$) δ 8.43 (dd, *J* = 7.8, 1.8 Hz, 1H), 7.99 (s, 1H), 7.36 (dd, *J* = 7.7, 1.8 Hz, 1H), 7.33 – 7.23 (m, 5H), 7.17 – 6.83 (m, 5H), 5.61 (d, *J* = 15.0 Hz, 1H), 5.37 (d, *J* = 15.0 Hz, 1H), 2.62 (s, 1H), 1.82 (s, 3H), 1.23 (s, 9H). ^{13}C NMR (101 MHz, $CDCl_3$) δ 161.0, 153.8, 142.3, 135.8, 134.7, 132.2, 130.9, 130.7, 130.7, 129.3, 129.2, 129.1, 128.8, 128.7, 128.6, 127.9, 125.7, 124.3, 124.3, 123.3, 122.2, 121.6, 116.2, 116.0, 110.6, 83.1, 78.3, 54.2, 35.2, 31.0, 17.4. HRMS (ESI) m/z calcd for $C_{34}H_{31}FN_3O$ $[M+H]^+$ = 516.2446, found = 516.2450; the ee value was > 99%, t_R (minor) = 10.8 min, t_R (major) = 14.6 min (Chiralpak IC, λ = 254 nm, 10% *i*-PrOH/Hexane, flow rate = 1.0 mL/min).

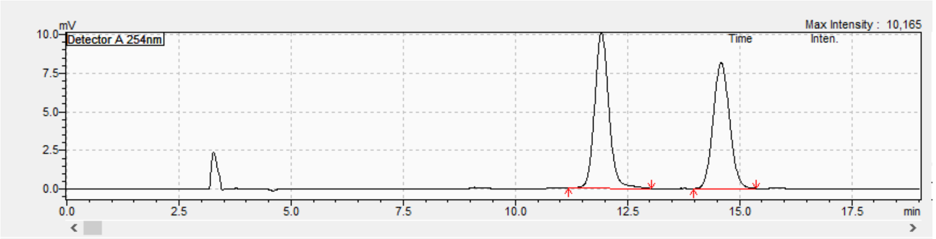

Results View - Peak Table

Peak Table | Compound | Group | Calibration Curve

Peak#	Ret. Time	Area	Height	Conc.	Unit	ID#	Name	Area%
1	11.923	222108	10109	51.756				51.756
2	14.590	207033	8149	48.244				48.244
Total		429141	18258	100.000				100.000

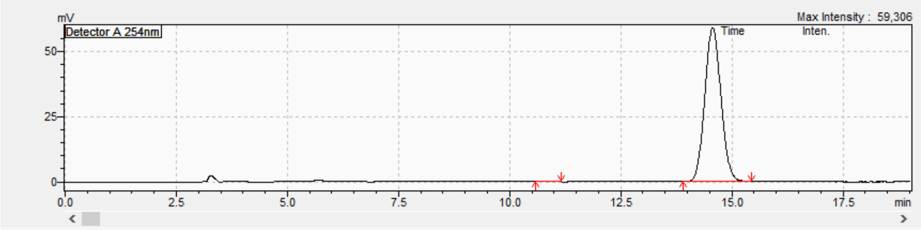

Results View - Peak Table

Peak Table | Compound | Group | Calibration Curve

Peak#	Ret. Time	Area	Height	Conc.	Unit	ID#	Name	Area%
1	10.787	1611	89	0.107				0.107
2	14.568	1508917	59267	99.893				99.893
Total		1510529	59356	100.000				100.000

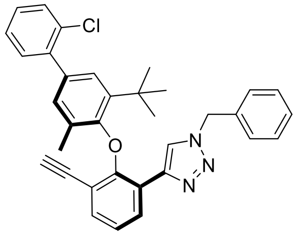

3am

1-Benzyl-4-(2-((3-(tert-butyl)-2'-chloro-5-methyl-[1,1'-biphenyl]-4-yl)oxy)-3-ethynylphenyl)-1*H*-1,2,3-triazole (3am)

79% yield; $[\alpha]_D^{25}$ = -83.7 (c 0.5, $CHCl_3$), a white foam, R_f = 0.31 (hexane/ethyl acetate 8:1). ^{1}H NMR (400 MHz, $CDCl_3$) δ 8.43 (dd, *J* = 7.8, 1.8 Hz, 1H), 8.02 (s, 1H), 7.47 – 7.39 (m, 1H), 7.31 – 7.16 (m, 10H), 7.02 (t, *J* = 7.7 Hz, 1H), 6.95 (d, *J* = 2.2 Hz, 1H), 5.62 (d, *J* = 15.0 Hz, 1H), 5.38 (d, *J* = 15.0 Hz, 1H), 2.69 (s, 1H), 1.82 (s, 3H), 1.23 (s, 9H). ^{13}C NMR (101 MHz, $CDCl_3$) δ 153.8, 152.7, 143.3, 141.9, 140.5, 135.8, 135.7, 134.8, 131.4, 130.6, 130.0, 129.6, 129.2, 129.1, 128.6, 128.4, 127.8, 126.9, 126.2, 123.3, 122.2, 121.6, 110.5, 83.4, 78.3, 54.2, 35.2, 31.0, 17.4. HRMS (ESI) m/z calcd for $C_{34}H_{31}ClN_3O$ $[M+H]^+$ = 532.2150, found = 532.2152; the ee value was 97%, t_R (minor) = 12.1 min, t_R (major) = 13.8 min (Chiralpak IC, λ = 254 nm, 10% *i*-PrOH/Hexane, flow rate = 1.0 mL/min).

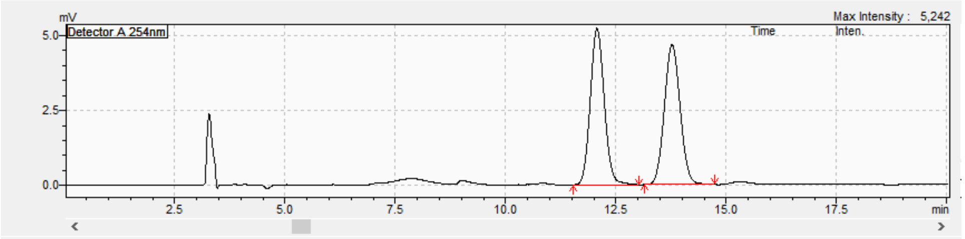

Results View - Peak Table

Peak Table | Compound | Group | Calibration Curve

Peak#	Ret. Time	Area	Height	Conc.	Unit	ID#	Name	Area%
1	12.070	114289	5220	49.761				49.761
2	13.769	115387	4664	50.239				50.239
Total		229676	9884	100.000				100.000

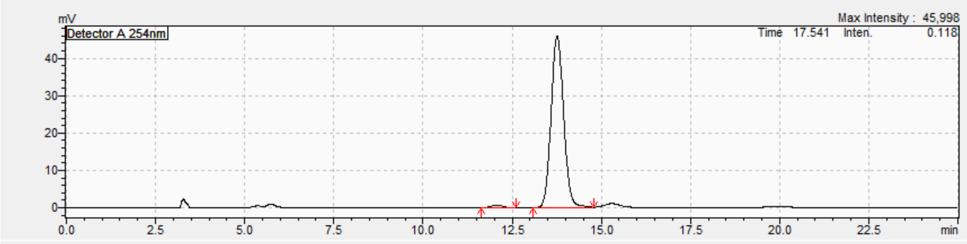

Results View - Peak Table

Peak Table | Compound | Group | Calibration Curve

Peak#	Ret. Time	Area	Height	Conc.	Unit	ID#	Name	Area%
1	12.065	15300	728	1.341				1.341
2	13.763	1125696	45943	98.659				98.659
Total		1140996	46671	100.000				100.000

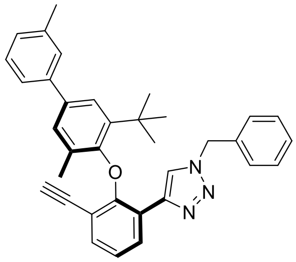

3an

1-Benzyl-4-(2-((3-(*tert*-butyl)-3',5-dimethyl-[1,1'-biphenyl]-4-yl)oxy)-3-ethynylphenyl)-1*H*-1,2,3-triazole (3an)

73% yield; $[\alpha]_D^{25}$ = -83.1 (c 0.5, $CHCl_3$), a white foam, R_f = 0.35 (hexane/ethyl acetate 8:1). ^{1}H NMR (400 MHz, $CDCl_3$) δ 8.43 (dd, *J* = 7.9, 1.8 Hz, 1H), 7.99 (s, 1H), 7.34 – 7.23 (m, 6H), 7.17 (dd, *J* = 4.8, 1.9 Hz, 1H), 7.13 – 6.99 (m, 3H), 5.61 (d, *J* = 15.0 Hz, 1H), 5.37 (d, *J* = 15.0 Hz, 1H), 2.57 (s, 1H), 2.36 (s, 3H), 1.83 (s, 3H), 1.24 (s, 9H). ^{13}C NMR (101 MHz, $CDCl_3$) δ 153.9, 142.5, 141.2, 138.4, 137.7, 135.8, 134.8, 131.0, 129.2, 129.1, 128. 7, 128.6, 127.9, 127.8, 127.5, 124.1, 123.6, 123.3, 122.2, 121.6, 83.1, 54.2, 35.2, 31.0, 21.6, 17.5. HRMS (ESI) m/z calcd for $C_{35}H_{34}N_3O$ $[M+H]^+$ = 512.2696, found = 512.2704; the ee value was > 99%, t_R (minor) = 11.5 min, t_R (major) = 16.5 min (Chiralpak IC, λ = 254 nm, 10% *i*-PrOH/Hexane, flow rate = 1.0 mL/min).

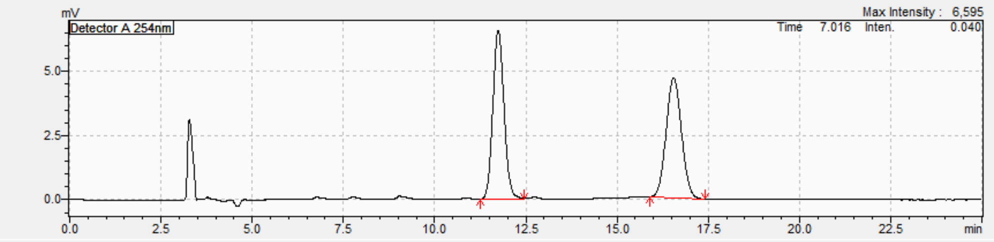

Results View - Peak Table

Peak Table | Compound | Group | Calibration Curve

Peak#	Ret. Time	Area	Height	Conc.	Unit	ID#	Name	Area%
1	11.746	140058	6578	50.563				50.563
2	16.553	136936	4674	49.437				49.437
Total		276995	11252	100.000				100.000

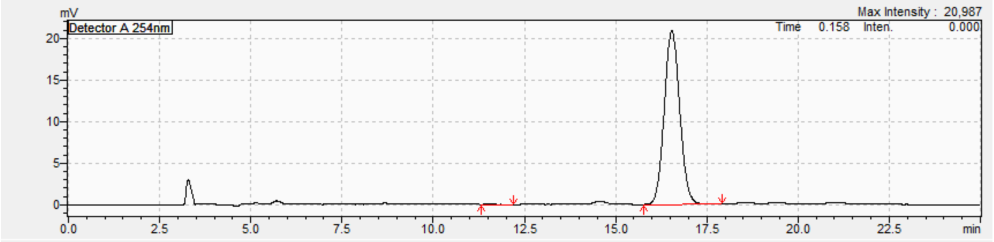

Results View - Peak Table

Peak Table | Compound | Group | Calibration Curve

Peak#	Ret. Time	Area	Height	Conc.	Unit	ID#	Name	Area%
1	11.518	162	13	0.026				0.026
2	16.542	619395	20939	99.974				99.974
Total		619556	20951	100.000				100.000

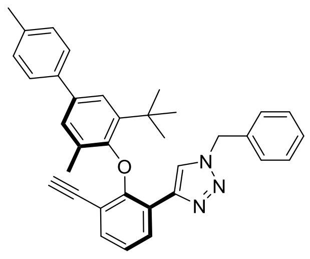

3ao

1-Benzyl-4-(2-((3-(*tert*-butyl)-4',5-dimethyl-[1,1'-biphenyl]-4-yl)oxy)-3-ethynylphenyl)-1*H*-1,2,3-triazole (3ao)

67% yield; $[\alpha]_D^{25}$ = -78.1 (c 0.5, $CHCl_3$), a white foam, R_f = 0.33 (hexane/ethyl acetate 8:1). ^1H NMR (400 MHz, $CDCl_3$) δ 8.43 (dd, *J* = 7.8, 1.8 Hz, 1H), 7.99 (s, 1H), 7.48 – 7.39 (m, 2H), 7.32 (d, *J* = 2.3 Hz, 1H), 7.26 (dd, *J* = 7.8, 1.7 Hz, 2H), 7.16 – 6.92 (m, 2H), 5.61 (d, *J* = 15.0 Hz, 1H), 5.37 (d, *J* = 15.0 Hz, 1H), 2.56 (s, 1H), 2.33 (s, 3H), 1.82 (s, 3H), 1.24 (s, 9H). ^{13}C NMR (101 MHz, $CDCl_3$) δ 153.9,152.6, 142.5, 137.5, 135.8, 134.8, 131.1, 129.5, 129.2, 129.1, 128.6, 127.9, 127.2, 126.9, 123.5, 123.3, 122.1, 121.6, 110.6, 83.1, 78.4, 54.2, 35.2, 31.0, 21.1, 17.5. HRMS (ESI) m/z calcd for $C_{35}H_{34}N_3O$ $[M+H]^+$ = 512.2696, found = 512.2701; the ee value was > 99%, t_R (minor) = 12.8 min, t_R (major) = 16.4 min (Chiralpak IC, λ = 254 nm, 10% *i*-PrOH/Hexane, flow rate = 1.0 mL/min).

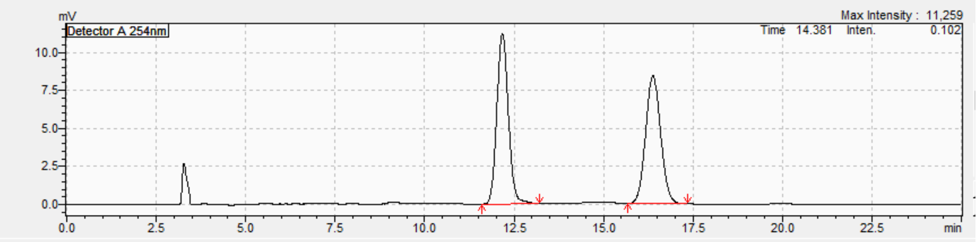

Results View - Peak Table

Peak Table | Compound | Group | Calibration Curve

Peak#	Ret. Time	Area	Height	Conc.	Unit	ID#	Name	Area%
1	12.174	250966	11218	50.401				50.401
2	16.383	246973	8399	49.599				49.599
Total		497940	19616	100.000				100.000

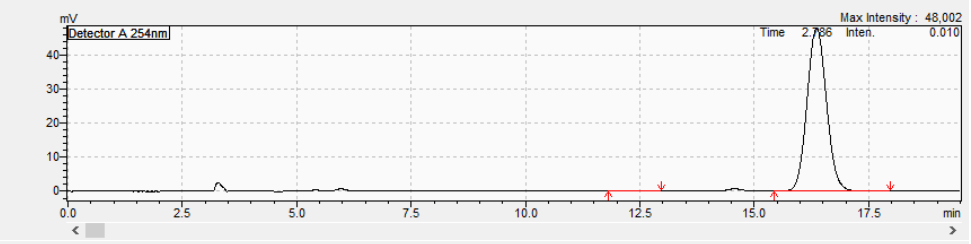

Results View - Peak Table

Peak Table | Compound | Group | Calibration Curve

Peak#	Ret. Time	Area	Height	Conc.	Unit	ID#	Name	Area%
1	12.770	436	16	0.031				0.031
2	16.360	1412679	47960	99.969				99.969
Total		1413115	47975	100.000				100.000

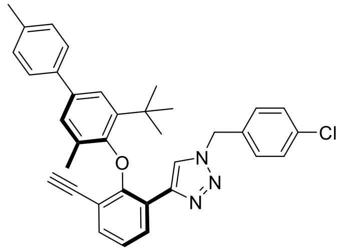

3ap

4-(2-((3-(*tert*-Butyl)-4',5-dimethyl-[1,1'-biphenyl]-4-yl)oxy)-3-ethynylphenyl)-1-(4-chlorobenzyl)-1*H*-1,2,3-triazole (3ap)

63% yield; $[\alpha]_D^{25}$ = -79.6 (c 0.5, $CHCl_3$), a white foam, R_f = 0.33 (hexane/ethyl acetate 8:1). ^{1}H NMR (400 MHz, $CDCl_3$) δ 8.42 (dd, *J* = 7.9, 1.8 Hz, 1H), 7.98 (s, 1H), 7.49 – 7.40 (m, 2H), 7.37 – 7.24 (m, 3H), 7.20 – 6.96 (m, 8H), 5.57 (d, *J* = 15.1 Hz, 1H), 5.34 (d, *J* = 15.1 Hz, 1H), 2.57 (s, 1H), 2.33 (s, 3H), 1.83 (s, 3H), 1.25 (s, 9H). ^{13}C NMR (101 MHz, $CDCl_3$) δ 153.9, 152.5, 142.5, 138.3, 136.9, 135.9, 133.3, 131.0, 129.5, 129.3, 129.2, 127.2, 126.9, 123.5, 123.2, 122.2, 110.6, 83.2, 78.3, 53.5, 35.2, 31.0, 21.1, 17.5. HRMS (ESI) m/z calcd for $C_{35}H_{33}ClN_3O$ $[M+H]^+$ = 546.2307, found = 546.2308; the ee value was > 99%, t_R (minor) = 10.9 min, t_R (major) = 15.1 min (Chiralpak IC, λ = 254 nm, 10% *i*-PrOH/Hexane, flow rate = 1.0 mL/min).

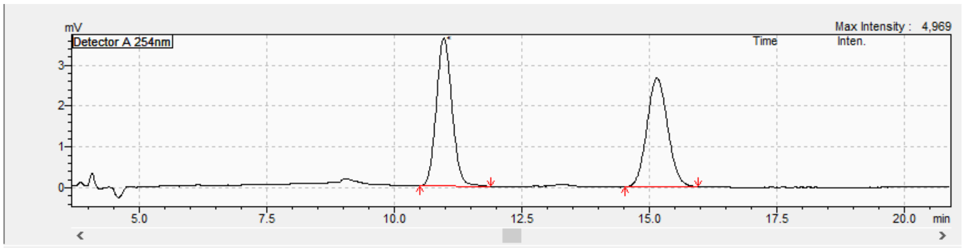

Results View - Peak Table

Peak Table | Compound | Group | Calibration Curve

Peak#	Ret. Time	Area	Height	Conc.	Unit	ID#	Name	Area%
1	10.974	77447	3613	50.646				50.646
2	15.152	75470	2657	49.354				49.354
Total		152917	6270	100.000				100.000

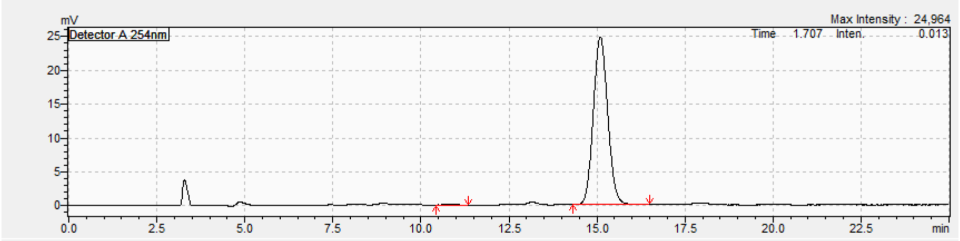

Results View - Peak Table

Peak Table | Compound | Group | Calibration Curve

Peak#	Ret. Time	Area	Height	Conc.	Unit	ID#	Name	Area%
1	10.902	721	38	0.102				0.102
2	15.097	706393	24818	99.898				99.898
Total		707114	24856	100.000				100.000

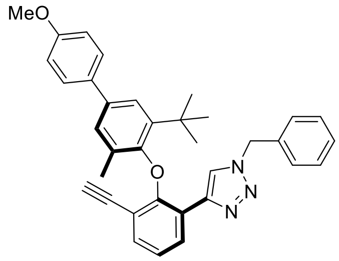

3aq

1-Benzyl-4-(2-((3-(*tert*-butyl)-4'-methoxy-5-methyl-[1,1'-biphenyl]-4-yl)oxy)-3-ethynylphenyl)-1*H*-1,2,3-triazole (3aq)

76% yield; $[\alpha]_D^{25}$ = -79.2 (c 0.5, $CHCl_3$), a white foam, R_f = 0.23 (hexane/ethyl acetate 8:1). ^{1}H NMR (400 MHz, $CDCl_3$) δ 8.43 (d, *J* = 9.6 Hz, 1H), 7.99 (s, 1H), 7.44 (d, *J* = 8.7 Hz, 2H), 7.34 – 7.24 (m, 4H), 7.18 – 7.15 (m, 1H), 7.06 (d, *J* = 2.3 Hz, 1H), 7.02 (t, *J* = 7.7 Hz, 1H), 6.90 (d, *J* = 8.8 Hz, 2H), 5.61 (d, *J* = 15.0 Hz, 1H), 5.37 (d, *J* = 15.0 Hz, 1H), 3.78 (s, 3H), 2.57 (s, 1H), 1.82 (s, 3H), 1.24 (s, 9H). ^{13}C NMR (101 MHz, $CDCl_3$) δ 159.0, 153.9, 143.4, 142.5, 137.2, 135.8, 134.8, 133.8, 131.1, 129.2, 129.1, 128.6, 128.0, 127.9, 127.0, 123.3, 123.2, 122.1, 121.6, 114.2, 110.5, 83.1, 78.4, 55.4, 54.2, 35.2, 31.0, 17.5. HRMS (ESI) m/z calcd for $C_{35}H_{34}N_3O_2$ $[M+H]^+$ = 528.2646, found = 528.2655; the ee value was 99%, t_R (minor) = 20.4 min, t_R (major) = 31.4 min (Chiralpak IC, λ = 254 nm, 10% *i*-PrOH/Hexane, flow rate = 1.0 mL/min).

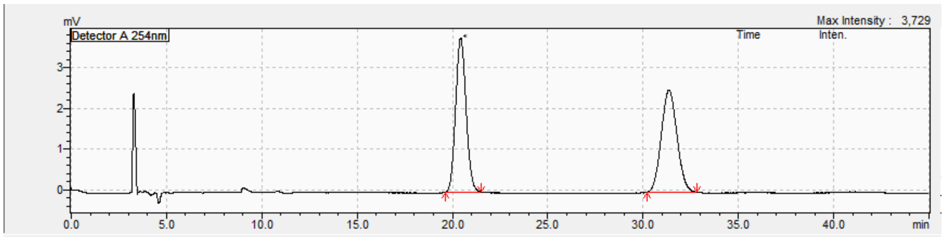

Results View - Peak Table

Peak Table | Compound | Group | Calibration Curve

Peak#	Ret. Time	Area	Height	Conc.	Unit	ID#	Name	Area%
1	20.431	144678	3783	50.135				50.135
2	31.355	143897	2505	49.865				49.865
Total		288575	6288	100.000				100.000

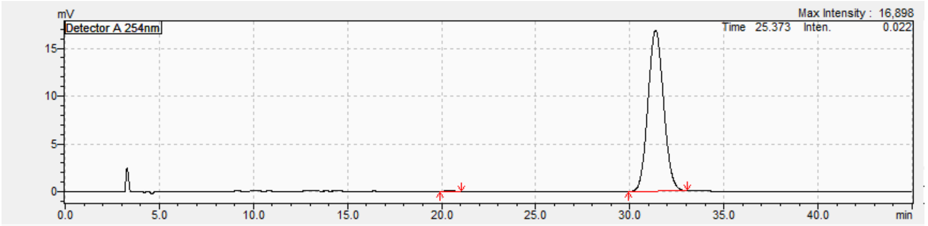

Results View - Peak Table

Peak Table | Compound | Group | Calibration Curve

Peak#	Ret. Time	Area	Height	Conc.	Unit	ID#	Name	Area%
1	20.386	3784	111	0.388				0.388
2	31.368	970823	16832	99.612				99.612
Total		974607	16943	100.000				100.000

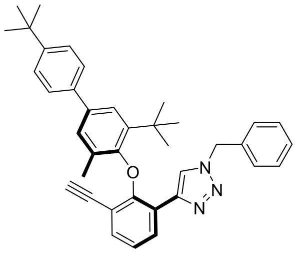

3ar

1-Benzyl-4-(2-((3,4'-di-*tert*-butyl-5-methyl-[1,1'-biphenyl]-4-yl)oxy)-3-ethynylphenyl)-1*H*-1,2,3-triazole (3ar)

64% yield; $[\alpha]_D^{25}$ = -81.9 (c 0.5, $CHCl_3$), a white foam, R_f = 0.35 (hexane/ethyl acetate 8:1). ^{1}H NMR (400 MHz, $CDCl_3$) δ 8.43 (d, *J* = 7.8 Hz, 1H), 8.00 (s, 1H), 7.46 (d, *J* = 8.4 Hz, 2H), 7.39 (d, *J* = 8.4 Hz, 2H), 7.33 (d, *J* = 2.3 Hz, 1H), 7.25 (dd, *J* = 5.2, 1.9 Hz, 4H), 7.17 (dd, *J* = 4.8, 1.8 Hz, 1H), 7.10 (d, *J* = 2.3 Hz, 1H), 7.01 (s, 1H), 5.61 (d, *J* = 15.0 Hz, 1H), 5.37 (d, *J* = 15.0 Hz, 1H), 2.55 (s, 1H), 1.82 (s, 3H), 1.30 (s, 9H), 1.24 (s, 9H). ^{13}C NMR (101 MHz, $CDCl_3$) δ 153.9, 152.6, 143.3, 142.5, 138.4, 135.9, 134.7, 131.1, 129.2, 129.1, 128.6, 127.9, 127.3, 126.7, 125.7, 123.6, 123.3, 122.2, 121.5, 110.5, 83.1, 54.2, 35.2, 34.5, 31.4, 31.0, 17.5. HRMS (ESI) m/z calcd for $C_{38}H_{40}N_3O$ $[M+H]^+$ = 554.3166, found = 554.3170; the ee value was > 99%, t_R (minor) = 9.1 min, t_R (major) = 13.4 min (Chiralpak IC, λ = 254 nm, 10% *i*-PrOH/Hexane, flow rate = 1.0 mL/min).

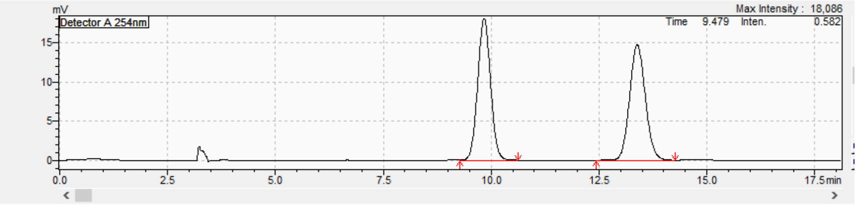

Results View - Peak Table

Peak Table | Compound | Group | Calibration Curve

Peak#	Ret. Time	Area	Height	Conc.	Unit	ID#	Name	Area%
1	9.836	375665	18083	50.028				50.028
2	13.385	375250	14732	49.972				49.972
Total		750916	32814	100.000				100.000

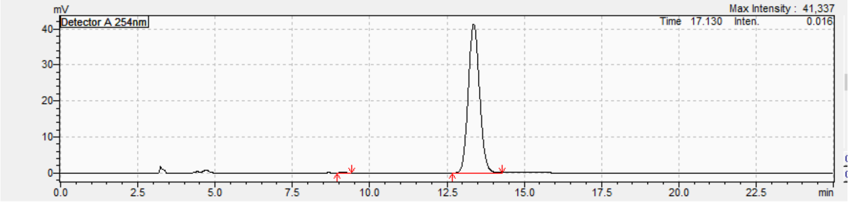

Results View - Peak Table

Peak Table | Compound | Group | Calibration Curve

Peak#	Ret. Time	Area	Height	Conc.	Unit	ID#	Name	Area%
1	9.090	1148	75	0.109				0.109
2	13.367	1050117	41330	99.891				99.891
Total		1051265	41405	100.000				100.000

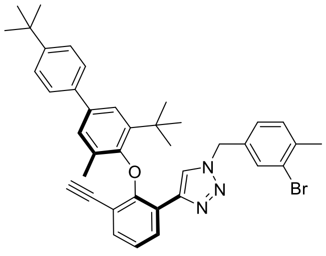

3as

1-(3-Bromo-4-methylbenzyl)-4-(2-((3,4'-di-*tert*-butyl-5-methyl-[1,1'-biphenyl]-4-yl)oxy)-3-ethynylphenyl)-1*H*-1,2,3-triazole (3as)

63% yield; $[\alpha]_D^{25}$ = -45.3 (c 0.5, $CHCl_3$), a white foam, R_f = 0.32 (hexane/ethyl acetate 8:1). ^1H NMR (400 MHz, $CDCl_3$) δ 8.43 (dd, *J* = 7.8, 1.8 Hz, 1H), 7.99 (s, 1H), 7.54 – 7.26 (m, 7H), 7.14 – 6.79 (m, 4H), 5.52 (d, *J* = 15.0 Hz, 1H), 5.29 (d, *J* = 15.0 Hz, 1H), 2.56 (s, 1H), 2.27 (s, 3H), 1.83 (s, 3H), 1.30 (s, 9H), 1.25 (s, 9H). ^{13}C NMR (101 MHz, $CDCl_3$) δ 153.9, 152.6, 150.1, 143.5, 142.5, 138.3, 135.9, 134.0, 133.0, 131.0, 130.2, 129.2, 127.3, 126.7, 126.7, 125.7, 123.6, 123.2, 122.2, 121.5, 83.2, 78.3, 53.5, 35.2, 34.5, 31.4, 31.0, 22.9, 17.5. HRMS (ESI) m/z calcd for $C_{39}H_{41}BrN_3O$ $[M+H]^+$ = 646.2428, found = 646.2433; the ee value was 99%, t_R (minor) = 9.5 min, t_R (major) = 13.8 min (Chiralpak IC, λ = 254 nm, 10% *i*-PrOH/Hexane, flow rate = 1.0 mL/min).

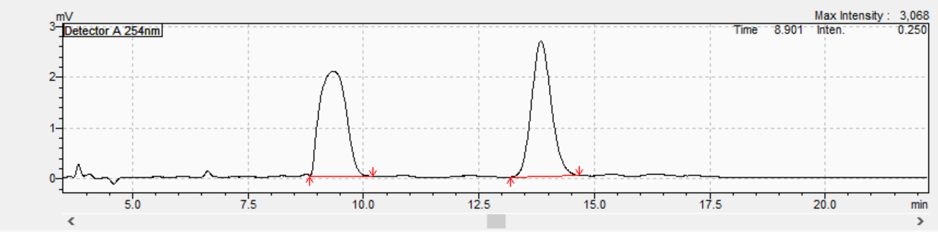

Results View - Peak Table

Peak Table | Compound | Group | Calibration Curve

Peak#	Ret. Time	Area	Height	Conc.	Unit	ID#	Name	Area%
1	9.355	81696	2065	50.882				50.882
2	13.844	78862	2661	49.118				49.118
Total		160558	4725	100.000				100.000

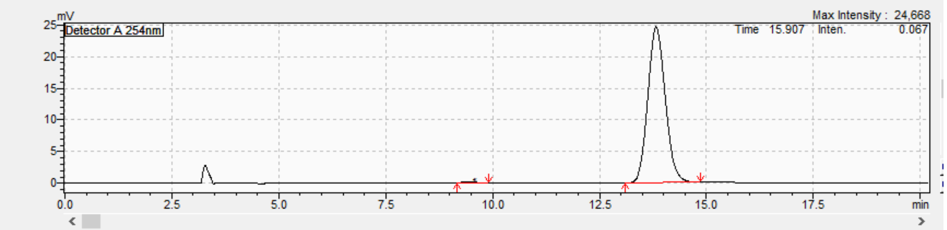

Results View - Peak Table

Peak Table | Compound | Group | Calibration Curve

Peak#	Ret. Time	Area	Height	Conc.	Unit	ID#	Name	Area%
1	9.473	3869	149	0.551				0.551
2	13.823	698082	24546	99.449				99.449
Total		701951	24695	100.000				100.000

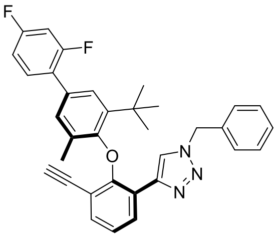

3at

1-Benzyl-4-(2-((3-(*tert*-butyl)-2',4'-difluoro-5-methyl-[1,1'-biphenyl]-4-yl)oxy)-3-ethynylphenyl)-1*H*-1,2,3-triazole (3at)

72% yield; $[\alpha]_D^{25}$ = -84.3 (c 0.5, $CHCl_3$), a white foam, R_f = 0.36 (hexane/ethyl acetate 8:1). ^{1}H NMR (400 MHz, $CDCl_3$) δ 8.44 (dd, *J* = 7.8, 1.8 Hz, 1H), 7.98 (s, 1H), 7.40 – 7.17 (m, 7H), 7.02 (dd, *J* = 14.3, 6.6 Hz, 2H), 6.93 – 6.74 (m, 2H), 5.62 (d, *J* = 14.9 Hz, 1H), 5.37 (d, *J* = 15.0 Hz, 1H), 2.61 (s, 1H), 1.82 (s, 3H), 1.23 (s, 9H). ^{13}C NMR (101 MHz, $CDCl_3$) δ 153.7, 143.3, 142.5, 135.9, 134.7, 130.9, 129.3, 129.1, 128.7, 127.9, 125.5, 123.2, 122.3, 121.6, 111.3, 110.6, 104.3, 83.1, 78.3, 54.2, 35.2, 30.9, 17.4. HRMS (ESI) m/z calcd for $C_{34}H_{30}F_2N_3O$ $[M+H]^+$ = 534.2351, found = 534.235; the ee value was 99%, t_R (minor) = 12.4 min, t_R (major) = 15.9 min (Chiralpak IC, λ = 254 nm, 10% *i*-PrOH/Hexane, flow rate = 1.0 mL/min).

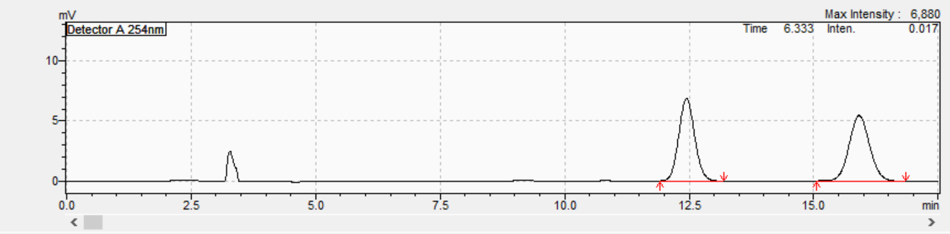

Results View - Peak Table

Peak Table | Compound | Group | Calibration Curve

Peak#	Ret. Time	Area	Height	Conc.	Unit	ID#	Name	Area%
1	12.447	152744	6851	49.335				49.335
2	15.914	156863	5448	50.665				50.665
Total		309606	12299	100.000				100.000

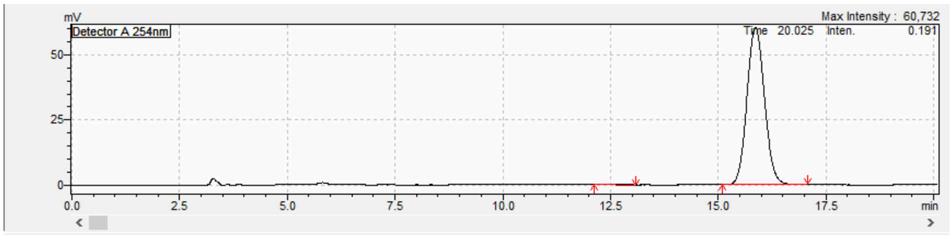

Results View - Peak Table

Peak Table | Compound | Group | Calibration Curve

Peak#	Ret. Time	Area	Height	Conc.	Unit	ID#	Name	Area%
1	12.444	5375	207	0.313				0.313
2	15.865	1711714	60671	99.687				99.687
Total		1717089	60878	100.000				100.000

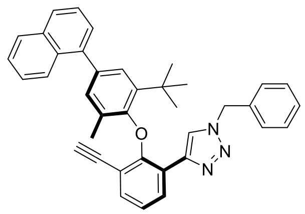

3au

1-Benzyl-4-(2-(2-(*tert*-butyl)-6-methyl-4-(naphthalen-1-yl)phenoxy)-3-ethynylphenyl)-1*H*-1,2,3-triazole (3au)

71% yield; $[\alpha]_D^{25}$ = -94.2 (c 0.5, $CHCl_3$), a white foam, R_f = 0.31 (hexane/ethyl acetate 8:1). ^{1}H NMR (400 MHz, $CDCl_3$) δ 8.46 (dd, *J* = 7.8, 1.8 Hz, 1H), 8.06 (s, 1H), 7.91 (d, *J* = 8.3 Hz, 1H), 7.84 (dd, *J* = 7.9, 1.6 Hz, 1H), 7.78 (d, *J* = 8.2 Hz, 1H), 7.53 – 7.21 (m, 9H), 7.09 – 6.98 (m, 2H), 5.64 (d, *J* = 15.0 Hz, 1H), 5.40 (d, *J* = 15.0 Hz, 1H), 2.75 (s, 1H), 1.85 (s, 3H), 1.25 (s, 9H). ^{13}C NMR (101 MHz, $CDCl_3$) δ 153.9, 152.6, 143.4, 140.2, 137.0, 136.1, 134.8, 133.9, 131.7, 130.5, 130.4, 129.4, 129.1, 128.6, 128.4, 127.8, 127.5, 126.9, 126.7, 126.0, 125.8, 125.8, 125.4, 123.3, 122.2, 121.7, 83.1, 78.8, 54.2, 35.2, 31.1, 17.4. HRMS (ESI) m/z calcd for $C_{38}H_{34}N_3O$ $[M+H]^+$ = 548.2696, found = 548.2700; the ee value was > 99%, t_R (minor) = 12.8 min, t_R (major) = 14.2 min (Chiralpak IC, λ = 254 nm, 10% *i*-PrOH/Hexane, flow rate = 1.0 mL/min).

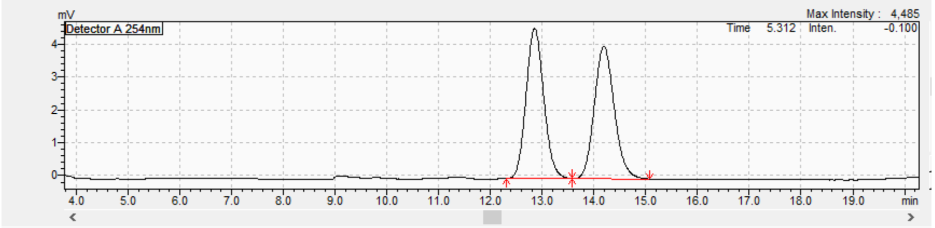

Results View - Peak Table

Peak Table | Compound | Group | Calibration Curve

Peak#	Ret. Time	Area	Height	Conc.	Unit	ID#	Name	Area%
1	12.855	109350	4579	50.157				50.157
2	14.189	108666	4042	49.843				49.843
Total		218016	8622	100.000				100.000

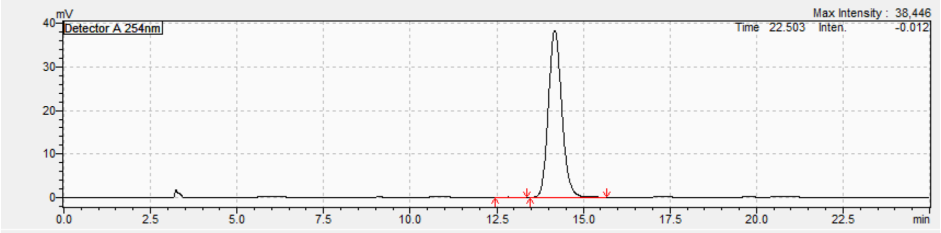

Results View - Peak Table

Peak Table | Compound | Group | Calibration Curve

Peak#	Ret. Time	Area	Height	Conc.	Unit	ID#	Name	Area%
1	12.835	1612	72	0.155				0.155
2	14.177	1036933	38429	99.845				99.845
Total		1038545	38500	100.000				100.000

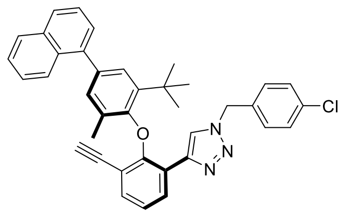

3at

4-(2-(2-(*tert*-Butyl)-6-methyl-4-(naphthalen-1-yl)phenoxy)-3-ethynylphenyl)-1-(4-chlorobenzyl)-1*H*-1,2,3-triazole (3av)

74% yield; $[\alpha]_D^{25}$ = -87.1 (c 0.5, $CHCl_3$), a white foam, R_f = 0.33 (hexane/ethyl acetate 8:1). ^{1}H NMR (400 MHz, $CDCl_3$) δ 8.45 (dd, *J* = 7.8, 1.8 Hz, 1H), 8.05 (s, 1H), 7.91 (d, *J* = 8.2 Hz, 1H), 7.88 – 7.82 (m, 1H), 7.79 (d, *J* = 8.2 Hz, 1H), 7.52 – 7.30 (m, 5H), 7.29 – 7.23 (m, 2H), 7.17 – 7.10 (m, 2H), 7.10 – 6.95 (m, 2H), 5.60 (d, *J* = 15.2 Hz, 1H), 5.38 (d, *J* = 15.1 Hz, 1H), 2.75 (s, 1H), 1.85 (s, 3H), 1.26 (s, 9H). ^{13}C NMR (101 MHz, $CDCl_3$) δ 153.9, 152.5, 143.5, 136.2, 133.9, 133.4, 130.5, 130.4, 129.3, 129.3, 129.2, 128.4, 127.6, 126.9, 126.7, 126.1, 125.8, 125.4, 123.2, 122.3, 83.2, 78.7, 53.5, 35.3, 31.0, 17.5. HRMS (ESI) m/z calcd for $C_{38}H_{33}ClN_3O$ $[M+H]^+$ = 582.2307, found = 582.2310; the ee value was 95%, t_R (minor) = 11.7 min, t_R (major) = 12.8 min (Chiralpak IC, λ = 254 nm, 10% *i*-PrOH/Hexane, flow rate = 1.0 mL/min).

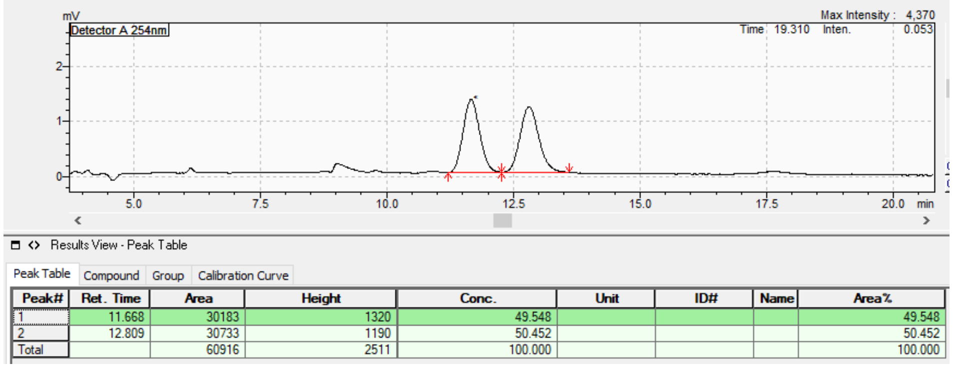

Peak#	Ret. Time	Area	Height	Conc.	Unit	ID#	Name	Area%
1	11.668	30183	1320	49.548				49.548
2	12.809	30733	1190	50.452				50.452
Total		60916	2511	100.000				100.000

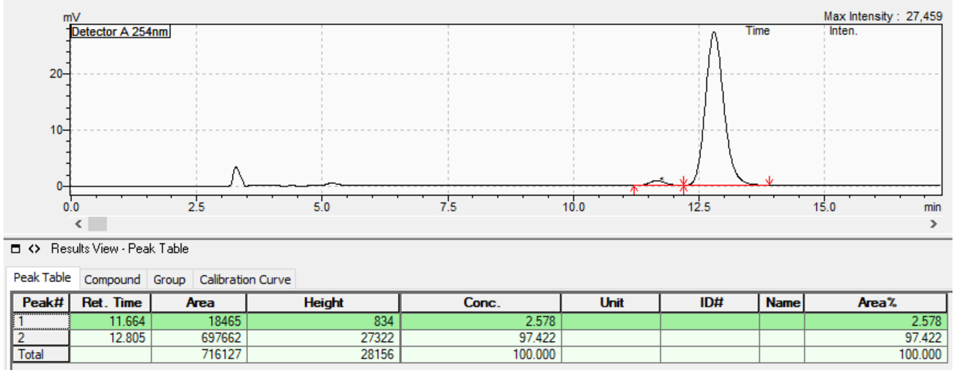

Peak#	Ret. Time	Area	Height	Conc.	Unit	ID#	Name	Area%
1	11.664	18465	834	2.578				2.578
2	12.805	697662	27322	97.422				97.422
Total		716127	28156	100.000				100.000

6. Synthetic Applications

(1) Modification of biologically active compounds

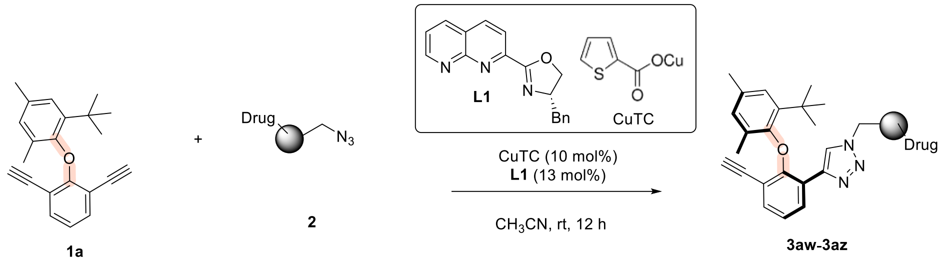

To a dried and argon-filled 10 mL Schlenk tube equipped with a magnetic stir bar was added CuTC (10 mol%) and **L1** (13 mol%) in CH_3CN (1.5 ml). The reaction mixture was stirred under at room temperature for 1 h. Then the solution of **1a** (0.05 mmol, 1.0 equiv.) and **2** (0.09 mmol, 1.8 equiv.) in CH_3CN (0.5 mL) was added. The reaction mixture was stirred at room temperature for 12 h. Then the reaction mixture was concentrated under reduced pressure and the residue was purified by column chromatography on silica gel to furnish the product.

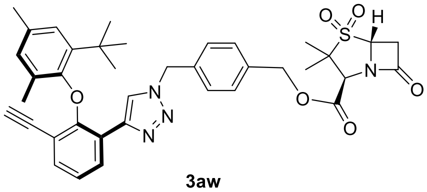

3aw

4-((4-(2-(2-(*tert*-Butyl)-4,6-dimethylphenoxy)-3-ethynylphenyl)-1*H*-1,2,3-triazol-1-yl)methyl)benzyl (2*S*,5*R*)-3,3-dimethyl-7-oxo-4-thia-1-azabicyclo[3.2.0]heptane-2-carboxylate 4,4-dioxide (3aw)

61% yield; $[\alpha]_D^{25}$ = -17.3 (c 1.0, $CHCl_3$), dr > 20 : 1, a white foam, R_f = 0.33 (hexane/ethyl acetate 1:1). ^{1}H NMR (400 MHz, $CDCl_3$) δ 8.41 (dd, *J* = 7.8, 1.8 Hz, 1H), 8.00 (s, 1H), 7.31 – 7.23 (m, 3H), 7.20 – 7.14 (m, 3H), 7.00 (t, *J* = 7.7 Hz, 1H), 6.91 (d, *J* = 2.2 Hz, 1H), 6.70 (dt, *J* = 2.3, 0.8 Hz, 1H), 5.58 (d, *J* = 15.2 Hz, 1H), 5.39 (d, *J* = 15.2 Hz, 1H), 5.16 (d, *J* = 12.2 Hz, 1H), 5.07 (d, *J* = 12.2 Hz, 1H), 4.52 (dd, *J* = 4.1, 2.3 Hz, 1H), 4.33 (s, 1H), 3.39 (dd, *J* = 6.2, 3.2 Hz, 2H), 2.58 (s, 1H), 2.24 (s, 3H), 1.73 (s, 3H), 1.47 (s, 3H), 1.21 (d, *J* = 3.9 Hz, 14H). ^{13}C NMR (101 MHz, $CDCl_3$) δ 170.7, 166.8, 141.9, 135.9, 135.8, 134.7, 134.0, 130.4, 129.3, 129.2, 129.1, 128.1, 125.3, 122.0, 110.5, 82.9, 78.4, 77.3, 67.5, 63.2, 62.7, 61.18, 53.6, 38.4, 35.0, 31.1, 21.1, 20.2, 18.6, 17.2. HRMS (ESI) m/z calcd for $C_{38}H_{41}N_4O_6S$ $[M+H]^+$ = 681.2741, found = 681.2740.

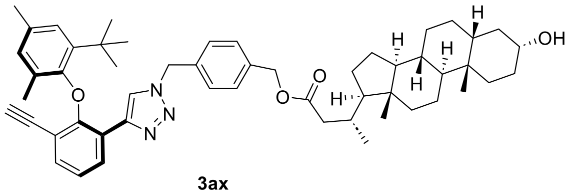

3ax

4-((4-(2-(2-(*tert*-Butyl)-4,6-dimethylphenoxy)-3-ethynylphenyl)-1*H*-1,2,3-triazol-1-yl)methyl)benzyl (*R*)-3-((3*R*,5*R*,8*R*,9*S*,10*S*,13*R*,14*S*,17*R*)-3-hydroxy-10,13-dimethylhexadecahydro-1H-cyclopenta[*a*]phenanthren-17-yl)butanoate (3ax)

75% yield; $[\alpha]_D^{25}$ = -30.6 (c 0.5, $CHCl_3$), dr > 20 : 1, a white foam, R_f = 0.32 (hexane/ethyl acetate 4:1). ^{1}H NMR (400 MHz, $CDCl_3$) δ 8.40 (dd, *J* = 7.8, 1.8 Hz, 1H), 7.96 (s, 1H), 7.33 – 7.14 (m, 6H), 6.99 (t, *J* = 7.7 Hz, 1H), 6.90 (d, *J* = 2.2 Hz, 1H), 6.69 (d, *J* = 2.2 Hz, 1H), 5.58 (d, *J* = 15.0 Hz, 1H), 5.35 (d, *J* = 15.0 Hz, 1H), 4.99 (s, 2H), 3.55 (tt, *J* = 11.0, 4.6 Hz, 1H), 2.58 (s, 1H), 2.38 – 2.13 (m, 5H), 1.98 – 1.39 (m, 19H), 1.39 – 0.78 (m, 45H), 0.55 (s, 3H). ^{13}C NMR (101 MHz, $CDCl_3$) δ 174.0, 154.1, 151.0, 143.4, 141.9, 136.7, 135.9, 134.7, 133.9, 130.5, 129.2, 129.1, 128.8, 128.0, 125.3, 123.2, 121.9, 121.4, 110.4, 82.8, 71.8, 65.4, 56.5, 55.9, 53.8, 42.7, 42.1, 40.4, 40.1, 36.4, 35.8, 35.3, 35.3, 34.9, 34.5, 31.2, 31.0, 30.9, 30.5, 28.2, 27.2, 26.4, 24.2, 23.3, 21.1, 20.8, 18.2, 17.2, 12.1. HRMS (ESI) m/z calcd for $C_{53}H_{681}N_3O_4$ $[M+H]^+$ = 810.5204, found = 810.5209.

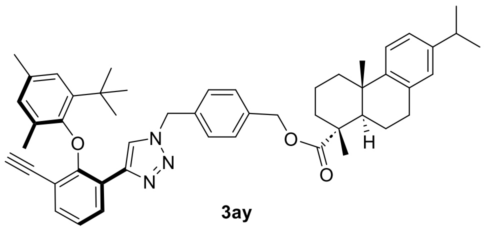

3ay

4-((4-(2-(2-(tert-butyl)-4,6-dimethylphenoxy)-3-ethynylphenyl)-1H-1,2,3-triazol-1-yl)methyl)benzyl (1R,4aS,10aR)-7-isopropyl-1,4a-dimethyl-1,2,3,4,4a,9,10,10a-octahydrophenanthrene-1-carboxylate (3ay)

72% yield; $[\alpha]_D^{25}$ = -26.5 (c 0.2, $CHCl_3$), dr > 20 : 1, a white foam, R_f = 0.32 (hexane/ethyl acetate 6:1). ^{1}H NMR (400 MHz, $CDCl_3$) δ 8.41 (dd, *J* = 7.8, 1.8 Hz, 1H), 7.97 (s, 1H), 7.25 (dd, *J* = 7.7, 1.8 Hz, 1H), 7.20 (d, *J* = 8.2 Hz, 2H), 7.14 (d, *J* = 8.2 Hz, 2H), 7.08 (d, *J* = 8.1 Hz, 1H), 6.99 (t, *J* = 7.7 Hz, 1H), 6.95 – 6.86 (m, 2H), 6.78 (d, *J* = 2.0 Hz, 1H), 6.68 (d, *J* = 2.2 Hz, 1H), 5.57 (d, *J* = 15.1 Hz, 1H), 5.35 (d, *J* = 15.1 Hz, 1H), 4.99 (q, *J* = 12.6 Hz, 2H), 2.71 (ddd, *J* = 12.5, 10.4, 6.3 Hz, 3H), 2.57 (s, 1H), 2.27 – 2.11 (m, 5H), 1.77 – 1.49 (m, 11H), 1.24 – 1.08 (m, 24H), 0.84 – 0.73 (m, 2H). ^{13}C NMR (101 MHz, $CDCl_3$) δ 178.2, 146.8, 145.8, 141.9, 136.9, 135.9, 134.6, 134.6,

133.9, 130.5, 129.2, 129.1, 128.6, 127.9, 126.9, 125.3, 124.1, 123.9, 123.2, 121.9, 110.5, 82.8, 65.7, 53.8, 47.7, 44.8, 37.9, 37.0, 36.6, 34.9, 33.4, 31.0, 30.0, 25.3, 24.0, 23.9, 21.7, 21.1, 18.6, 17.2, 16.6. HRMS (ESI) m/z calcd for $C_{50}H_{58}N_3O_3$ $[M+H]^+$ = 748.4473, found = 748.4470.

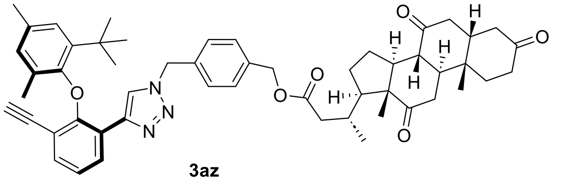

3az

4-((4-(2-(2-(*tert*-Butyl)-4,6-dimethylphenoxy)-3-ethynylphenyl)-1H-1,2,3-triazol-1-yl)methyl)benzyl (R)-3-((5S,8R,9S,10S,13R,14S,17R)-10,13-dimethyl-3,7,12-trioxohexadecahydro-1H-cyclopenta[a]phenanthren-17-yl)butanoate (3az)

79% yield; $[\alpha]_D^{25}$ = -22.6 (c 1.0, $CHCl_3$), dr > 20 : 1, a white foam, R_f = 0.33 (hexane/ethyl acetate 3:1). ^{1}H NMR (400 MHz, $CDCl_3$) δ 8.49 (dd, *J* = 7.8, 1.8 Hz, 1H), 8.07 (s, 1H), 7.33 (dd, *J* = 8.5, 6.9 Hz, 3H), 7.24 (d, *J* = 8.0 Hz, 2H), 7.08 (t, *J* = 7.7 Hz, 1H), 6.99 (d, *J* = 2.1 Hz, 1H), 6.78 (d, *J* = 2.1 Hz, 1H), 5.66 (d, *J* = 15.1 Hz, 1H), 5.45 (d, *J* = 15.1 Hz, 1H), 5.19 – 5.01 (m, 2H), 3.07 – 2.80 (m, 3H), 2.67 (s, 1H), 2.52 – 1.49 (m, 31H), 1.38 – 0.84 (m, 49H). ^{13}C NMR (101 MHz, $CDCl_3$) δ 212.0, 209.1, 208.8, 173.7, 136.6, 135.9, 134.8, 134.0, 130.4, 129.2, 129.1, 128.9, 127.9, 125.3, 121.9, 82.9, 77.2, 65.4, 56.9, 53.8, 51.7, 48.9, 46.8, 45.6, 45.5, 45.0, 42.8, 38.6, 36.5, 36.0, 35.4, 35.3, 34.9, 31.6, 31.4, 31.0, 30.3, 29.0, 27.6, 25.2, 25.1, 22.7, 21.9, 21.1, 18.6, 17.2, 14.1, 11.8, 11.4. HRMS (ESI) m/z calcd for $C_{53}H_{62}N_3O_6$ $[M+H]^+$ = 836.4633, found = 836.4639.

(2) Synthesis of bis-triazoles

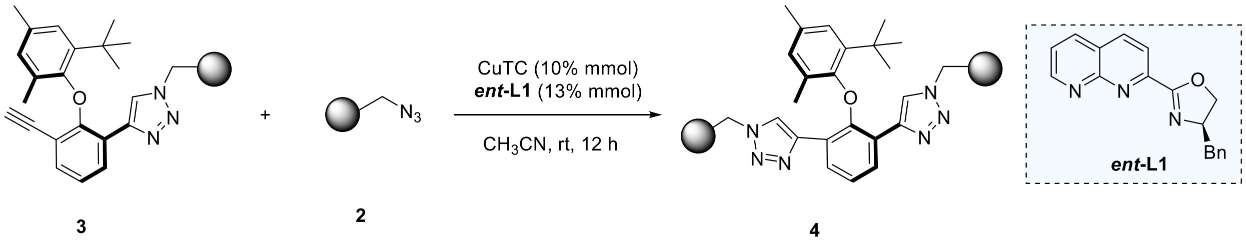

To a dried and argon-filled 10 mL Schlenk tube equipped with a magnetic stir bar was added CuTC (10 mol%) and *ent*-**L1** (13 mol%) in CH_3CN (1.5 ml). The reaction mixture was stirred under at room temperature for 1 h. Then the solution of **3** (0.05 mmol, 1.0 equiv.) and **2** (0.09 mmol, 1.8 equiv.) in CH_3CN (0.5 mL) was added. The reaction mixture was stirred at room temperature for 12 h. Then the reaction mixture was concentrated under reduced pressure and the residue was purified by column chromatography on silica gel to furnish the product.

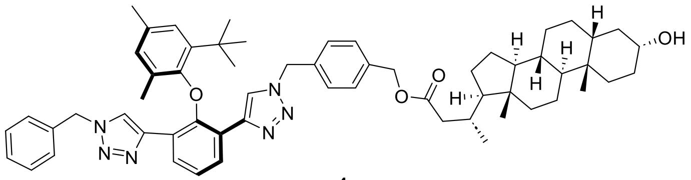

4a

4-((4-(3-(1-Benzyl-1H-1,2,3-triazol-4-yl)-2-(2-(*tert*-butyl)-4,6-dimethylphenoxy)phenyl)-1H-1,2,3-triazol-1-yl)methyl)benzyl *(R)-3-((3R,5R,8R,9S,10S,13R,14S,17R)*-3-hydroxy-10,13-dimethylhexadecahydro-1H-cyclopenta[a]phenanthren-17-yl)butanoate (4a)

92% yield; $[\alpha]_D^{25}$ = +5.4 (c 0.5, $CHCl_3$), dr > 20 : 1, a white foam, R_f = 0.31 (hexane/ethyl acetate 2:1). ^{1}H NMR (400 MHz, $CDCl_3$) δ 7.85 (t, *J* = 6.9 Hz, 1H), 7.34 – 7.23 (m, 2H), 7.19 – 7.06 (m, 2H), 6.82 (d, *J* = 13.7 Hz, 1H), 6.61 (d, *J* = 3.2 Hz, 1H), 5.36 (dd, *J* = 14.9, 1.8 Hz, 1H), 5.17 (dd, *J* = 14.9, 1.9 Hz, 1H), 5.01 (s, 1H), 3.56 (tt, *J* = 10.6, 4.6 Hz, 1H), 2.38 – 2.13 (m, 3H), 1.96 – 1.38 (m, 10H), 1.38 – 0.92 (m, 12H), 0.91 – 0.71 (m, 10H), 0.55 (s, 2H). ^{13}C NMR (101 MHz, $CDCl_3$) δ 174.0, 136.8, 134.5, 132.4, 131.6, 131.5, 130.8, 129.0, 128.8, 128.7, 128.3, 128.1, 128.1, 126.3, 123.4, 122.6, 71.9, 65.5, 56.5, 55.9, 54.0, 53.6, 42.8, 42.1, 40.4, 40.2, 36.5, 35.9, 35.4, 34.8, 34.6, 31.2, 30.9, 30.6, 30.4, 28.2, 27.2, 26.4, 24.2, 23.4, 21.0, 20.8, 18.3, 18.0, 12.1. HRMS (ESI) m/z calcd for $C_{60}H_{75}N_6O_4$ $[M+H]^+$ = 943.5844, found = 943.5847.

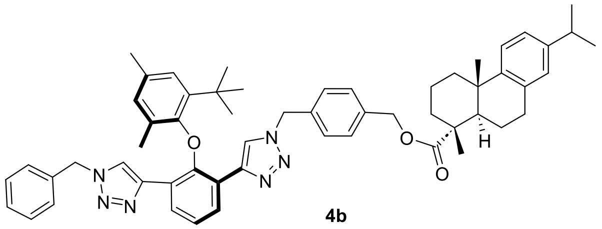

4b

4-((4-(3-(1-Benzyl-1H-1,2,3-triazol-4-yl)-2-(2-(*tert*-butyl)-4,6-dimethylphenoxy)phenyl)-1H-1,2,3-triazol-1-yl)methyl)benzyl (*1R,4aS,10aR*)-7-isopropyl-1,4a-dimethyl-1,2,3,4,4a,9,10,10a-octahydrophenanthrene-1-carboxylate (4b)

88% yield; $[\alpha]_D^{25}$ = +7.2 (c 0.5, $CHCl_3$), dr > 20 : 1, a white foam, R_f = 0.34 (hexane/ethyl acetate 2:1). ^{1}H NMR (400 MHz, $CDCl_3$) δ 7.93 – 7.83 (m, 1H), 7.35 – 7.20 (m, 3H), 7.16 – 7.02 (m, 3H), 6.92 (dd, *J* = 8.2, 2.0 Hz, 1H), 6.86 – 6.54 (m, 3H), 5.35 (dd, *J* = 14.8, 7.5 Hz, 1H), 5.17 (dd, *J* = 14.8, 2.1 Hz, 1H), 5.00 (q, *J* = 12.6 Hz, 1H), 2.83 – 2.63 (m, 2H), 2.28 – 2.14 (m, 3H), 1.80 – 1.55 (m, 5H), 1.47 – 1.26 (m, 2H), 1.23 – 1.11 (m, 8H), 0.82 (s, 6H). ^{13}C NMR (101 MHz, $CDCl_3$) δ 178.3, 152.8, 140.5, 134.5, 134.3, 132.4, 131.5, 131.4, 130.7, 129.0, 128.7, 128.5, 128.2, 128.1, 126.9, 126.2, 124.2, 124.0, 123.3, 122.9, 65.7, 53.9, 53.6, 47.7, 44.8, 37.9, 36.9, 36.6, 34.8, 33.5,

30.4, 30.0, 25.3, 24.0, 23.9, 21.7, 21.0, 18.6, 18.0, 16.6. HRMS (ESI) m/z calcd for $C_{57}H_{65}N_6O_3$ $[M+H]^+$ = 881.5113, found = 881.5112.

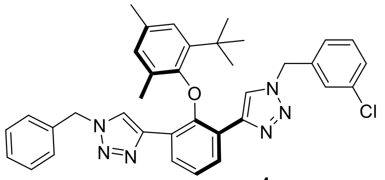

4c

1-Benzyl-4-(2-(2-(*tert*-butyl)-4,6-dimethylphenoxy)-3-(1-(3-chlorobenzyl)-1*H*-1,2,3-triazol-4-yl)phenyl)-1*H*-1,2,3-triazole (4c)

84% yield; $[\alpha]_D^{25}$ = +5.0 (c 0.5, $CHCl_3$), a white foam, R_f = 0.30 (hexane/ethyl acetate 2:1). ^1H NMR (400 MHz, $CDCl_3$) δ 7.86 (dq, *J* = 7.7, 1.8 Hz, 2H), 7.32 – 7.22 (m, 4H), 7.17 – 7.09 (m, 3H), 7.02 (dd, *J* = 7.4, 1.6 Hz, 1H), 6.82 (d, *J* = 1.1 Hz, 2H), 6.68 – 6.57 (m, 2H), 5.35 (dd, *J* = 14.9, 6.8 Hz, 2H), 5.14 (dd, *J* = 16.2, 14.8 Hz, 2H), 2.22 (s, 3H), 1.68 (s, 4H), 0.83 (s, 9H). ^{13}C NMR (101 MHz, $CDCl_3$) δ 152.8, 151.0, 140.3, 136.5, 134.9, 134.5, 132.5, 131.6, 131.4, 130.8, 130.3, 129.0, 128.9, 128.7, 128.2, 128.1, 128.0, 126.3, 126.2, 123.4, 123.0, 122.7, 54.0, 53.2, 34.8, 30.4, 21.0, 18.0. HRMS (ESI) m/z calcd for $C_{36}H_{36}ClN_6O$ $[M+H]^+$ = 881.5113, found = 881.5110; the ee value was 91%, t_R (minor) = 14.5 min, t_R (major) = 24.4 min (Chiralpak IC, λ = 254 nm, 10% *i*-PrOH/Hexane, flow rate = 1.0 mL/min).

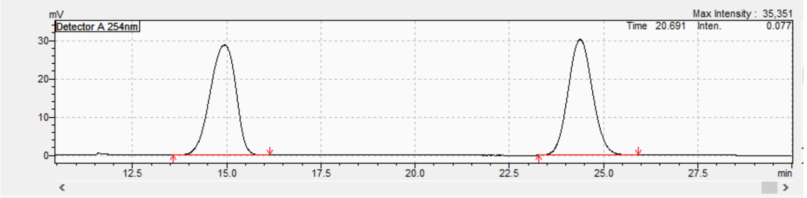

Results View - Peak Table

Peak Table | Compound | Group | Calibration Curve

Peak#	Ret. Time	Area	Height	Conc.	Unit	ID#	Name	Area%
1	14.944	1367008	28772	50.145				50.145
2	24.379	1359116	30280	49.855				49.855
Total		2726124	59053	100.000				100.000

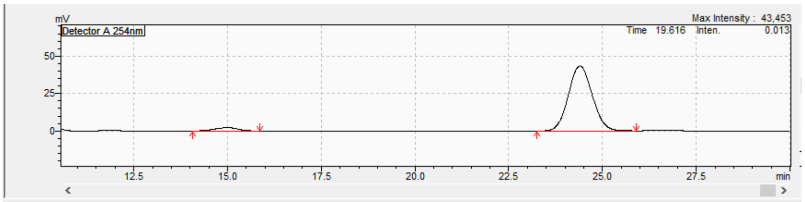

Results View - Peak Table

Peak Table | Compound | Group | Calibration Curve

Peak#	Ret. Time	Area	Height	Conc.	Unit	ID#	Name	Area%
1	14.995	89677	2108	4.461				4.461
2	24.398	1920372	43451	95.539				95.539
Total		2010050	45559	100.000				100.000

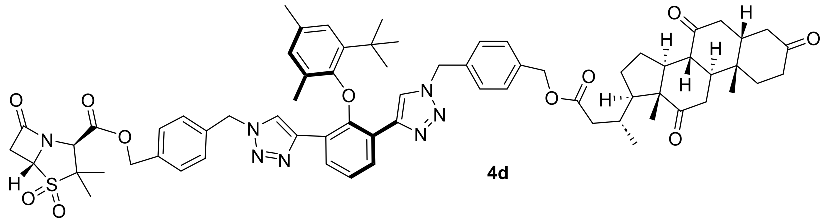

4d

4-((4-(2-(2-(*tert*-Butyl)-4,6-dimethylphenoxy)-3-(1-(4-((((*R*)-3-((5*S*,8*R*,9*S*,10*S*,13*R*,14*S*,17*R*)-10,13-dimethyl-3,7,12-trioxohexadecahydro-1H-cyclopenta[a]phenanthren-17-yl)butanoyl)oxy)methyl)benzyl)-1H-1,2,3-triazol-4-yl)phenyl)-1H-1,2,3-triazol-1-yl)methyl)benzyl (2*S*,5*R*)-3,3-dimethyl-7-oxo-4-thia-1-azabicyclo[3.2.0]heptane-2-carboxylate 4,4-dioxide (4d)

87% yield; $[\alpha]_D^{25}$ = +7.2 (c 0.5, $CHCl_3$), dr > 20 : 1, a white foam, R_f = 0.30 (hexane/ethyl acetate 1:1). ^{1}H NMR (400 MHz, $CDCl_3$) δ 7.90 (d, *J* = 7.6 Hz, 1H), 7.80 (d, *J* = 7.6 Hz, 1H), 7.26 (dd, *J* = 8.1, 3.5 Hz, 4H), 7.13 (dt, *J* = 13.9, 7.1 Hz, 5H), 6.93 (s, 1H), 6.75 (s, 1H), 6.68 – 6.57 (m, 2H), 5.52 – 4.95 (m, 9H), 4.53 (dd, *J* = 4.1, 2.3 Hz, 1H), 4.33 (s, 1H), 3.40 (dd, *J* = 6.4, 3.2 Hz, 2H), 2.94 – 2.73 (m, 3H), 2.46 – 1.73 (m, 23H), 1.36 – 1.09 (m, 18H), 1.02 – 0.71 (m, 20H). ^{13}C NMR (101 MHz, $CDCl_3$) δ 212.0, 209.1, 208.8, 173.8, 134.5, 132.5, 131.8, 131.3, 130.8, 129.3, 128.8, 128.4, 128.2, 126.2, 123.4, 122.6, 67.5, 65.5, 63.2, 62.7, 61.1, 56.9, 53.6, 53.4, 51.8, 49.0, 46.9, 45.6, 45.0, 42.8, 38.6, 38.3, 36.5, 36.0, 35.5, 35.2, 34.8, 31.5, 30.5, 27.6, 25.1, 21.9, 21.0, 20.3, 18.6, 18.6, 18.0, 11.8. HRMS (ESI) m/z calcd for $C_{69}H_{80}N_7O_{11}S$ $[M+H]^+$ = 1214.5631, found = 1214.5639.

(3) Scale-up experiment

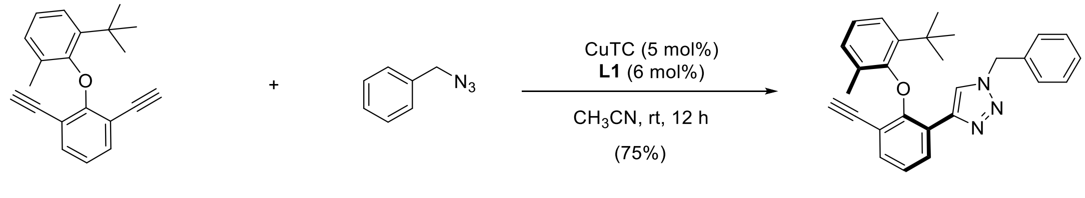

1b (5 mmol) **2a** **3aa**, 1.58 g, 98% ee

To a dried and argon-filled 10 mL Schlenk tube equipped with a magnetic stir bar was added CuTC (5 mol%) and *ent*-**L1** (6 mol%) in CH_3CN (20 ml). The reaction mixture was stirred under at room temperature for 1 h. Then the solution of **1b** (5 mmol, 1.0 equiv.) and **2a** (9 mmol, 1.8 equiv.) in CH_3CN (2 mL) was added. The reaction mixture was stirred at room temperature for 12 h. Then the reaction mixture was concentrated under reduced pressure and the residue was purified by column chromatography on silica gel to furnish the product **3aa** (1.58 g, 75%, 98% ee).

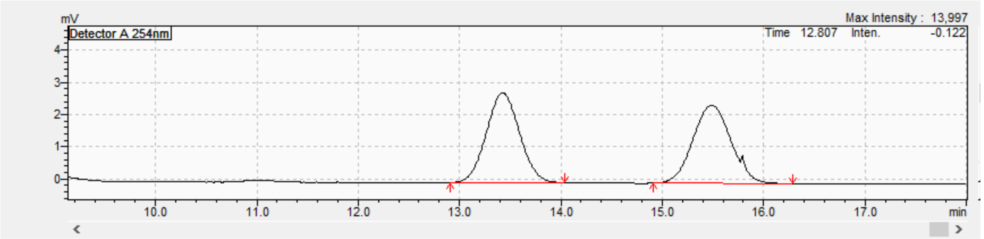

Results View - Peak Table

Peak Table | Compound | Group | Calibration Curve

Peak#	Ret. Time	Area	Height	Conc.	Unit	ID#	Name	Area%
1	13.426	63530	2787	49.892				49.892
2	15.490	63806	2424	50.108				50.108
Total		127336	5211	100.000				100.000

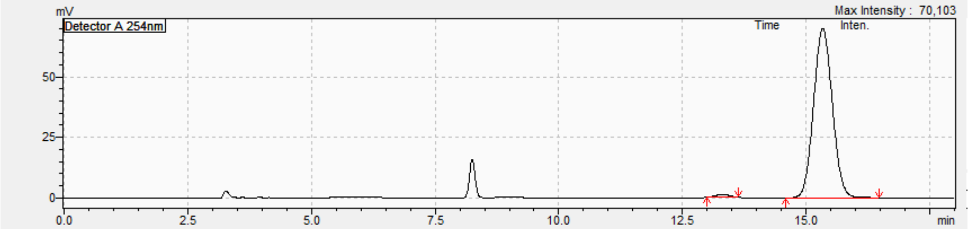

Results View - Peak Table

Peak Table | Compound | Group | Calibration Curve

Peak#	Ret. Time	Area	Height	Conc.	Unit	ID#	Name	Area%
1	13.313	22714	1116	1.216				1.216
2	15.337	1844567	70099	98.784				98.784
Total		1867281	71216	100.000				100.000

(4) Transformation of products

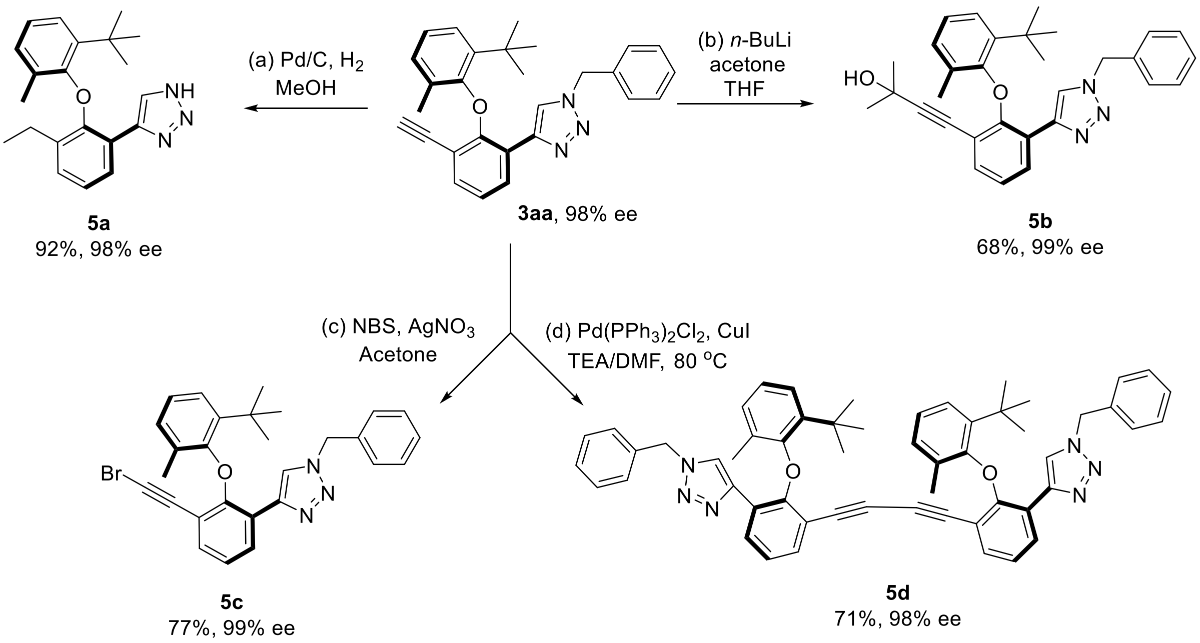

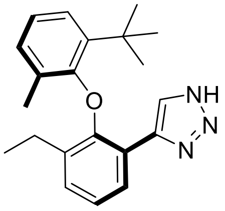

5a

4-(2-(2-(*tert*-Butyl)-6-methylphenoxy)-3-ethylphenyl)-1H-1,2,3-triazole (5a)

To a solution of **3aa** (0.1 mmol, 1.0 equiv.) in MeOH (2 mL) was added Pd/C (10%,0.01 mmol) at room temperature, and the flask was evacuated and purged with H_2 for 3 times. After stirring for 12 h, the mixture was filtered through Celite and washed with EtOAc. The filtrate was concentrated in vacuo to give a residue, which was purified by chromatography column to afford desired product. 92% yield; $[\alpha]_D^{25}$ = -105.6 (c 0.5, $CHCl_3$), a white foam, R_f = 0.33 (hexane/ethyl acetate 3:1). ^{1}H NMR (400 MHz, $CDCl_3$) δ 7.92 (s, 1H), 7.76 (dd, *J* = 7.6, 1.8 Hz, 1H), 7.26 – 7.14 (m, 3H), 7.08 (t, *J* = 7.6 Hz, 1H), 6.90 (t, *J* = 7.6 Hz, 1H), 6.84 (dd, *J* = 7.6, 1.8 Hz, 1H), 5.35 (s, 1H), 2.26 (dt, *J* = 15.1, 7.5 Hz, 1H), 2.10 (dt, *J* = 15.0, 7.5 Hz, 1H), 1.67 (s, 3H), 1.35 (s, 9H), 0.90 (t, *J* = 7.5 Hz, 3H). ^{13}C NMR (101 MHz, $CDCl_3$) δ 154.5, 151.7, 139.6, 133.9, 130.8, 130.8, 127.4, 127.1, 125.7, 123.5, 123.4, 35.4, 30.8, 22.4, 18.3, 14.1. HRMS (ESI) m/z calcd for $C_{21}H_{26}N_3O$ $[M+H]^+$ = 336.2070, found = 336.2074; the ee value was 98%, t_R (minor) = 15.4 min, t_R (major) = 22.8 min (Chiralpak IC, λ = 254 nm, 10% *i*-PrOH/Hexane, flow rate = 1.0 mL/min).

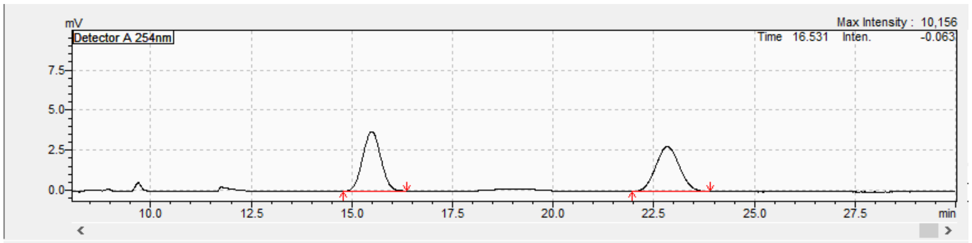

Peak#	Ret. Time	Area	Height	Conc.	Unit	ID#	Name	Area%
1	15.494	116889	3730	50.230				50.230
2	22.840	115819	2812	49.770				49.770
Total		232709	6542	100.000				100.000

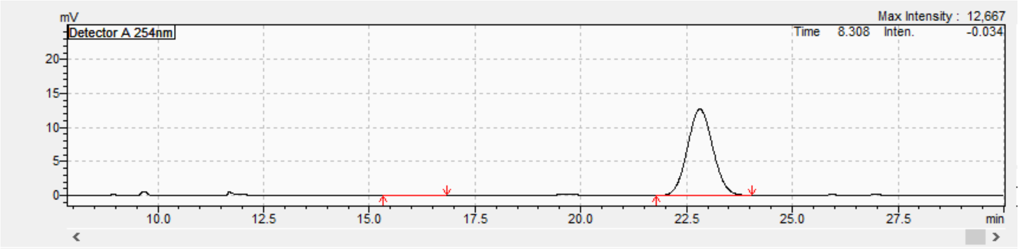

Results View - Peak Table

Peak Table | Compound | Group | Calibration Curve

Peak#	Ret. Time	Area	Height	Conc.	Unit	ID#	Name	Area%
1	15.369	1011	62	0.190				0.190
2	22.815	531032	12673	99.810				99.810
Total		532043	12735	100.000				100.000

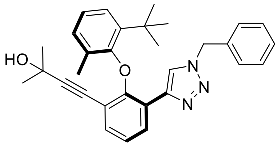

5b

4-(3-(1-Benzyl-1*H*-1,2,3-triazol-4-yl)-2-(2-(tert-butyl)-6-methylphenoxy)phenyl)-2-methylbut-3-yn-2-ol (5b)

To a solution of **3aa** (0.1 mmol, 1.0 equiv.) in THF (2 mL) was added *n*-BuLi (0.14 mmol) at -78 ^{o}C. After stirring for 30 min, acetone (0.2 mmol) was added to the reaction mixture. After 12 h, the reaction mixture was concentrated in vacuo to give a residue, which was purified by chromatography column to afford desired product. 68% yield; $[\alpha]_D^{25}$ = -120.2 (c 0.5, $CHCl_3$), a white foam, R_f = 0.33 (hexane/ethyl acetate 4:1). ^{1}H NMR (400 MHz, $CDCl_3$) δ 8.38 (dd, *J* = 7.8, 1.8 Hz, 1H), 7.91 (s, 1H), 7.31 – 7.14 (m, 7H), 7.06 – 6.92 (m, 3H), 5.60 (d, *J* = 14.9 Hz, 1H), 5.34 (d, *J* = 15.0 Hz, 1H), 1.76 (s, 3H), 1.22 (s, 9H), 1.17 (s, 3H), 1.15 (s, 3H). ^{13}C NMR (101 MHz, $CDCl_3$) δ 153.4, 152.5, 143.4, 142.2, 135.4, 134.8, 131.2, 129.5, 129.0, 128.8, 128.6, 127.8, 124.9, 123.9, 123.2, 122.3, 121.7, 111.3, 100.0, 77.7, 64.7, 54.2, 35.2, 30.7, 30.7, 30.5, 17.6. HRMS (ESI) m/z calcd for $C_{31}H_{34}N_3O_2$ $[M+H]^+$ = 480.2646, found = 480.2649; the ee value was 99%, t_R (minor) = 20.4 min, t_R (major) = 30.4 min (Chiralpak IC, λ = 254 nm, 10% *i*-PrOH/Hexane, flow rate = 1.0 mL/min).

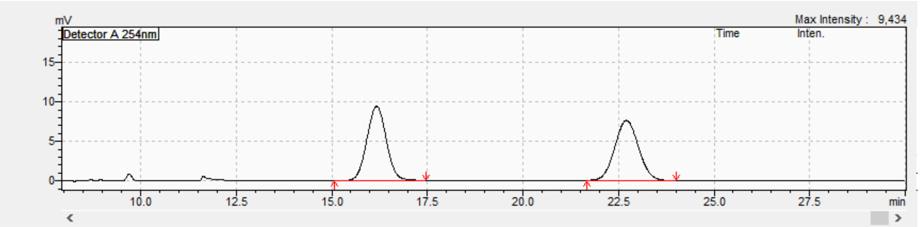

Results View - Peak Table

Peak Table | Compound | Group | Calibration Curve

Peak#	Ret. Time	Area	Height	Conc.	Unit	ID#	Name	Area%
1	16.173	333641	9407	50.416				50.416
2	22.705	328133	7600	49.584				49.584
Total		661774	17007	100.000				100.000

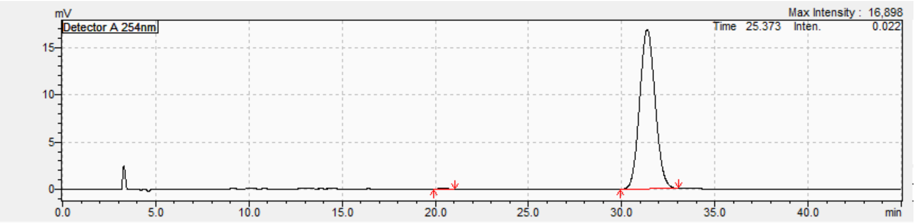

Results View - Peak Table

Peak Table | Compound | Group | Calibration Curve

Peak#	Ret. Time	Area	Height	Conc.	Unit	ID#	Name	Area%
1	20.386	3784	111	0.388				0.388
2	31.368	970823	16832	99.612				99.612
Total		974607	16943	100.000				100.000

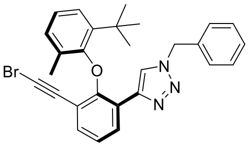

5c

1-Benzyl-4-(3-(bromoethynyl)-2-(2-(*tert*-butyl)-6-methylphenoxy)phenyl)-1*H*-1,2,3-triazole (5c)

To a solution of **3aa** (0.1 mmol, 1.0 equiv.) in acetone (2 mL) was added $AgNO_3$(0.12 mmol) and NBS (0.12 mmol) at room temperature. After stirring for 12 h, the mixture was filtered through Celite and washed with EtOAc. The filtrate was concentrated in vacuo to give a residue, which was purified by chromatography column to afford desired product. 77% yield; $[\alpha]_D^{25}$ = -85.0 (c 0.5, $CHCl_3$), a white foam, R_f = 0.34 (hexane/ethyl acetate 8:1). ^{1}H NMR (400 MHz, $CDCl_3$) δ 8.41 (dd, *J* = 7.8, 1.8 Hz, 1H), 7.96 (s, 1H), 7.28 – 7.13 (m, 7H), 7.05 – 6.86 (m, 3H), 5.59 (d, *J* = 15.0 Hz, 1H), 5.35 (d, *J* = 14.9 Hz, 1H), 1.71 (s, 3H), 1.23 (s, 9H). ^{13}C NMR (101 MHz, $CDCl_3$) δ 152.5, 151.3, 142.2, 140.6, 134.3, 133.7, 128.6, 128.5, 128.0, 128.0, 127.6, 126.8, 124.3, 124.1, 122.2, 121.2, 120.8, 110.2, 74.4, 53.1, 34.1, 29.8, 16.3. HRMS (ESI) m/z calcd for $C_{28}H_{27}BrN_3O$ $[M+H]^+$ = 500.1332, found = 500.1334; the ee value was 99%, t_R (minor) = 15.8 min, t_R (major) = 20.0 min (Chiralpak IC, λ = 254 nm, 10% *i*-PrOH/Hexane, flow rate = 1.0 mL/min).

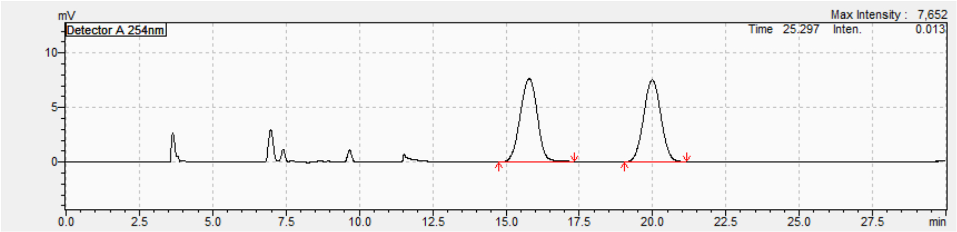

Results View - Peak Table

Peak Table | Compound | Group | Calibration Curve

Peak#	Ret. Time	Area	Height	Conc.	Unit	ID#	Name	Area%
1	15.793	311197	7629	50.284				50.284
2	19.988	307685	7521	49.716				49.716
Total		618882	15150	100.000				100.000

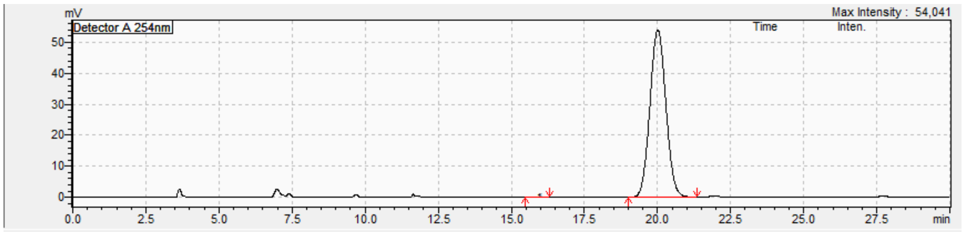

Results View - Peak Table

Peak Table | Compound | Group | Calibration Curve

Peak#	Ret. Time	Area	Height	Conc.	Unit	ID#	Name	Area%
1	15.800	2556	82	0.124				0.124
2	20.011	2060819	54064	99.876				99.876
Total		2063376	54145	100.000				100.000

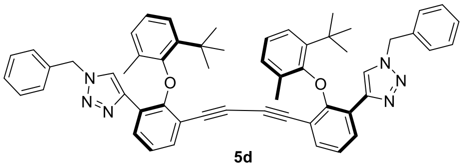

5d

1,4-Bis(3-(1-benzyl-1*H*-1,2,3-triazol-4-yl)-2-(2-(*tert*-butyl)-6-methylphenoxy)phenyl)buta-1,3-diyne (5d)

3aa (0.1 mmol, 1.0 equiv.), $Pd(PPh_3)_2Cl_2$ (0.01 mmol, 0.1 equiv.), and CuI (0.01 mmol, 0.1 equiv.) were added into a sealed tube. TEA (1 mL) and DMF (1 mL) was added into the mixture. The suspension was stirred at 60 °C for 12 h. After cooling to room temperature, the mixture was extracted with EtOAc and concentrated in vacuo to give a residue, which was purified by column to afford desired product 71% yield; $[\alpha]_D^{25}$ = +60.2 (c 0.5, $CHCl_3$), a white foam, R_f = 0.30 (hexane/ethyl acetate 8:1). ^{1}H NMR (400 MHz, $CDCl_3$) δ 8.42 (dd, *J* = 7.8, 1.8 Hz, 1H), 7.95 (s, 1H), 7.32 – 6.89 (m, 10H), 5.59 (d, *J* = 15.0 Hz, 1H), 5.36 (d, *J* = 15.0 Hz, 1H), 1.71 (s, 3H), 1.15 (s, 9H). ^{13}C NMR (101 MHz, $CDCl_3$) δ 154.1, 152.2, 143.1, 142.0, 135.4, 134.7, 130.1, 129.3, 129.2, 129.1, 128.6, 127.9, 125.3, 125.1, 123.3, 122.1, 121.5, 110.5, 79.7, 77.8, 54.2, 35.1, 30.9, 17.2.

HRMS (ESI) m/z calcd for $C_{56}H_{53}N_6O_2$ $[M+H]^+$ = 841.4225, found = 841.4229; the ee value was 99%, t_R (minor) = 15.8 min, t_R (major) = 20.0 min (Chiralpak IE, λ = 254 nm, 8% *i*-PrOH/Hexane, flow rate = 1.0 mL/min).

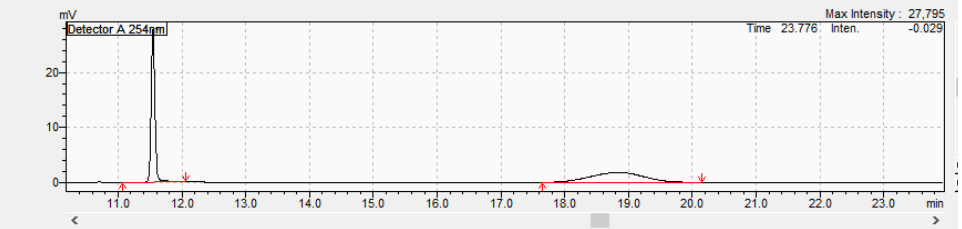

Results View - Peak Table

Peak Table | Compound | Group | Calibration Curve

Peak#	Ret. Time	Area	Height	Conc.	Unit	ID#	Name	Area%
1	11.543	111007	27745	50.839				50.839
2	18.845	107342	1805	49.161				49.161
Total		218349	29549	100.000				100.000

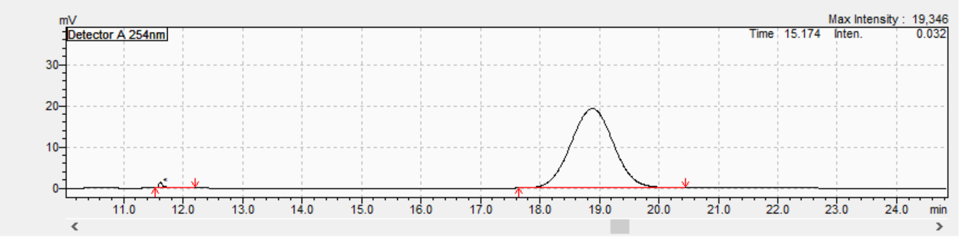

Results View - Peak Table

Peak Table | Compound | Group | Calibration Curve

Peak#	Ret. Time	Area	Height	Conc.	Unit	ID#	Name	Area%
1	11.615	9327	1351	0.961				0.961
2	18.881	961421	19270	99.039				99.039
Total		970747	20622	100.000				100.000

7. Mechanistic Studies

(1) Nonlinear relationship between the ee values of **3a** and **L1**

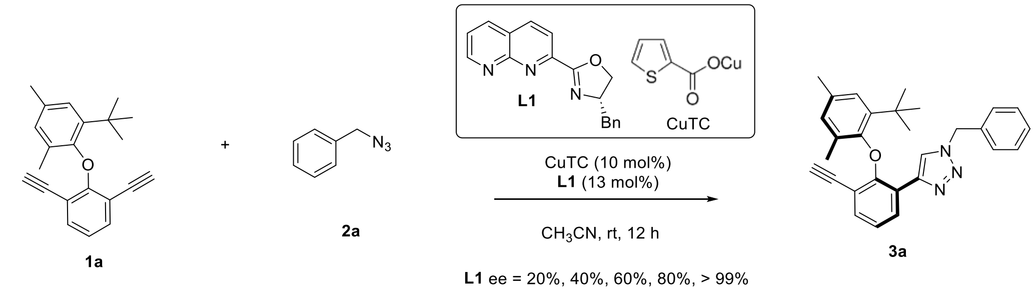

To a dried and argon-filled 10 mL Schlenk tube equipped with a magnetic stir bar was added CuTC (10 mol%) and **L1** (13 mol%) in CH_3CN (1.5 ml). The reaction mixture was stirred under at room temperature for 1 h. Then the solution of **1** (0.05 mmol, 1.0 equiv.) and **2** (0.09 mmol, 1.8 equiv.) in CH_3CN (0.5 mL) was added. The reaction mixture was stirred at room temperature for 12 h. The ee value was determined by HPLC analysis on a chiral-stationary-phase.

ee of **L1** (%)	ee of **3a** (%)
0	0
20	29
40	51
60	69
80	84
100	98

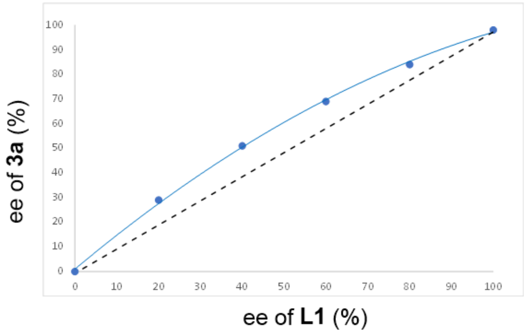

(2) Evidence for synergic desymmetrization and kinetic resolution.

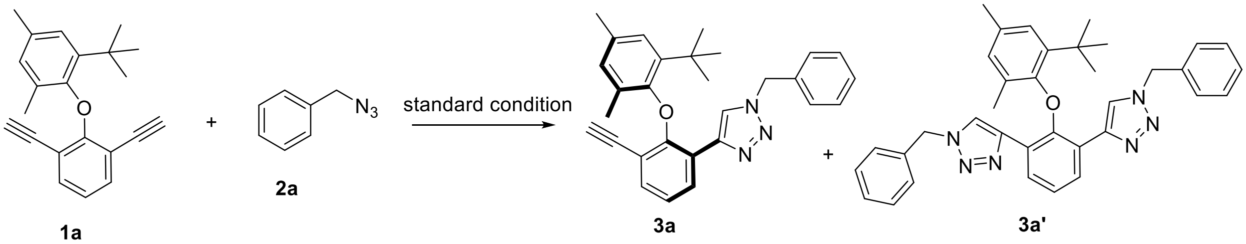

To a dried and argon-filled 10 mL Schlenk tube equipped with a magnetic stir bar was added CuTC (10 mol%) and **L1** (13 mol%) in CH_3CN (1.5 ml). The reaction mixture was stirred under at room temperature for 1 h. Then the solution of **1** (0.05 mmol, 1.0 equiv.) and **2** (0.09 mmol, 1.8 equiv.) in CH_3CN (0.5 mL) was added. The reaction mixture was stirred at room temperature. The yield and ee values were determined at specific time.

entry	time	yield of **3a**	ee of **3a**
1	1h	99%	73%
2	2h	94%	76%
3	3h	87%	82%
4	4h	84%	87%
5	5h	82%	90%
6	6h	80%	92%
7	7h	78%	94%
8	8h	77%	96%
9	9h	76%	96%

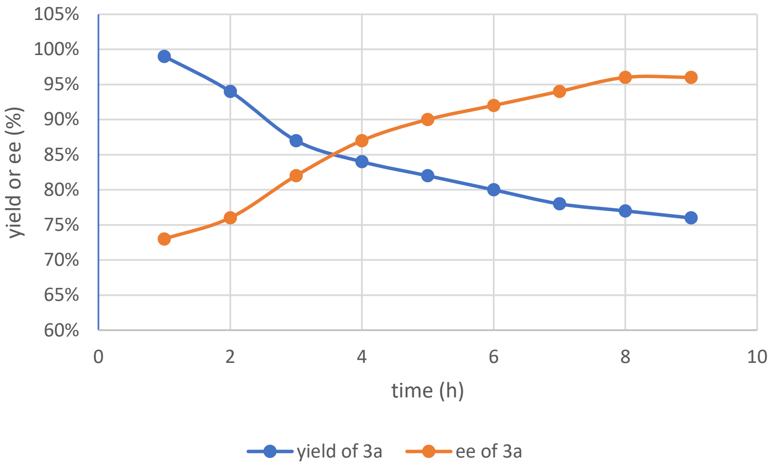

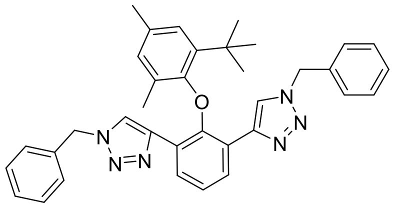

3a'

4,4'-(2-(2-(*tert*-Butyl)-4,6-dimethylphenoxy)-1,3-phenylene)bis(1-benzyl-1H-1,2,3-triazole) (3a')

^{1}H NMR (400 MHz, $CDCl_3$) δ 7.85 (d, *J* = 7.7 Hz, 2H), 7.29 – 7.23 (m, 6H), 7.19 (d, *J* = 1.6 Hz, 1H), 7.15 – 7.08 (m, 4H), 6.82 (s, 2H), 6.66 – 6.54 (m, 2H), 5.36 (d, *J* = 14.8 Hz, 2H), 5.16 (d, *J* = 14.8 Hz, 2H), 2.21 (s, 3H), 1.67 (s, 4H), 0.83 (s, 10H). ^{13}C NMR (101 MHz, $CDCl_3$) δ 152.8, 151.0, 143.9, 140.5, 134.5, 132.4, 131.5, 130.8, 129.0, 128.7, 128.1, 128.1, 126.3, 123.3, 122.9, 122.7, 53.9, 34.8, 30.4, 21.0, 18.0. HRMS (ESI) m/z calcd for $C_{36}H_{37}N_6O$ $[M+H]^+$ = 569.3023, found = 569.3028.

8. Single Crystal Structure X-ray Analysis of 3w

<u>Single Crystal Structure X-ray Analysis</u>

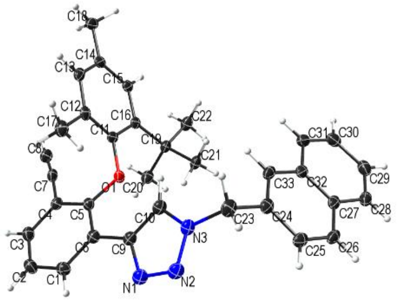

Sample Code: N182

Sample ID: DL-748

Student/Researcher: Dai Lei

Supervisor: Prof Lu Yixin

CCDC 2322008

Date: 15-5-2023

Note: The crystal is orthorhombic, space group P2(1)2(1)2(1). The asymmetric unit contains one molecule of the compound $C_{33}H_{31}N_3O$.

As the Flack x =-0.055(211) by classical fit to all intensities and =0.026(20) from 2195 selected quotients by Parsons' method (in the LST file attached), the reported structure is the correct hand.

Final R values are R1=0.0251 and wR2=0.0639 for 2- theta up to 144º.

Table 1. Crystal data and structure refinement for N182.

Identification code	N182	
Empirical formula	C33 H31 N3 O	
Formula weight	485.61	
Temperature	100(2) K	
Wavelength	1.54178 Å	
Crystal system	Orthorhombic	
Space group	$P2_12_12_1$	
Unit cell dimensions	a = 7.8263(4) Å	α= 90°.
	b = 11.6180(6) Å	β= 90°.
	c = 28.9031(15) Å	γ = 90°.
Volume	2628.0(2) $Å^3$	
Z	4	
Density (calculated)	1.227 Mg/m^3	
Absorption coefficient	0.580 mm^{-1}	
F(000)	1032	
Crystal size	0.251 x 0.146 x 0.063 mm^3	
Theta range for data collection	3.058 to 72.293°.	
Index ranges	-9<=h<=9, -14<=k<=14, -35<=l<=35	
Reflections collected	129942	
Independent reflections	5185 [R(int) = 0.0252]	
Completeness to theta = 67.679°	100.0 %	
Absorption correction	Semi-empirical from equivalents	
Max. and min. transmission	0.7536 and 0.6953	
Refinement method	Full-matrix least-squares on F^2	
Data / restraints / parameters	5185 / 0 / 339	
Goodness-of-fit on F^2	1.067	
Final R indices [I>2sigma(I)]	R1 = 0.0251, wR2 = 0.0637	
R indices (all data)	R1 = 0.0252, wR2 = 0.0639	
Absolute structure parameter	0.03(2)	
Extinction coefficient	n/a	
Largest diff. peak and hole	0.141 and -0.209 e.$Å^{-3}$	

Table 2. Atomic coordinates (x 10^4) and equivalent isotropic displacement parameters (Å^{2}x 10^3) for N182. U(eq) is defined as one third of the trace of the orthogonalized U^{ij} tensor.

	x	y	z	U(eq)
O(1)	5021(1)	5059(1)	3506(1)	19(1)
N(1)	3496(2)	8278(1)	3106(1)	24(1)
N(2)	4279(2)	9073(1)	3350(1)	24(1)
N(3)	5499(2)	8542(1)	3600(1)	21(1)
C(1)	2582(2)	6307(1)	2565(1)	22(1)
C(2)	2043(2)	5347(1)	2321(1)	24(1)
C(3)	2507(2)	4258(1)	2469(1)	22(1)
C(4)	3518(2)	4105(1)	2866(1)	19(1)
C(5)	4079(2)	5086(1)	3104(1)	17(1)
C(6)	3611(2)	6193(1)	2958(1)	19(1)
C(7)	3876(2)	2942(1)	3009(1)	20(1)
C(8)	4044(2)	1948(1)	3093(1)	24(1)
C(9)	4210(2)	7232(1)	3200(1)	19(1)
C(10)	5497(2)	7400(1)	3519(1)	20(1)
C(11)	6258(2)	4216(1)	3588(1)	16(1)
C(12)	7600(2)	4081(1)	3273(1)	19(1)
C(13)	8798(2)	3224(1)	3364(1)	20(1)
C(14)	8693(2)	2536(1)	3755(1)	19(1)
C(15)	7411(2)	2772(1)	4079(1)	18(1)
C(16)	6182(2)	3629(1)	4011(1)	16(1)
C(17)	7786(2)	4849(1)	2854(1)	24(1)
C(18)	9945(2)	1567(1)	3835(1)	24(1)
C(19)	4825(2)	3910(1)	4379(1)	18(1)
C(20)	3049(2)	3557(1)	4203(1)	25(1)
C(21)	4847(2)	5200(1)	4505(1)	21(1)
C(22)	5159(2)	3256(1)	4833(1)	24(1)
C(23)	6610(2)	9200(1)	3910(1)	24(1)
C(24)	6073(2)	9126(1)	4413(1)	21(1)
C(25)	5083(2)	10023(1)	4609(1)	23(1)
C(26)	4653(2)	10004(1)	5068(1)	23(1)
C(27)	5154(2)	9080(1)	5357(1)	21(1)

C(28)	4782(2)	9059(1)	5839(1)	25(1)
C(29)	5306(2)	8155(1)	6107(1)	28(1)
C(30)	6205(2)	7223(1)	5909(1)	28(1)
C(31)	6600(2)	7226(1)	5447(1)	24(1)
C(32)	6097(2)	8156(1)	5160(1)	20(1)
C(33)	6561(2)	8216(1)	4685(1)	21(1)

Table 3. Bond lengths [Å] and angles [°] for N182.

O(1)-C(5)	1.3761(15)
O(1)-C(11)	1.3970(16)
N(1)-N(2)	1.3143(18)
N(1)-C(9)	1.3646(18)
N(2)-N(3)	1.3456(18)
N(3)-C(10)	1.3470(17)
N(3)-C(23)	1.4646(18)
C(1)-C(2)	1.384(2)
C(1)-C(6)	1.3981(19)
C(1)-H(1)	0.9500
C(2)-C(3)	1.384(2)
C(2)-H(2)	0.9500
C(3)-C(4)	1.405(2)
C(3)-H(3)	0.9500
C(4)-C(5)	1.4027(19)
C(4)-C(7)	1.4395(19)
C(5)-C(6)	1.4022(18)
C(6)-C(9)	1.4727(18)
C(7)-C(8)	1.188(2)
C(8)-H(8)	0.9500
C(9)-C(10)	1.380(2)
C(10)-H(10)	0.9500
C(11)-C(12)	1.3979(18)
C(11)-C(16)	1.4023(18)
C(12)-C(13)	1.392(2)
C(12)-C(17)	1.5108(18)
C(13)-C(14)	1.388(2)
C(13)-H(13)	0.9500
C(14)-C(15)	1.3995(19)
C(14)-C(18)	1.5099(19)
C(15)-C(16)	1.3977(19)
C(15)-H(15)	0.9500
C(16)-C(19)	1.5386(17)
C(17)-H(17A)	0.9800

C(17)-H(17B)	0.9800
C(17)-H(17C)	0.9800
C(18)-H(18A)	0.9800
C(18)-H(18B)	0.9800
C(18)-H(18C)	0.9800
C(19)-C(20)	1.5363(19)
C(19)-C(22)	1.5385(18)
C(19)-C(21)	1.5419(17)
C(20)-H(20A)	0.9800
C(20)-H(20B)	0.9800
C(20)-H(20C)	0.9800
C(21)-H(21A)	0.9800
C(21)-H(21B)	0.9800
C(21)-H(21C)	0.9800
C(22)-H(22A)	0.9800
C(22)-H(22B)	0.9800
C(22)-H(22C)	0.9800
C(23)-C(24)	1.5145(19)
C(23)-H(23A)	0.9900
C(23)-H(23B)	0.9900
C(24)-C(33)	1.371(2)
C(24)-C(25)	1.4172(19)
C(25)-C(26)	1.371(2)
C(25)-H(25)	0.9500
C(26)-C(27)	1.415(2)
C(26)-H(26)	0.9500
C(27)-C(32)	1.4223(19)
C(27)-C(28)	1.4225(19)
C(28)-C(29)	1.370(2)
C(28)-H(28)	0.9500
C(29)-C(30)	1.412(2)
C(29)-H(29)	0.9500
C(30)-C(31)	1.372(2)
C(30)-H(30)	0.9500
C(31)-C(32)	1.418(2)
C(31)-H(31)	0.9500

C(32)-C(33)	1.4211(19)
C(33)-H(33)	0.9500
C(5)-O(1)-C(11)	121.97(10)
N(2)-N(1)-C(9)	109.16(12)
N(1)-N(2)-N(3)	107.22(11)
N(2)-N(3)-C(10)	111.01(12)
N(2)-N(3)-C(23)	120.64(11)
C(10)-N(3)-C(23)	128.34(13)
C(2)-C(1)-C(6)	120.81(13)
C(2)-C(1)-H(1)	119.6
C(6)-C(1)-H(1)	119.6
C(3)-C(2)-C(1)	119.96(13)
C(3)-C(2)-H(2)	120.0
C(1)-C(2)-H(2)	120.0
C(2)-C(3)-C(4)	121.06(13)
C(2)-C(3)-H(3)	119.5
C(4)-C(3)-H(3)	119.5
C(5)-C(4)-C(3)	118.28(12)
C(5)-C(4)-C(7)	124.15(12)
C(3)-C(4)-C(7)	117.55(12)
O(1)-C(5)-C(6)	114.62(11)
O(1)-C(5)-C(4)	124.26(12)
C(6)-C(5)-C(4)	121.01(12)
C(1)-C(6)-C(5)	118.87(12)
C(1)-C(6)-C(9)	119.45(12)
C(5)-C(6)-C(9)	121.66(12)
C(8)-C(7)-C(4)	172.96(15)
C(7)-C(8)-H(8)	180.0
N(1)-C(9)-C(10)	107.81(12)
N(1)-C(9)-C(6)	120.36(12)
C(10)-C(9)-C(6)	131.78(12)
N(3)-C(10)-C(9)	104.80(12)
N(3)-C(10)-H(10)	127.6
C(9)-C(10)-H(10)	127.6
O(1)-C(11)-C(12)	119.35(11)

O(1)-C(11)-C(16)	117.27(11)
C(12)-C(11)-C(16)	123.01(12)
C(13)-C(12)-C(11)	117.66(12)
C(13)-C(12)-C(17)	120.54(12)
C(11)-C(12)-C(17)	121.78(12)
C(14)-C(13)-C(12)	121.68(12)
C(14)-C(13)-H(13)	119.2
C(12)-C(13)-H(13)	119.2
C(13)-C(14)-C(15)	118.31(12)
C(13)-C(14)-C(18)	121.08(13)
C(15)-C(14)-C(18)	120.61(12)
C(16)-C(15)-C(14)	122.67(12)
C(16)-C(15)-H(15)	118.7
C(14)-C(15)-H(15)	118.7
C(15)-C(16)-C(11)	116.05(12)
C(15)-C(16)-C(19)	121.94(11)
C(11)-C(16)-C(19)	122.01(12)
C(12)-C(17)-H(17A)	109.5
C(12)-C(17)-H(17B)	109.5
H(17A)-C(17)-H(17B)	109.5
C(12)-C(17)-H(17C)	109.5
H(17A)-C(17)-H(17C)	109.5
H(17B)-C(17)-H(17C)	109.5
C(14)-C(18)-H(18A)	109.5
C(14)-C(18)-H(18B)	109.5
H(18A)-C(18)-H(18B)	109.5
C(14)-C(18)-H(18C)	109.5
H(18A)-C(18)-H(18C)	109.5
H(18B)-C(18)-H(18C)	109.5
C(20)-C(19)-C(22)	107.77(11)
C(20)-C(19)-C(16)	109.76(10)
C(22)-C(19)-C(16)	111.58(11)
C(20)-C(19)-C(21)	110.34(11)
C(22)-C(19)-C(21)	106.07(10)
C(16)-C(19)-C(21)	111.21(11)
C(19)-C(20)-H(20A)	109.5

C(19)-C(20)-H(20B)	109.5
H(20A)-C(20)-H(20B)	109.5
C(19)-C(20)-H(20C)	109.5
H(20A)-C(20)-H(20C)	109.5
H(20B)-C(20)-H(20C)	109.5
C(19)-C(21)-H(21A)	109.5
C(19)-C(21)-H(21B)	109.5
H(21A)-C(21)-H(21B)	109.5
C(19)-C(21)-H(21C)	109.5
H(21A)-C(21)-H(21C)	109.5
H(21B)-C(21)-H(21C)	109.5
C(19)-C(22)-H(22A)	109.5
C(19)-C(22)-H(22B)	109.5
H(22A)-C(22)-H(22B)	109.5
C(19)-C(22)-H(22C)	109.5
H(22A)-C(22)-H(22C)	109.5
H(22B)-C(22)-H(22C)	109.5
N(3)-C(23)-C(24)	113.16(11)
N(3)-C(23)-H(23A)	108.9
C(24)-C(23)-H(23A)	108.9
N(3)-C(23)-H(23B)	108.9
C(24)-C(23)-H(23B)	108.9
H(23A)-C(23)-H(23B)	107.8
C(33)-C(24)-C(25)	119.33(13)
C(33)-C(24)-C(23)	121.12(12)
C(25)-C(24)-C(23)	119.53(13)
C(26)-C(25)-C(24)	120.61(13)
C(26)-C(25)-H(25)	119.7
C(24)-C(25)-H(25)	119.7
C(25)-C(26)-C(27)	121.09(13)
C(25)-C(26)-H(26)	119.5
C(27)-C(26)-H(26)	119.5
C(26)-C(27)-C(32)	118.66(12)
C(26)-C(27)-C(28)	122.25(13)
C(32)-C(27)-C(28)	119.08(13)
C(29)-C(28)-C(27)	120.45(13)

C(29)-C(28)-H(28)	119.8
C(27)-C(28)-H(28)	119.8
C(28)-C(29)-C(30)	120.48(14)
C(28)-C(29)-H(29)	119.8
C(30)-C(29)-H(29)	119.8
C(31)-C(30)-C(29)	120.34(14)
C(31)-C(30)-H(30)	119.8
C(29)-C(30)-H(30)	119.8
C(30)-C(31)-C(32)	120.66(13)
C(30)-C(31)-H(31)	119.7
C(32)-C(31)-H(31)	119.7
C(31)-C(32)-C(33)	122.13(13)
C(31)-C(32)-C(27)	118.96(13)
C(33)-C(32)-C(27)	118.86(13)
C(24)-C(33)-C(32)	121.38(12)
C(24)-C(33)-H(33)	119.3
C(32)-C(33)-H(33)	119.3

Symmetry transformations used to generate equivalent atoms:

Table 4. Anisotropic displacement parameters ($Å^2$x 10^3) for N182. The anisotropic displacement factor exponent takes the form: $-2\pi^2$[h^2 $a^{*2}U^{11}$ + ... + 2 h k a* b* U^{12}]

	U^{11}	U^{22}	U^{33}	U^{23}	U^{13}	U^{12}
O(1)	25(1)	16(1)	16(1)	-1(1)	-3(1)	2(1)
N(1)	29(1)	19(1)	22(1)	4(1)	1(1)	1(1)
N(2)	31(1)	17(1)	24(1)	4(1)	3(1)	2(1)
N(3)	26(1)	16(1)	21(1)	1(1)	3(1)	-2(1)
C(1)	22(1)	24(1)	20(1)	5(1)	2(1)	1(1)
C(2)	23(1)	33(1)	18(1)	2(1)	-2(1)	-2(1)
C(3)	21(1)	27(1)	20(1)	-4(1)	1(1)	-4(1)
C(4)	19(1)	19(1)	18(1)	-1(1)	4(1)	-1(1)
C(5)	18(1)	20(1)	14(1)	1(1)	2(1)	-1(1)
C(6)	20(1)	19(1)	18(1)	2(1)	3(1)	-1(1)
C(7)	21(1)	22(1)	18(1)	-5(1)	1(1)	-2(1)
C(8)	27(1)	20(1)	25(1)	-4(1)	1(1)	-1(1)
C(9)	23(1)	16(1)	17(1)	4(1)	4(1)	0(1)
C(10)	25(1)	15(1)	21(1)	1(1)	3(1)	-1(1)
C(11)	17(1)	12(1)	18(1)	-2(1)	-2(1)	-1(1)
C(12)	21(1)	19(1)	16(1)	-4(1)	0(1)	-6(1)
C(13)	17(1)	24(1)	20(1)	-7(1)	2(1)	-4(1)
C(14)	16(1)	20(1)	22(1)	-6(1)	-4(1)	-2(1)
C(15)	20(1)	16(1)	17(1)	-2(1)	-2(1)	-2(1)
C(16)	18(1)	14(1)	17(1)	-3(1)	0(1)	-4(1)
C(17)	26(1)	27(1)	19(1)	1(1)	5(1)	-4(1)
C(18)	20(1)	23(1)	29(1)	-6(1)	-3(1)	2(1)
C(19)	20(1)	16(1)	18(1)	-1(1)	4(1)	-1(1)
C(20)	20(1)	29(1)	28(1)	-6(1)	5(1)	-4(1)
C(21)	27(1)	17(1)	19(1)	-3(1)	3(1)	2(1)
C(22)	33(1)	20(1)	20(1)	2(1)	6(1)	0(1)
C(23)	26(1)	16(1)	28(1)	-2(1)	3(1)	-5(1)
C(24)	20(1)	17(1)	26(1)	-4(1)	0(1)	-5(1)
C(25)	23(1)	14(1)	31(1)	-2(1)	-2(1)	-1(1)
C(26)	20(1)	16(1)	32(1)	-7(1)	1(1)	1(1)
C(27)	16(1)	19(1)	27(1)	-7(1)	-1(1)	-4(1)

C(28)	20(1)	26(1)	28(1)	-9(1)	0(1)	-1(1)
C(29)	26(1)	36(1)	23(1)	-5(1)	-1(1)	-4(1)
C(30)	27(1)	28(1)	28(1)	1(1)	-7(1)	-1(1)
C(31)	22(1)	22(1)	29(1)	-4(1)	-4(1)	3(1)
C(32)	17(1)	18(1)	26(1)	-6(1)	-2(1)	-2(1)
C(33)	19(1)	16(1)	27(1)	-7(1)	1(1)	0(1)

Table 5. Hydrogen coordinates (x 10^4) and isotropic displacement parameters ($Å^2$x 10^3) for N182.

	x	y	z	U(eq)
H(1)	2247	7052	2464	27
H(2)	1356	5436	2053	29
H(3)	2136	3604	2300	27
H(8)	4178	1152	3160	29
H(10)	6223	6835	3653	24
H(13)	9711	3107	3152	24
H(15)	7374	2333	4356	21
H(17A)	8987	4872	2759	36
H(17B)	7401	5628	2932	36
H(17C)	7090	4544	2600	36
H(18A)	10379	1608	4153	36
H(18B)	10899	1638	3618	36
H(18C)	9369	828	3788	36
H(20A)	2204	3674	4448	38
H(20B)	3065	2744	4113	38
H(20C)	2746	4029	3934	38
H(21A)	4110	5332	4774	32
H(21B)	4427	5651	4242	32
H(21C)	6018	5435	4579	32
H(22A)	4290	3468	5062	36
H(22B)	6294	3458	4952	36
H(22C)	5107	2426	4775	36
H(23A)	6604	10017	3813	28
H(23B)	7794	8911	3880	28
H(25)	4715	10645	4420	27
H(26)	4008	10620	5195	27
H(28)	4165	9677	5975	30
H(29)	5063	8154	6429	34
H(30)	6539	6591	6097	33
H(31)	7216	6599	5318	29

H(33)	7223	7614	4553	25

Table 6. Torsion angles [°] for N182.

C(9)-N(1)-N(2)-N(3)	0.20(14)
N(1)-N(2)-N(3)-C(10)	-0.44(15)
N(1)-N(2)-N(3)-C(23)	179.90(11)
C(6)-C(1)-C(2)-C(3)	0.8(2)
C(1)-C(2)-C(3)-C(4)	0.2(2)
C(2)-C(3)-C(4)-C(5)	-1.4(2)
C(2)-C(3)-C(4)-C(7)	177.16(13)
C(11)-O(1)-C(5)-C(6)	-148.01(11)
C(11)-O(1)-C(5)-C(4)	35.80(18)
C(3)-C(4)-C(5)-O(1)	177.55(12)
C(7)-C(4)-C(5)-O(1)	-0.9(2)
C(3)-C(4)-C(5)-C(6)	1.59(19)
C(7)-C(4)-C(5)-C(6)	-176.86(13)
C(2)-C(1)-C(6)-C(5)	-0.6(2)
C(2)-C(1)-C(6)-C(9)	177.91(13)
O(1)-C(5)-C(6)-C(1)	-176.94(12)
C(4)-C(5)-C(6)-C(1)	-0.62(19)
O(1)-C(5)-C(6)-C(9)	4.58(18)
C(4)-C(5)-C(6)-C(9)	-179.09(13)
N(2)-N(1)-C(9)-C(10)	0.10(15)
N(2)-N(1)-C(9)-C(6)	-177.70(12)
C(1)-C(6)-C(9)-N(1)	13.73(19)
C(5)-C(6)-C(9)-N(1)	-167.80(12)
C(1)-C(6)-C(9)-C(10)	-163.45(14)
C(5)-C(6)-C(9)-C(10)	15.0(2)
N(2)-N(3)-C(10)-C(9)	0.49(15)
C(23)-N(3)-C(10)-C(9)	-179.88(12)
N(1)-C(9)-C(10)-N(3)	-0.35(15)
C(6)-C(9)-C(10)-N(3)	177.10(13)
C(5)-O(1)-C(11)-C(12)	56.98(16)
C(5)-O(1)-C(11)-C(16)	-129.82(12)
O(1)-C(11)-C(12)-C(13)	-179.38(11)
C(16)-C(11)-C(12)-C(13)	7.84(19)
O(1)-C(11)-C(12)-C(17)	2.03(18)

C(16)-C(11)-C(12)-C(17)	-170.76(12)
C(11)-C(12)-C(13)-C(14)	-1.07(19)
C(17)-C(12)-C(13)-C(14)	177.54(12)
C(12)-C(13)-C(14)-C(15)	-4.41(19)
C(12)-C(13)-C(14)-C(18)	176.61(12)
C(13)-C(14)-C(15)-C(16)	3.62(19)
C(18)-C(14)-C(15)-C(16)	-177.39(12)
C(14)-C(15)-C(16)-C(11)	2.58(18)
C(14)-C(15)-C(16)-C(19)	-177.53(12)
O(1)-C(11)-C(16)-C(15)	178.59(10)
C(12)-C(11)-C(16)-C(15)	-8.48(18)
O(1)-C(11)-C(16)-C(19)	-1.30(17)
C(12)-C(11)-C(16)-C(19)	171.62(12)
C(15)-C(16)-C(19)-C(20)	-112.07(14)
C(11)-C(16)-C(19)-C(20)	67.82(15)
C(15)-C(16)-C(19)-C(22)	7.34(17)
C(11)-C(16)-C(19)-C(22)	-172.78(12)
C(15)-C(16)-C(19)-C(21)	125.55(13)
C(11)-C(16)-C(19)-C(21)	-54.56(16)
N(2)-N(3)-C(23)-C(24)	100.35(15)
C(10)-N(3)-C(23)-C(24)	-79.25(18)
N(3)-C(23)-C(24)-C(33)	83.52(17)
N(3)-C(23)-C(24)-C(25)	-97.93(15)
C(33)-C(24)-C(25)-C(26)	1.8(2)
C(23)-C(24)-C(25)-C(26)	-176.79(13)
C(24)-C(25)-C(26)-C(27)	-1.2(2)
C(25)-C(26)-C(27)-C(32)	-1.0(2)
C(25)-C(26)-C(27)-C(28)	177.54(13)
C(26)-C(27)-C(28)-C(29)	-179.27(14)
C(32)-C(27)-C(28)-C(29)	-0.8(2)
C(27)-C(28)-C(29)-C(30)	-0.8(2)
C(28)-C(29)-C(30)-C(31)	1.5(2)
C(29)-C(30)-C(31)-C(32)	-0.7(2)
C(30)-C(31)-C(32)-C(33)	176.64(13)
C(30)-C(31)-C(32)-C(27)	-0.9(2)
C(26)-C(27)-C(32)-C(31)	-179.83(12)

C(28)-C(27)-C(32)-C(31)	1.62(19)
C(26)-C(27)-C(32)-C(33)	2.54(19)
C(28)-C(27)-C(32)-C(33)	-176.01(12)
C(25)-C(24)-C(33)-C(32)	-0.1(2)
C(23)-C(24)-C(33)-C(32)	178.42(12)
C(31)-C(32)-C(33)-C(24)	-179.57(13)
C(27)-C(32)-C(33)-C(24)	-2.0(2)

Symmetry transformations used to generate equivalent atoms:

9. NMR Spectra

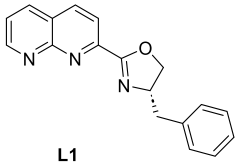

L1

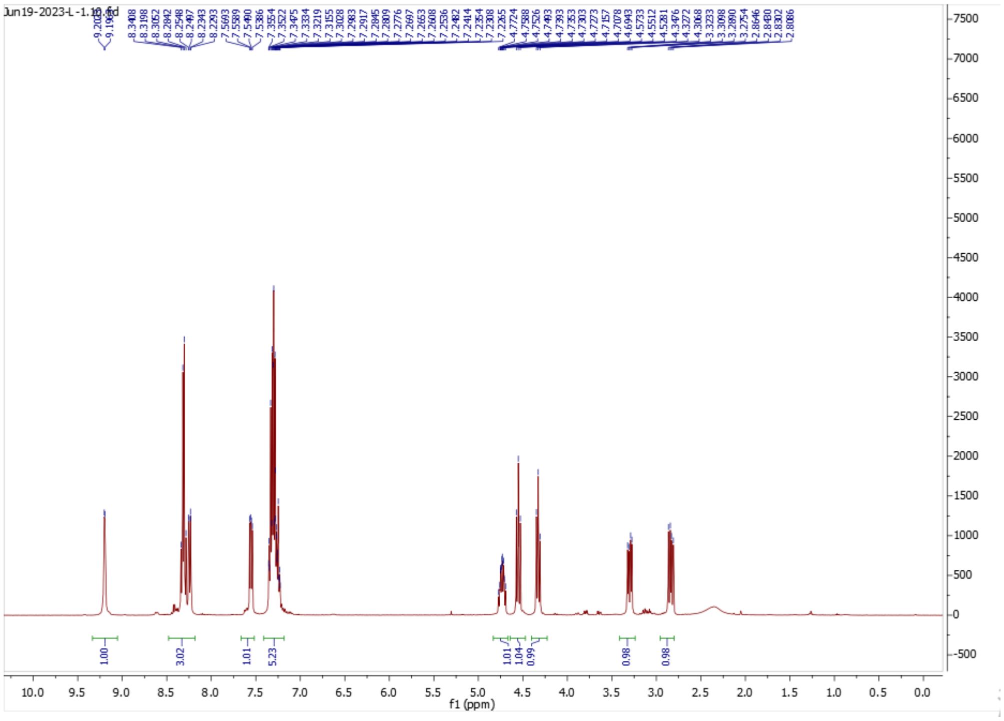

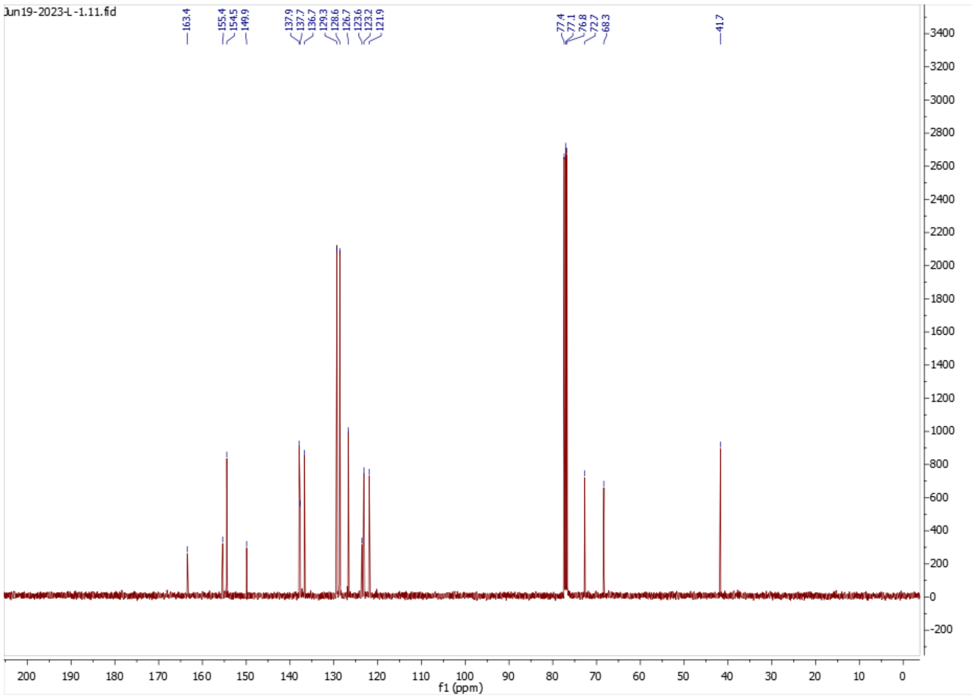

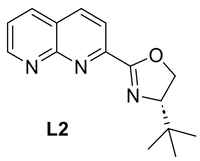

L2

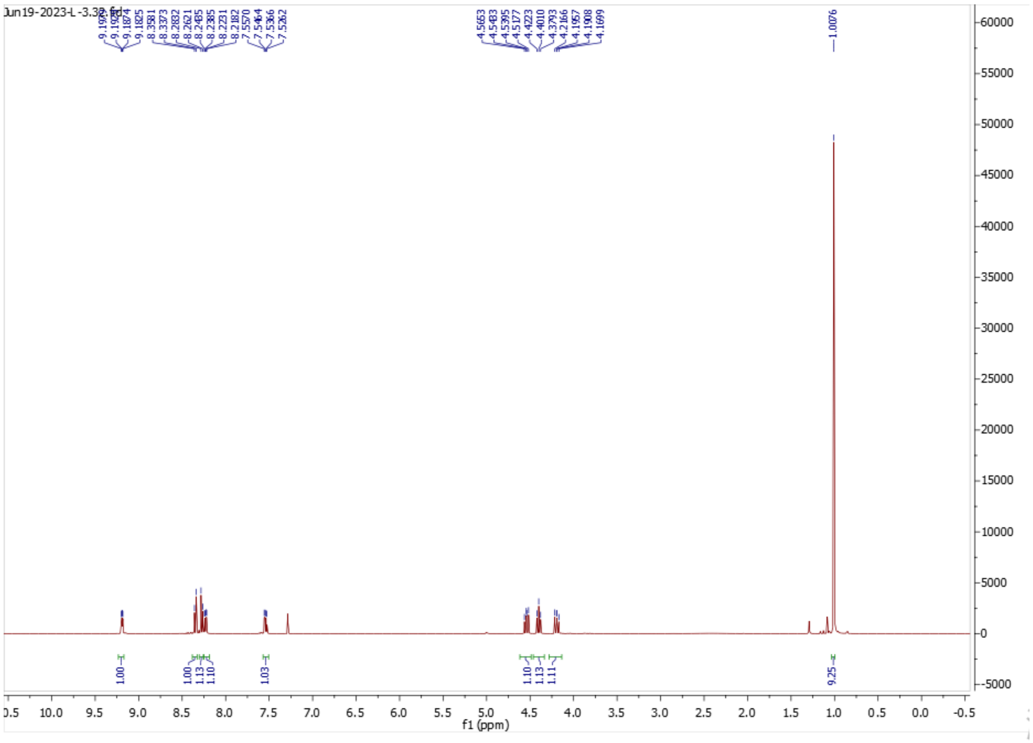

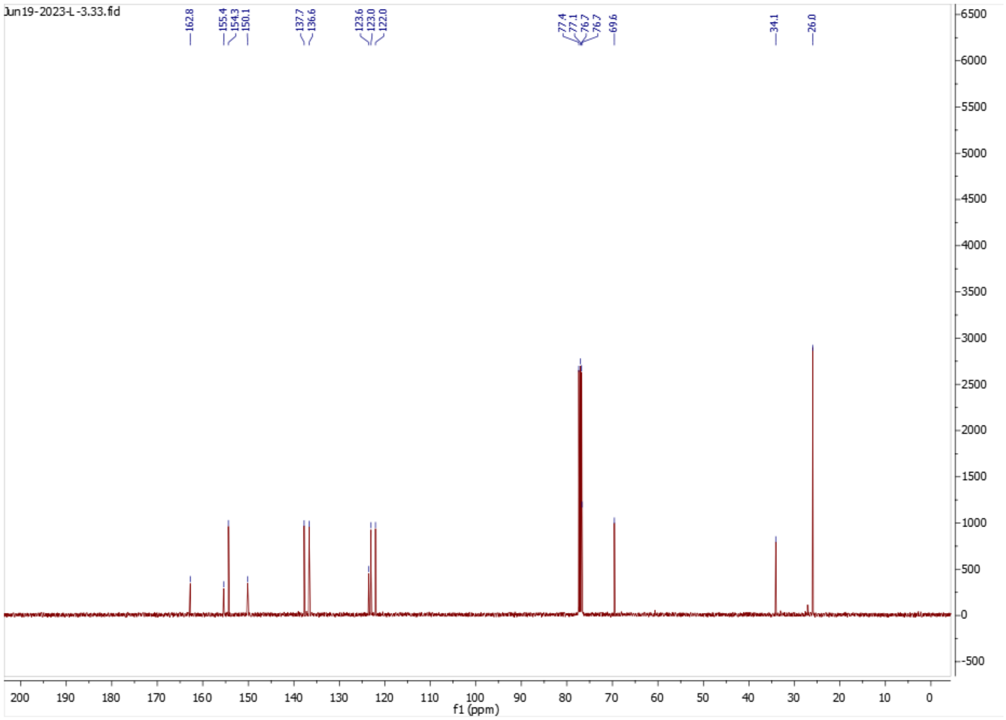

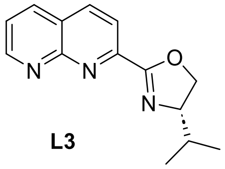
N
N
O
N

L3

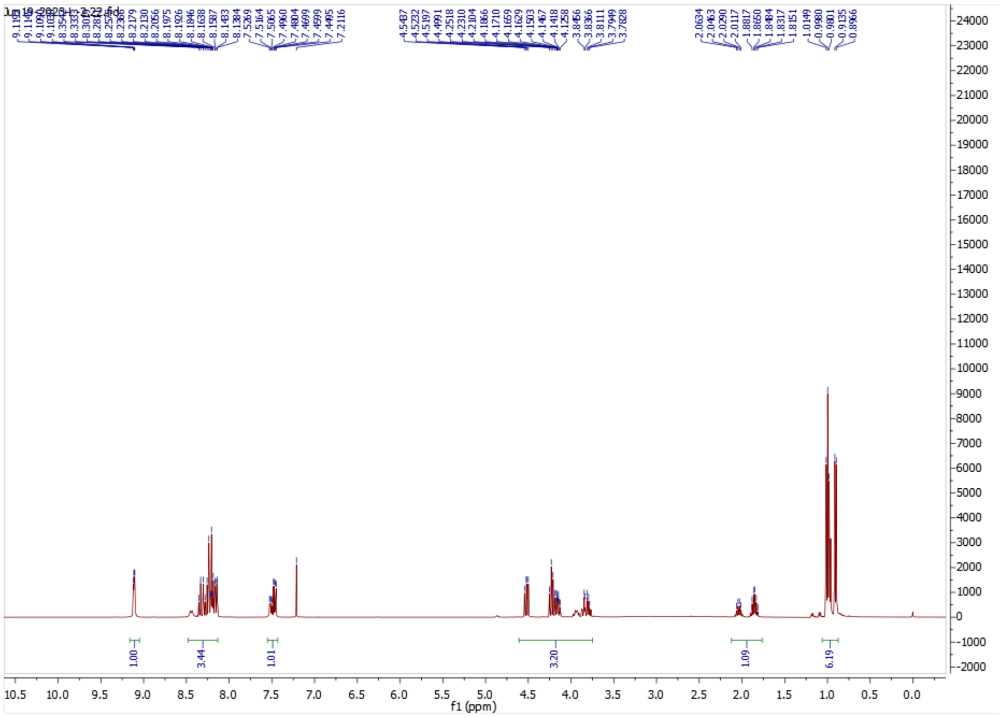
1.00
3.44
1.01
3.20
1.09
6.19
f1 (ppm)

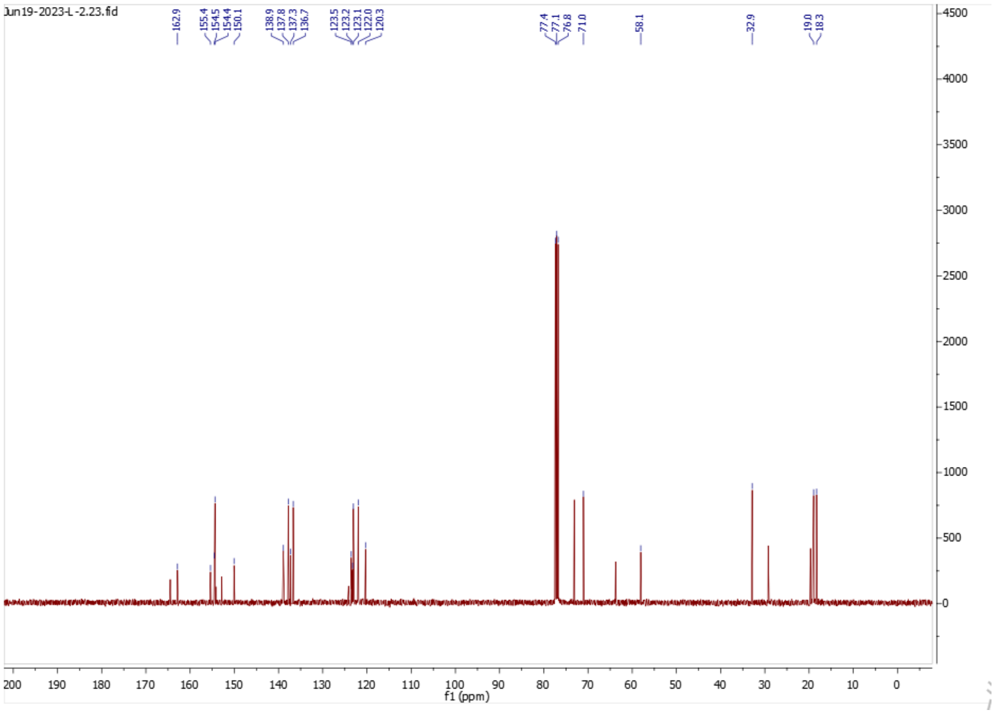
Jun19-2023-L-2.23.fid
162.9
155.4
154.5
154.4
150.1
138.9
137.8
137.3
136.7
123.5
123.2
123.1
122.0
120.3
77.4
77.1
76.8
71.0
58.1
32.9
19.0
18.3
f1 (ppm)

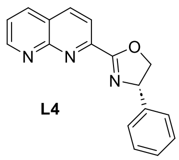

N
N
O
N

L4

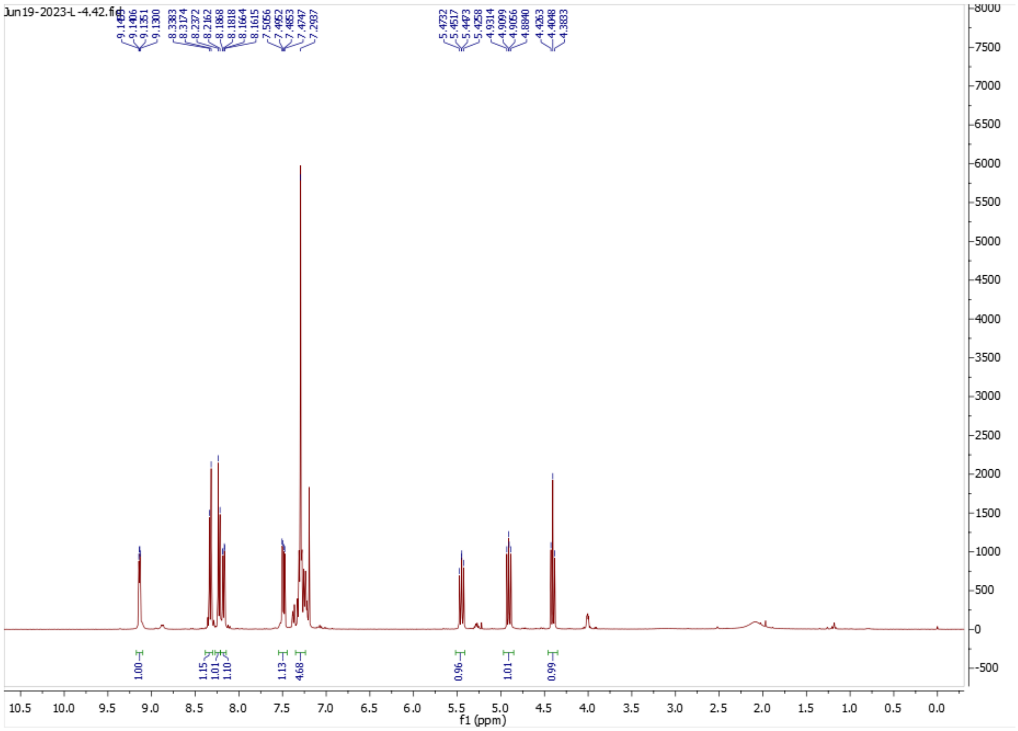

Jun19-2023-L-4.42.fid
9.1406
9.1351
9.1300
8.3383
8.3174
8.2372
8.2162
8.1868
8.1818
8.1664
8.1615
7.5056
7.4952
7.4853
7.4747
7.2937
5.4732
5.4517
5.4473
5.4258
4.9314
4.9099
4.9056
4.8840
4.4263
4.4048
4.3833
1.00
1.15
1.01
1.10
1.13
4.68
0.96
1.01
0.99
f1 (ppm)

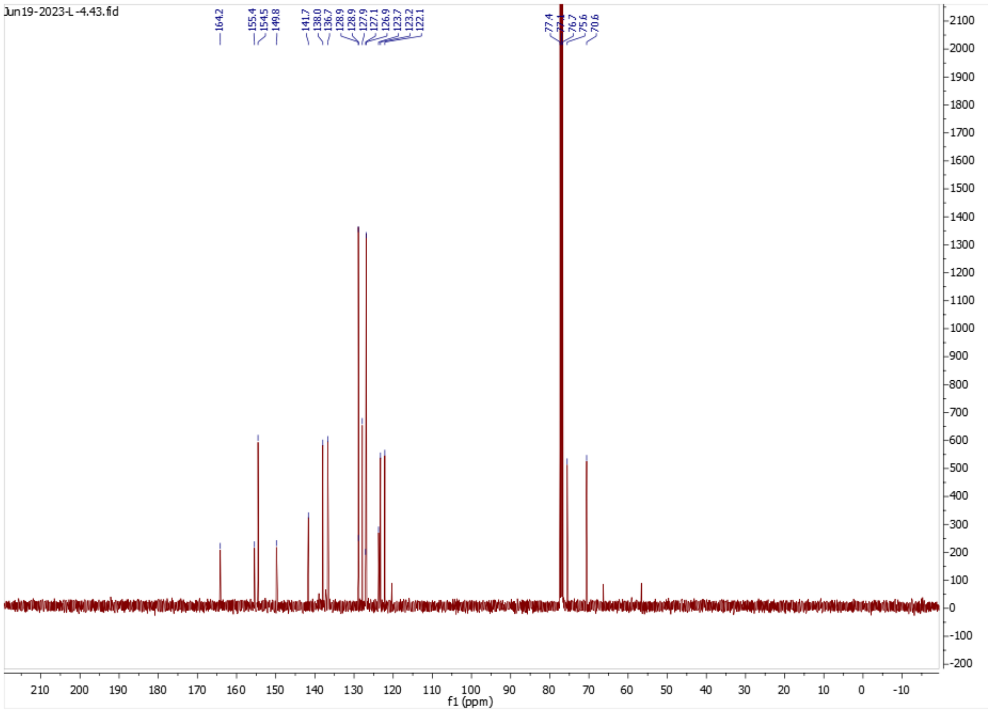

Jun19-2023-L-4.43.fid
164.2
155.4
154.5
149.8
141.7
138.0
136.7
128.9
127.9
127.1
126.9
123.7
123.2
122.1
77.4
76.7
75.6
70.6
f1 (ppm)

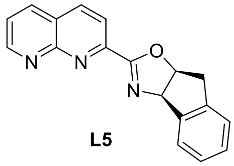

L5

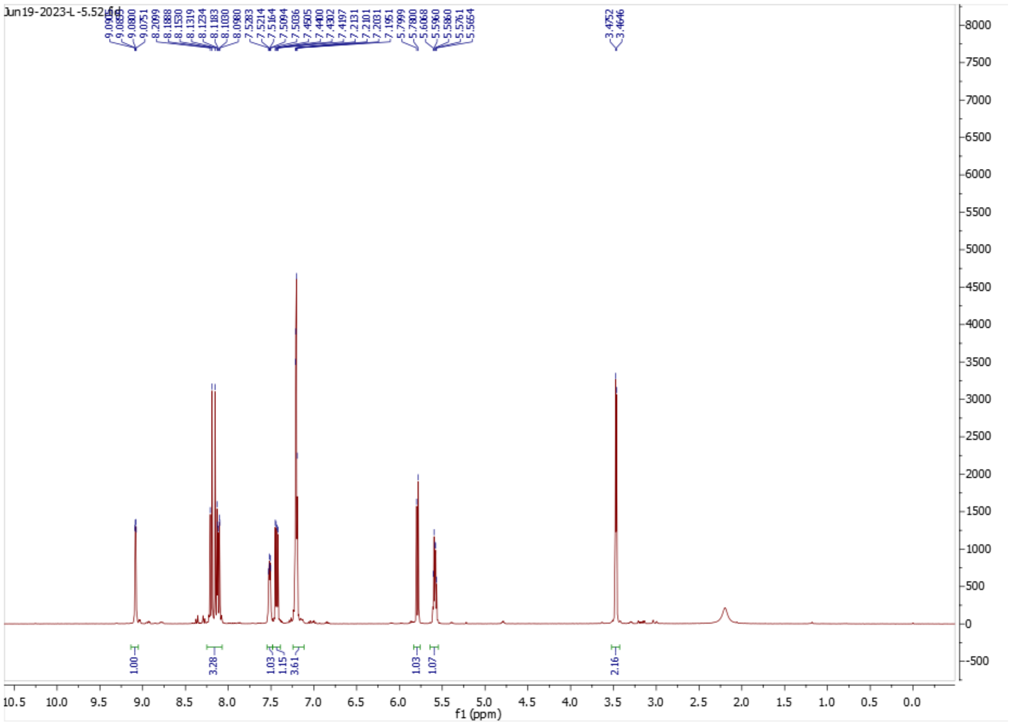

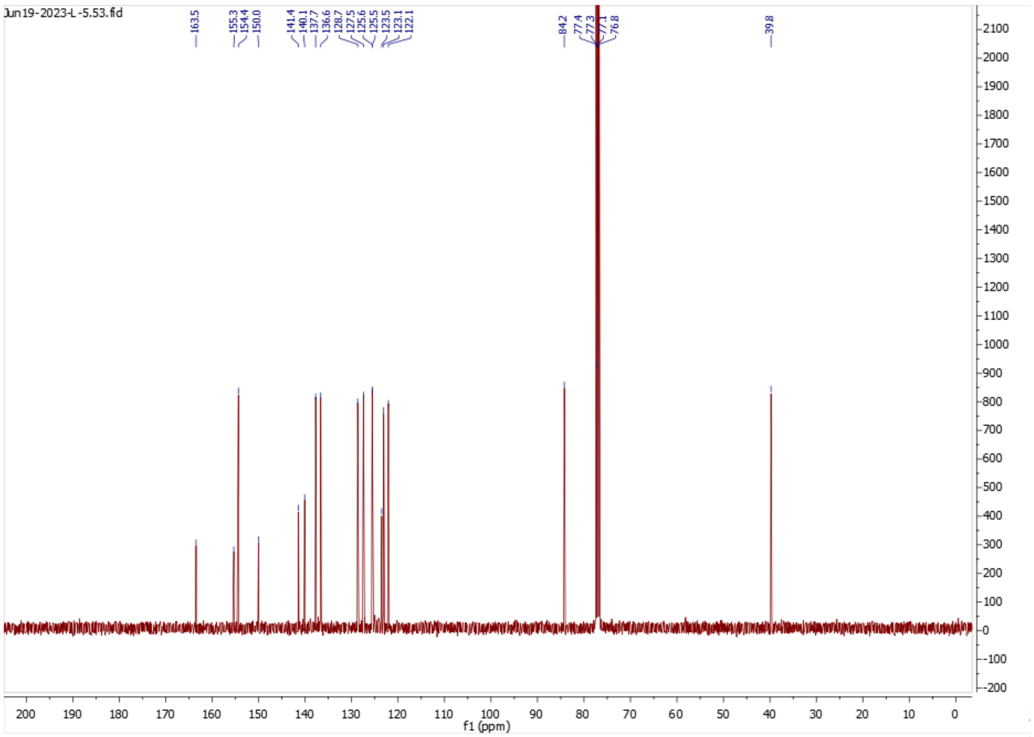

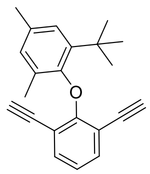
O

1a

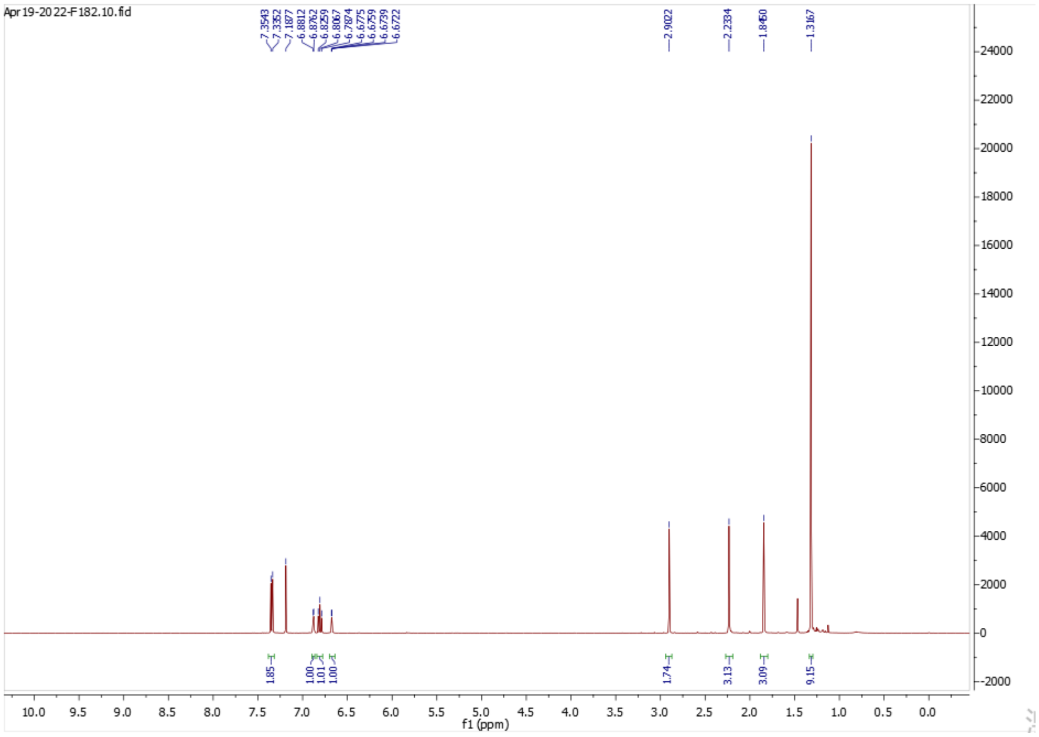
Apr19-2022-F182.10.fid
7.3543
7.3352
7.1877
6.8812
6.8762
6.8259
6.8067
6.7874
6.6775
6.6759
6.6739
6.6722
2.9022
2.2334
1.8450
1.3167
1.85
1.00
1.01
1.00
1.74
3.13
3.09
9.15
f1 (ppm)

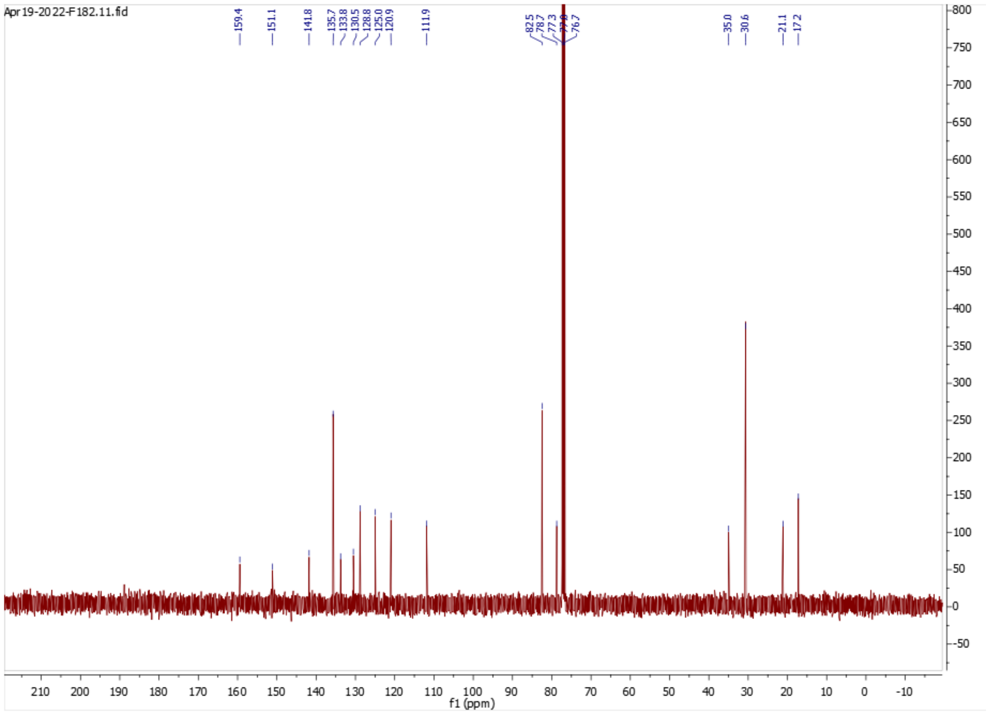
Apr19-2022-F182.11.fid
159.4
151.1
141.8
135.7
133.8
130.5
128.8
125.0
120.9
111.9
82.5
78.7
77.3
77.0
76.7
35.0
30.6
21.1
17.2
f1 (ppm)

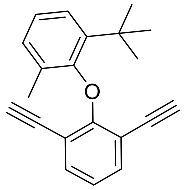

1b

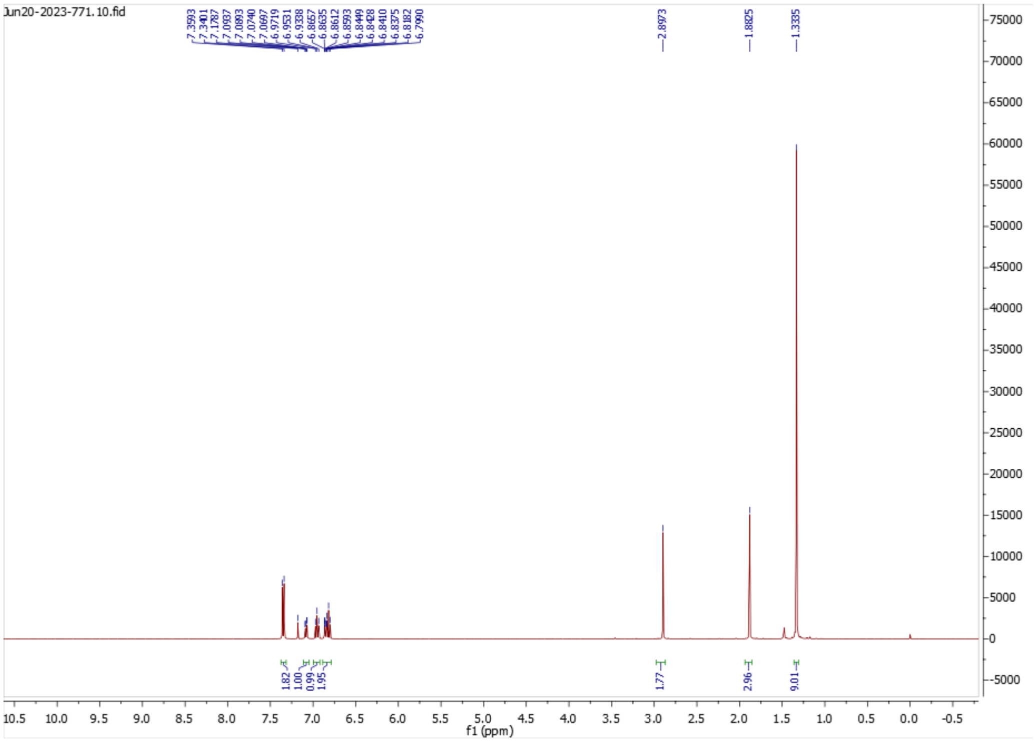

Jun20-2023-771.10.fid
7.3593
7.3401
7.1787
7.0937
7.0893
7.0740
7.0697
6.9719
6.9531
6.9338
6.8657
6.8635
6.8612
6.8593
6.8449
6.8428
6.8410
6.8375
6.8182
6.7990
2.8973
1.8825
1.3335
1.82
1.00
0.99
1.95
1.77
2.96
9.01
f1 (ppm)

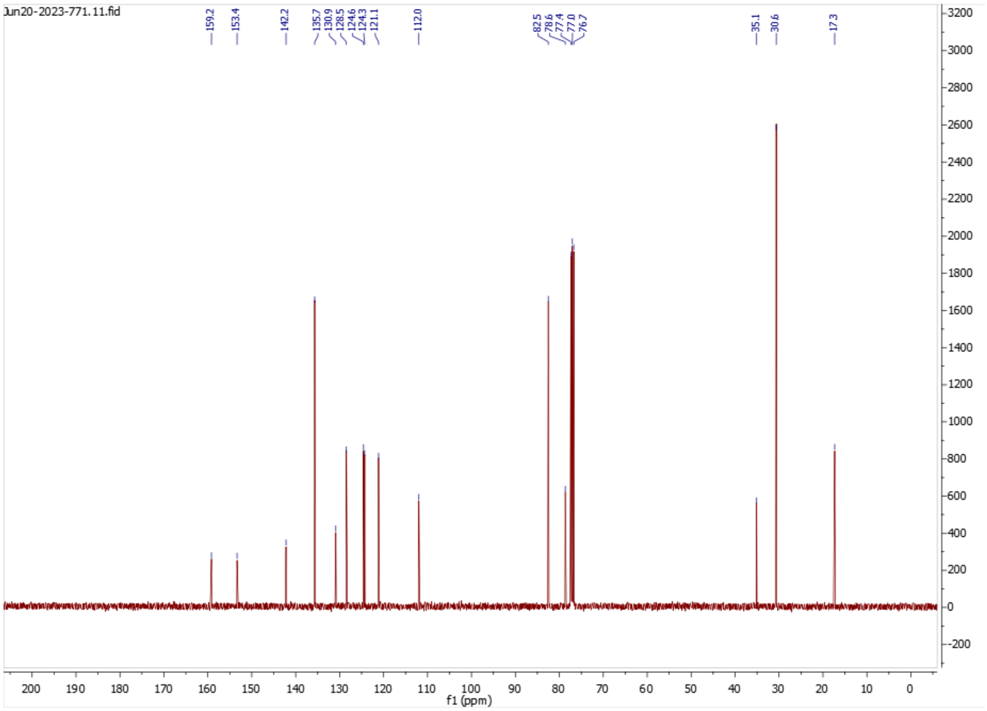

Jun20-2023-771.11.fid
159.2
153.4
142.2
135.7
130.9
128.5
124.6
124.3
121.1
112.0
82.5
78.6
77.4
77.0
76.7
35.1
30.6
17.3
f1 (ppm)

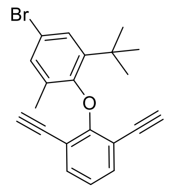

Br
O

1c

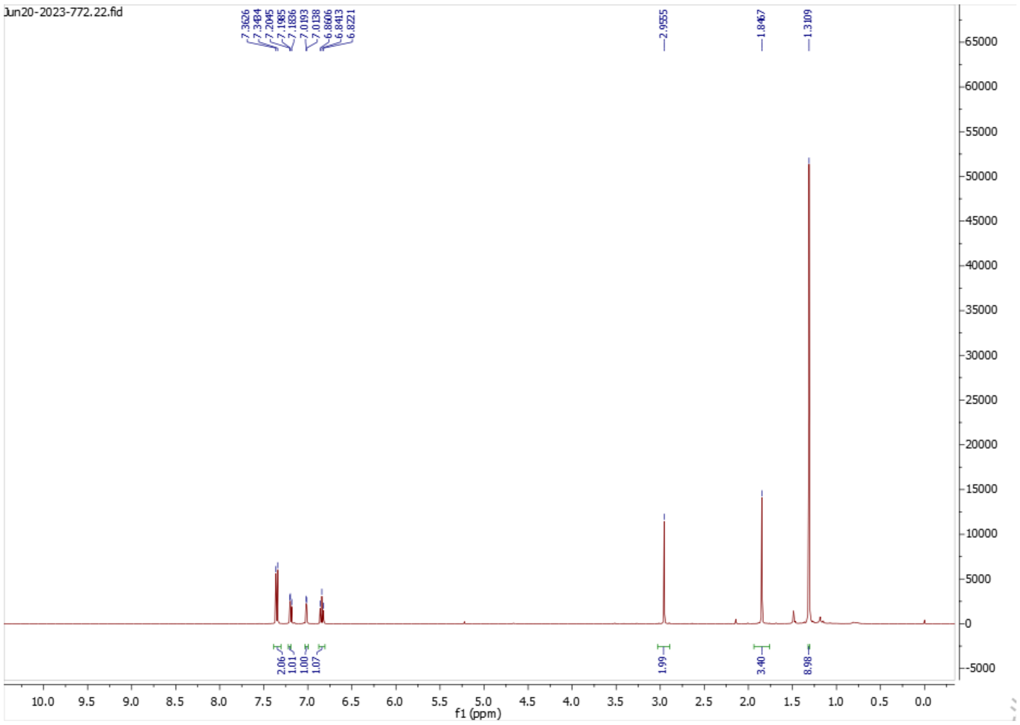

Jun20-2023-772.22.fid
7.3626
7.3434
7.2045
7.1985
7.1836
7.0193
7.0138
6.8606
6.8413
6.8221
2.9555
1.8467
1.3109
2.06
1.01
1.00
1.07
1.99
3.40
8.98
f1 (ppm)

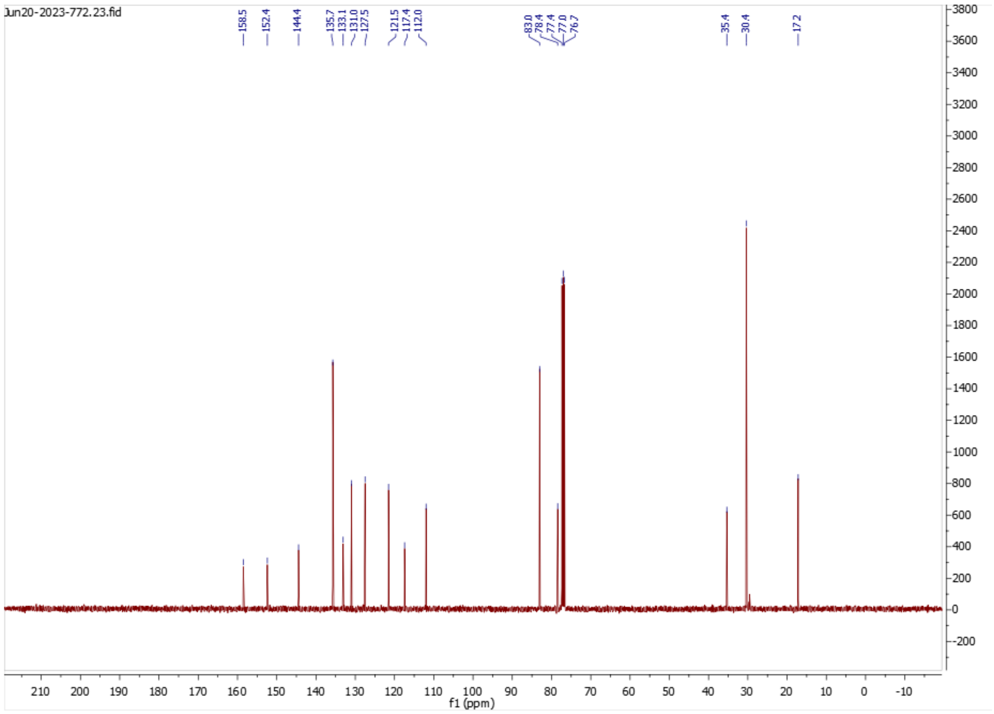

Jun20-2023-772.23.fid
158.5
152.4
144.4
135.7
133.1
131.0
127.5
121.5
117.4
112.0
83.0
78.4
77.4
77.0
76.7
35.4
30.4
17.2
f1 (ppm)

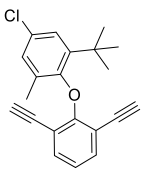

1d

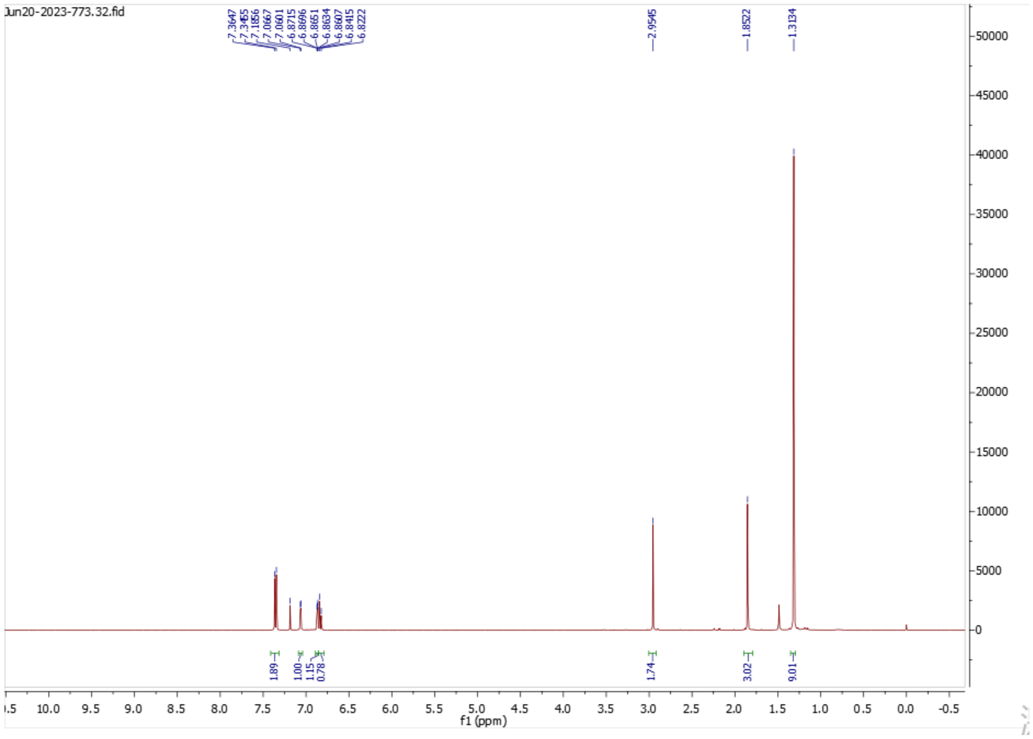
Jun20-2023-773.32.fid
7.3647
7.3455
7.1856
7.0667
7.0601
6.8715
6.8696
6.8651
6.8634
6.8607
6.8415
6.8222
2.9545
1.8522
1.3134
1.89
1.00
1.15
0.78
1.74
3.02
9.01
f1 (ppm)

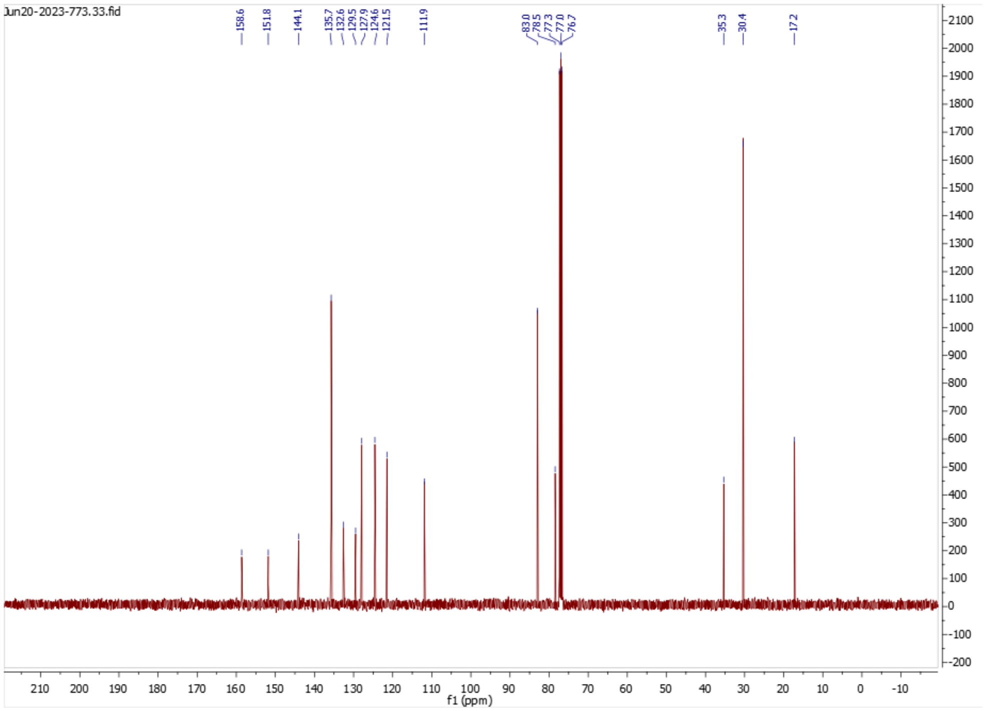
Jun20-2023-773.33.fid
158.6
151.8
144.1
135.7
132.6
129.5
127.9
124.6
121.5
111.9
83.0
78.5
77.3
77.0
76.7
35.3
30.4
17.2
f1 (ppm)

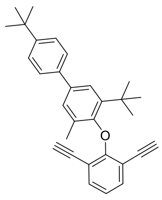

1e

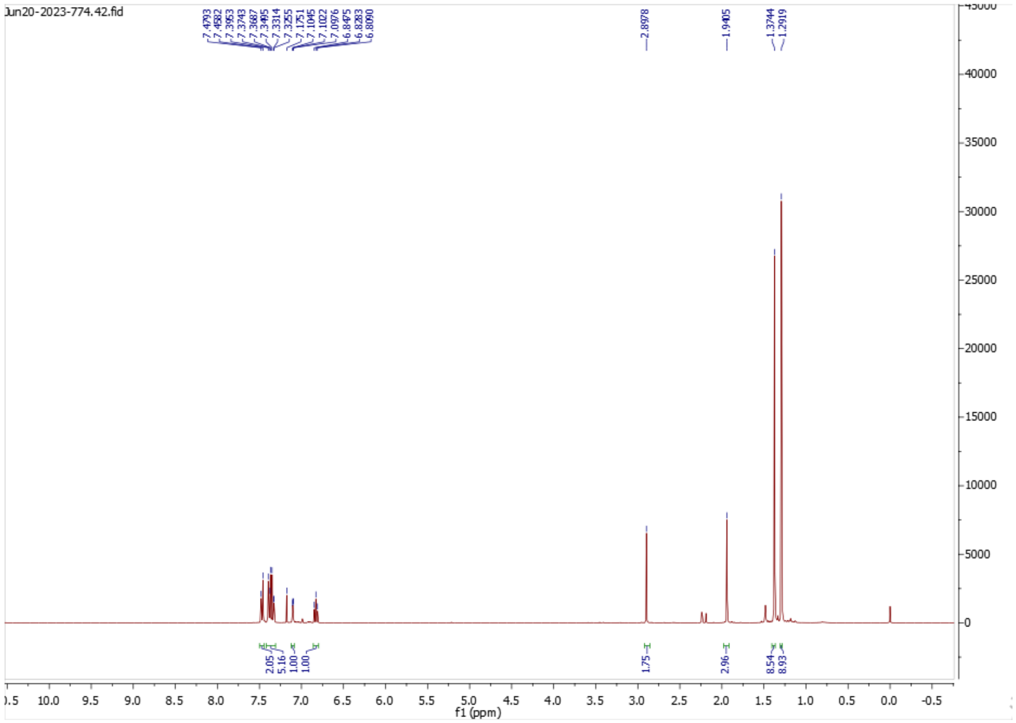

Jun20-2023-774.42.fid
7.4793
7.4582
7.3953
7.3743
7.3687
7.3495
7.3314
7.3255
7.1251
7.1045
7.1022
7.0976
6.8475
6.8283
6.8090
2.8978
1.9405
1.3744
1.2919
2.05
5.16
1.00
1.00
1.75
2.96
8.54
8.93
f1 (ppm)

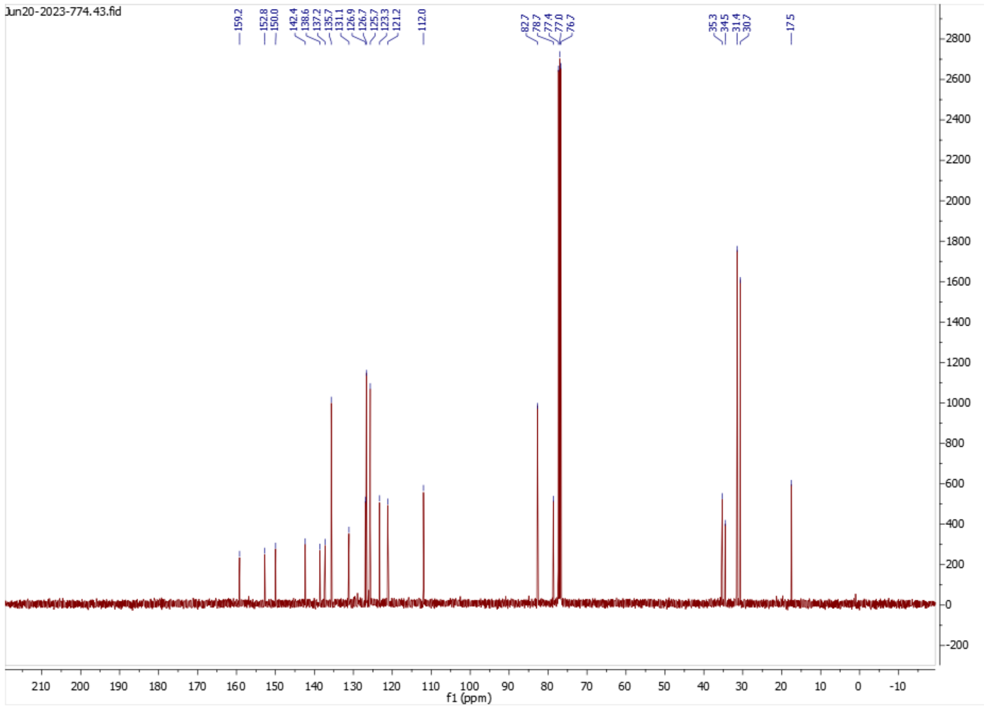

Jun20-2023-774.43.fid
159.2
152.8
150.0
142.4
138.6
137.2
135.7
131.1
126.9
126.7
125.7
123.3
121.2
112.0
82.7
78.7
77.4
77.0
76.7
35.3
34.5
31.4
30.7
17.5
f1 (ppm)

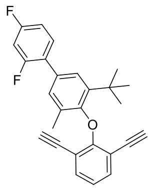
F
F
O

1f

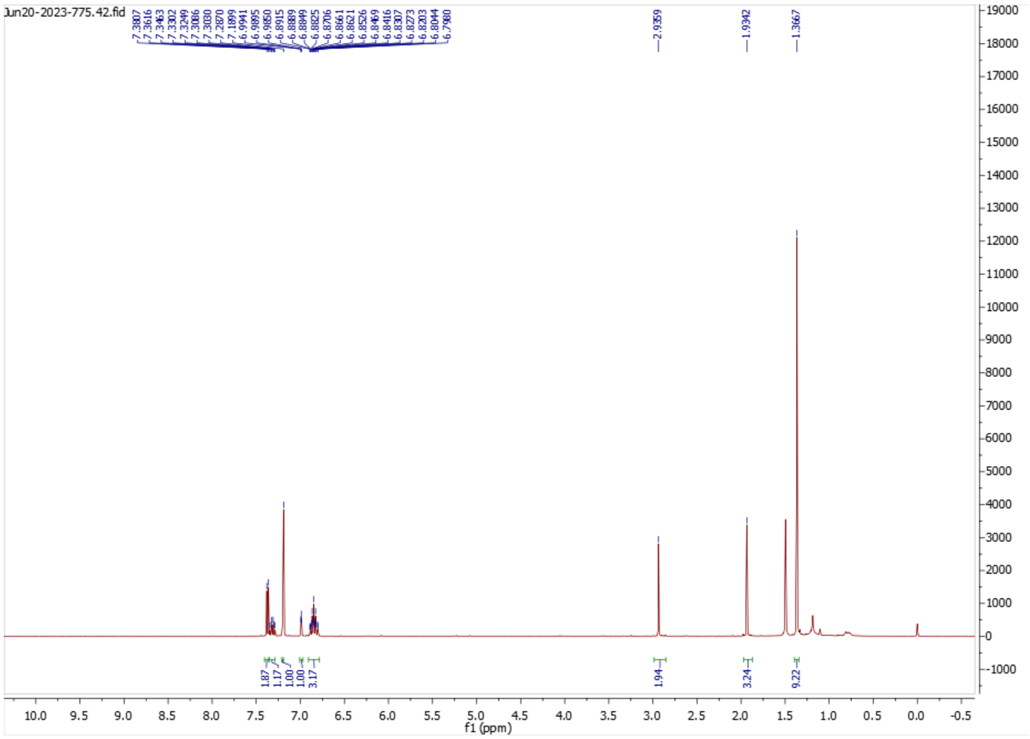
Jun20-2023-775.42.fid
7.3807
7.3616
7.3463
7.3302
7.3249
7.3086
7.3030
7.2870
7.1899
6.9941
6.9895
6.9850
6.8915
6.8889
6.8849
6.8825
6.8706
6.8661
6.8621
6.8526
6.8469
6.8416
6.8307
6.8273
6.8203
6.8044
6.7980
2.9359
1.9342
1.3667
1.87
1.17
1.00
1.00
3.17
1.94
3.24
9.22
f1 (ppm)

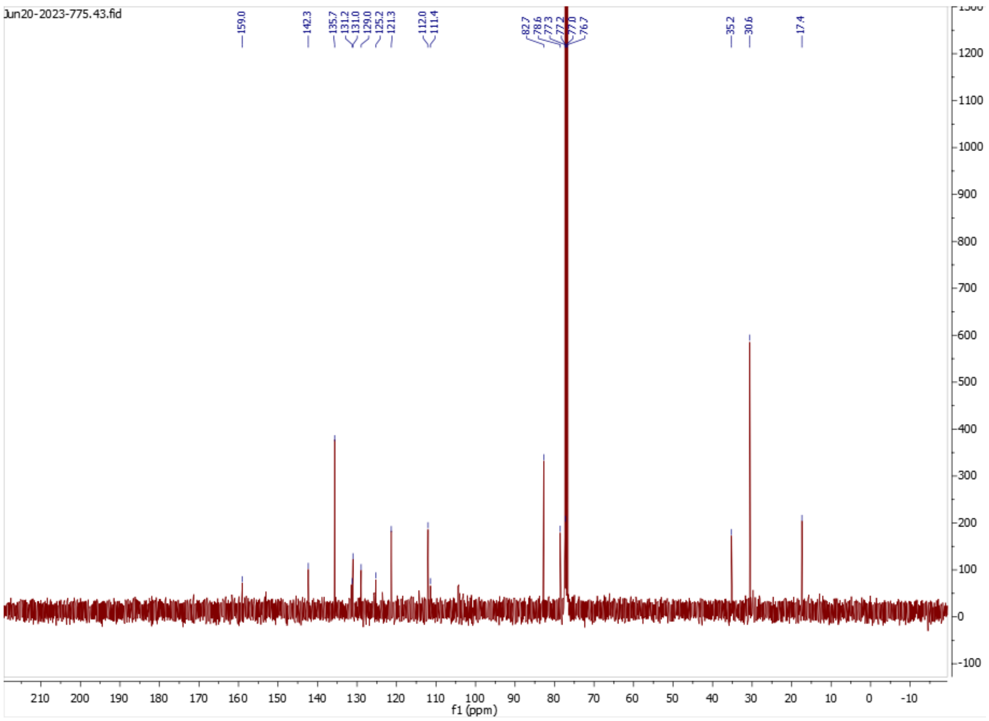
Jun20-2023-775.43.fid
159.0
142.3
135.7
131.2
131.0
129.0
125.2
121.3
112.0
111.4
82.7
78.6
77.3
77.2
77.0
76.7
35.2
30.6
17.4
f1 (ppm)

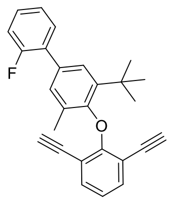
F
O

1g

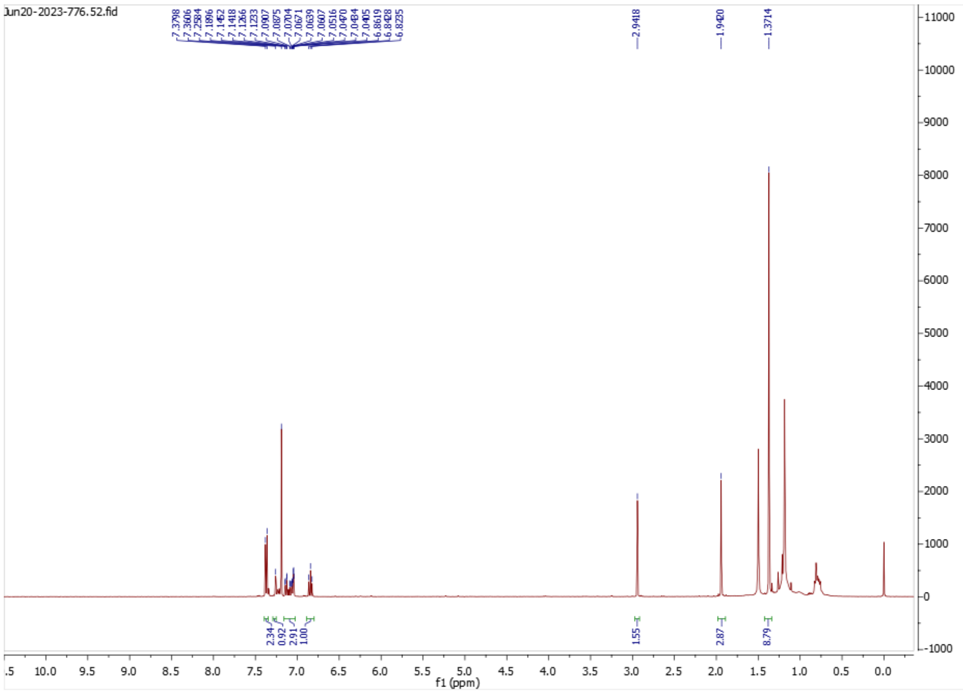
Jun20-2023-776.52.fid
7.3798
7.3606
7.2584
7.1896
7.1452
7.1418
7.1266
7.1233
7.0907
7.0875
7.0704
7.0671
7.0639
7.0607
7.0516
7.0470
7.0434
7.0405
6.8619
6.8428
6.8235
2.9418
1.9420
1.3714
2.34
0.92
2.91
1.00
1.55
2.87
8.79
f1 (ppm)

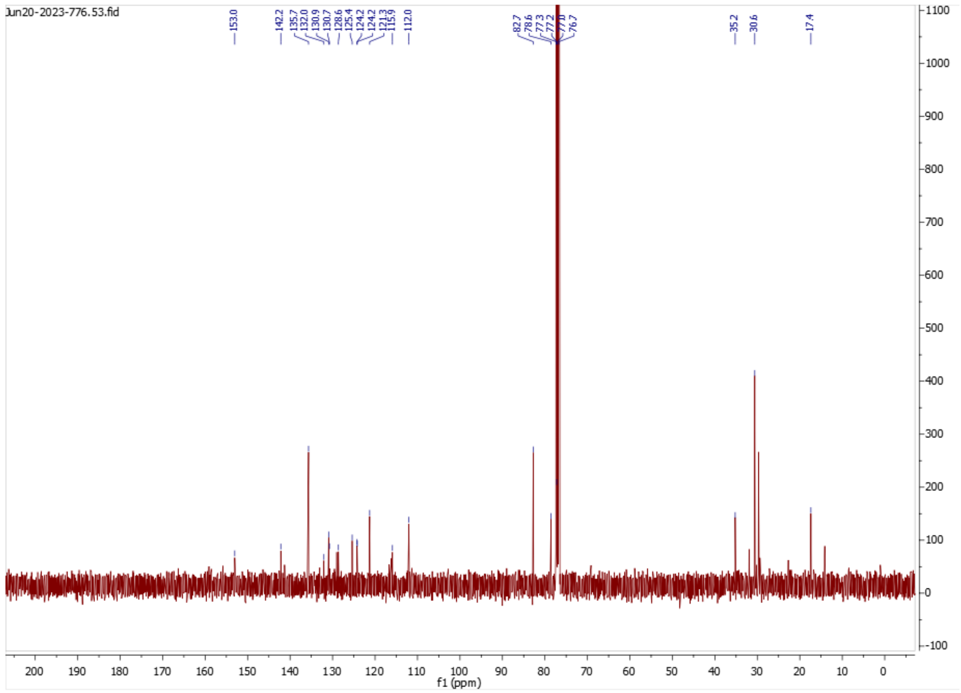
Jun20-2023-776.53.fid
153.0
142.2
135.7
132.0
130.9
130.7
128.6
125.4
124.2
124.2
121.3
115.9
112.0
82.7
78.6
77.3
77.2
77.0
76.7
35.2
30.6
17.4
f1 (ppm)

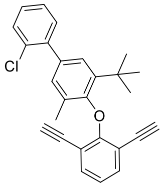

1h

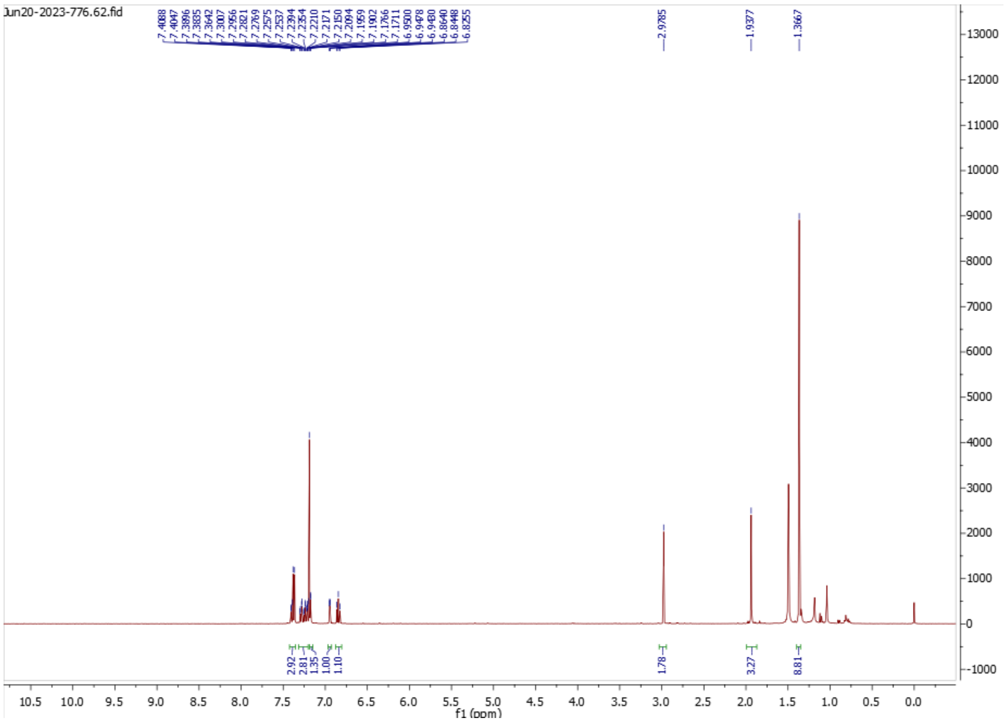

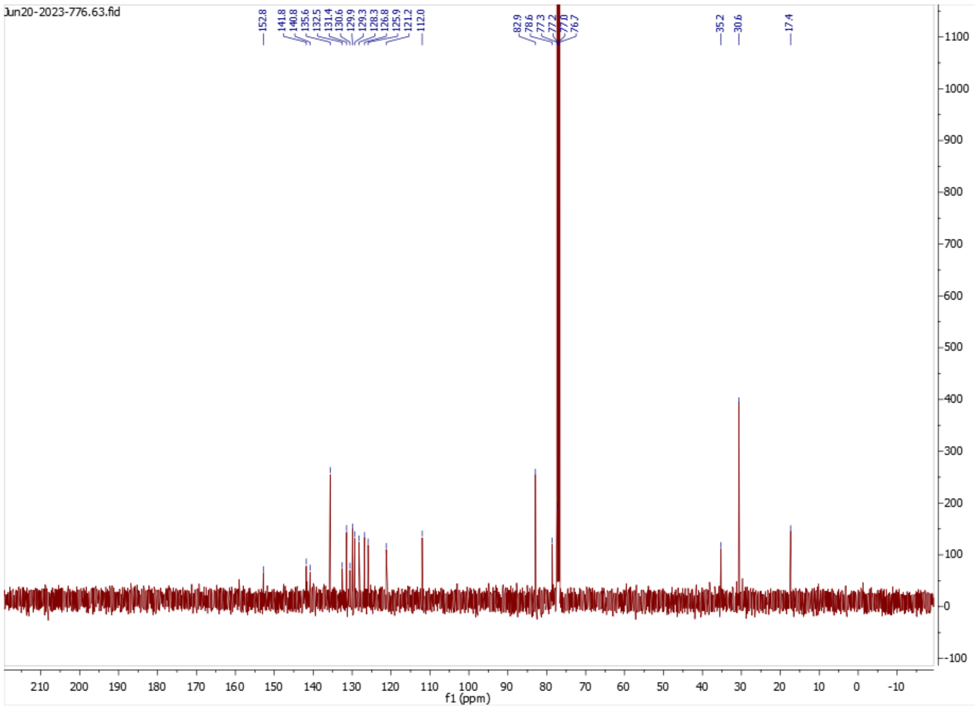

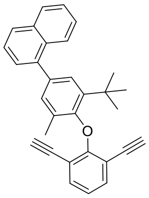

1i

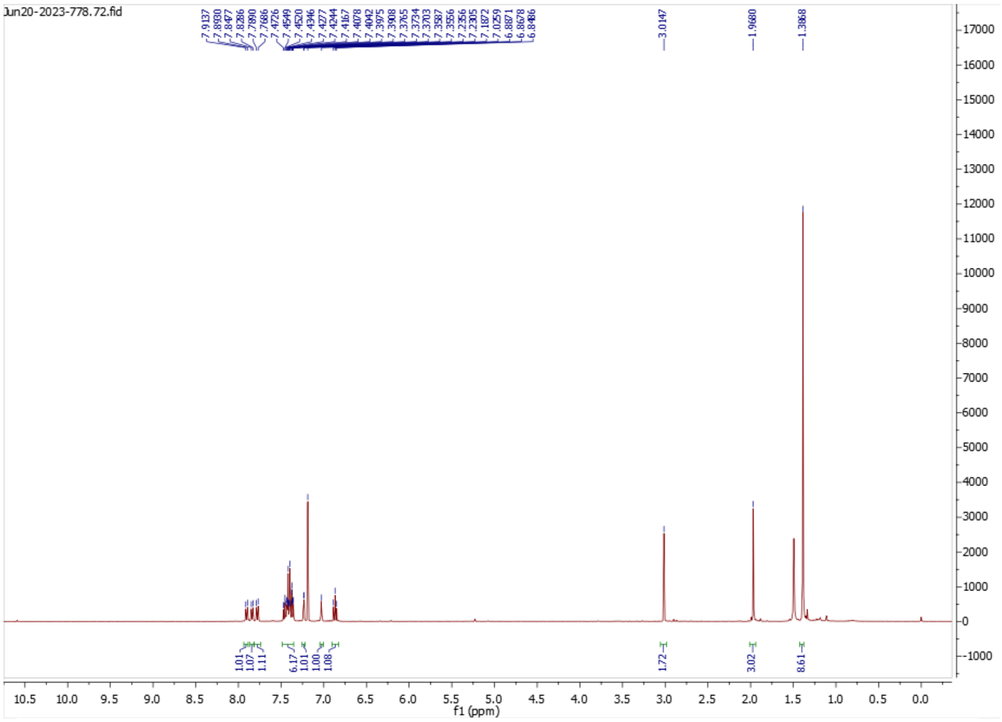
Jun20-2023-778.72.fid
7.9137 7.8930 7.8477 7.8286 7.7890 7.7686 7.4726 7.4549 7.4520 7.4346 7.4277 7.4244 7.4167 7.4078 7.4042 7.3975 7.3908 7.3765 7.3734 7.3703 7.3587 7.3556 7.2356 7.2305 7.1872 7.0259 6.8871 6.8678 6.8486
3.0147
1.9680
1.3868
1.01 1.07 1.11 6.17 1.01 1.00 1.08 1.72 3.02 8.61
f1 (ppm)

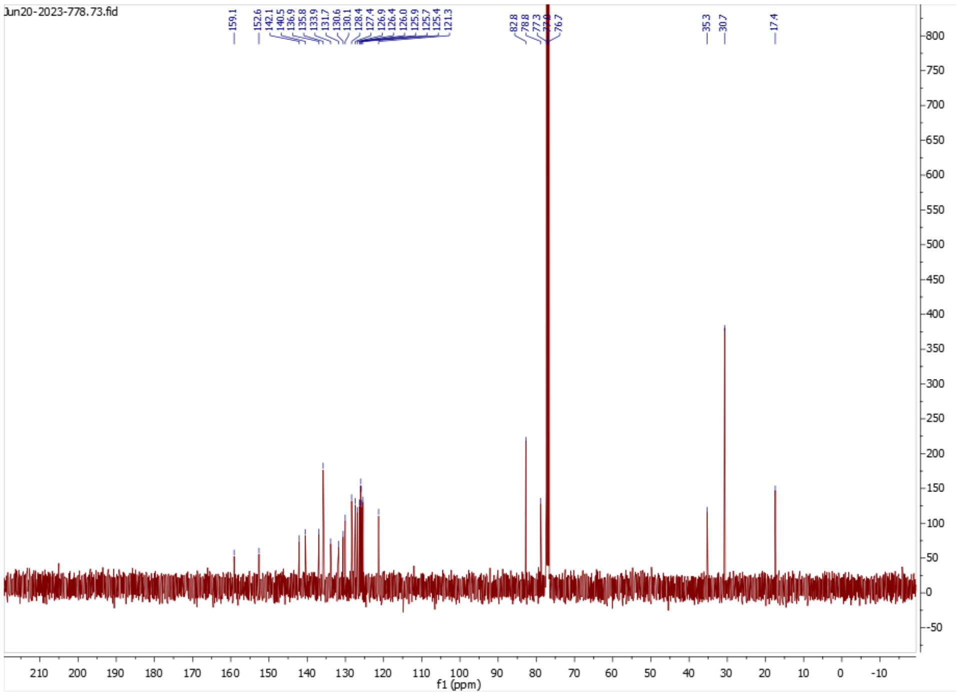
Jun20-2023-778.73.fid
159.1 152.6 142.1 140.5 136.9 135.8 133.9 131.7 130.6 130.1 128.4 127.4 126.9 126.4 126.0 125.9 125.7 125.4 121.3
82.8 78.8 77.3 77.0 76.7
35.3 30.7 17.4
f1 (ppm)

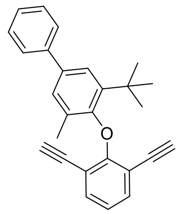

1j

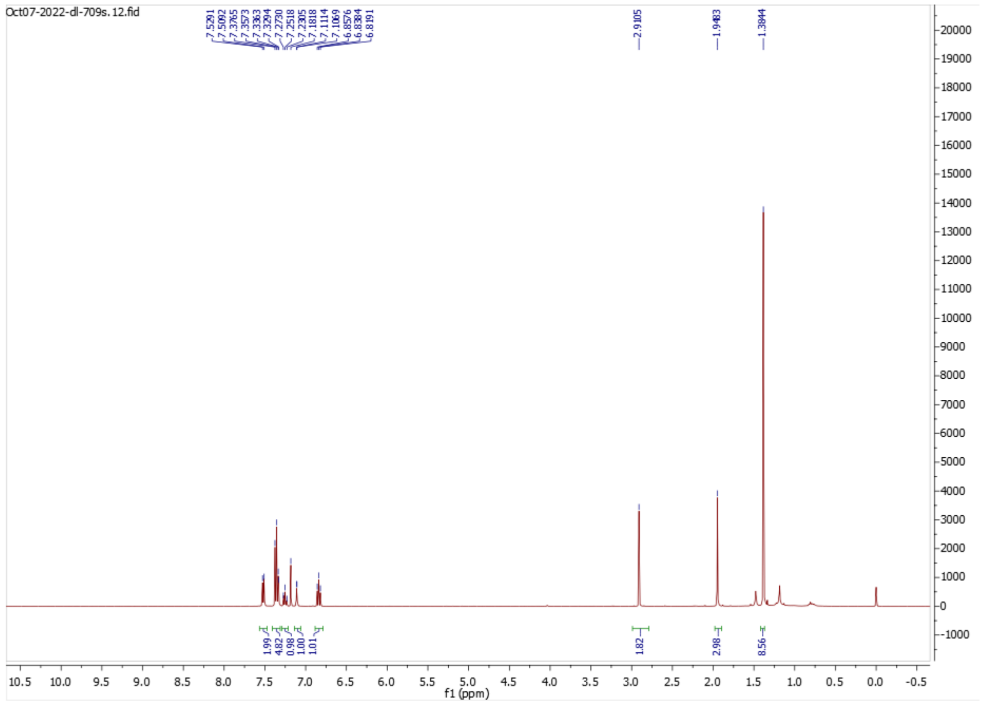

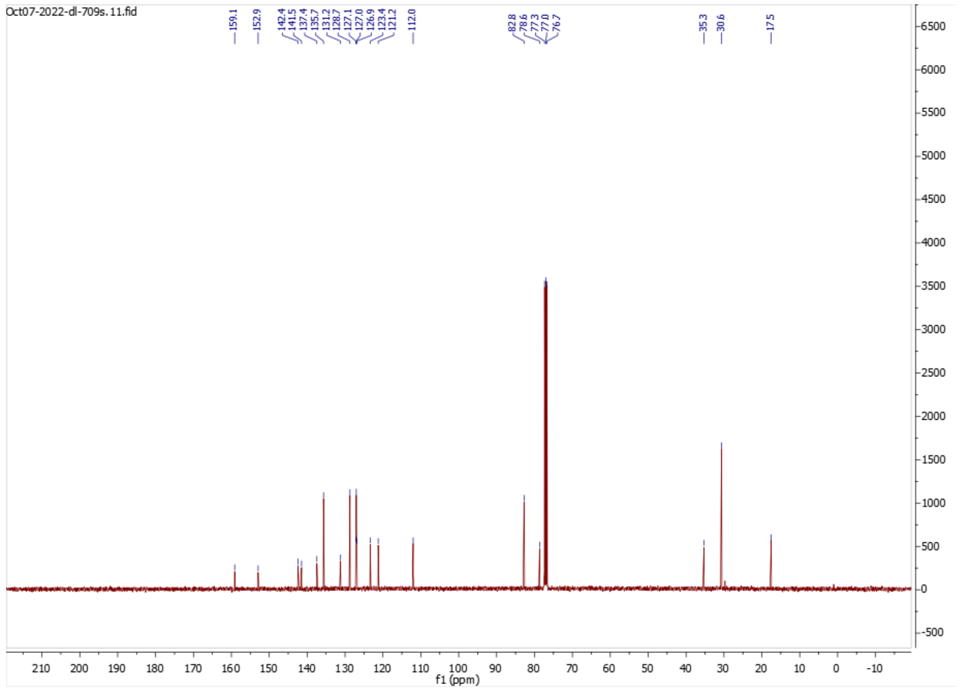

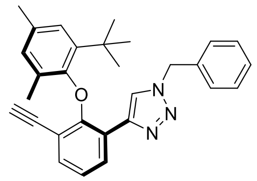

3a

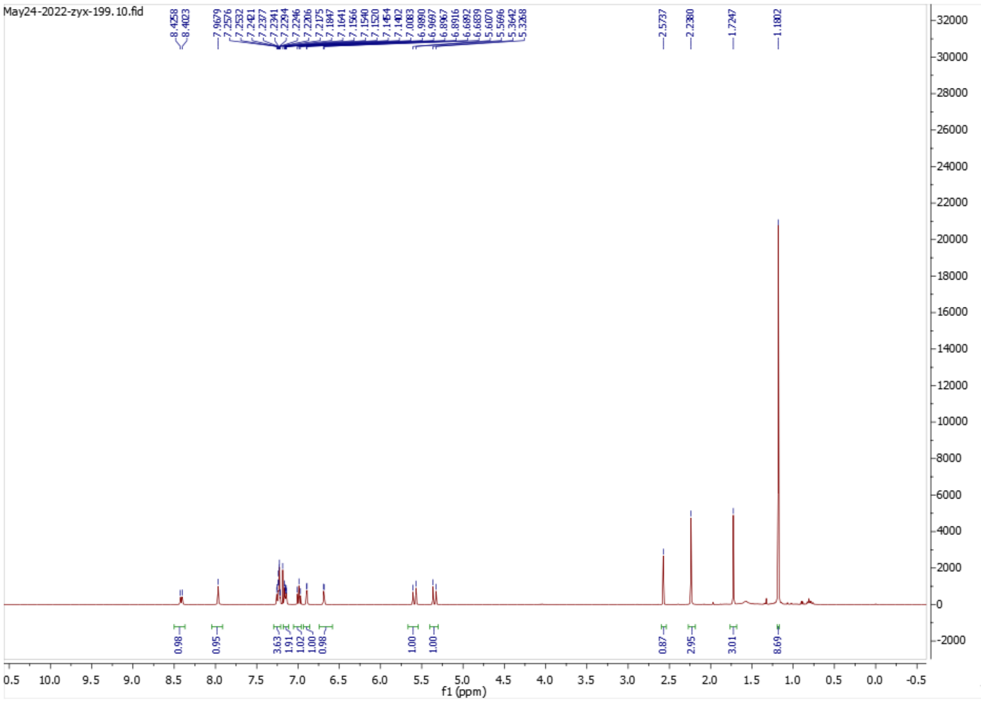

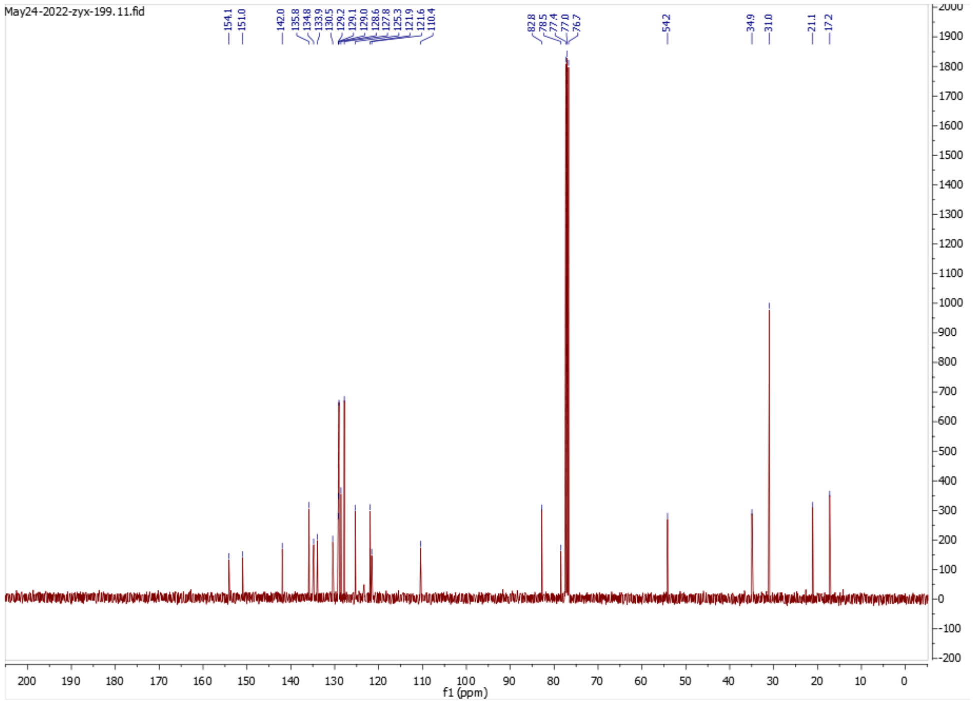

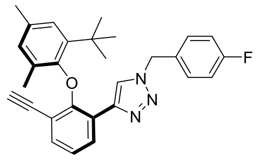

3b

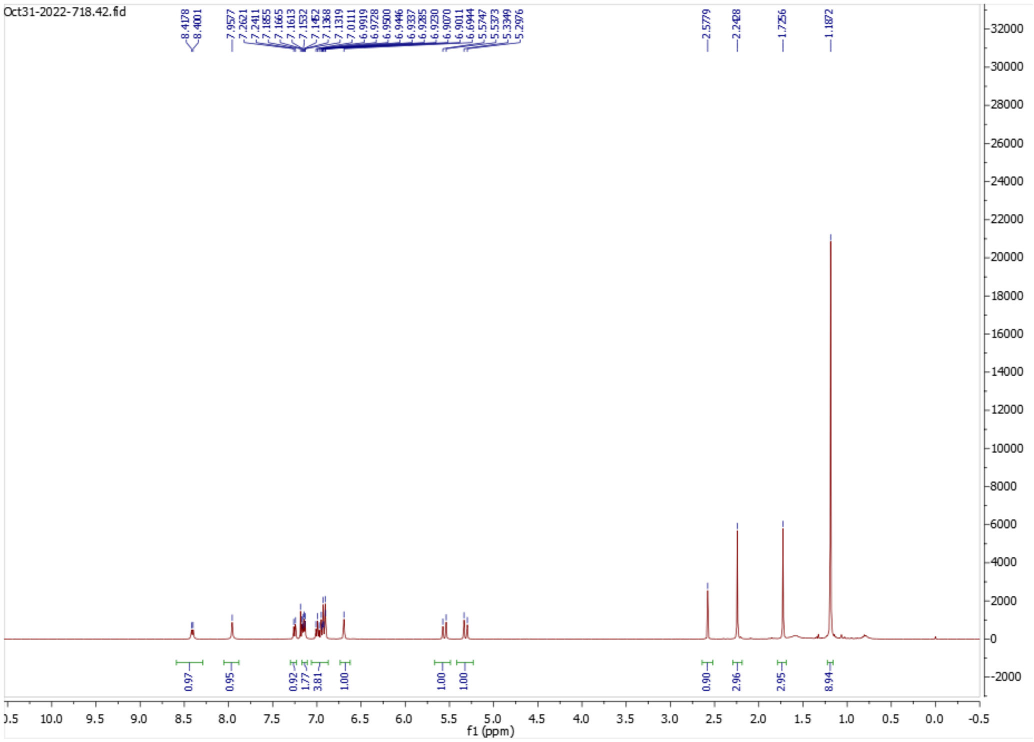

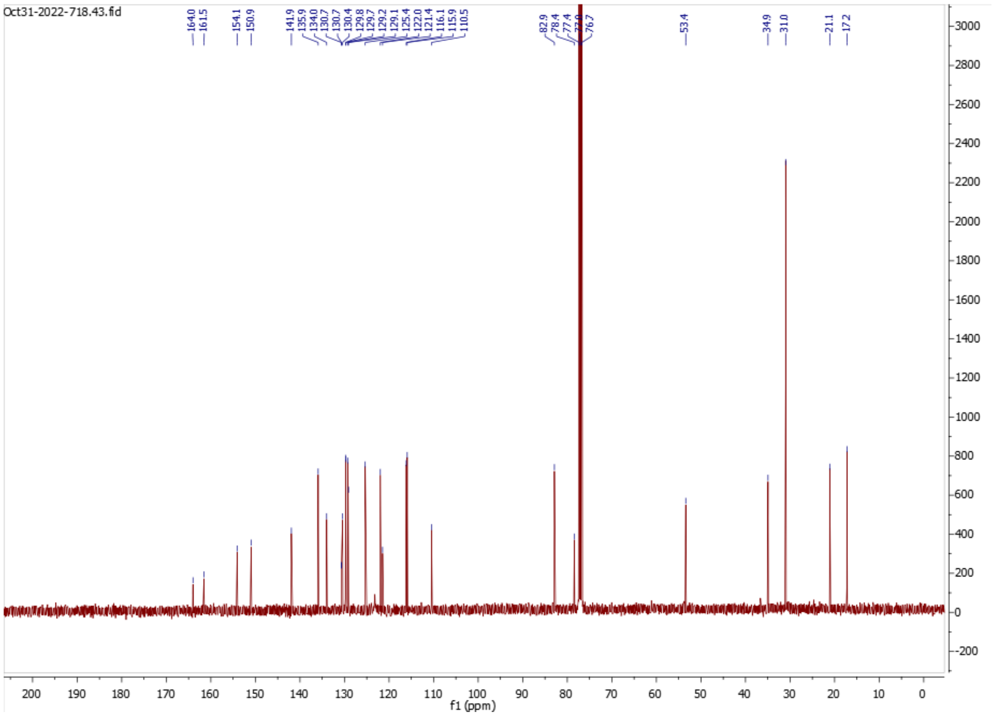

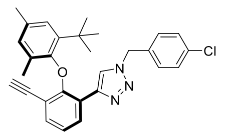

3c

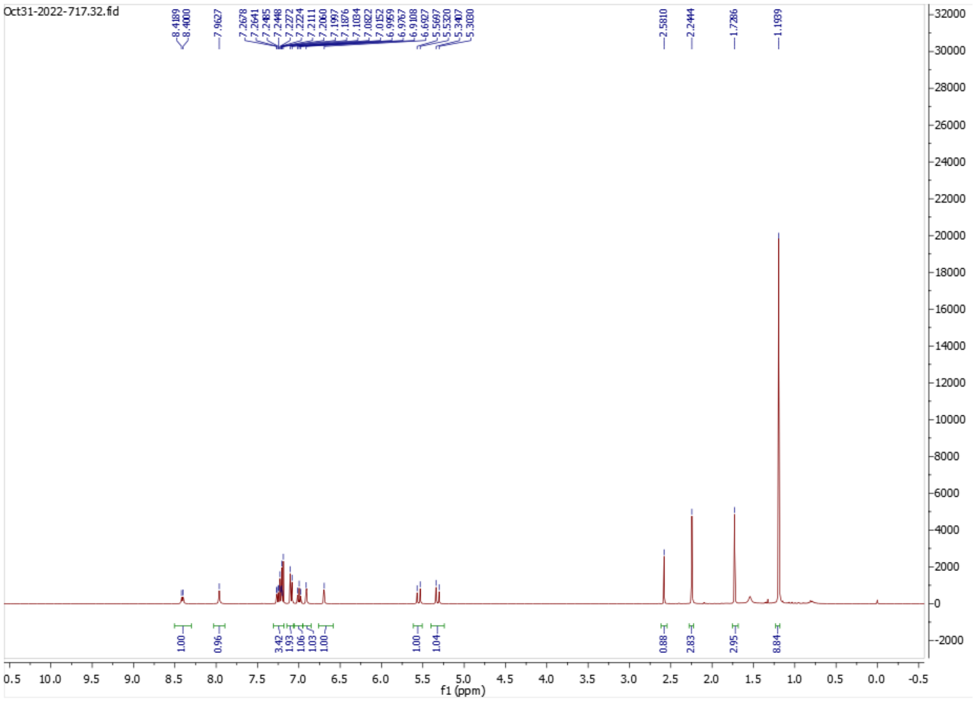

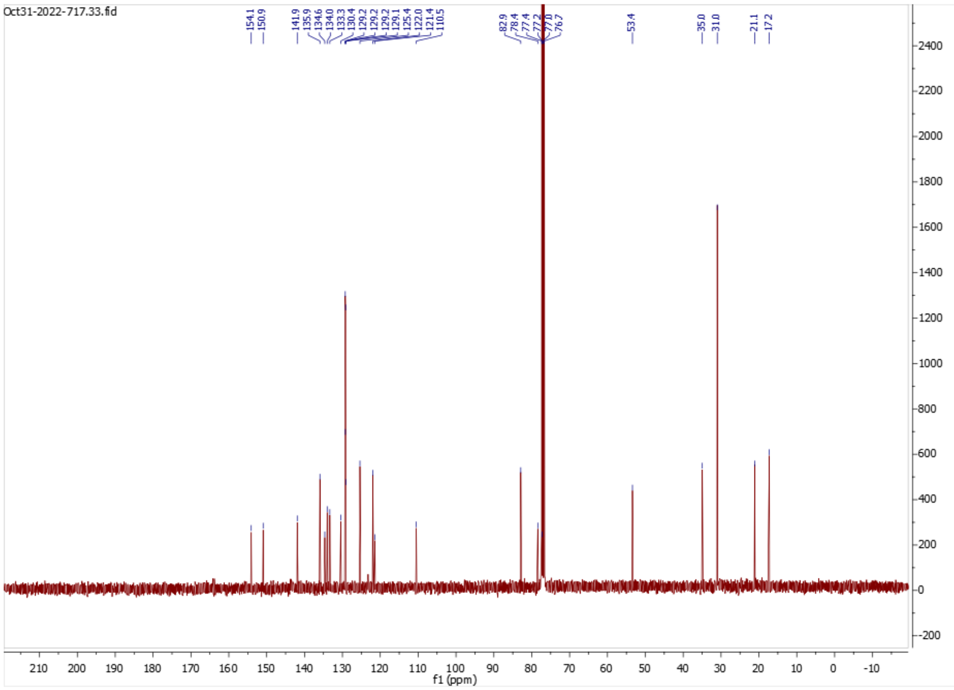

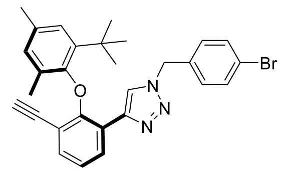

3d

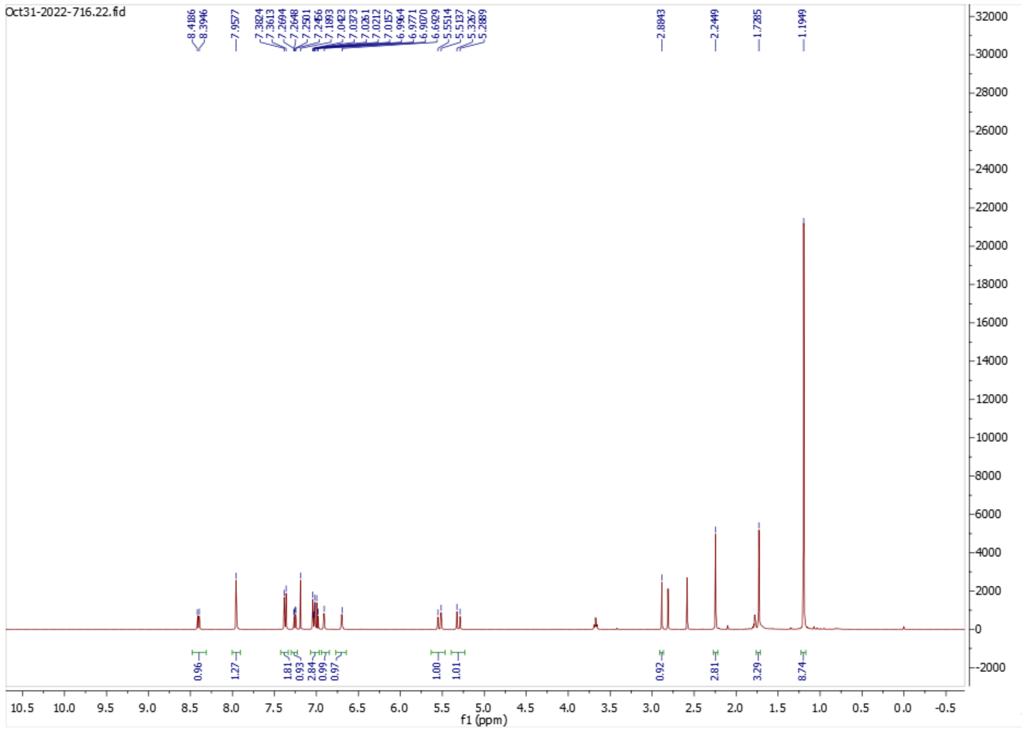

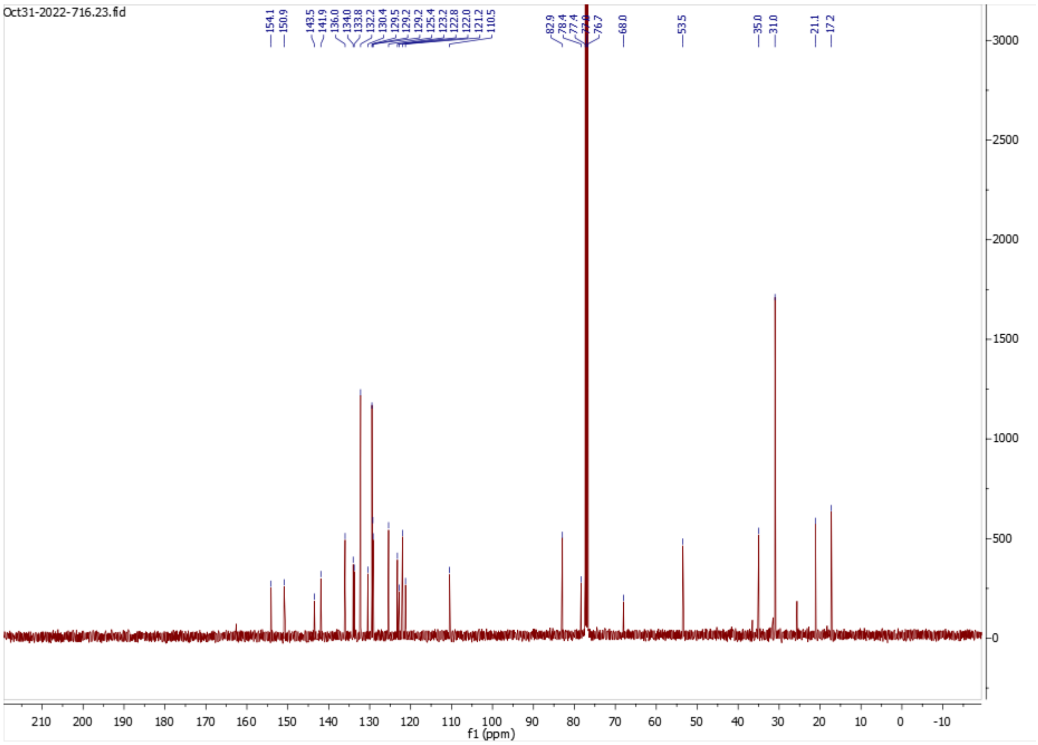

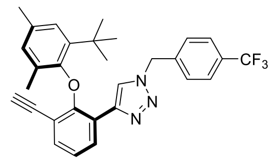
CF3
O
N
3e

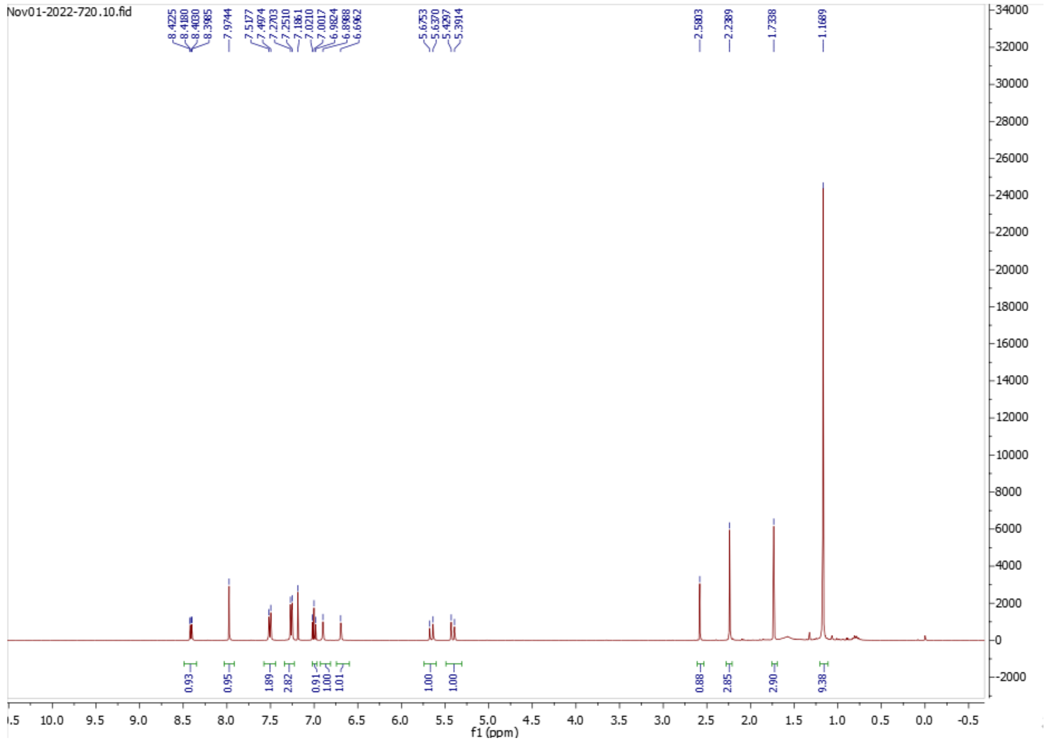
Nov01-2022-720.10.fid
8.4225
8.4180
8.4030
8.3985
7.9744
7.5177
7.4974
7.2703
7.2510
7.1861
7.0210
7.0017
6.9824
6.8988
6.6962
5.6753
5.6370
5.4297
5.3914
2.5803
2.2389
1.7338
1.1689
0.93
0.95
1.89
2.82
0.91
1.00
1.01
1.00
1.00
0.88
2.85
2.90
9.38
f1 (ppm)

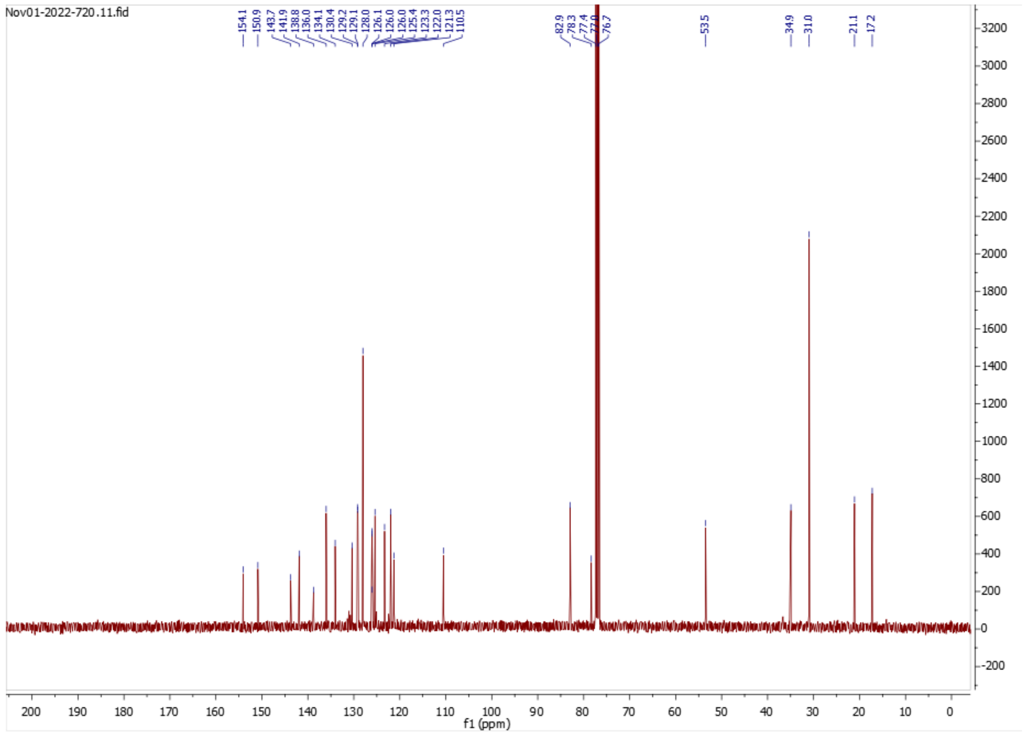
Nov01-2022-720.11.fid
154.1
150.9
143.7
141.9
138.8
136.0
134.1
130.4
129.2
129.1
128.0
126.1
126.0
126.0
125.4
123.3
122.0
121.3
110.5
82.9
78.3
77.4
77.0
76.7
53.5
34.9
31.0
21.1
17.2
f1 (ppm)

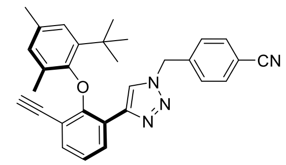

CN
O
N
N
N

3f

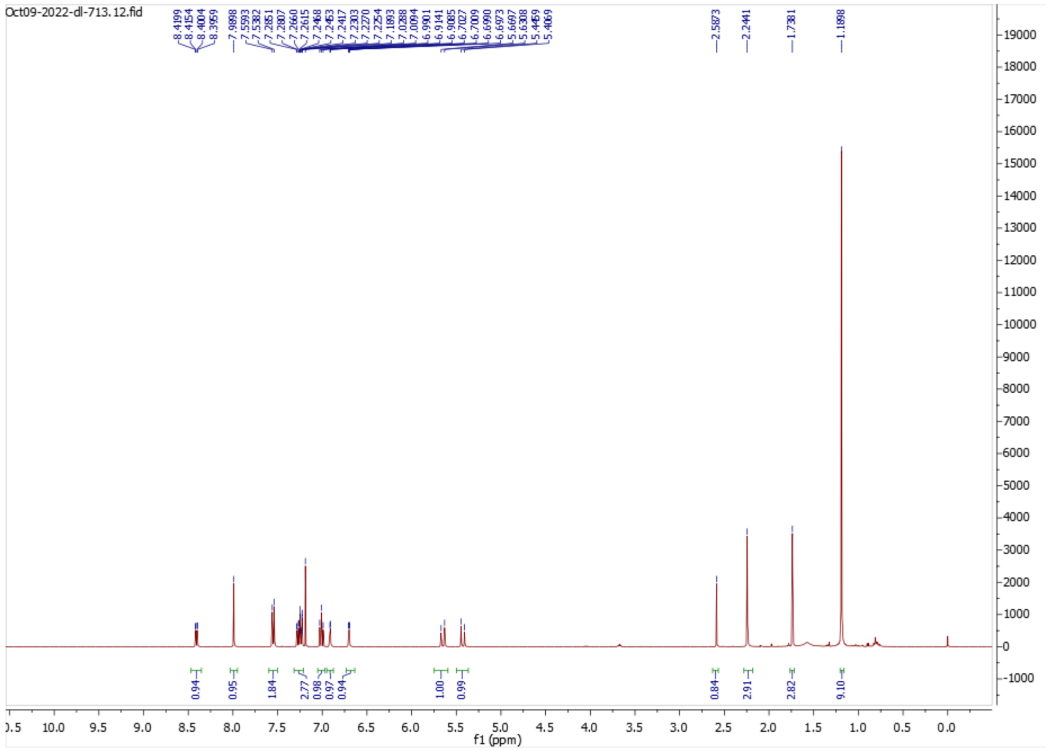

Oct09-2022-dl-713.12.fid
8.4199
8.4154
8.4004
8.3959
7.9898
7.5593
7.5382
7.2851
7.2807
7.2660
7.2615
7.2468
7.2453
7.2417
7.2303
7.2270
7.2254
7.1893
7.0288
7.0094
6.9901
6.9141
6.9085
6.7027
6.7009
6.6990
6.6973
6.6697
5.6308
5.4459
5.4069
2.5873
2.2441
1.7381
1.1898
0.94
0.95
1.84
2.77
0.98
0.97
0.94
1.00
0.99
0.84
2.91
2.82
9.10
f1 (ppm)

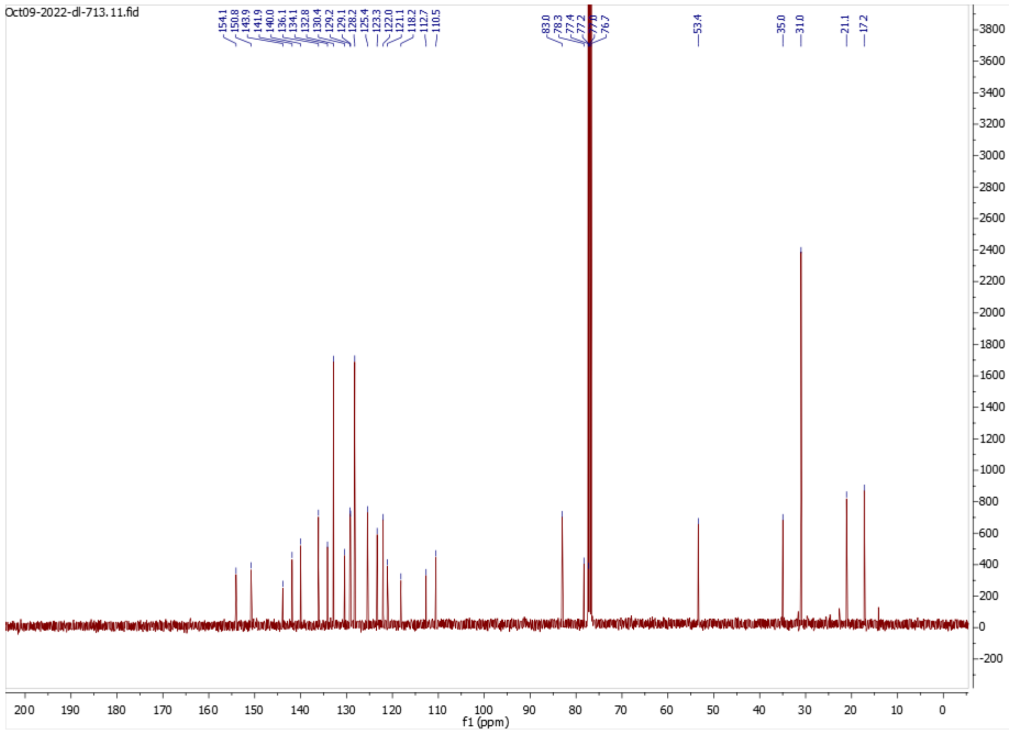

Oct09-2022-dl-713.11.fid
154.1
150.8
143.9
141.9
140.0
136.1
134.1
132.8
130.4
129.2
129.1
128.2
125.4
123.3
122.0
121.1
118.2
112.7
110.5
83.0
78.3
77.4
77.2
77.0
76.7
53.4
35.0
31.0
21.1
17.2
f1 (ppm)

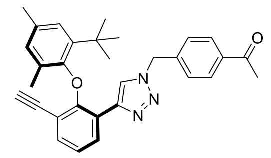

3g

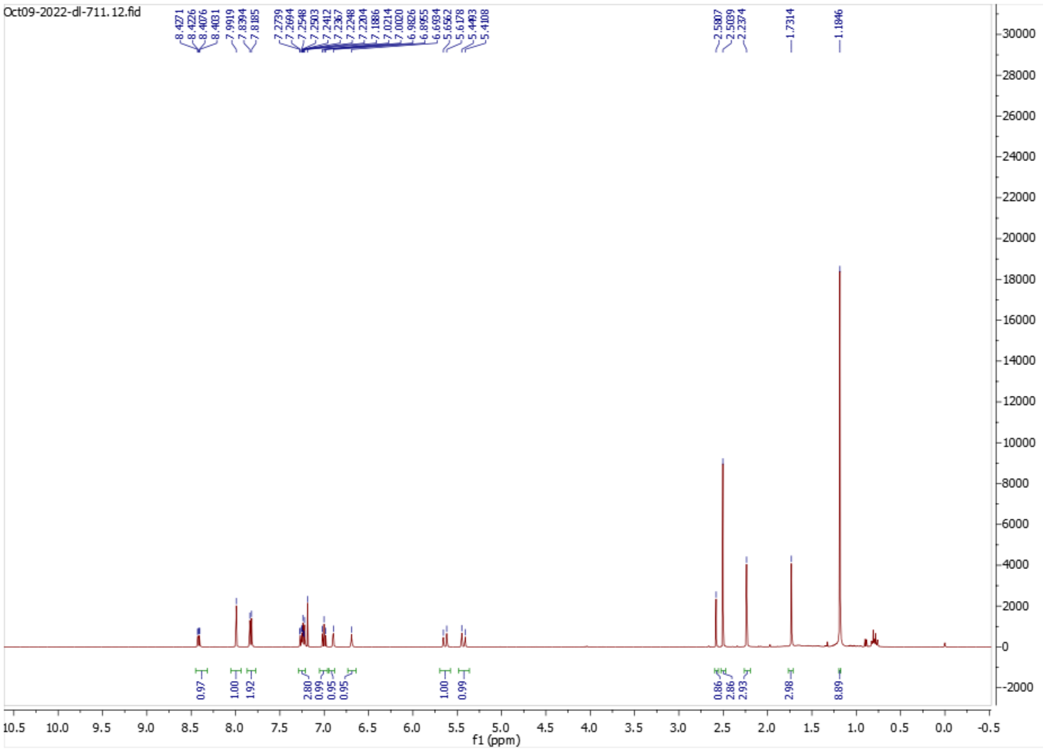
Oct09-2022-dl-711.12.fid
8.4271
8.4226
8.4076
8.4031
7.9919
7.8394
7.8185
7.2739
7.2694
7.2548
7.2503
7.2412
7.2367
7.2248
7.2204
7.1886
7.0214
7.0020
6.9826
6.8955
6.6934
5.6562
5.6178
5.4493
5.4108
2.5807
2.5039
2.2374
1.7314
1.1846
0.97
1.00
1.92
2.80
0.99
0.95
0.95
1.00
0.99
0.86
2.86
2.93
2.98
8.89
f1 (ppm)

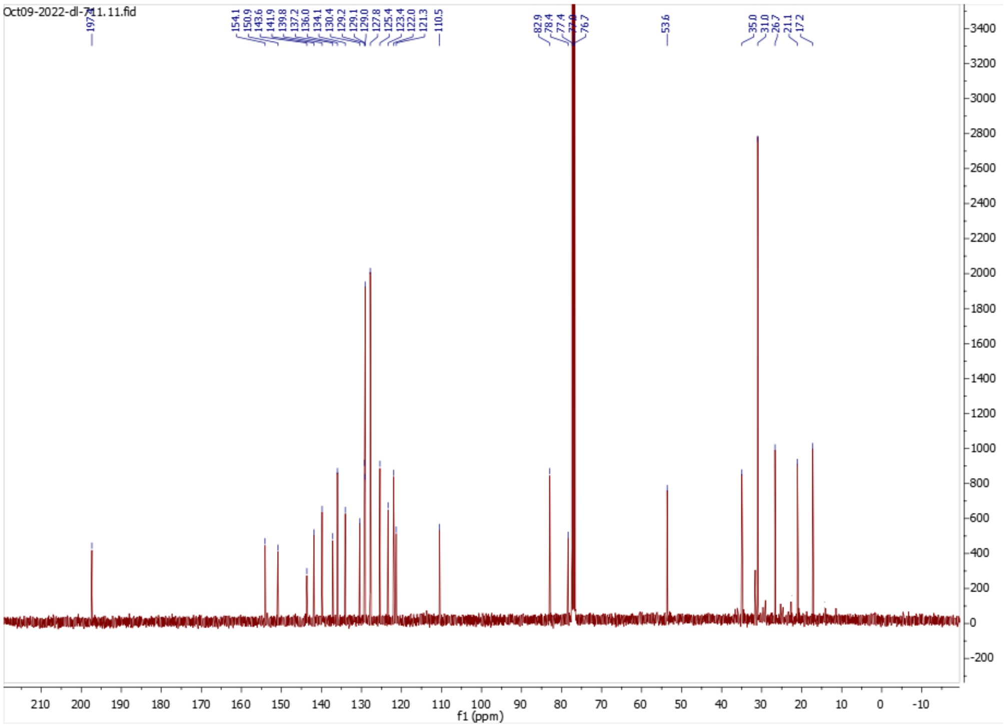
Oct09-2022-dl-711.11.fid
154.1
150.9
143.6
141.9
139.8
137.2
136.0
134.1
130.4
129.2
129.1
129.0
127.8
125.4
123.4
122.0
121.3
110.5
82.9
78.4
77.4
77.0
76.7
53.6
35.0
31.0
26.7
21.1
17.2
f1 (ppm)

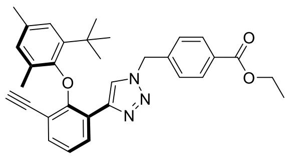

3h

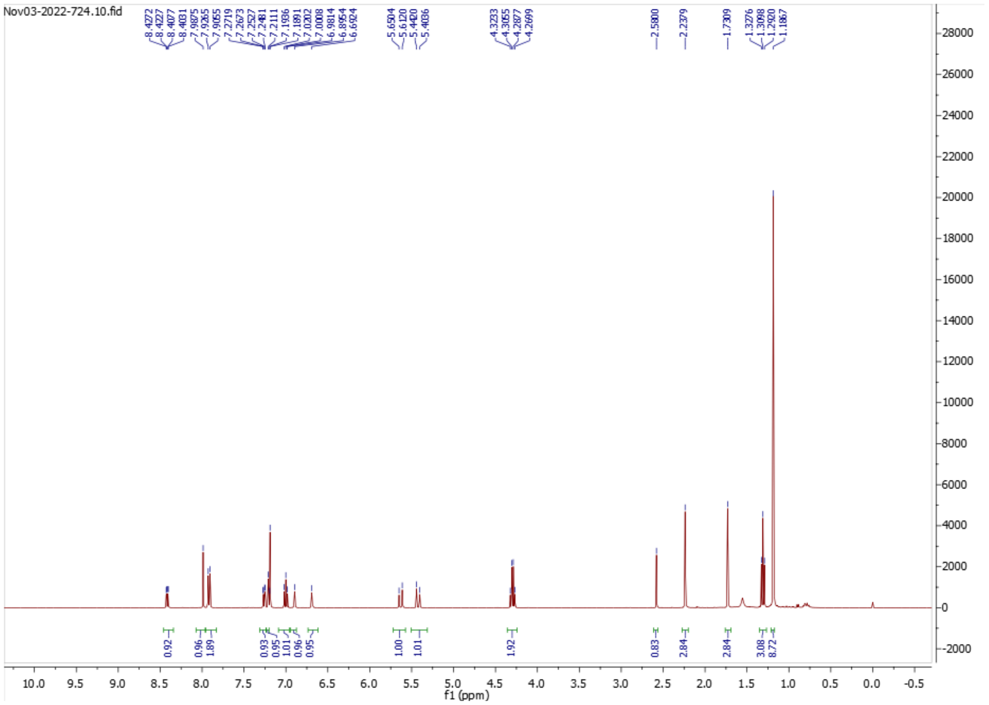

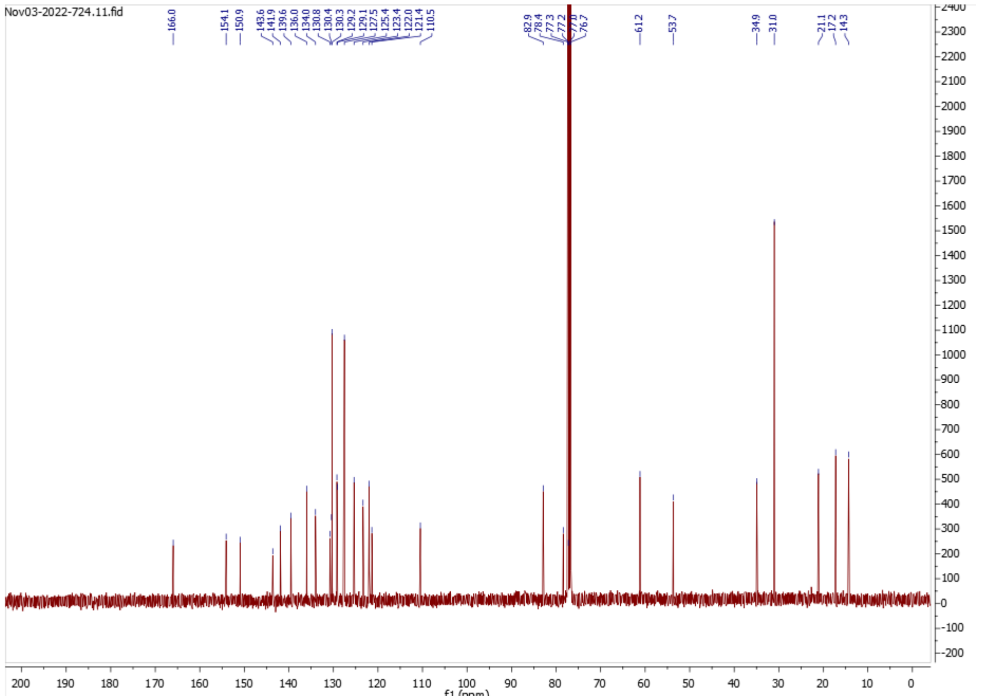

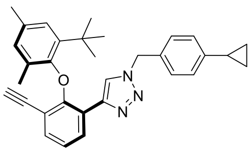

3i

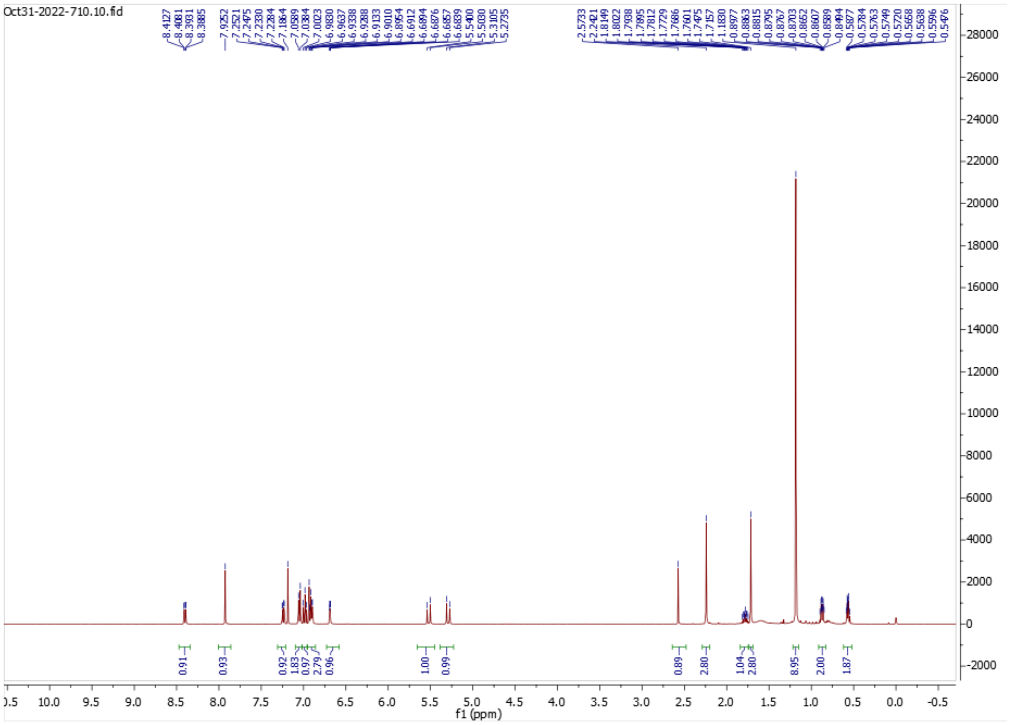

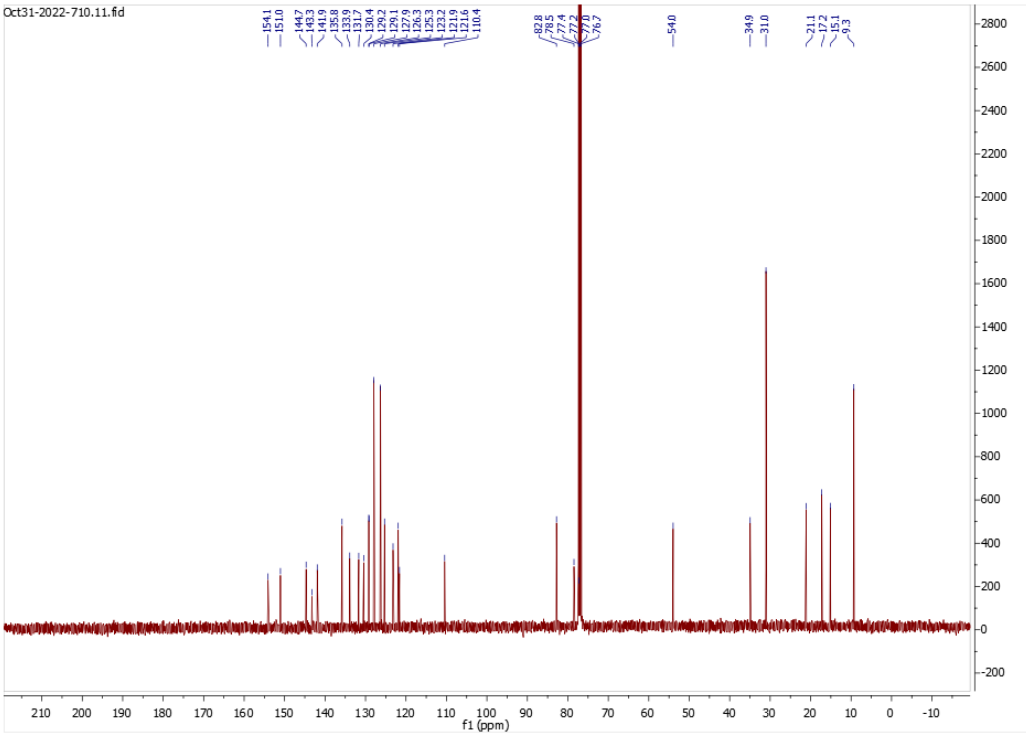

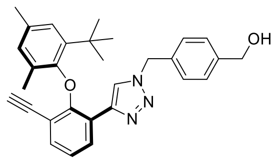

OH
O
N
N
N

3j

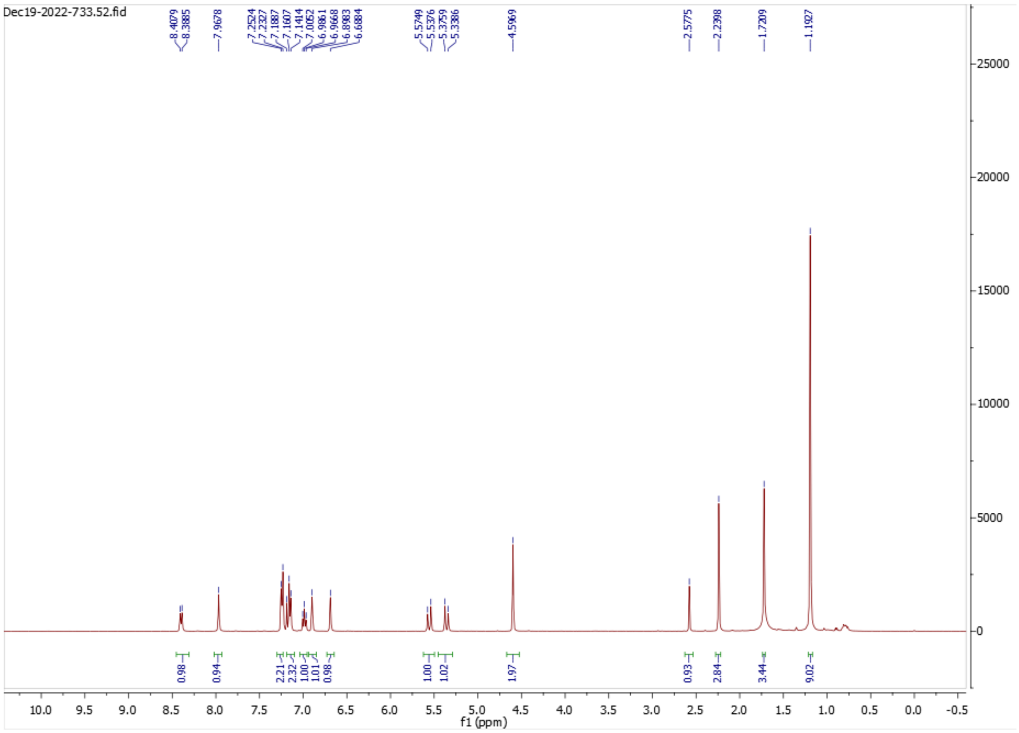

Dec19-2022-733.52.fid
8.4079
8.3885
7.9678
7.2524
7.2327
7.1887
7.1607
7.1414
7.0052
6.9861
6.9668
6.8983
6.6884
5.5749
5.5376
5.3759
5.3386
4.5969
2.5775
2.2398
1.7209
1.1927
0.98
0.94
2.21
2.32
1.00
1.01
0.98
1.00
1.02
1.97
0.93
2.84
3.44
9.02
f1 (ppm)

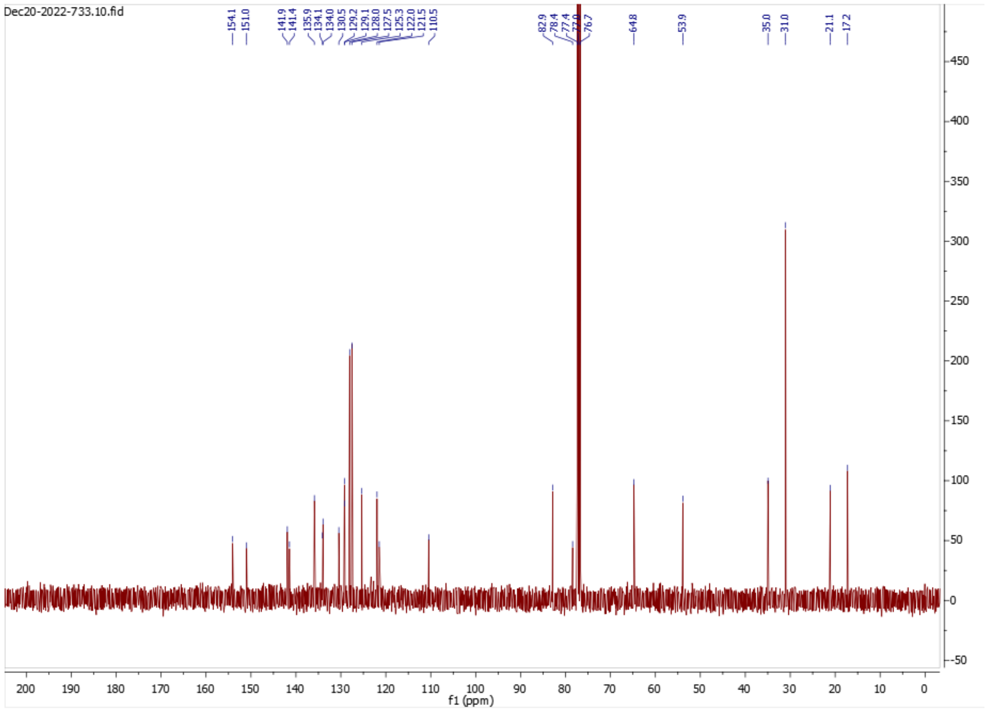

Dec20-2022-733.10.fid
154.1
151.0
141.9
141.4
135.9
134.1
134.0
130.5
129.2
129.1
128.0
127.5
125.3
122.0
121.5
110.5
82.9
78.4
77.4
77.0
76.7
64.8
53.9
35.0
31.0
21.1
17.2
f1 (ppm)

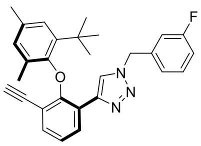

3k

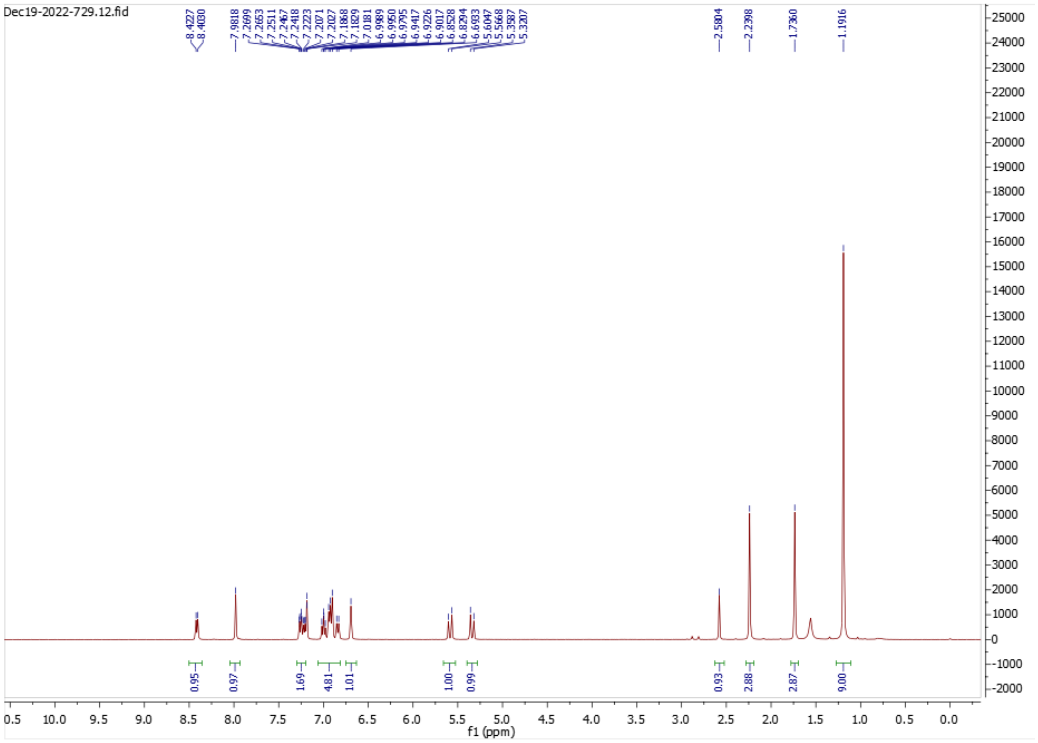

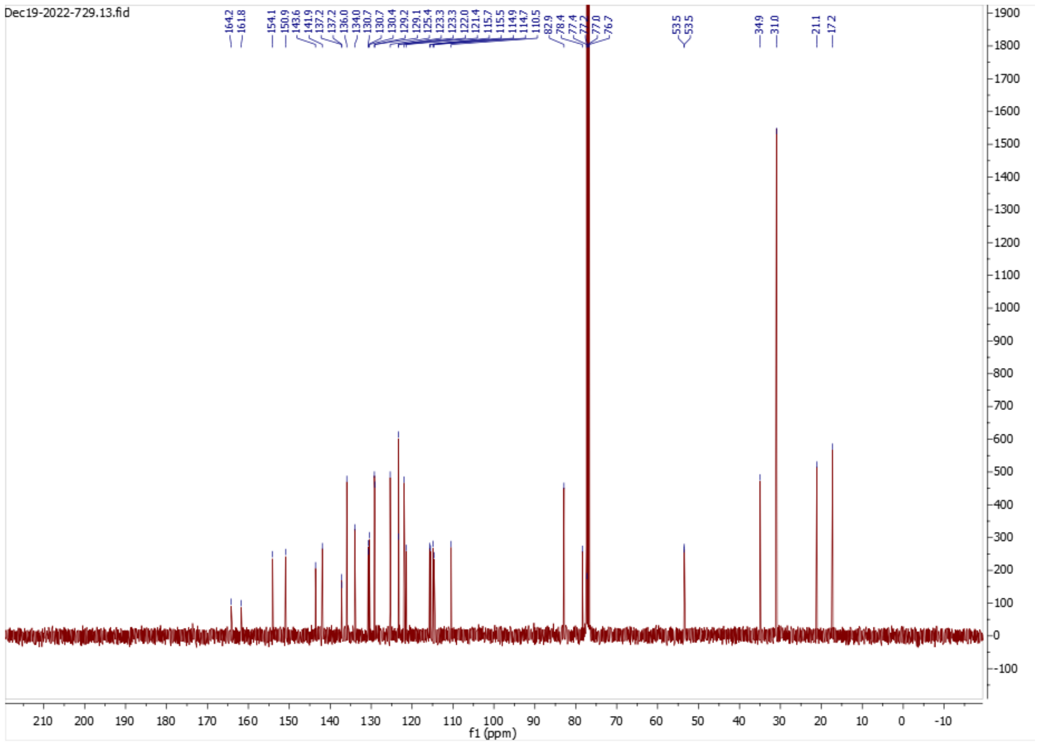

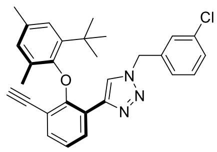
Cl
O
N
N
N

3l

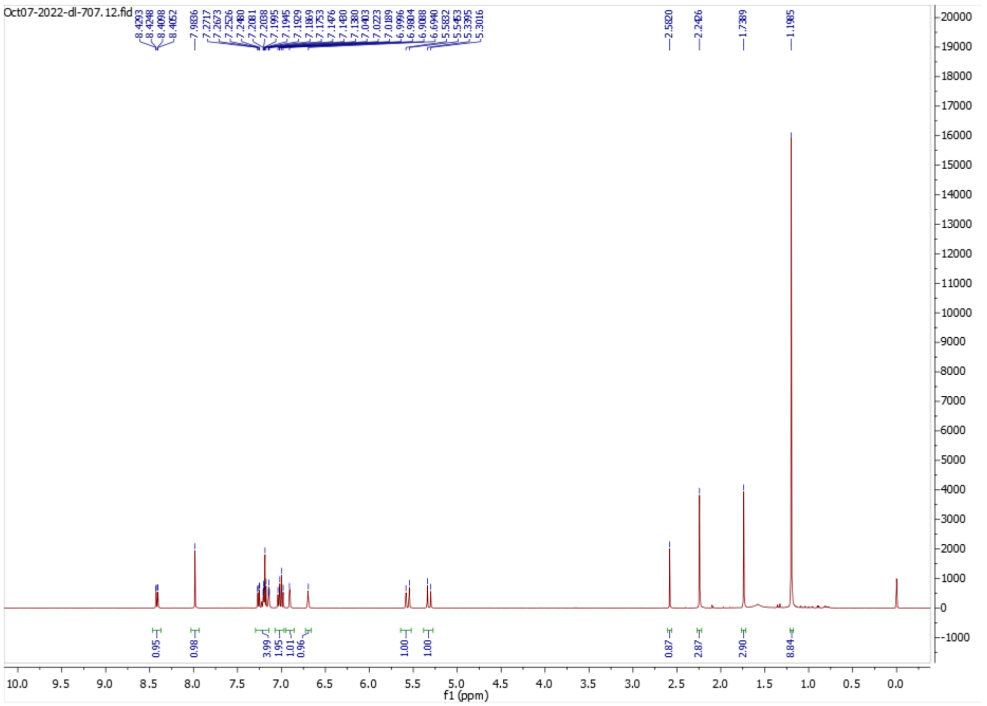
Oct07-2022-dl-707.12.fid
8.4293
8.4248
8.4098
8.4052
7.9836
7.2717
7.2673
7.2526
7.2480
7.2081
7.2038
7.1995
7.1945
7.1929
7.1869
7.1753
7.1476
7.1430
7.1380
7.0403
7.0223
7.0189
6.9996
6.9804
6.9088
6.6940
5.5832
5.5463
5.3395
5.3016
2.5820
2.2426
1.7389
1.1985
0.95
0.98
3.99
1.95
1.01
0.96
1.00
1.00
0.87
2.87
2.90
8.84
f1 (ppm)

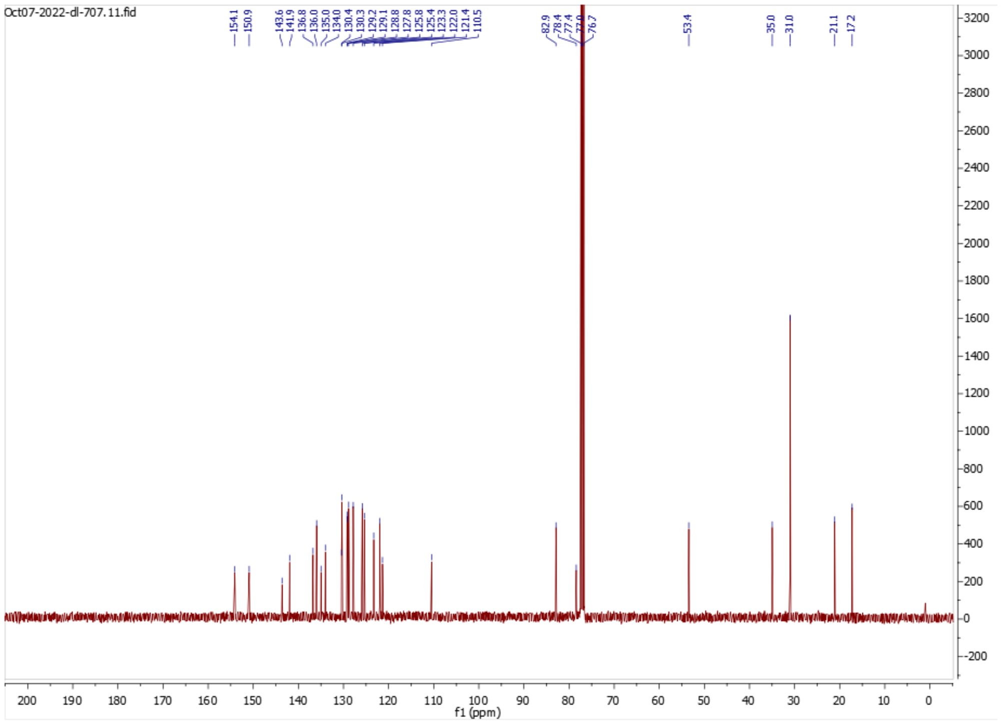
Oct07-2022-dl-707.11.fid
154.1
150.9
143.6
141.9
136.8
136.0
135.0
134.0
130.4
130.3
129.2
129.1
127.8
125.8
125.4
123.3
122.0
121.4
110.5
82.9
78.4
77.4
77.0
76.7
53.4
35.0
31.0
21.1
17.2
f1 (ppm)

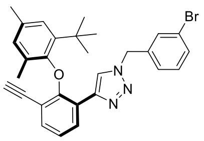

Br
O
N
N
N

3m

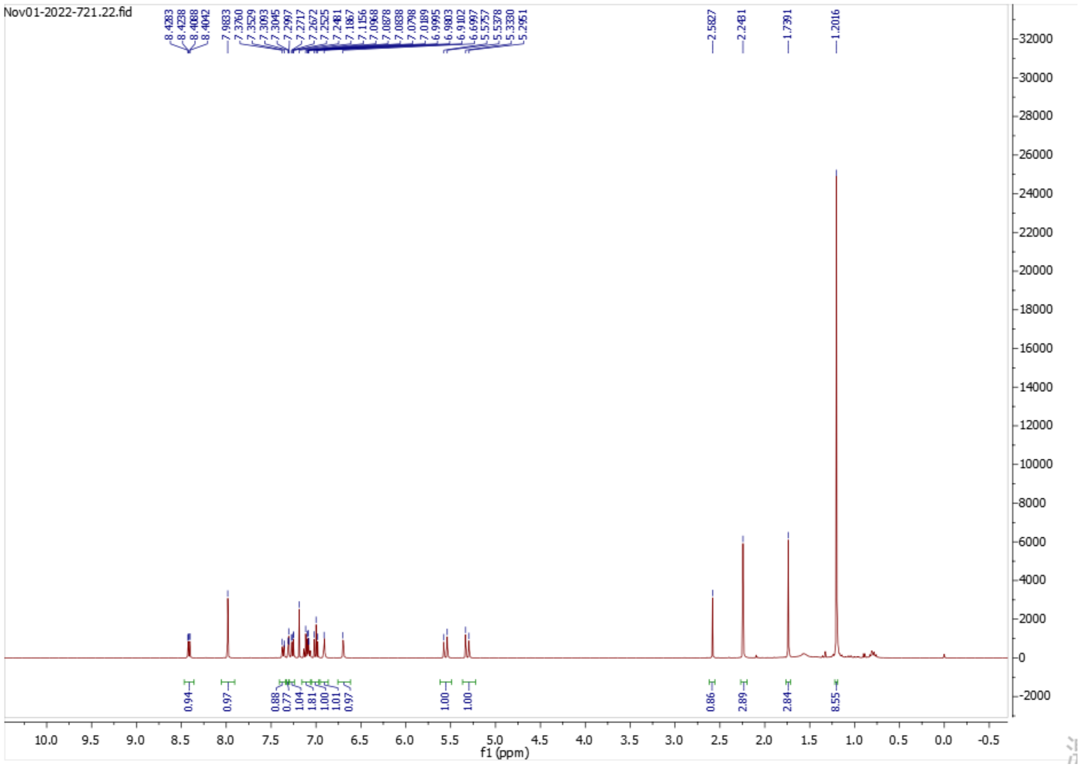

Nov01-2022-721.22.fid
8.4283
8.4238
8.4088
8.4042
7.9833
7.3760
7.3529
7.3093
7.3045
7.2907
7.2717
7.2672
7.2525
7.2481
7.1867
7.1156
7.0968
7.0878
7.0838
7.0798
7.0189
6.9995
6.9803
6.9102
6.6997
5.5757
5.5378
5.3330
5.2951
2.5827
2.2431
1.7391
1.2016
0.94
0.97
0.88
0.77
1.04
1.81
1.00
1.01
0.97
1.00
1.00
0.86
2.89
2.84
8.55
f1 (ppm)

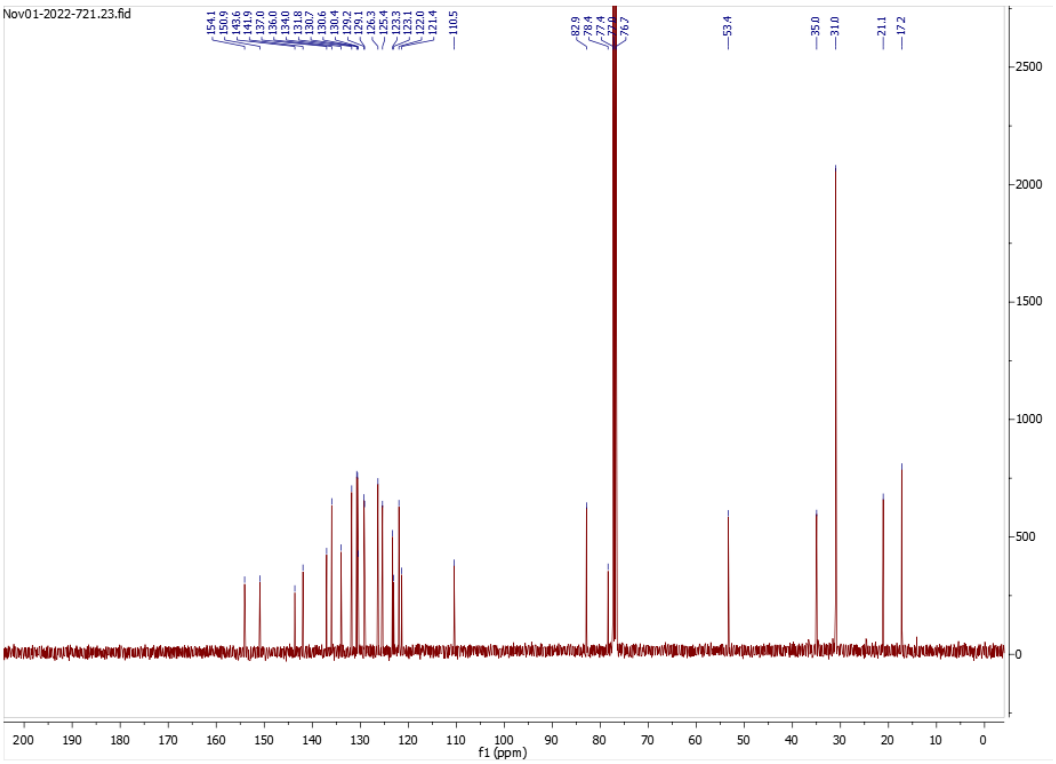

Nov01-2022-721.23.fid
154.1
150.9
143.6
141.9
137.0
136.0
134.0
131.8
130.7
130.6
130.4
129.2
129.1
126.3
125.4
123.3
123.1
122.0
121.4
110.5
82.9
78.4
77.4
77.0
76.7
53.4
35.0
31.0
21.1
17.2
f1 (ppm)

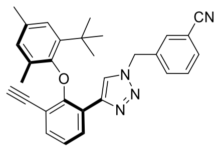
CN
O
N
N
N

3n

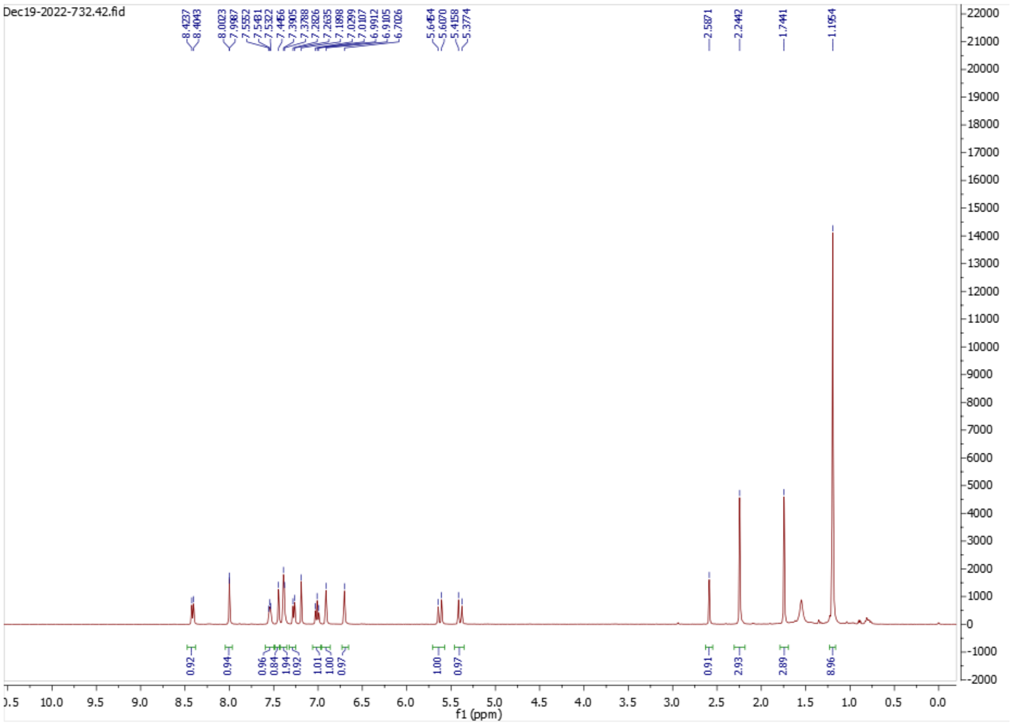
Dec19-2022-732.42.fid
8.4237
8.4043
8.0023
7.9987
7.5552
7.5431
7.5322
7.4456
7.3905
7.3788
7.2826
7.2635
7.1898
7.0299
7.0107
6.9912
6.9105
6.7026
5.6454
5.6070
5.4158
5.3774
2.5871
2.2442
1.7441
1.1954
0.92
0.94
0.96
0.84
1.94
0.92
1.01
1.00
0.97
1.00
0.97
0.91
2.93
2.89
8.96
f1 (ppm)

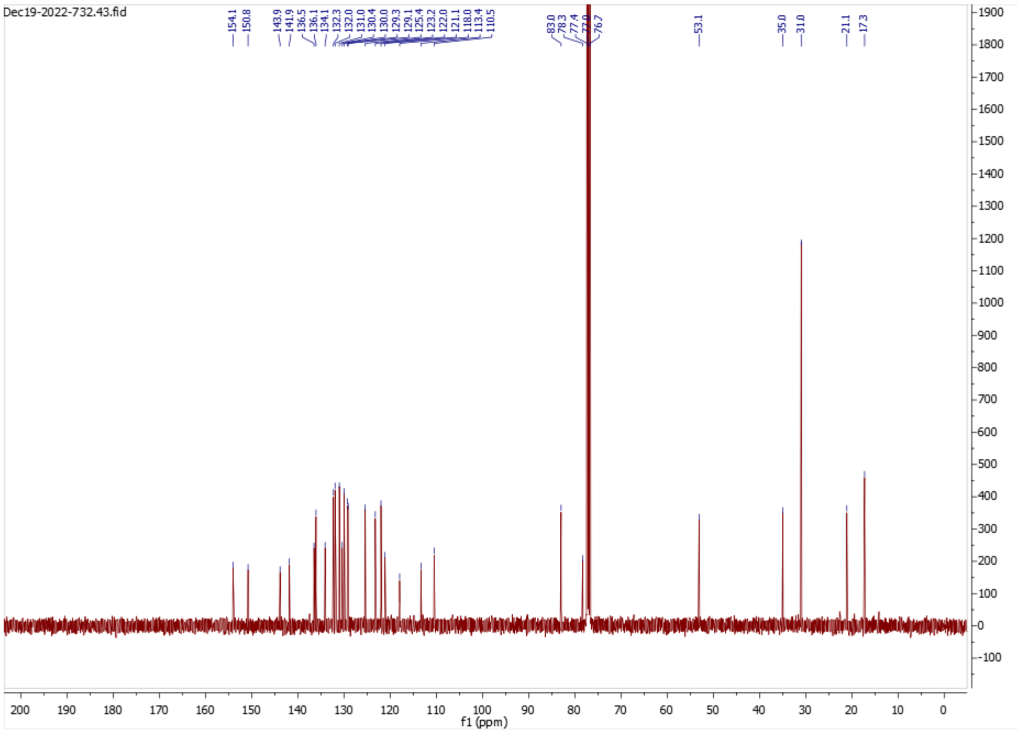
Dec19-2022-732.43.fid
154.1
150.8
143.9
141.9
136.5
136.1
134.1
132.3
132.0
131.0
130.4
130.0
129.3
129.1
125.4
123.2
122.0
121.1
118.0
113.4
110.5
83.0
78.3
77.4
77.0
76.7
53.1
35.0
31.0
21.1
17.3
f1 (ppm)

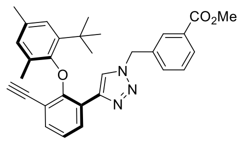
CO2Me
O
N
N
N

3o

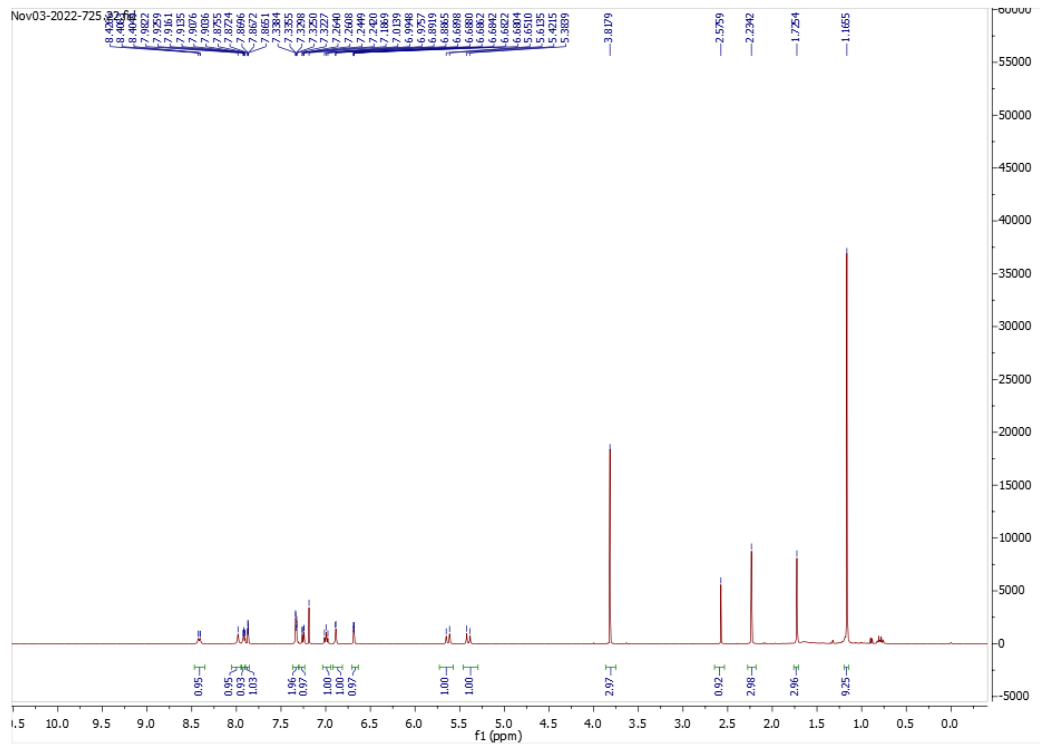
Nov03-2022-725.32.fid
3.8179
2.5759
2.2342
1.7254
1.1655
0.95
0.95
0.93
1.03
1.96
0.97
1.00
1.00
0.97
1.00
1.00
2.97
0.92
2.98
2.96
9.25
f1 (ppm)

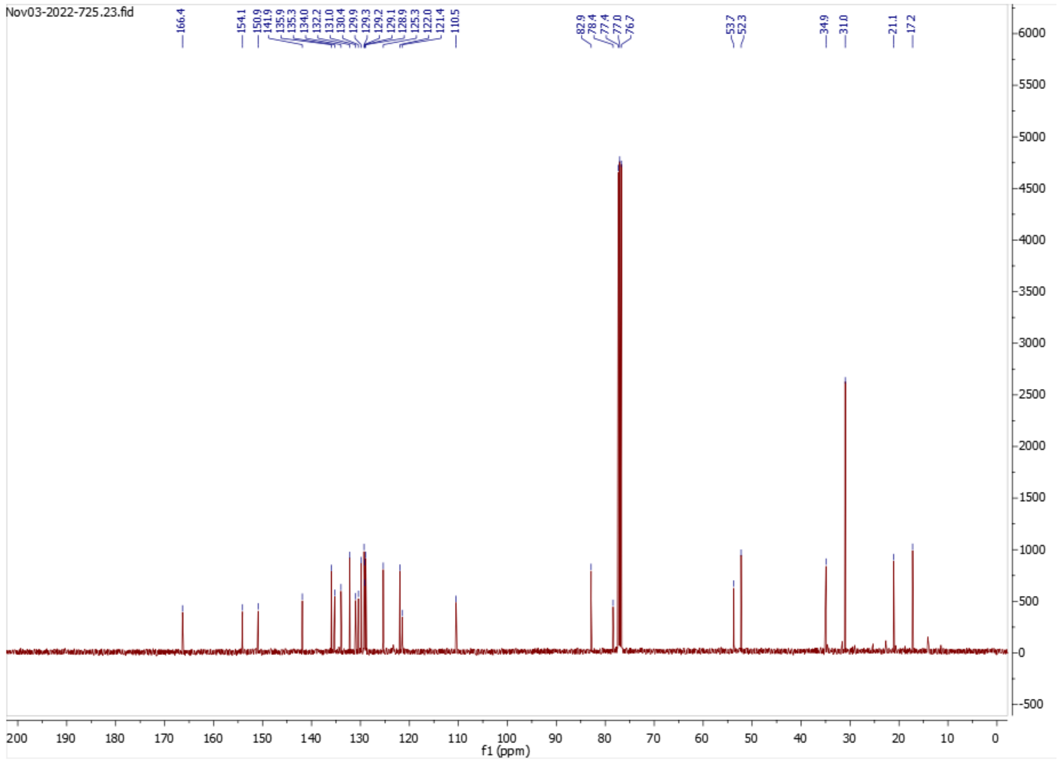
Nov03-2022-725.23.fid
166.4
154.1
150.9
141.9
135.9
135.3
134.0
132.2
131.0
130.4
129.9
129.3
129.2
129.1
128.9
125.3
122.0
121.4
110.5
82.9
78.4
77.4
77.0
76.7
53.7
52.3
34.9
31.0
21.1
17.2
f1 (ppm)

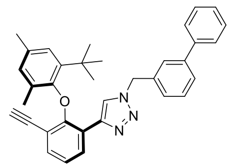

3p

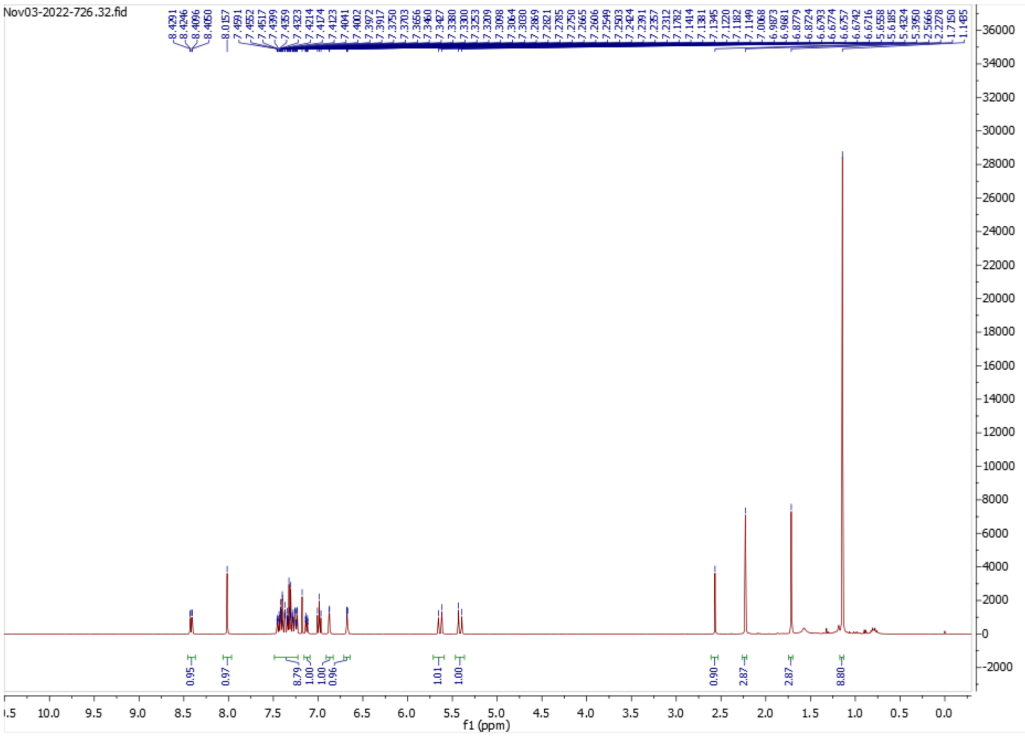

Nov03-2022-726.32.fid
f1 (ppm)

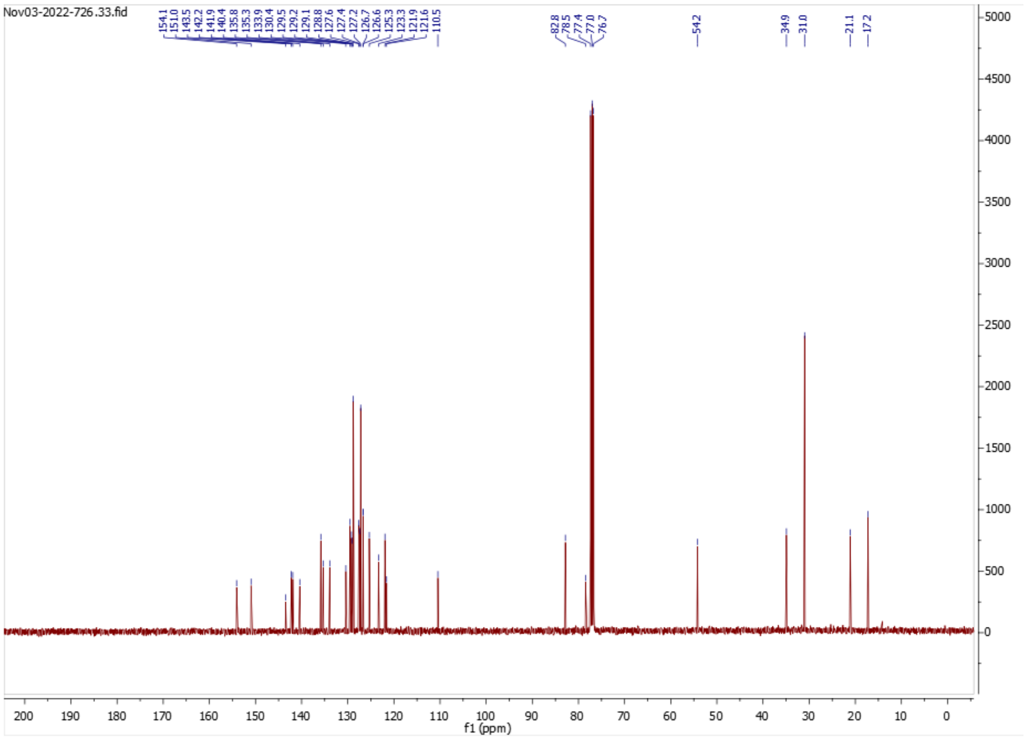

Nov03-2022-726.33.fid
154.1
151.0
143.5
142.2
141.9
140.4
135.8
135.3
133.9
130.4
129.5
129.2
129.1
128.8
127.6
127.4
127.2
126.7
126.6
125.3
123.3
121.9
121.6
110.5
82.8
78.5
77.4
77.0
76.7
54.2
34.9
31.0
21.1
17.2
f1 (ppm)

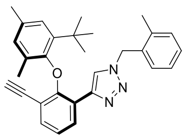
O
N
N
N

3q

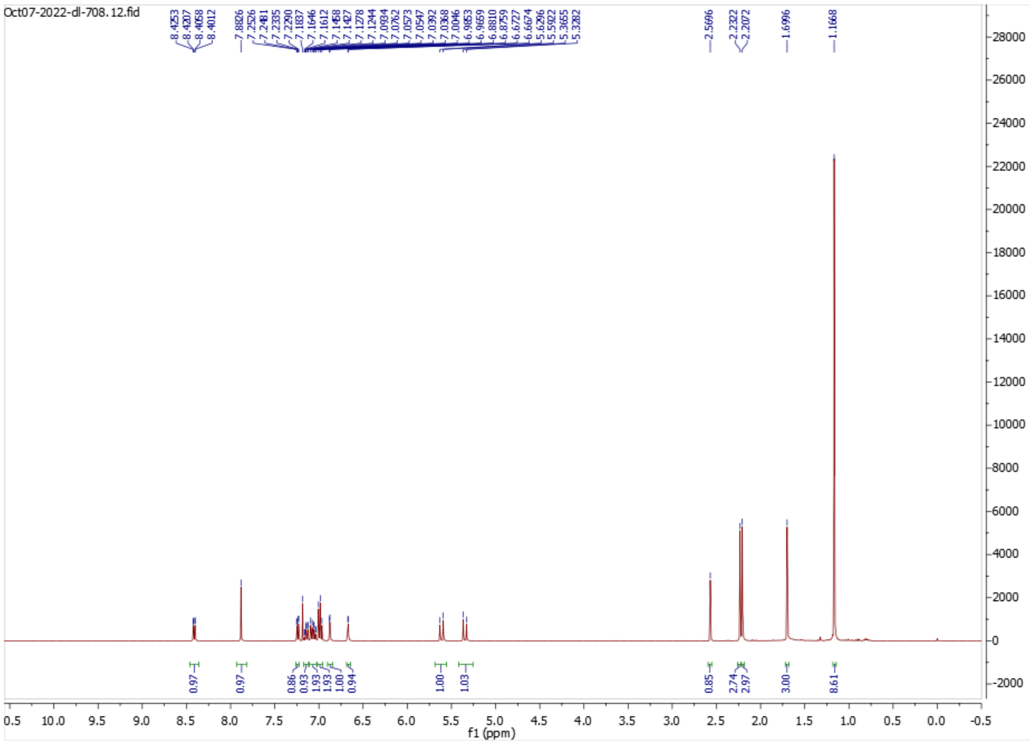
Oct07-2022-dl-708.12.fid
f1 (ppm)

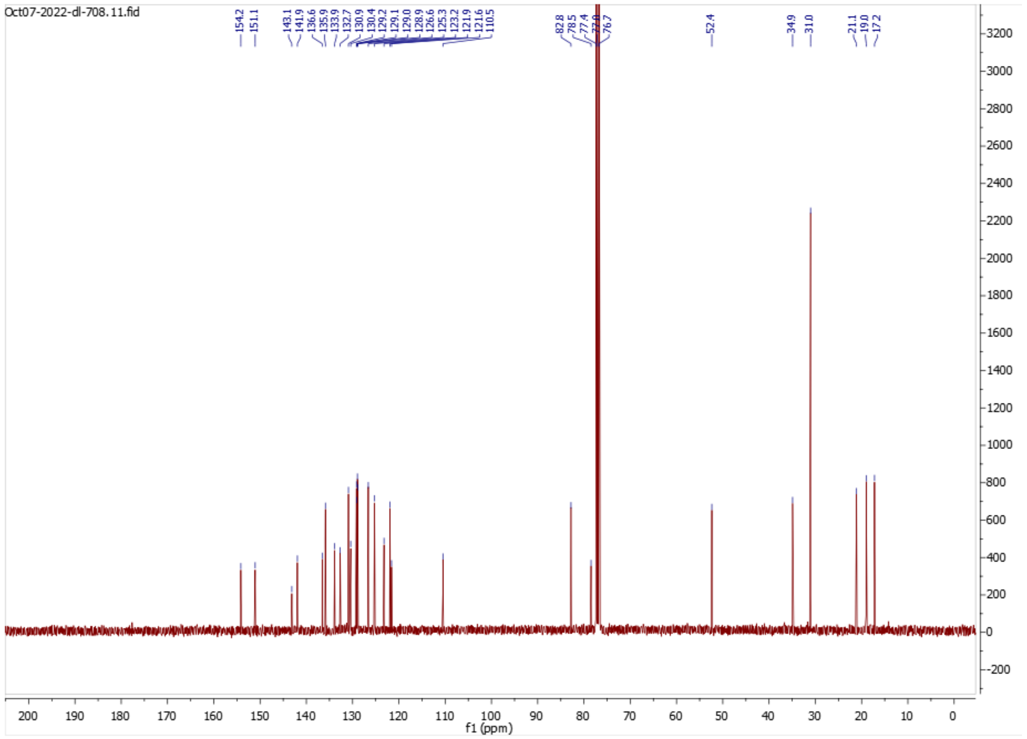
Oct07-2022-dl-708.11.fid
154.2
151.1
143.1
141.9
136.6
135.9
133.9
132.7
130.9
130.4
129.2
129.1
129.0
128.9
126.6
125.3
123.2
121.9
121.6
110.5
82.8
78.5
77.4
77.0
76.7
52.4
34.9
31.0
21.1
19.0
17.2
f1 (ppm)

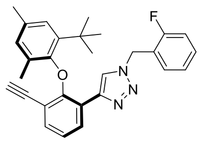
F
O
N
N
N

3r

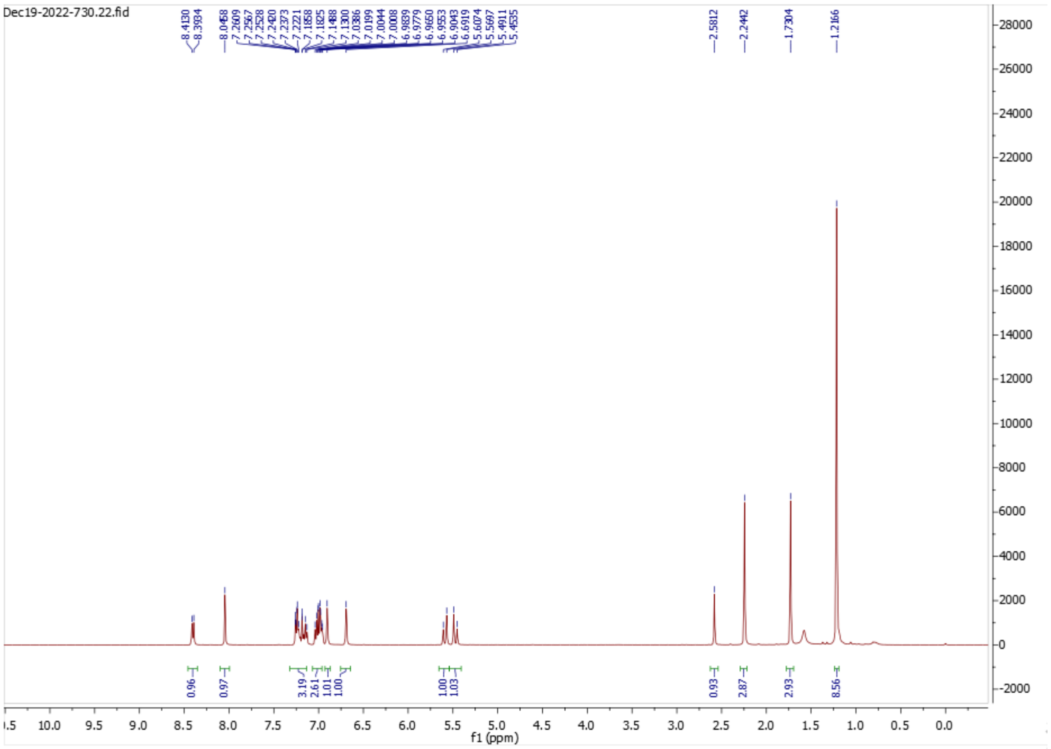
Dec19-2022-730.22.fid
8.4130
8.3934
8.0458
7.2609
7.2567
7.2528
7.2420
7.2373
7.2221
7.1858
7.1825
7.1488
7.1300
7.0386
7.0199
7.0044
7.0008
6.9839
6.9779
6.9650
6.9553
6.9043
6.6919
5.6074
5.5697
5.4911
5.4535
2.5812
2.2442
1.7304
1.2166
0.96
0.97
3.19
2.61
1.01
1.00
1.00
1.03
0.93
2.87
2.93
8.56
f1 (ppm)

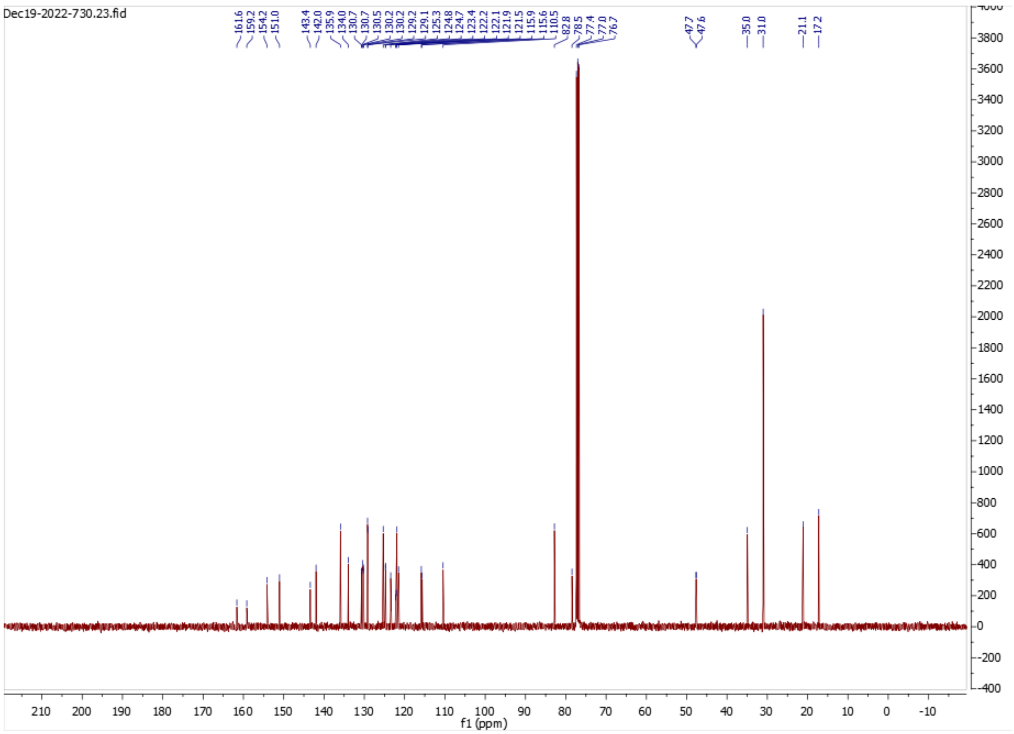
Dec19-2022-730.23.fid
161.6
159.2
154.2
151.0
143.4
142.0
135.9
134.0
130.7
130.7
130.5
130.2
130.2
129.2
129.1
125.3
124.8
124.7
123.4
122.2
122.1
121.9
121.5
115.9
115.6
110.5
82.8
78.5
77.4
77.0
76.7
47.7
47.6
35.0
31.0
21.1
17.2
f1 (ppm)

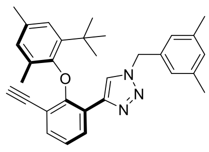

3s

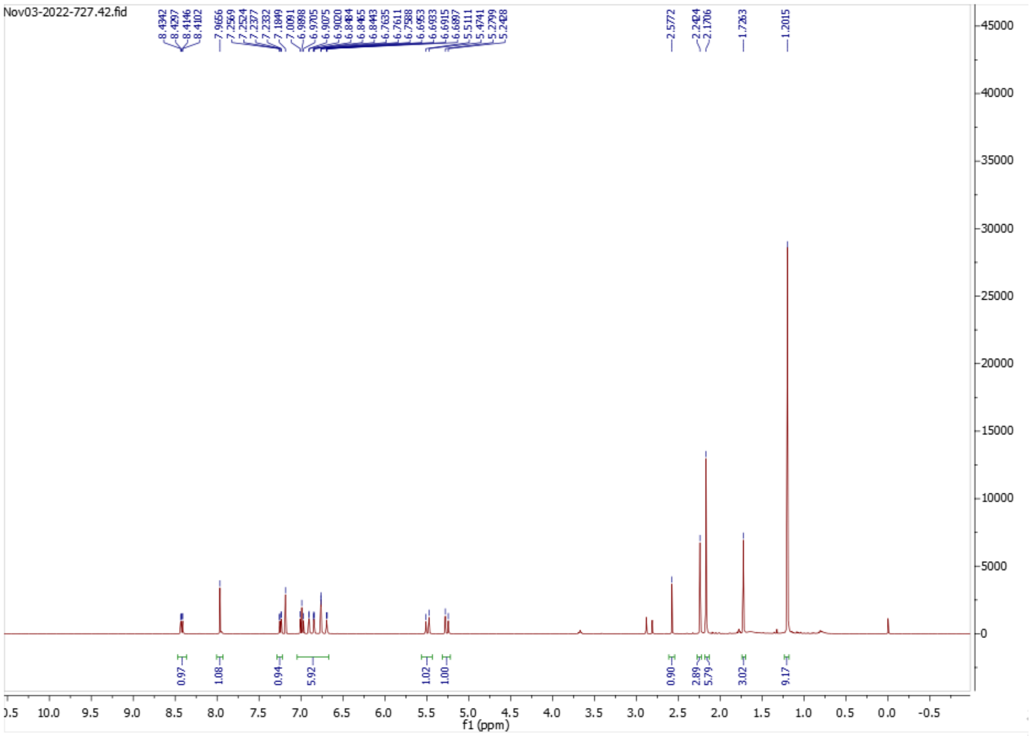
Nov03-2022-727.42.fid
8.4342
8.4297
8.4146
8.4102
7.9656
7.2569
7.2524
7.2377
7.2332
7.1899
7.0091
6.9898
6.9705
6.9075
6.9020
6.8484
6.8465
6.8443
6.7635
6.7611
6.7588
6.6953
6.6933
6.6915
6.6897
5.5111
5.4741
5.2799
5.2428
2.5772
2.2424
2.1706
1.7263
1.2015
0.97
1.08
0.94
5.92
1.02
1.00
0.90
2.89
5.79
3.02
9.17
f1 (ppm)

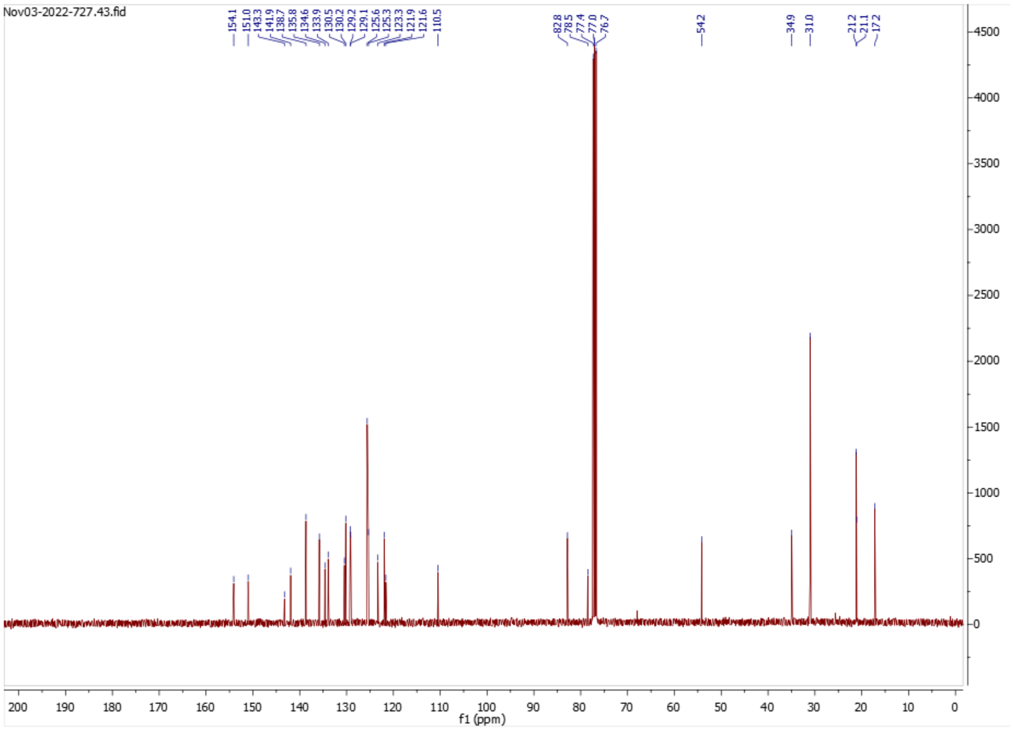
Nov03-2022-727.43.fid
154.1
151.0
143.3
141.9
138.7
135.8
134.6
133.9
130.5
130.2
129.2
129.1
125.6
125.3
123.3
121.9
121.6
110.5
82.8
78.5
77.4
77.0
76.7
54.2
34.9
31.0
21.2
21.1
17.2
f1 (ppm)

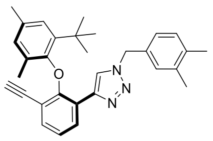
O
N
N
N

3t

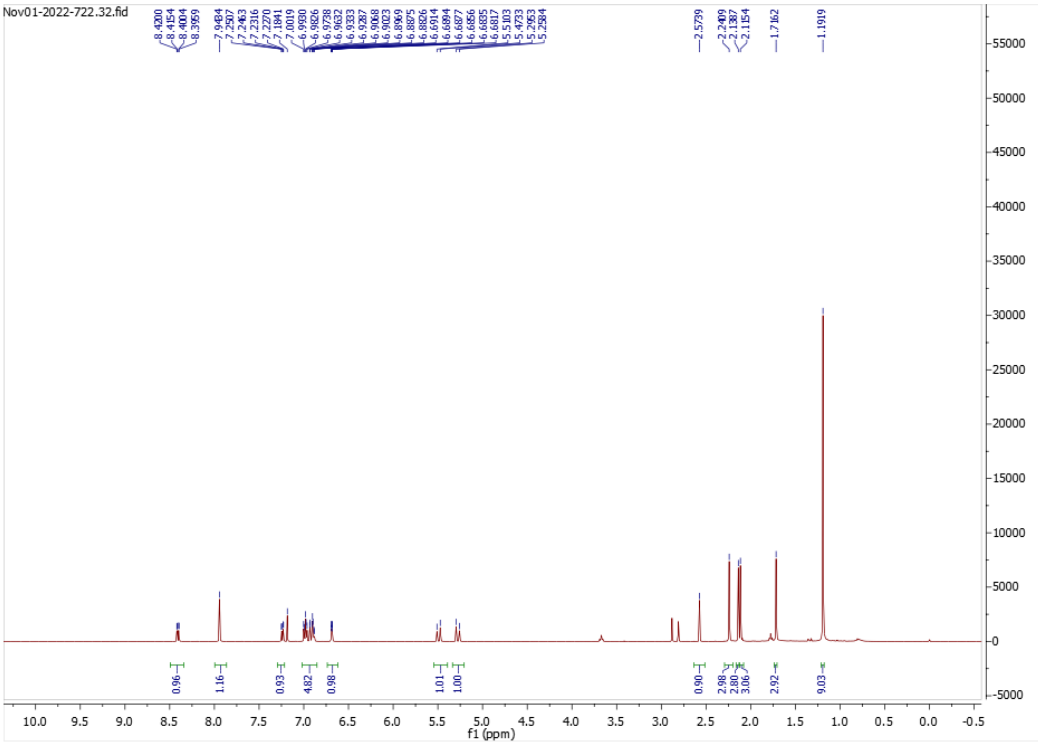
Nov01-2022-722.32.fid
8.4200
8.4154
8.4004
8.3959
7.9434
7.2507
7.2463
7.2316
7.2270
7.1841
7.0019
6.9930
6.9826
6.9738
6.9632
6.9333
6.9287
6.9068
6.9023
6.8969
6.8875
6.8826
6.6914
6.6894
6.6877
6.6856
6.6835
6.6817
5.5103
5.4733
5.2953
5.2584
2.5739
2.2409
2.1387
2.1154
1.7162
1.1919
0.96
1.16
0.93
4.82
0.98
1.01
1.00
0.90
2.98
2.80
3.06
2.92
9.03
f1 (ppm)

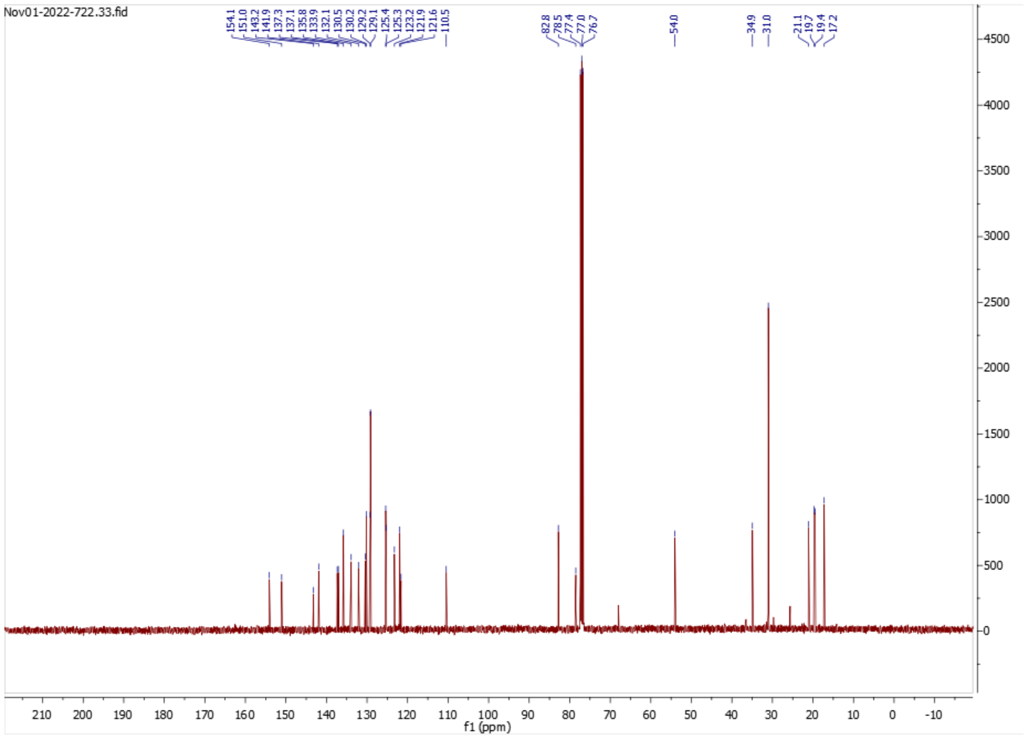
Nov01-2022-722.33.fid
154.1
151.0
143.2
141.9
137.3
137.1
135.8
133.9
132.1
130.5
130.2
129.2
129.1
125.4
125.3
123.2
121.9
121.6
110.5
82.8
78.5
77.4
77.0
76.7
54.0
34.9
31.0
21.1
19.7
19.4
17.2
f1 (ppm)

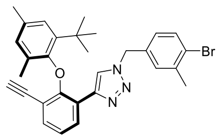

3u

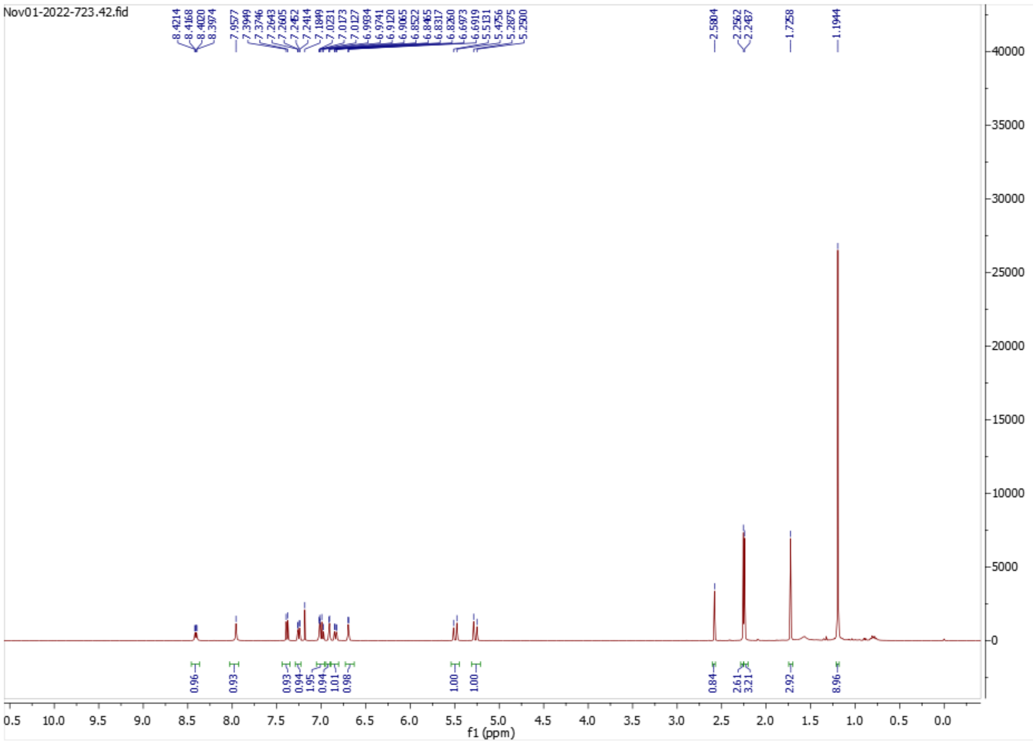

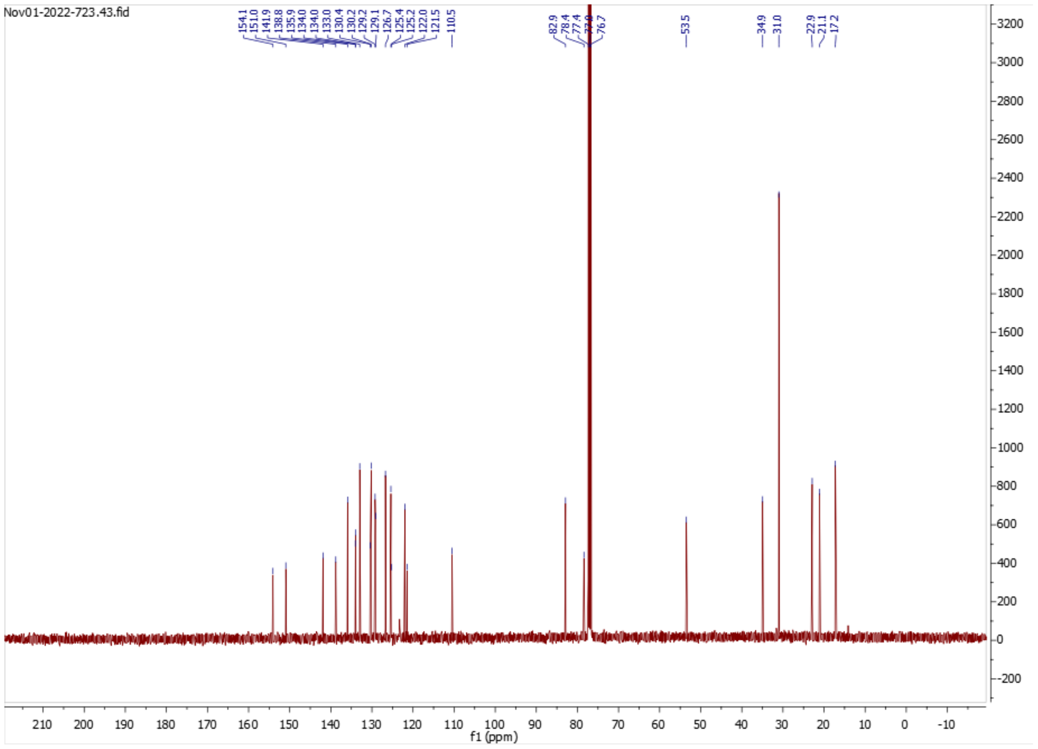

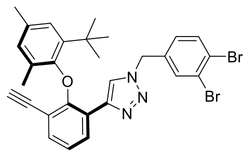
O
N
N
N
Br
Br

3v

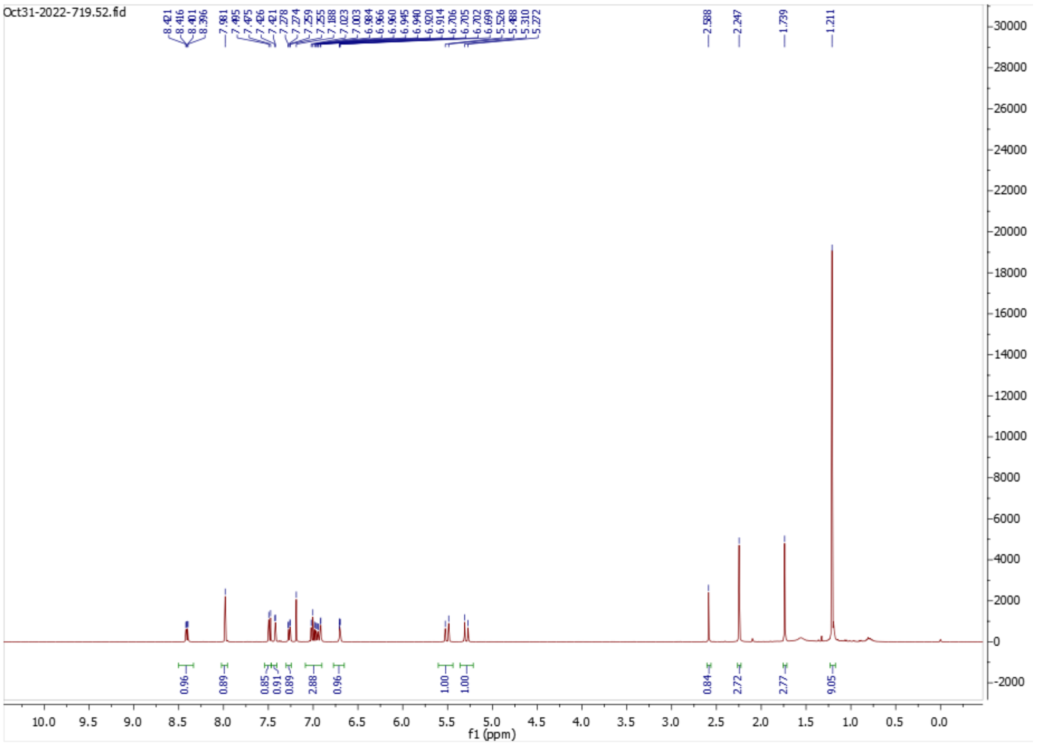
Oct31-2022-719.52.fid
f1 (ppm)

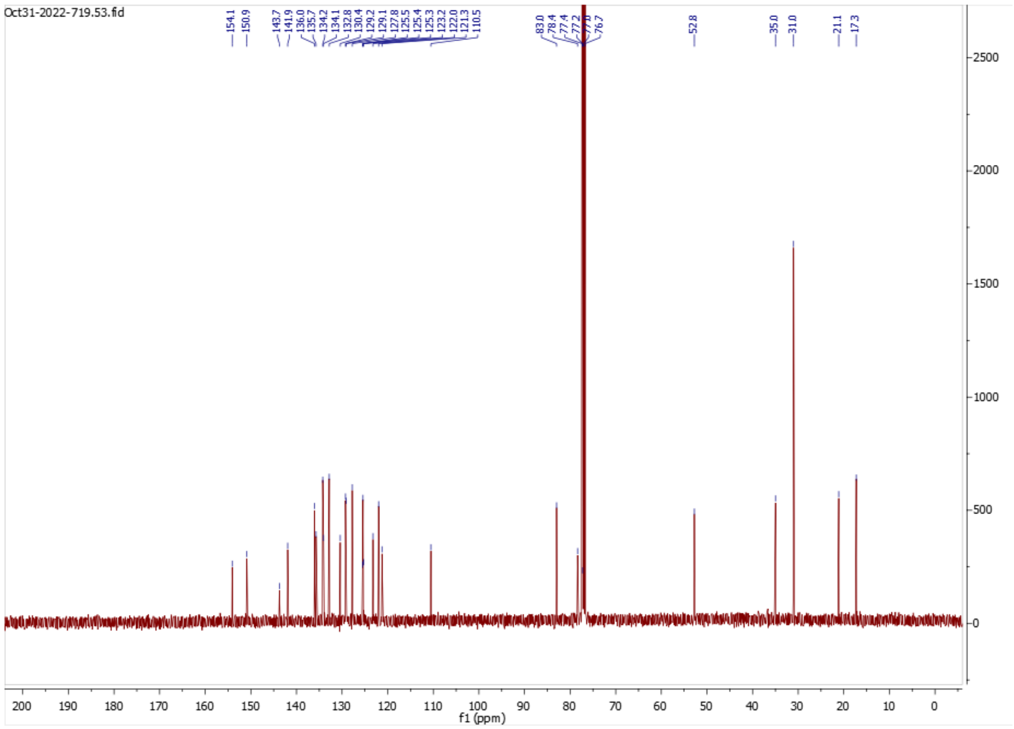
Oct31-2022-719.53.fid
f1 (ppm)

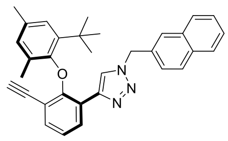

3w

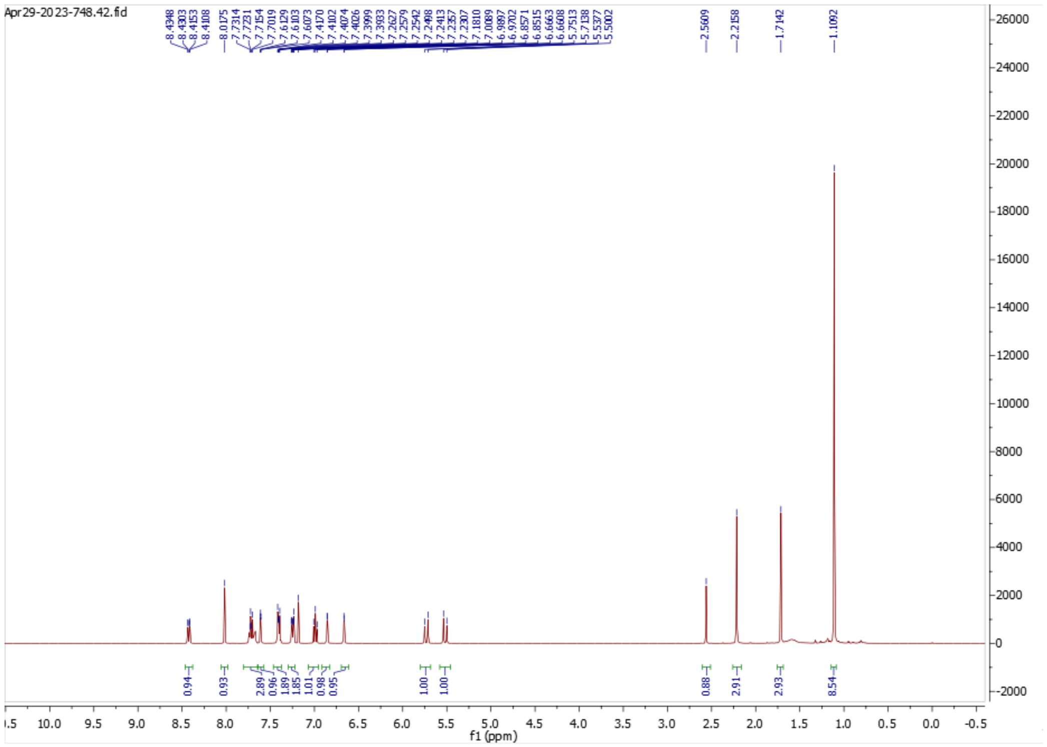

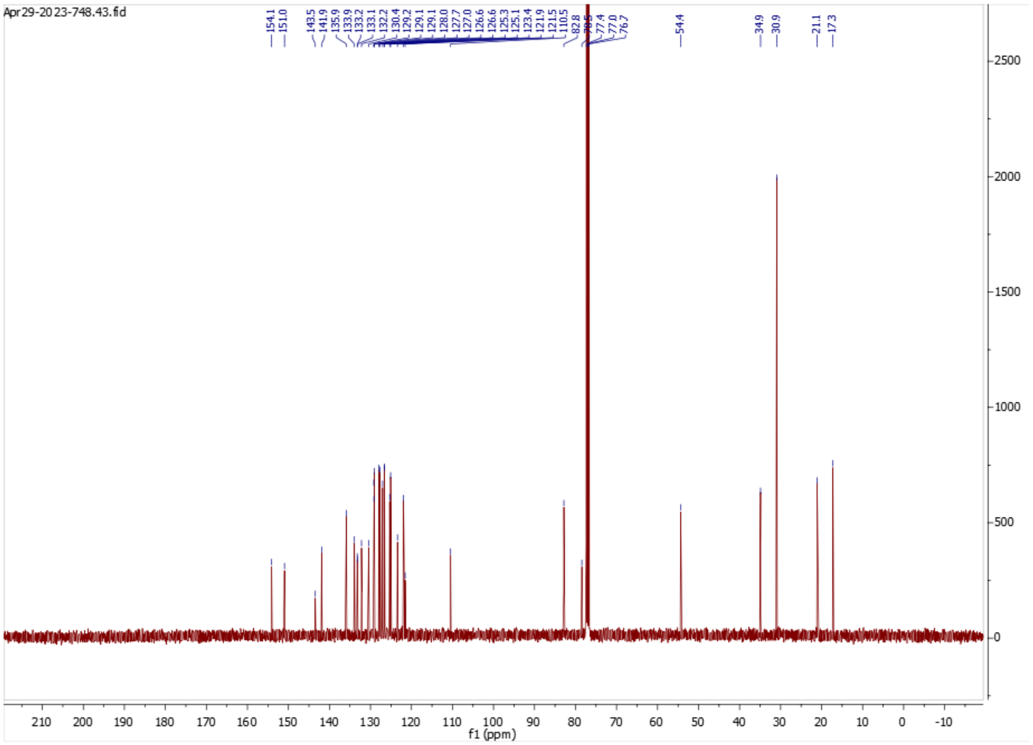

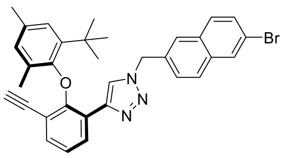

3x

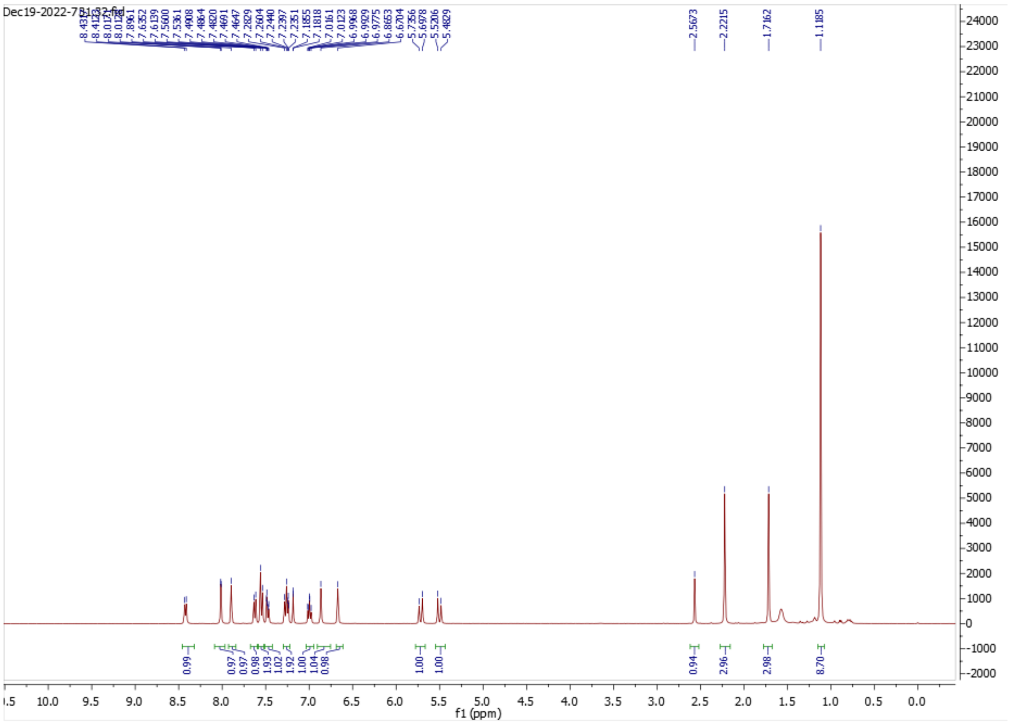

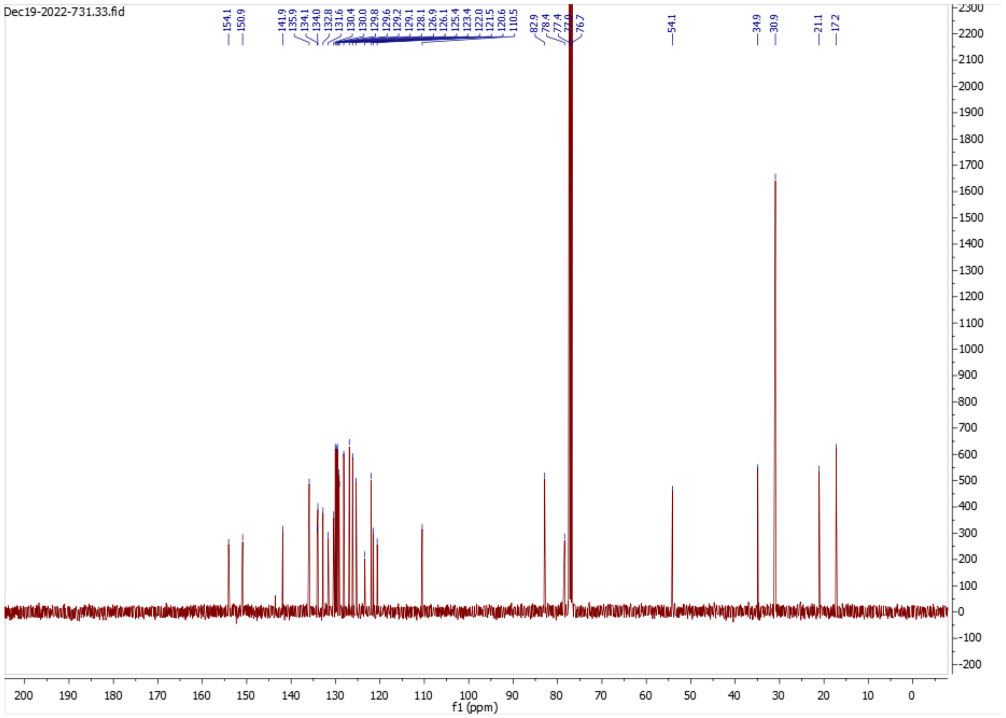

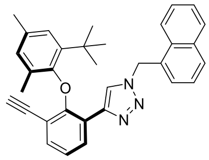

3y

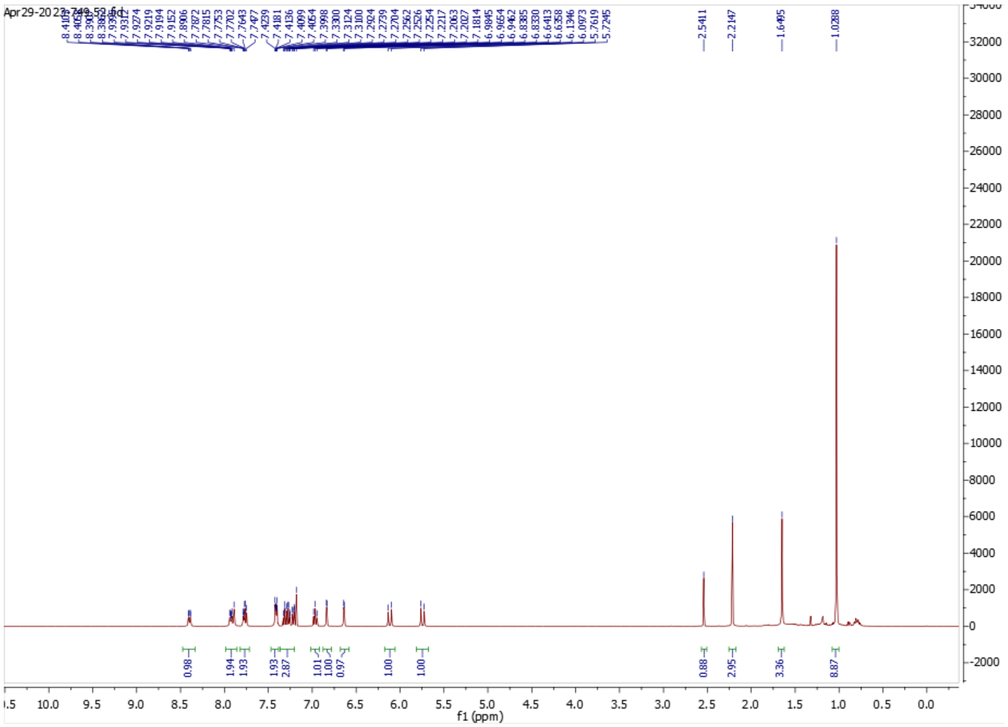

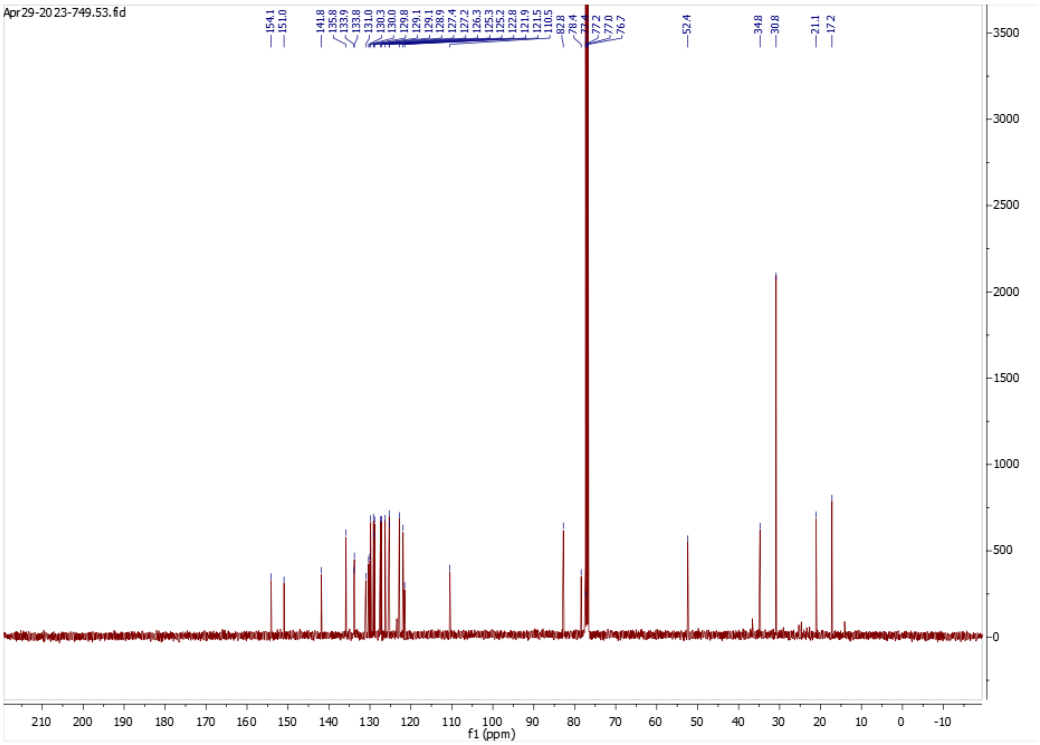

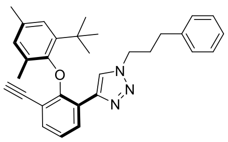

O
N
N
N

3z

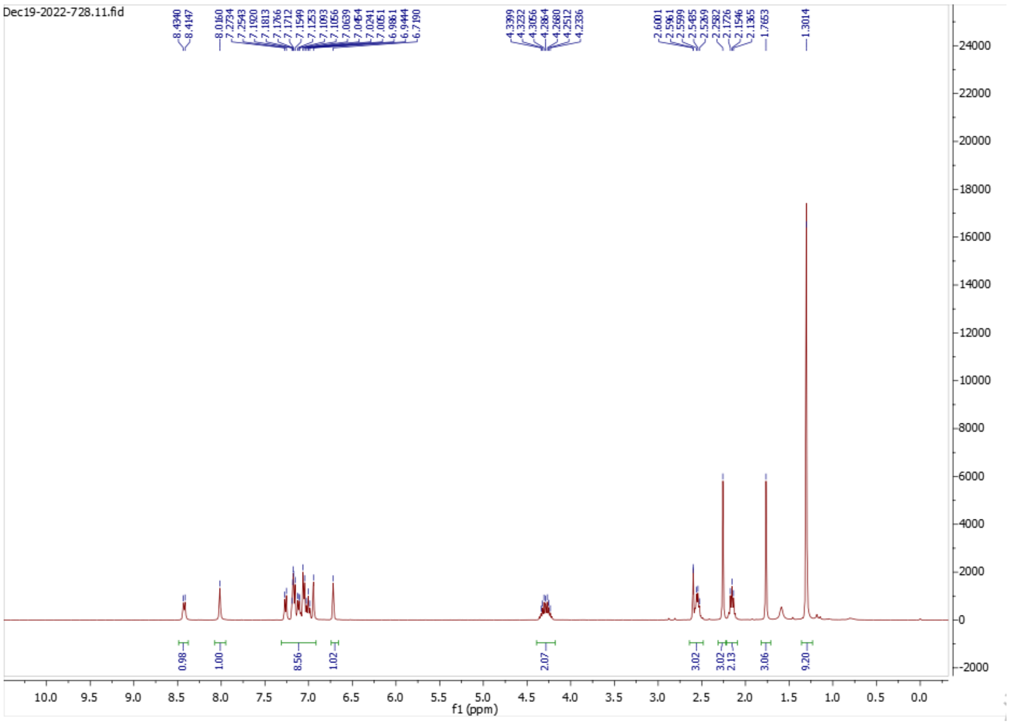

Dec19-2022-728.11.fid
8.4340
8.4147
8.0160
7.2734
7.2543
7.1920
7.1813
7.1766
7.1712
7.1549
7.1253
7.1093
7.1056
7.0639
7.0454
7.0241
7.0051
6.9861
6.9444
6.7190
4.3399
4.3232
4.3056
4.2864
4.2680
4.2512
4.2336
2.6001
2.5961
2.5599
2.5435
2.5269
2.2382
2.1726
2.1546
2.1365
1.7663
1.3014
0.98
1.00
8.56
1.02
2.07
3.02
3.02
2.13
3.06
9.20
f1 (ppm)

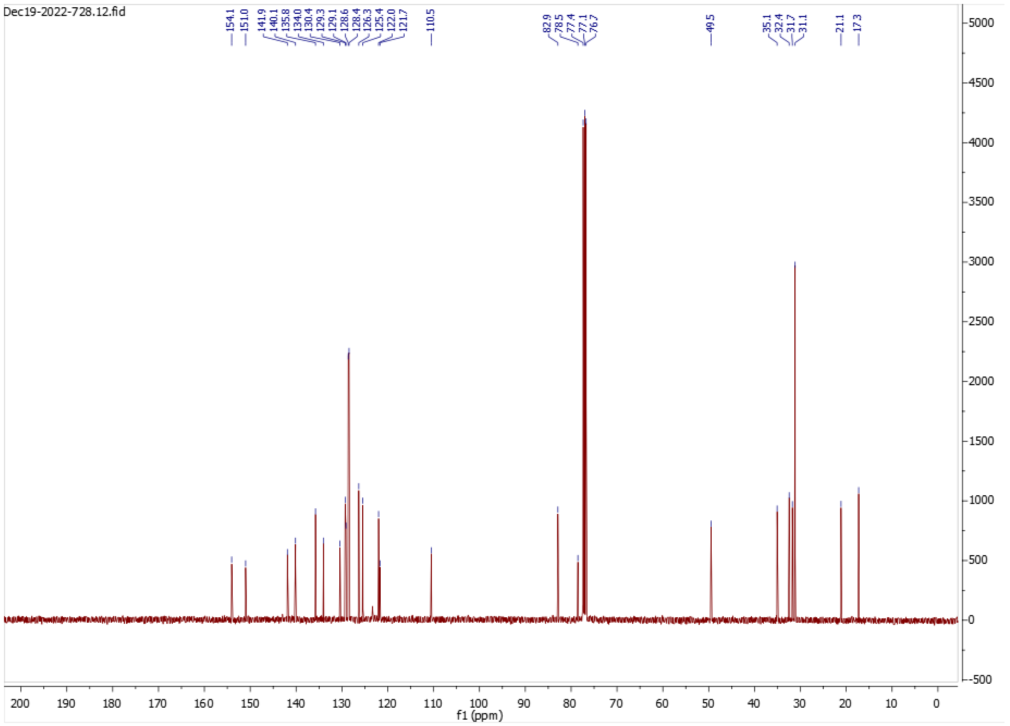

Dec19-2022-728.12.fid
154.1
151.0
141.9
140.1
135.8
134.0
130.4
129.3
129.1
128.6
128.4
126.3
125.4
122.0
121.7
110.5
82.9
78.5
77.4
77.1
76.7
49.5
35.1
32.4
31.7
31.1
21.1
17.3
f1 (ppm)

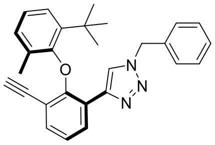
O
N
N
N

3aa

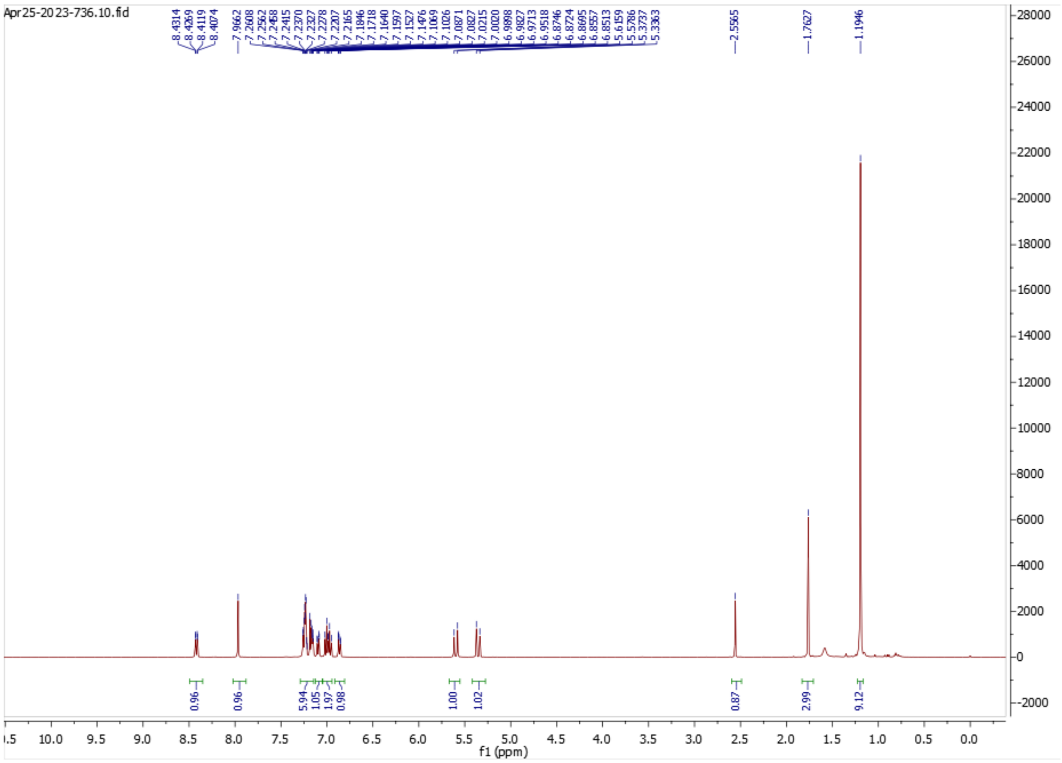
Apr25-2023-736.10.fid
8.4314
8.4269
8.4119
8.4074
7.9662
2.5565
1.7627
1.1946
0.96
0.96
5.94
1.05
1.97
0.98
1.00
1.02
0.87
2.99
9.12
f1 (ppm)

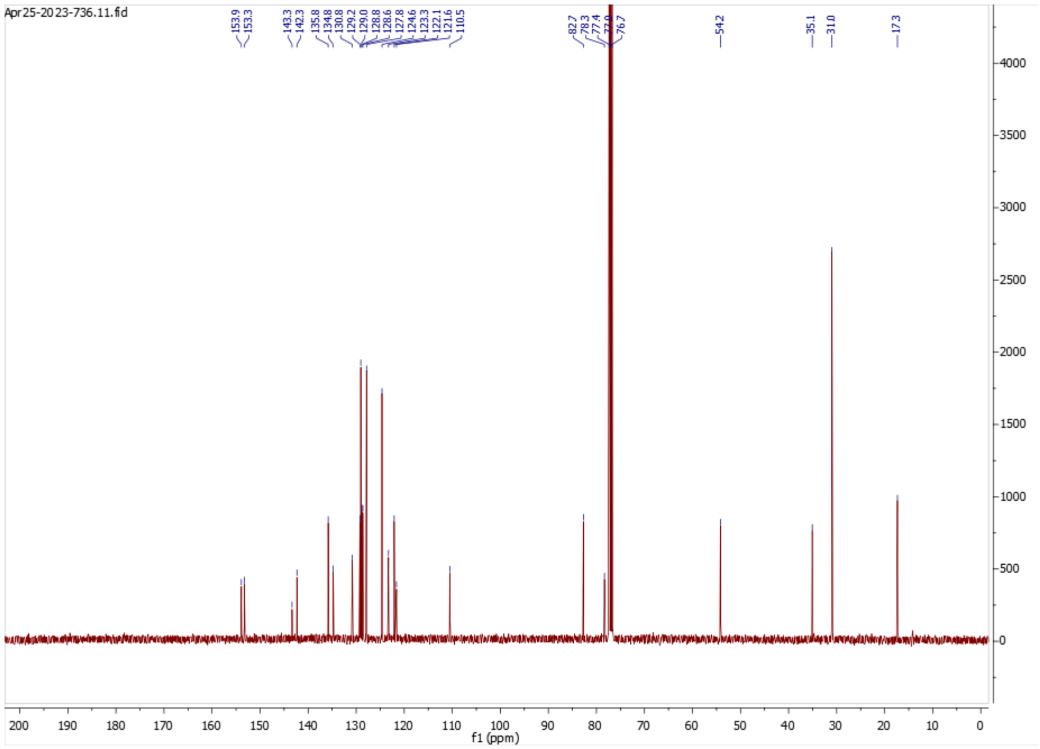
Apr25-2023-736.11.fid
153.9
153.3
143.3
142.3
135.8
134.8
130.8
129.2
129.0
128.8
128.6
127.8
124.6
123.3
122.1
121.6
110.5
82.7
78.3
77.4
77.0
76.7
54.2
35.1
31.0
17.3
f1 (ppm)

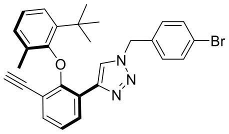
O
N
N
N
Br

3ab

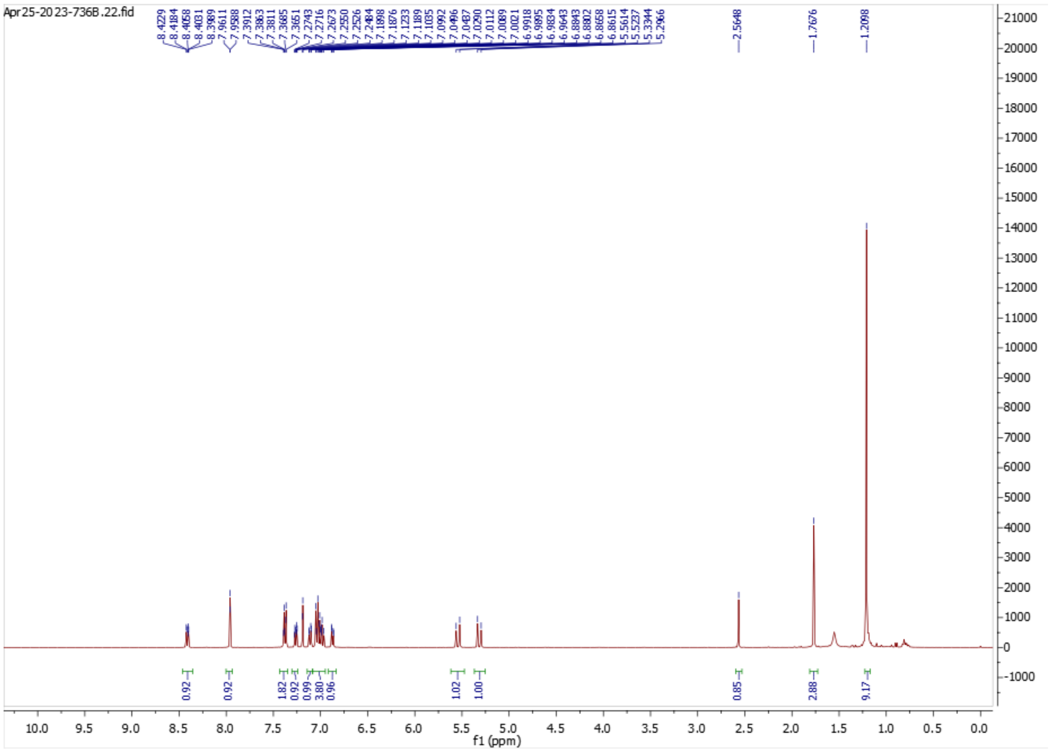
Apr25-2023-736B.22.fid
8.4229
8.4184
8.4058
8.4031
8.3989
7.9611
7.9588
7.3912
7.3863
7.3811
7.3685
7.3651
7.2743
7.2716
7.2673
7.2550
7.2526
7.2484
7.1998
7.1876
7.1189
7.1233
7.1035
7.0992
7.0496
7.0437
7.0290
7.0112
7.0089
7.0021
6.9918
6.9895
6.9834
6.9643
6.8943
6.8802
6.8658
6.8615
5.5614
5.5237
5.3344
5.2966
2.5648
1.7676
1.2098
0.92
0.92
1.82
0.92
0.99
3.80
0.96
1.02
1.00
0.85
2.88
9.17
f1 (ppm)

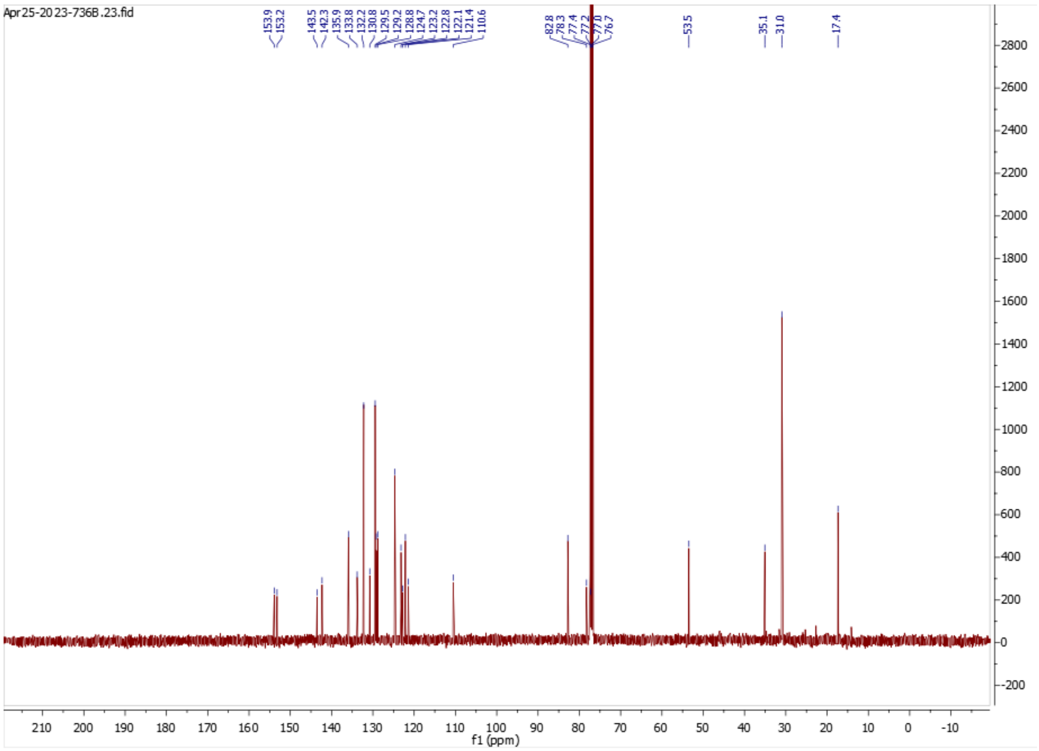
Apr25-2023-736B.23.fid
153.9
153.2
143.5
142.3
135.9
133.8
132.2
130.8
129.5
129.2
128.8
124.7
123.2
122.8
122.1
121.4
110.6
82.8
78.3
77.4
77.2
77.0
76.7
53.5
35.1
31.0
17.4
f1 (ppm)

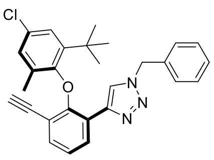
Cl
O
N
N
N

3ac

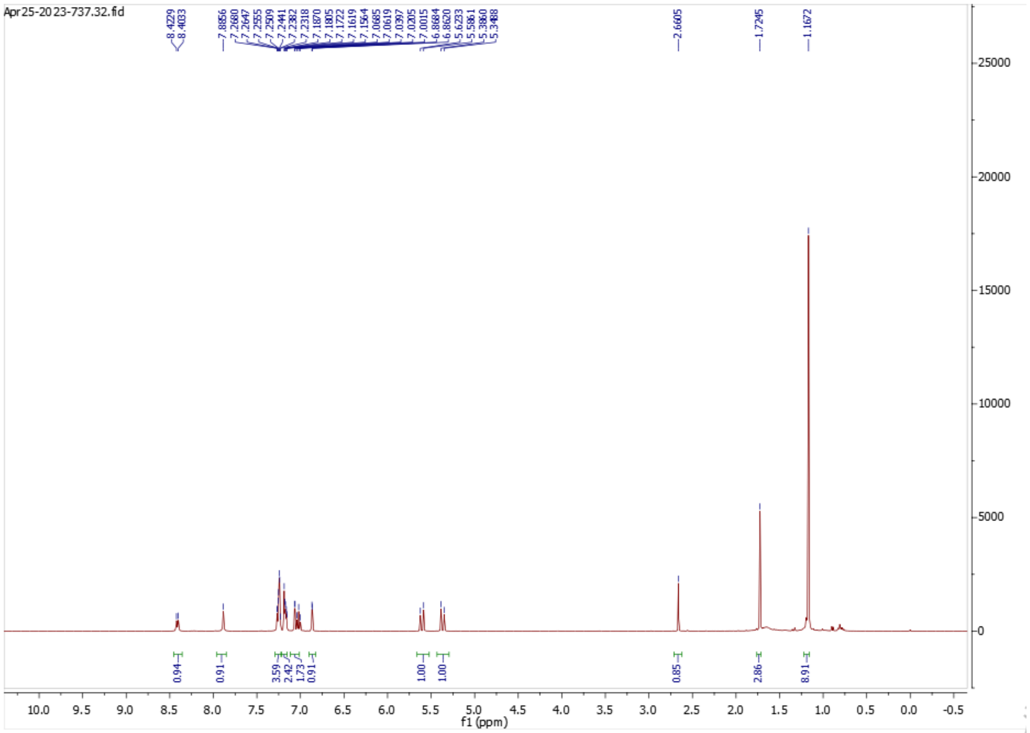
Apr25-2023-737.32.fid
8.4229
8.4033
7.8856
2.6605
1.7245
1.1672
0.94
0.91
3.59
2.42
1.73
0.91
1.00
1.00
0.85
2.86
8.91
f1 (ppm)

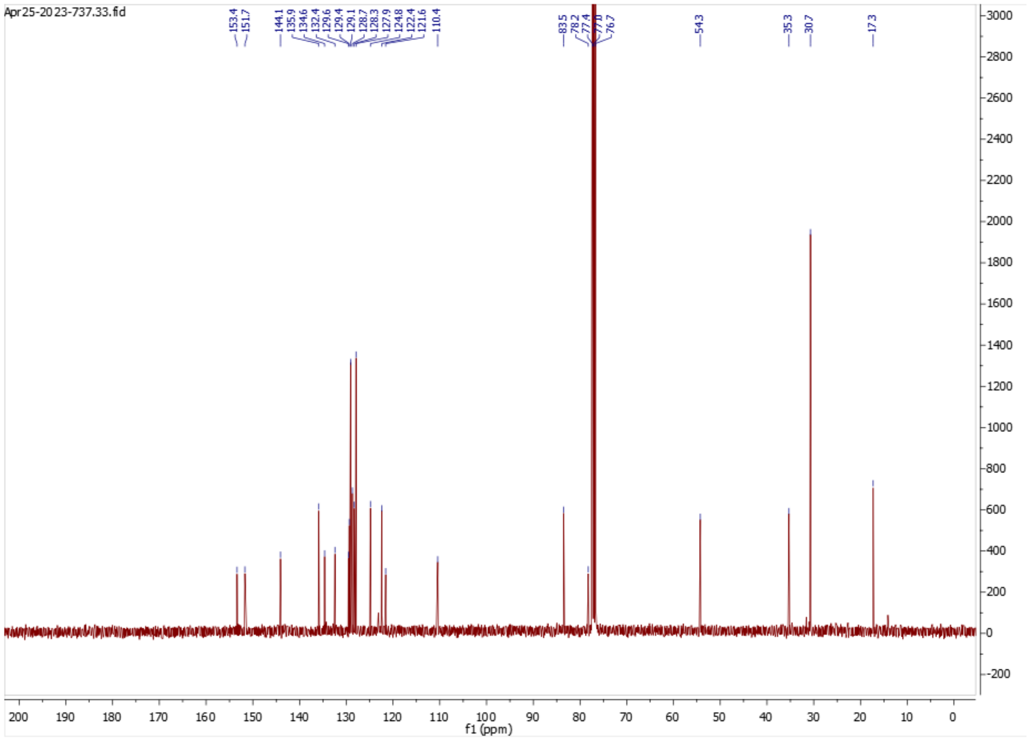
Apr25-2023-737.33.fid
153.4
151.7
144.1
135.9
134.6
132.4
129.6
129.4
129.1
128.7
128.3
127.9
124.8
122.4
121.6
110.4
83.5
78.2
77.4
77.0
76.7
54.3
35.3
30.7
17.3
f1 (ppm)

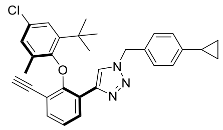

3ad

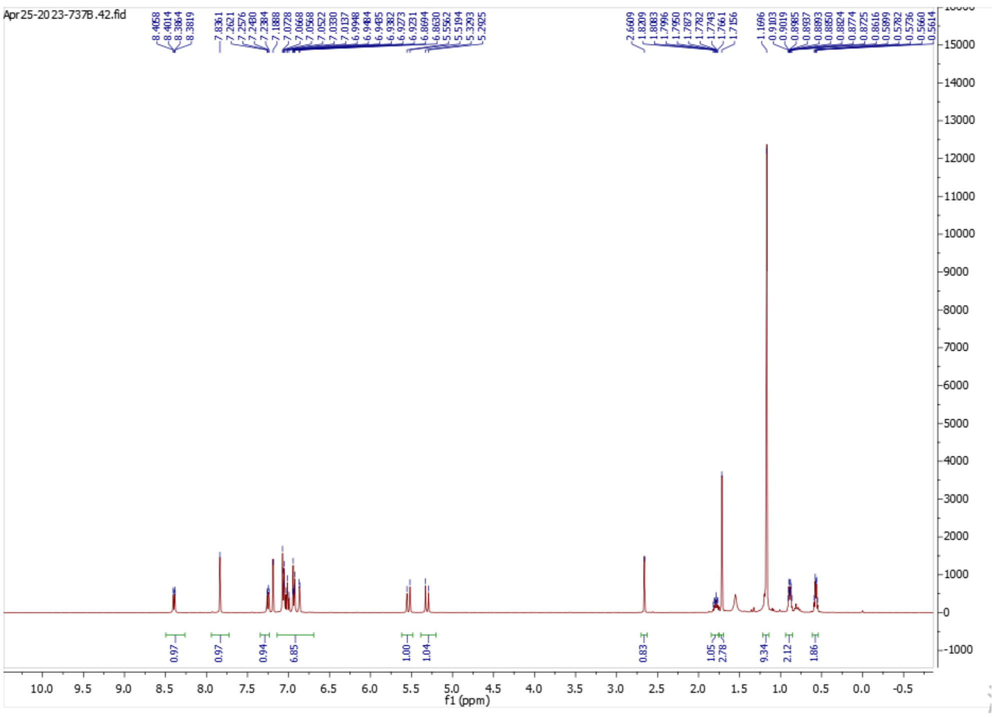

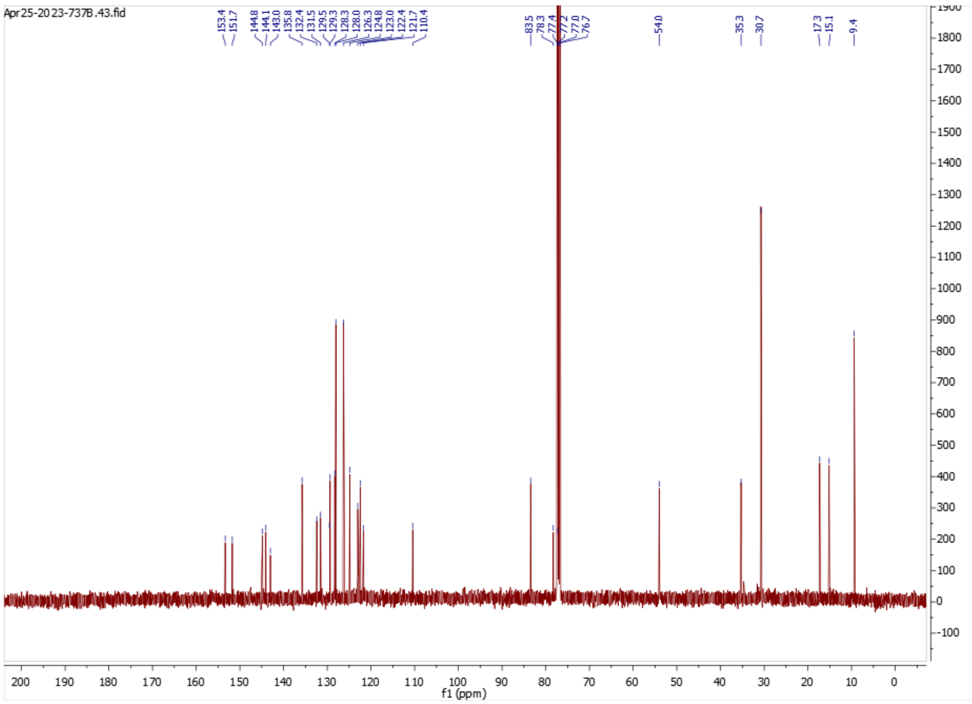

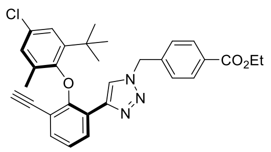
Cl
O
N
N
N
CO2Et

3ae

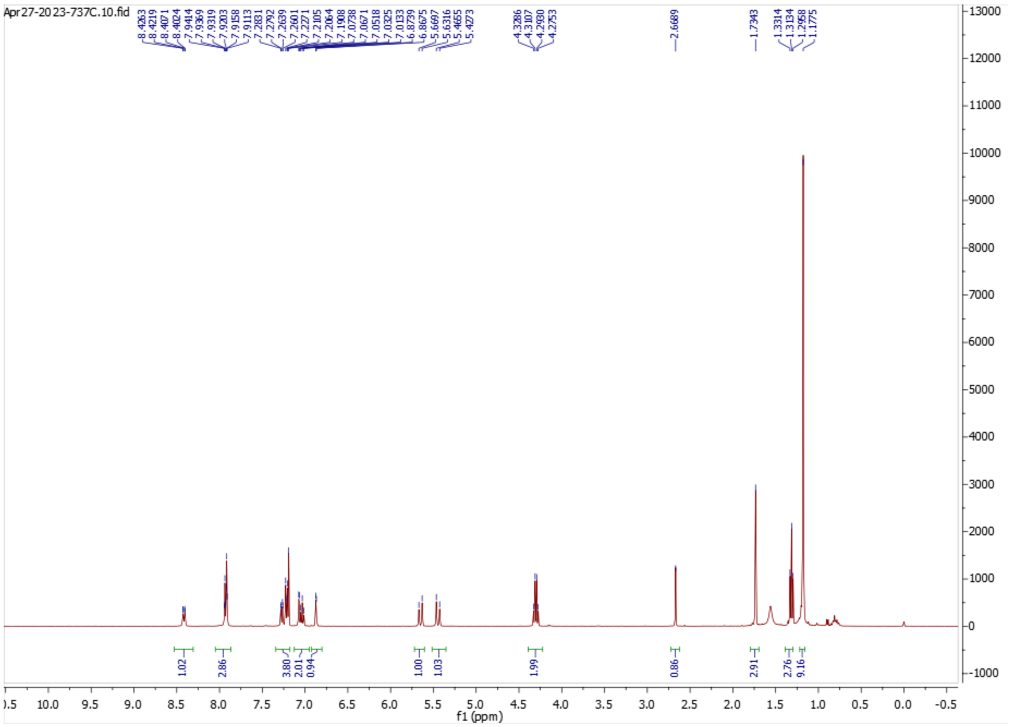
Apr27-2023-737C.10.fid
f1 (ppm)

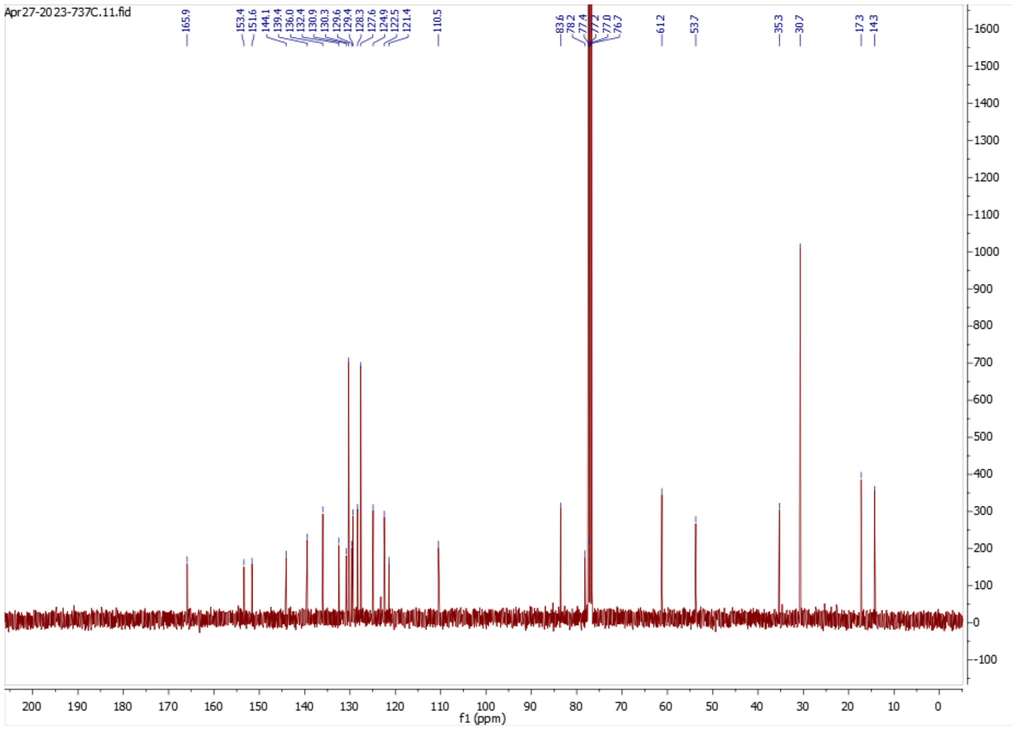
Apr27-2023-737C.11.fid
f1 (ppm)

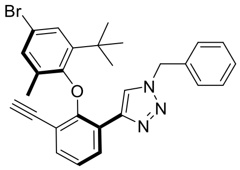

3af

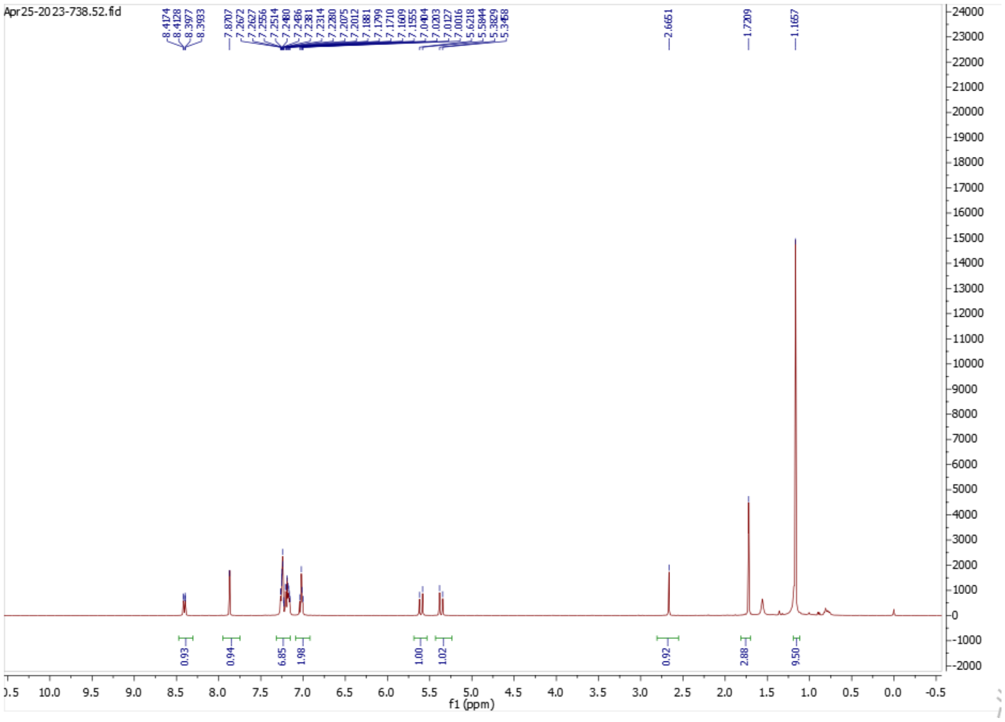

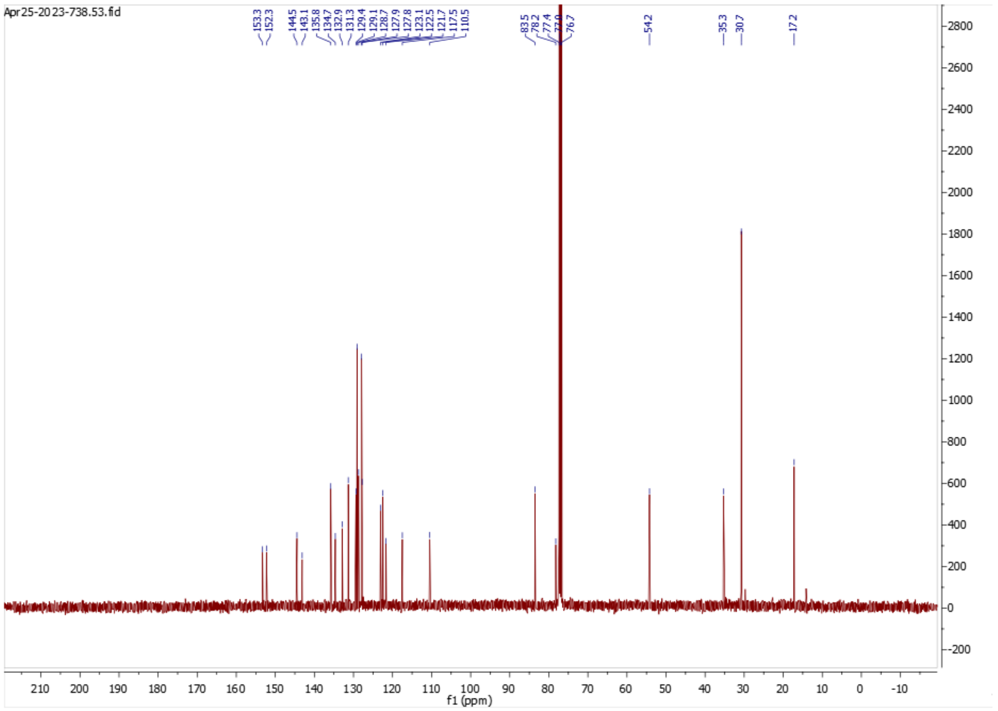

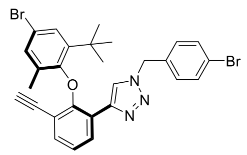
Br
O
N
N
N
Br

3ag

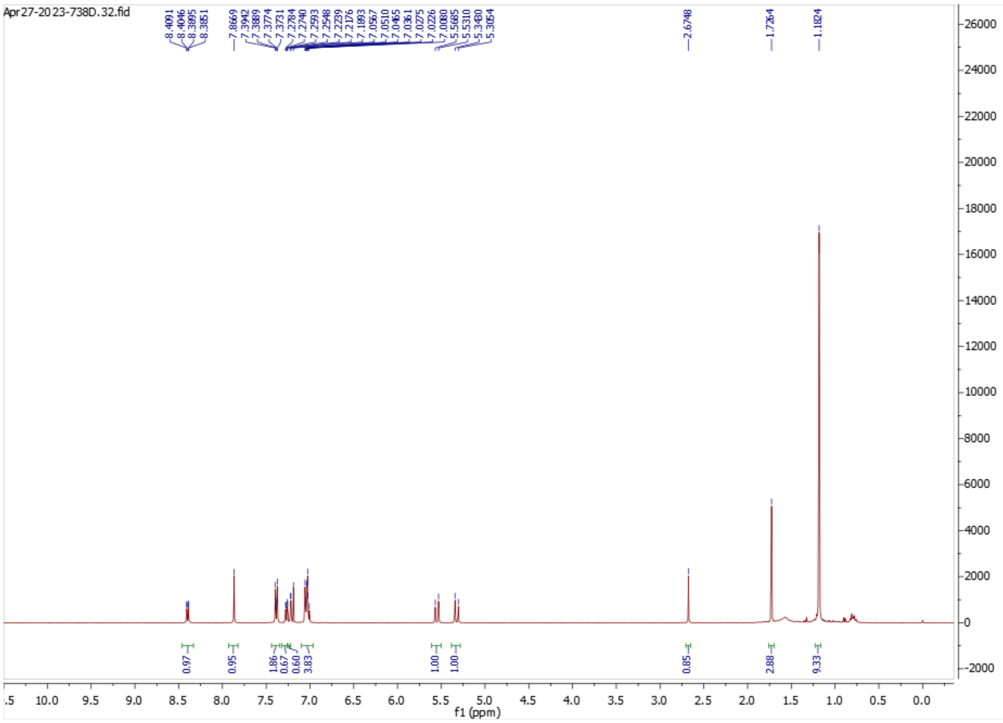
Apr27-2023-738D.32.fid
8.4091
8.4046
8.3895
8.3851
7.8669
7.3942
7.3889
7.3774
7.3721
7.2784
7.2740
7.2593
7.2548
7.2239
7.2176
7.1893
7.0567
7.0510
7.0465
7.0361
7.0275
7.0226
7.0080
5.5685
5.5310
5.3430
5.3054
2.6748
1.7264
1.1824
0.97
0.95
1.86
0.67
0.60
3.83
1.00
1.00
0.85
2.88
9.33
f1 (ppm)

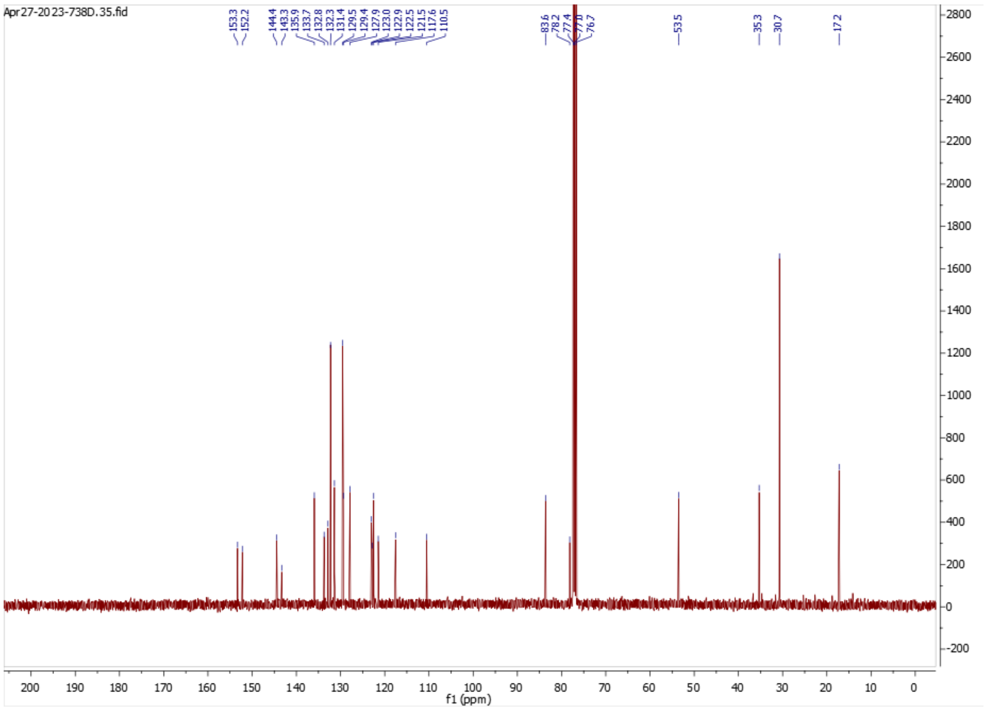
Apr27-2023-738D.35.fid
153.3
152.2
144.4
143.3
135.9
133.7
132.8
132.3
131.4
129.5
129.4
127.9
123.0
122.9
122.5
121.5
117.6
110.5
83.6
78.2
77.4
77.0
76.7
53.5
35.3
30.7
17.2
f1 (ppm)

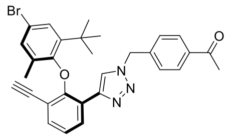
Br
O
N
N
N
O

3ah

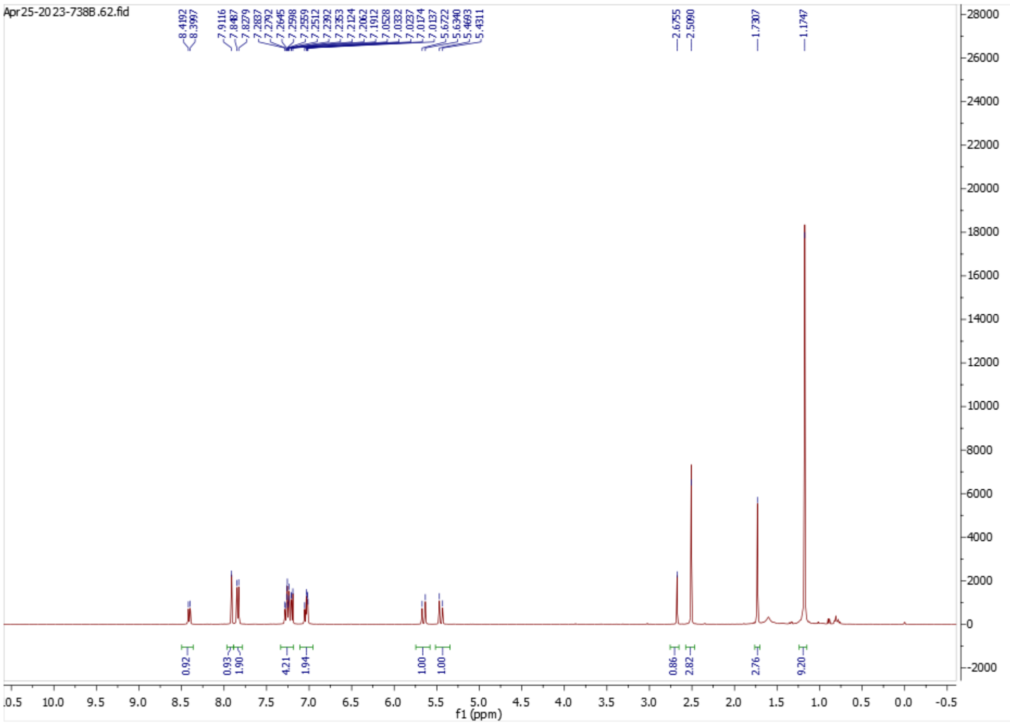
Apr25-2023-738B.62.fid
f1 (ppm)

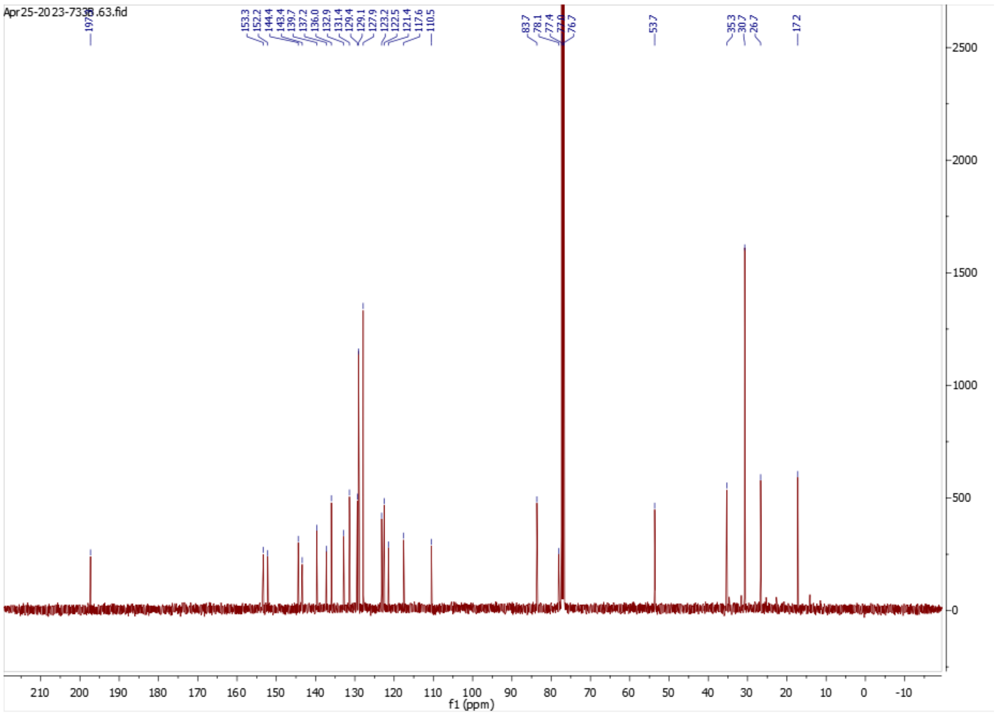
Apr25-2023-738B.63.fid
f1 (ppm)

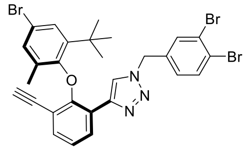
Br
Br
Br
O
N
N
N

3ai

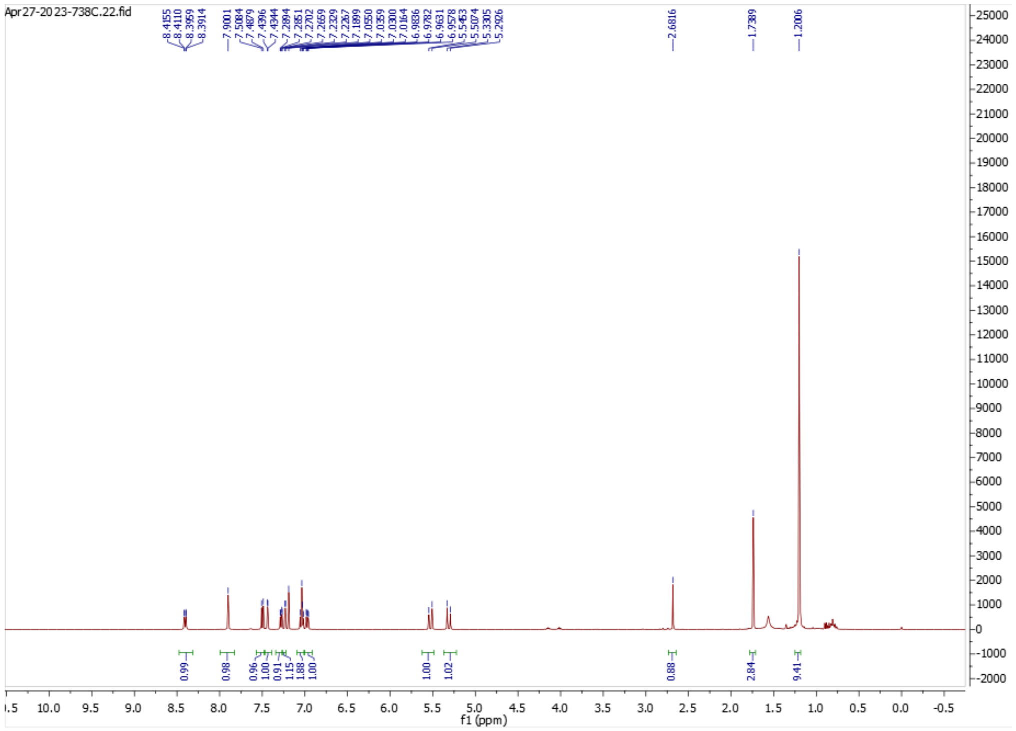
Apr27-2023-738C.22.fid
8.4155
8.4110
8.3959
8.3914
7.9001
7.5084
7.4879
7.4396
7.4344
7.2894
7.2851
7.2702
7.2659
7.2329
7.2267
7.1899
7.0550
7.0359
7.0300
7.0164
6.9836
6.9782
6.9631
6.9578
5.5463
5.5074
5.3305
5.2926
2.6816
1.7389
1.2006
0.99
0.98
0.96
1.00
0.91
1.15
1.88
1.00
1.00
1.02
0.88
2.84
9.41
f1 (ppm)

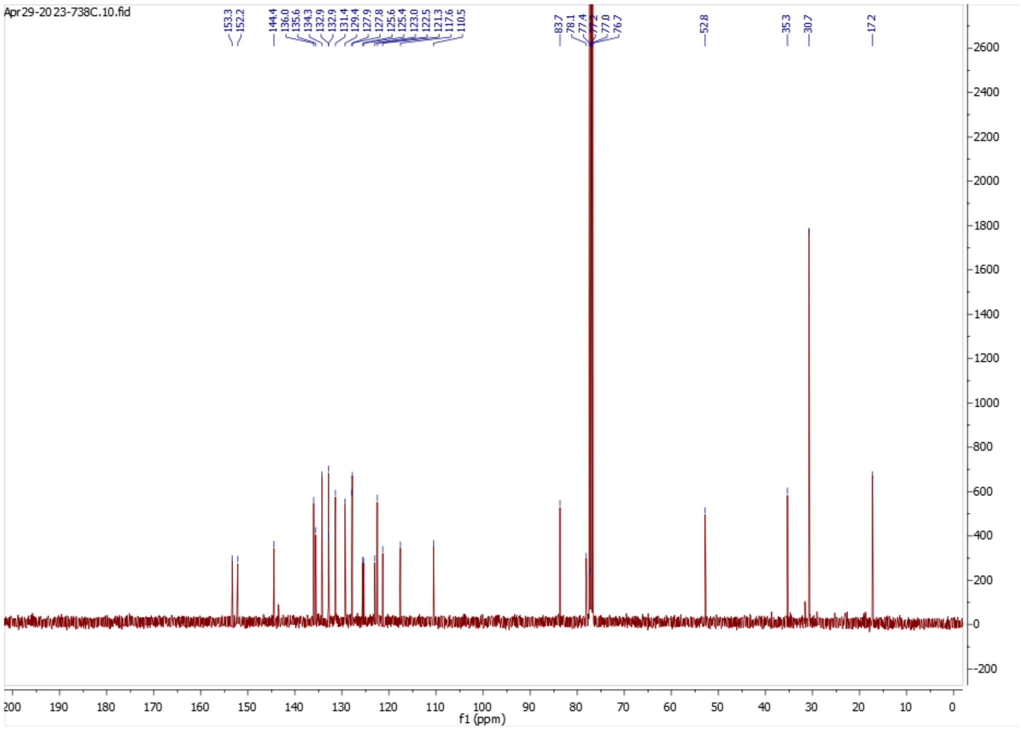
Apr29-2023-738C.10.fid
153.3
152.2
144.4
136.0
135.6
134.3
132.9
131.4
129.4
127.9
127.8
125.6
125.4
123.0
122.5
121.3
117.6
110.5
83.7
78.1
77.4
77.2
77.0
76.7
52.8
35.3
30.7
17.2
f1 (ppm)

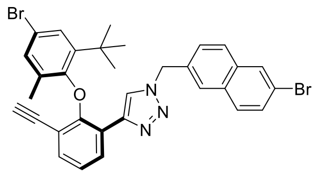

3aj

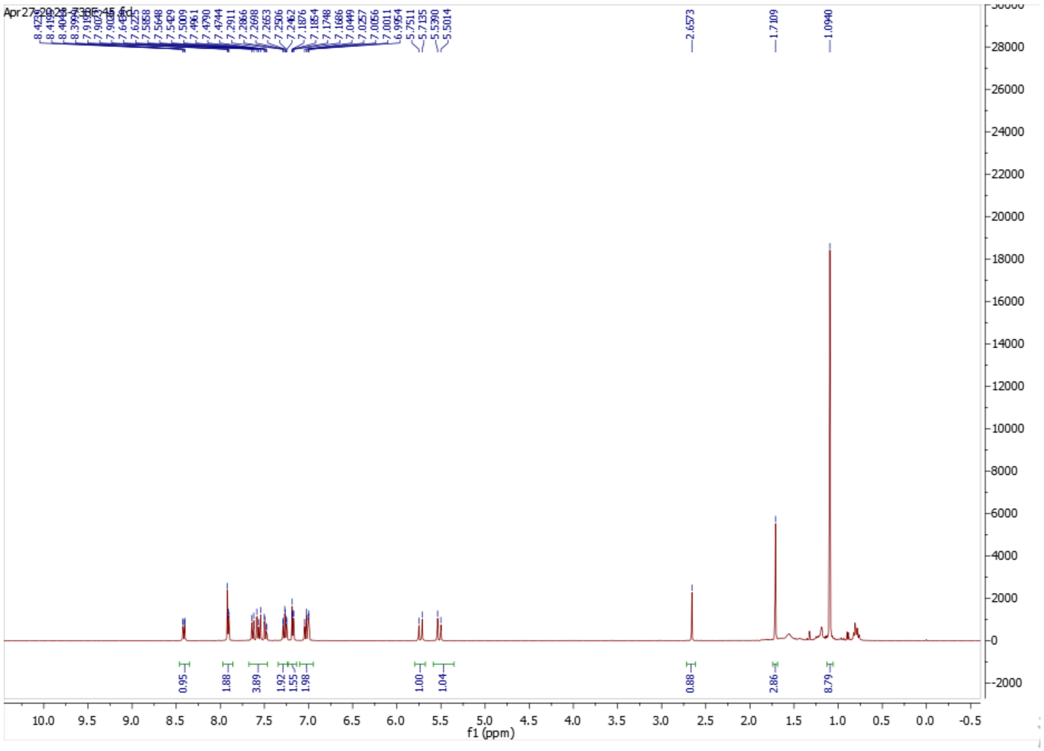

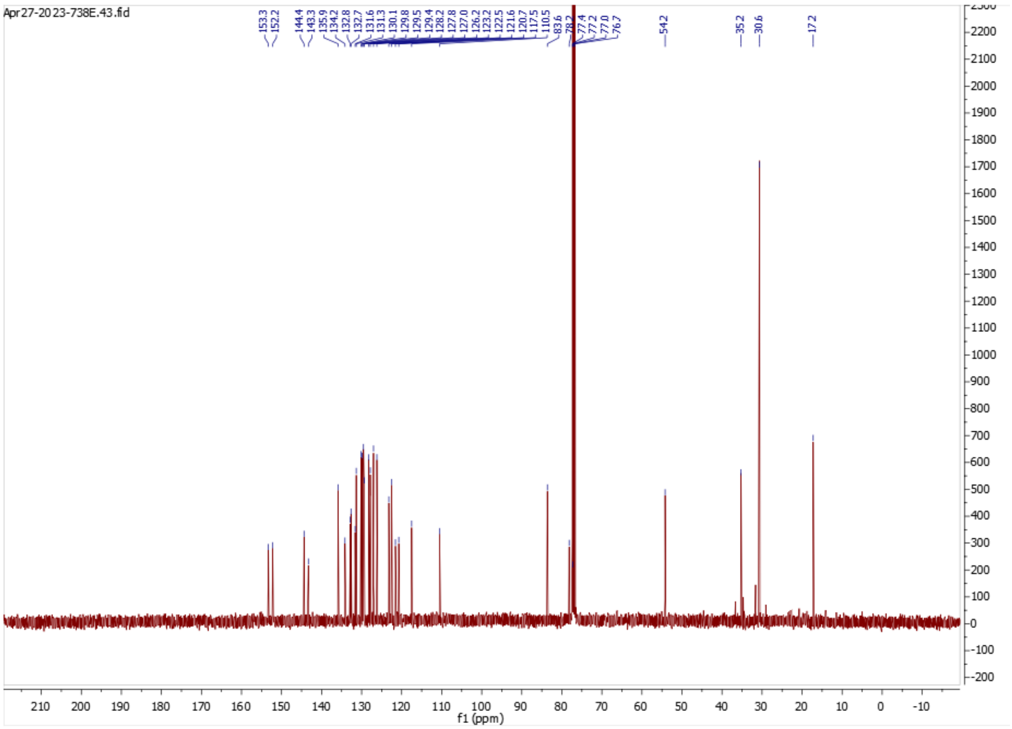

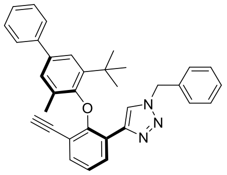

O
N
N
N

3ak

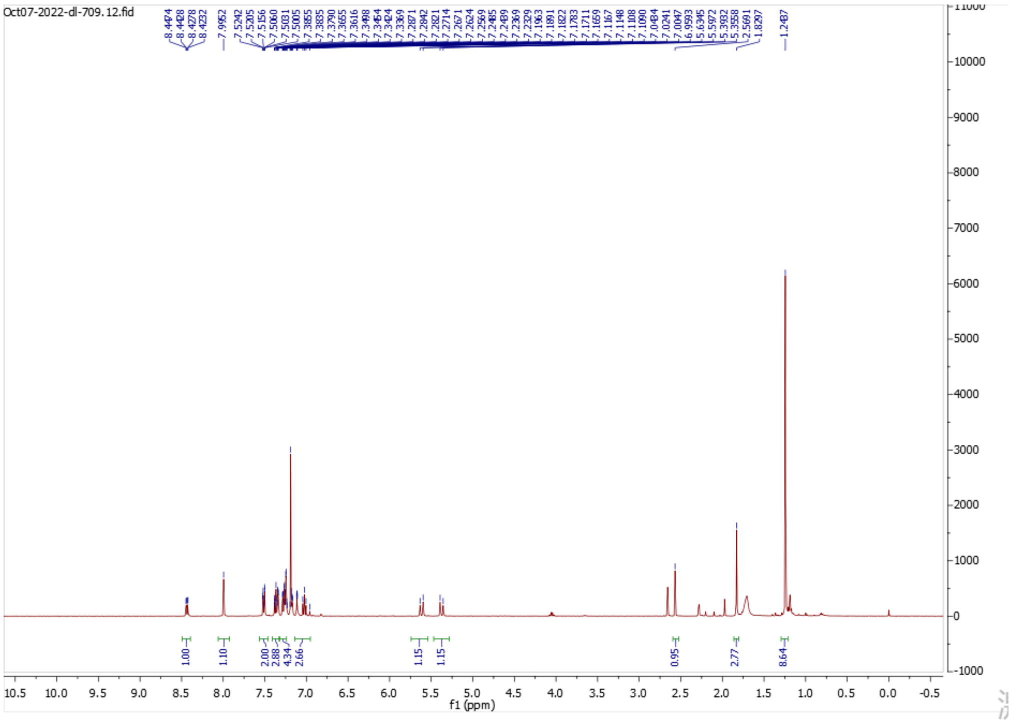

Oct07-2022-dl-709.12.fid
8.4474 8.4438 8.4278 8.4232 7.9952 7.5242 7.5205 7.5156 7.5060 7.5031 7.5005 7.3885 7.3835 7.3790 7.3655 7.3616 7.3498 7.3464 7.3404 7.3369 7.2871 7.2842 7.2821 7.2714 7.2671 7.2624 7.2569 7.2485 7.2459 7.2369 7.2329 7.1963 7.1891 7.1822 7.1783 7.1711 7.1659 7.1167 7.1148 7.1108 7.1090 7.0484 7.0241 7.0047 6.9993 5.6345 5.5972 5.3932 5.3558 2.5691 1.8297 1.2487
1.00 1.10 2.00 2.88 4.34 2.66 1.15 1.15 0.95 2.77 8.64
f1 (ppm)

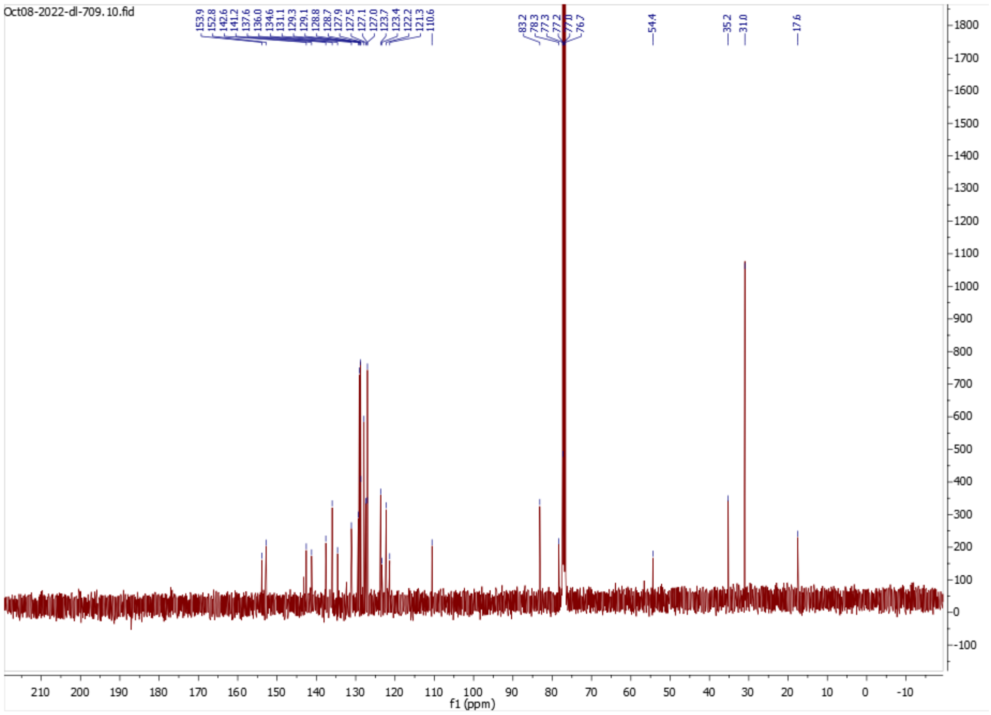

Oct08-2022-dl-709.10.fid
153.9 152.8 142.6 141.2 137.6 136.0 134.6 131.1 129.3 129.1 128.8 128.7 127.9 127.5 127.1 127.0 123.7 123.4 122.2 121.3 110.6
83.2 78.3 77.3 77.0 76.7
54.4
35.2 31.0
17.6
f1 (ppm)

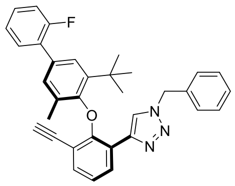

3al

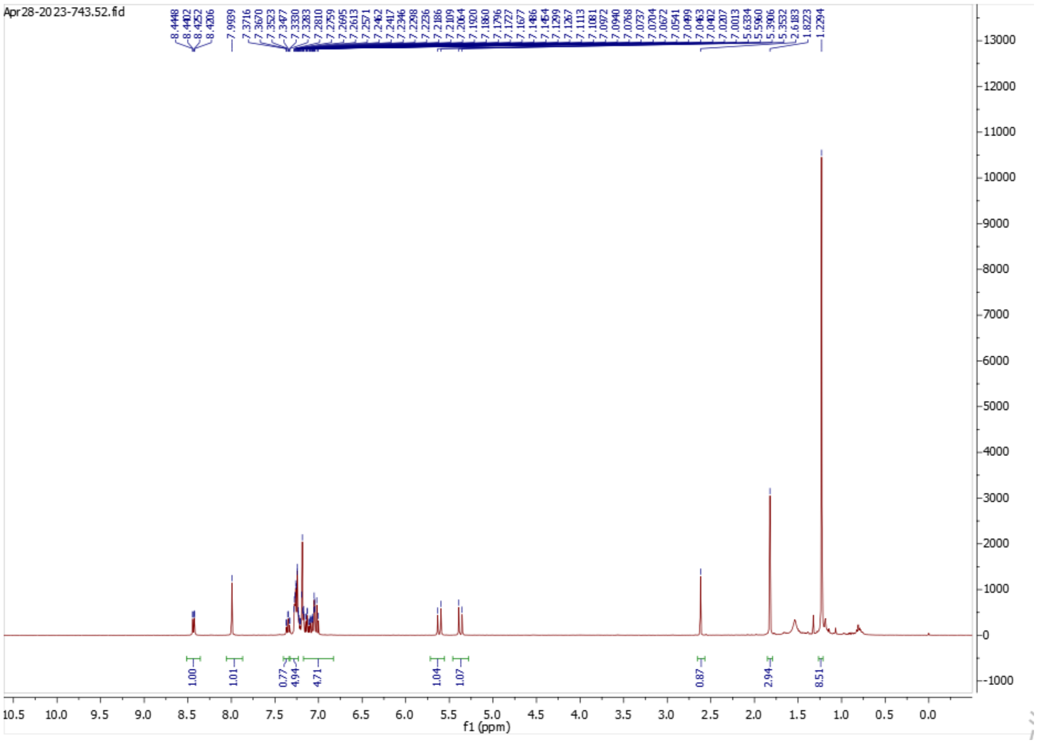

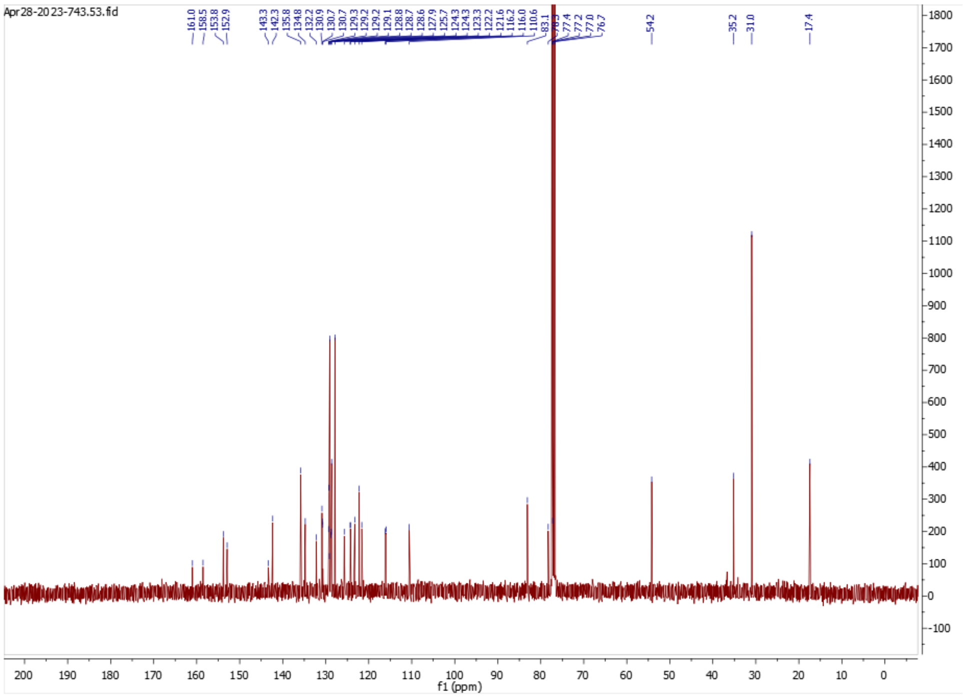

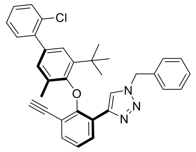
Cl
O
N
N
N

3am

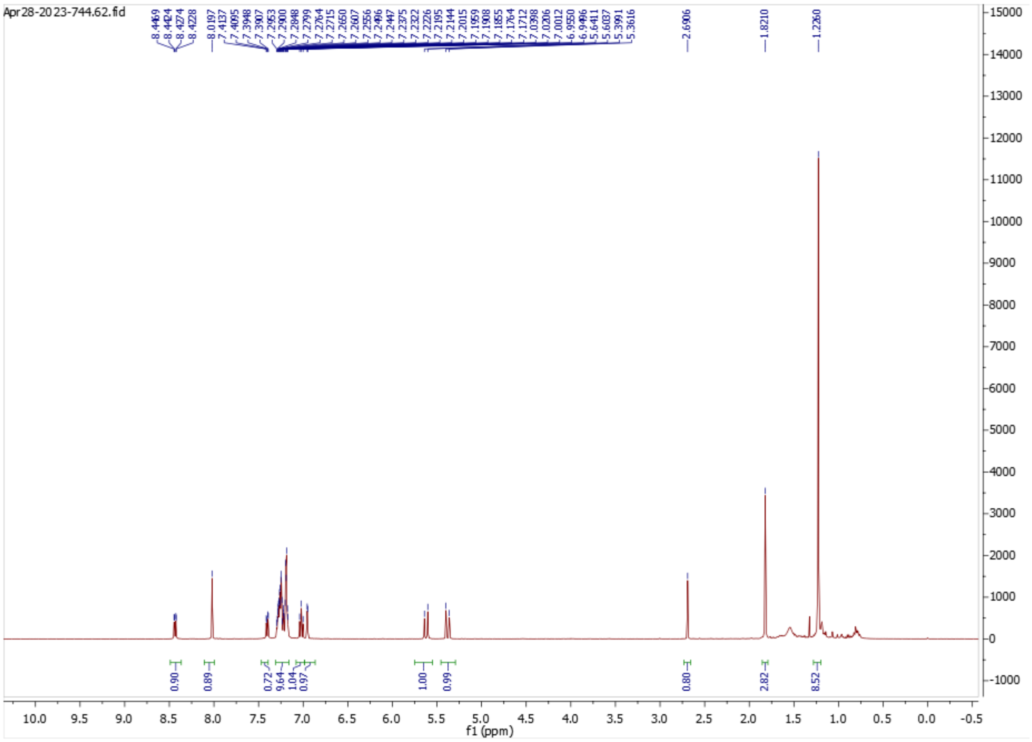
Apr28-2023-744.62.fid
8.4469
8.4424
8.4274
8.4228
8.0187
7.4137
7.4095
7.3948
7.3907
7.2953
7.2900
7.2848
7.2799
7.2764
7.2715
7.2650
7.2607
7.2556
7.2496
7.2447
7.2375
7.2322
7.2226
7.2195
7.2144
7.2015
7.1959
7.1908
7.1855
7.1764
7.1712
7.0398
7.0206
7.0012
6.9550
6.9496
5.6411
5.6037
5.3991
5.3616
2.6906
1.8210
1.2260
0.90
0.89
0.72
9.64
1.04
0.97
1.00
0.99
0.80
2.82
8.52
f1 (ppm)

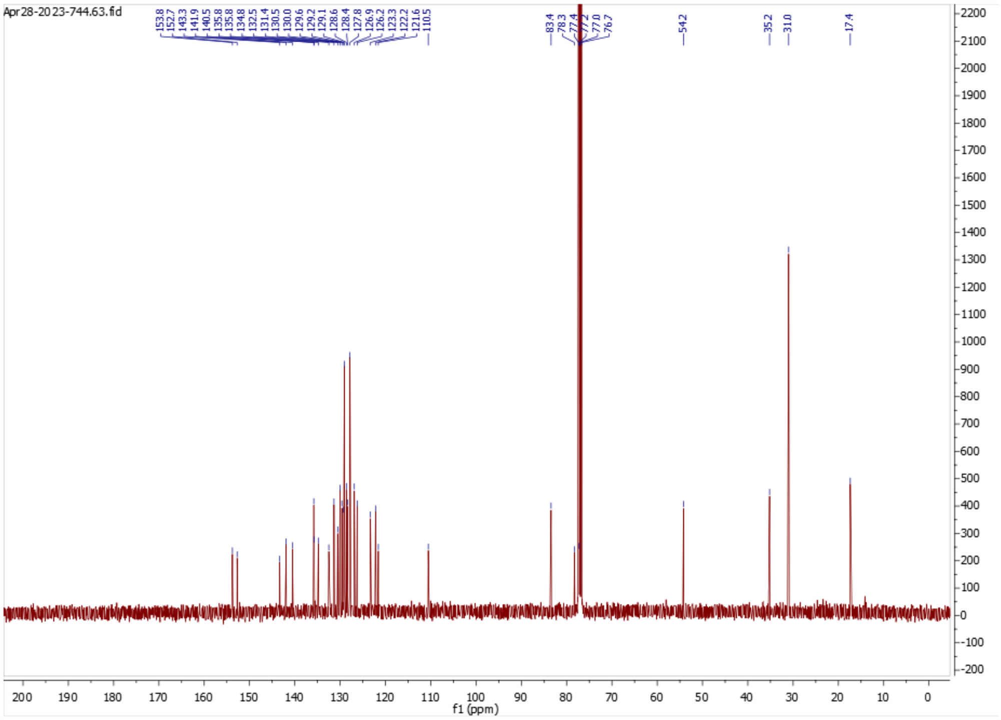
Apr28-2023-744.63.fid
153.8
152.7
143.3
141.9
140.5
135.8
135.8
134.8
132.5
131.4
130.5
130.0
129.6
129.2
129.1
128.6
128.4
127.8
126.9
126.2
123.3
122.2
121.6
110.5
83.4
78.3
77.4
77.2
77.0
76.7
54.2
35.2
31.0
17.4
f1 (ppm)

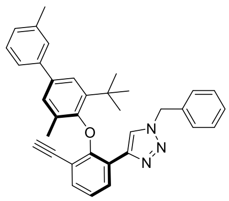

3an

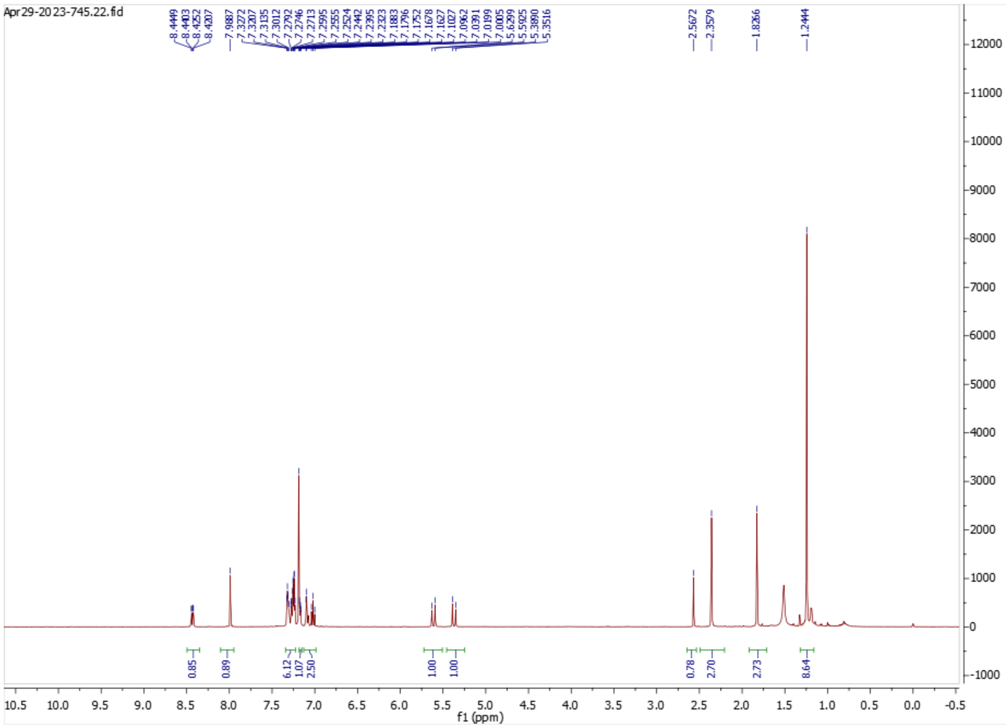

Apr29-2023-745.22.fid
8.4449
8.4403
8.4252
8.4207
7.9887
7.3272
7.3207
7.3135
7.3012
7.2792
7.2746
7.2713
7.2595
7.2555
7.2524
7.2442
7.2395
7.2323
7.1883
7.1796
7.1752
7.1678
7.1627
7.1027
7.0962
7.0391
7.0199
7.0005
5.6299
5.5925
5.3890
5.3516
2.5672
2.3579
1.8266
1.2444
0.85
0.89
6.12
1.07
2.50
1.00
1.00
0.78
2.70
2.73
8.64
f1 (ppm)

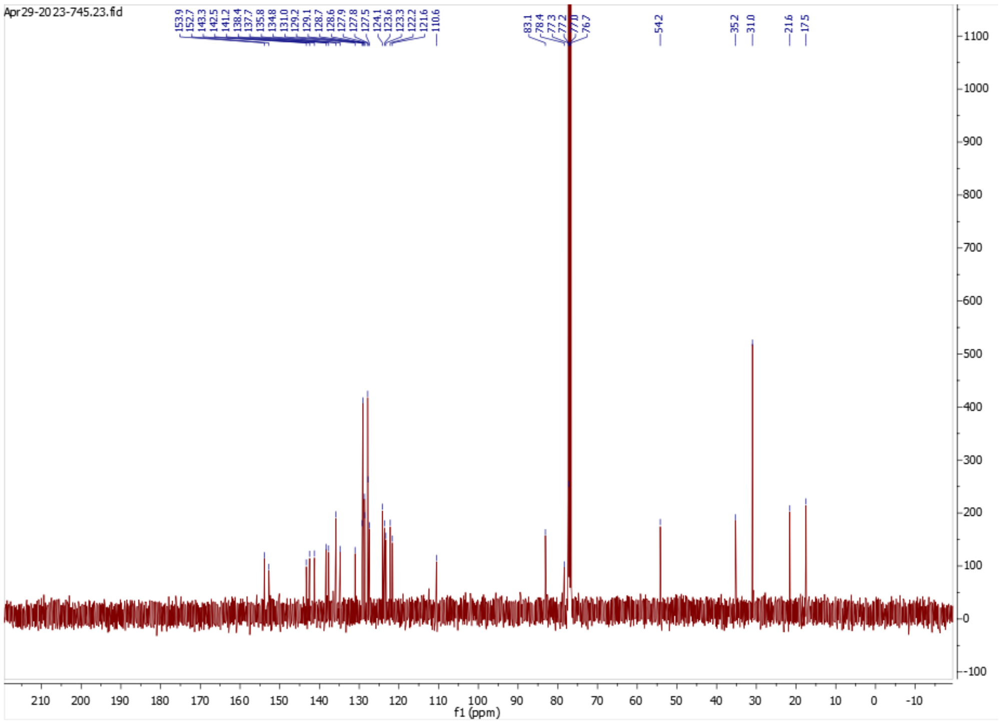

Apr29-2023-745.23.fid
153.9
152.7
143.3
142.5
141.2
138.4
137.7
135.8
134.8
131.0
129.2
129.1
128.7
128.6
127.9
127.8
127.5
124.1
123.6
123.3
122.2
121.6
110.6
83.1
78.4
77.3
77.0
76.7
54.2
35.2
31.0
21.6
17.5
f1 (ppm)

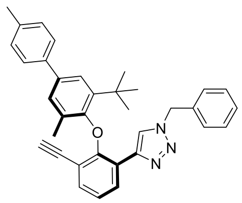

3ao

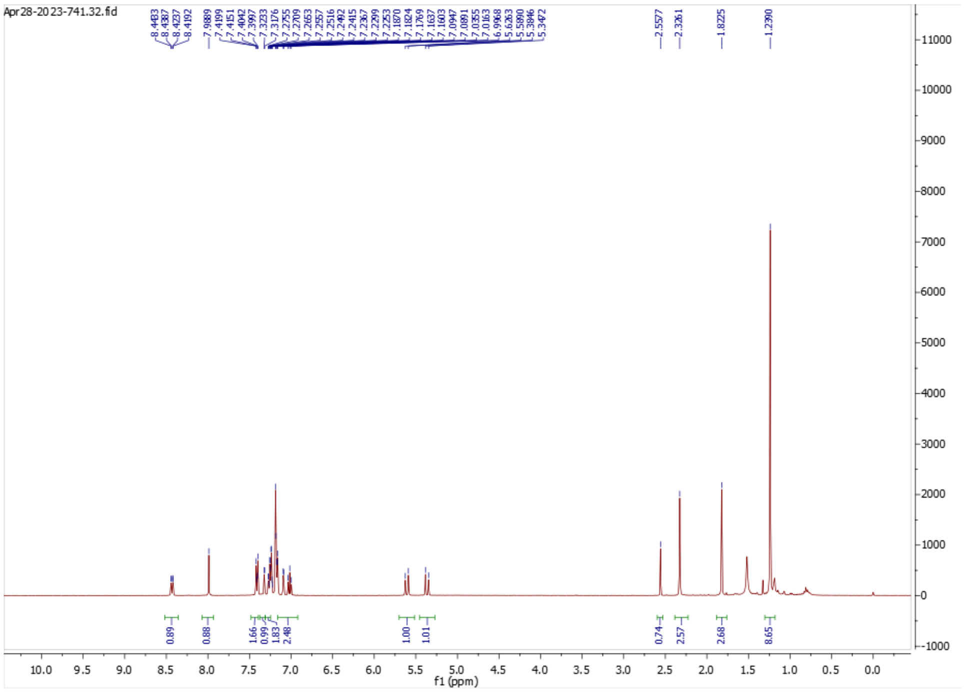

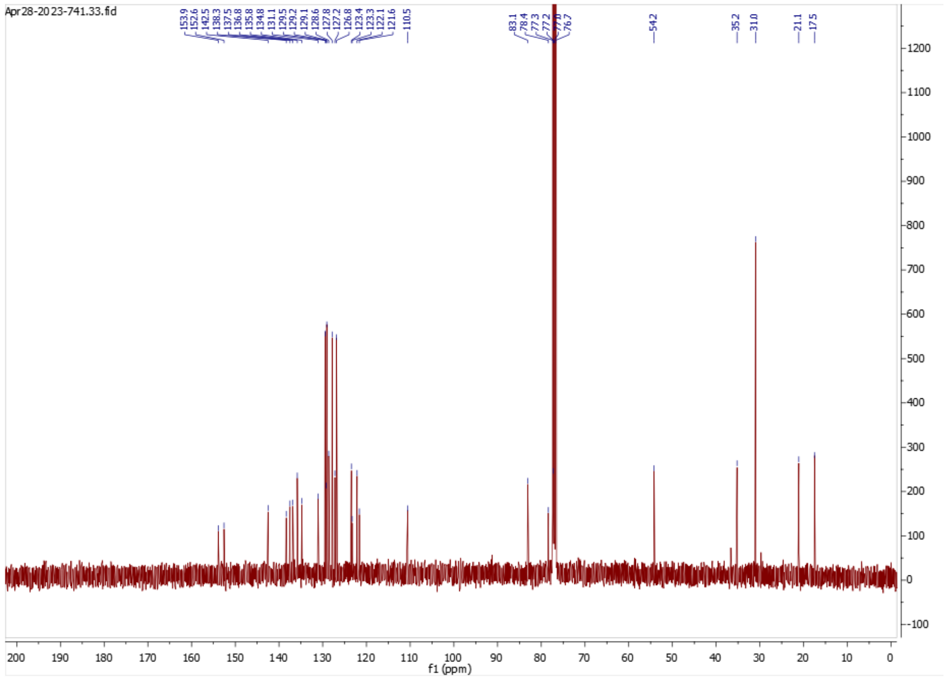

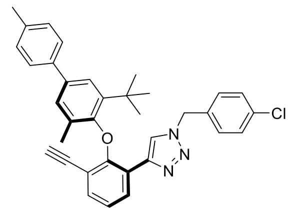

O
N
N
N
Cl

3ap

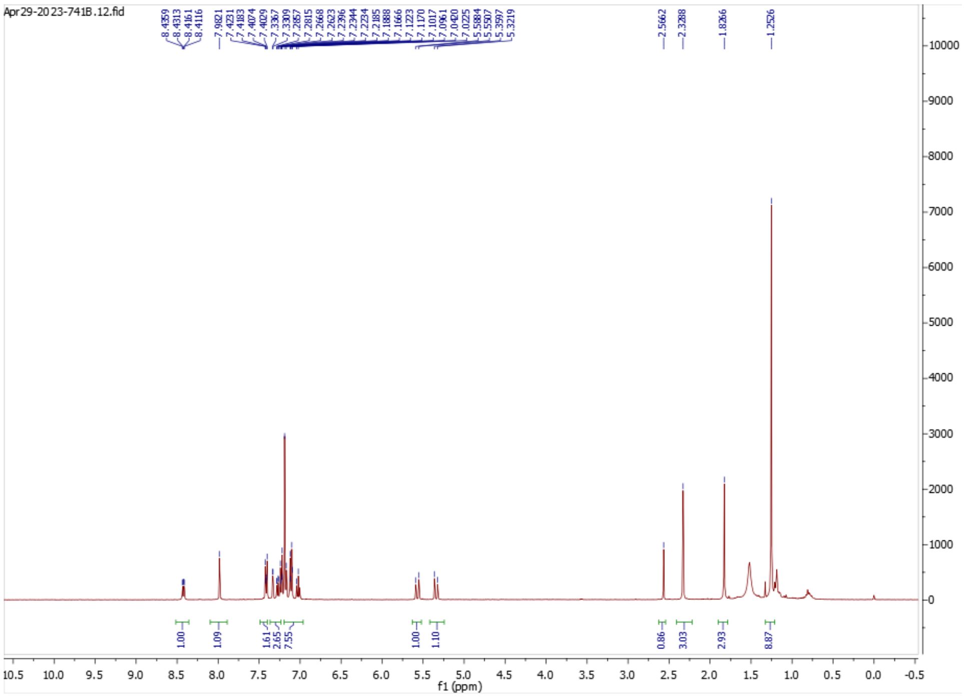

Apr29-2023-741B.12.fid
8.4359 8.4313 8.4161 8.4116 7.9821 7.4231 7.4183 7.4074 7.4029 7.3367 7.3309 7.2857 7.2815 7.2668 7.2623 7.2396 7.2344 7.2234 7.2185 7.1888 7.1666 7.1123 7.1170 7.1017 7.0961 7.0420 7.0225 5.5884 5.5507 5.3997 5.3219
2.5662 2.3288 1.8266 1.2526
1.00 1.09 1.61 2.65 7.55 1.00 1.10 0.86 3.03 2.93 8.87
10.5 10.0 9.5 9.0 8.5 8.0 7.5 7.0 6.5 6.0 5.5 5.0 4.5 4.0 3.5 3.0 2.5 2.0 1.5 1.0 0.5 0.0 -0.5
f1 (ppm)
10000 9000 8000 7000 6000 5000 4000 3000 2000 1000 0

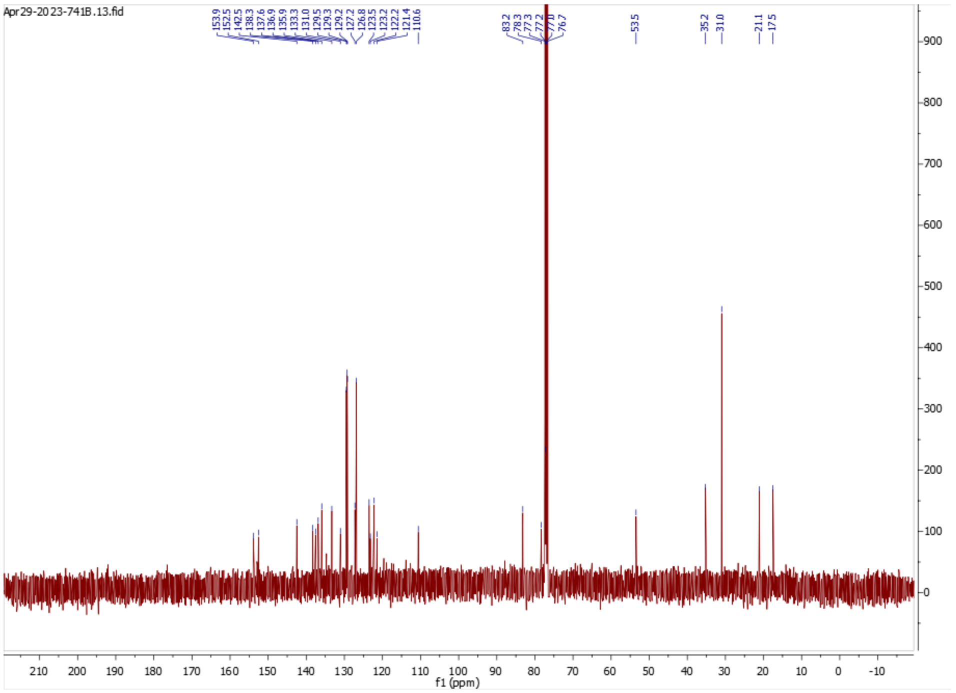

Apr29-2023-741B.13.fid
153.9 152.5 142.5 138.3 137.6 136.9 135.9 133.3 131.0 129.5 129.3 129.2 127.2 126.8 123.5 123.2 122.2 121.4 110.6
83.2 78.3 77.3 77.2 77.0 76.7
53.5
35.2 31.0
21.1 17.5
210 200 190 180 170 160 150 140 130 120 110 100 90 80 70 60 50 40 30 20 10 0 -10
f1 (ppm)
900 800 700 600 500 400 300 200 100 0

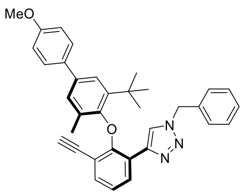
MeO
O
N
N
N

3aq

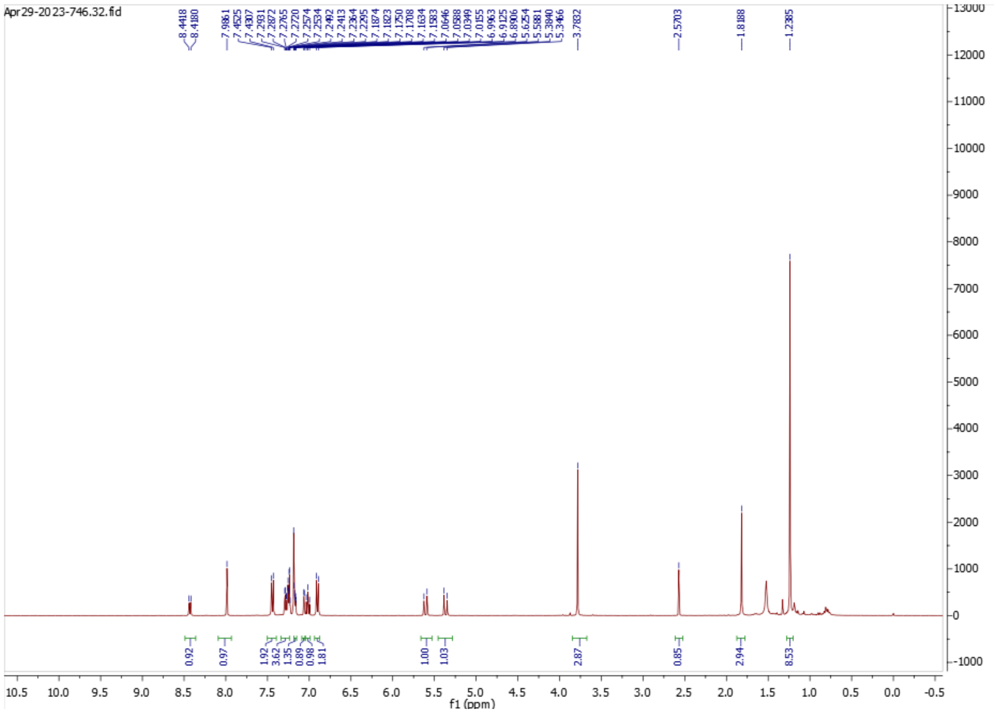
Apr29-2023-746.32.fid
8.4418
8.4180
7.9861
7.4525
7.4307
7.2931
7.2872
7.2765
7.2720
7.2574
7.2534
7.2492
7.2413
7.2364
7.2295
7.1874
7.1823
7.1750
7.1708
7.1634
7.1583
7.0646
7.0588
7.0349
7.0155
6.9363
6.9125
6.8906
5.6254
5.5881
5.3840
5.3466
3.7832
2.5703
1.8188
1.2385
0.92
0.97
1.92
3.62
1.35
0.89
0.98
1.81
1.00
1.03
2.87
0.85
2.94
8.53
f1 (ppm)

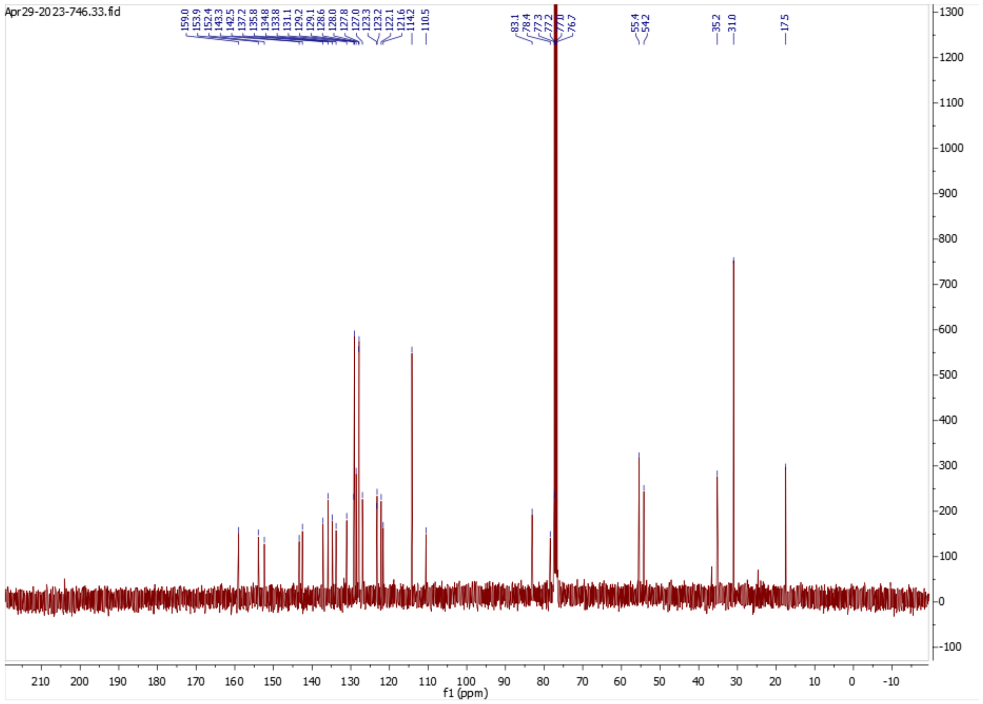
Apr29-2023-746.33.fid
159.0
153.9
152.4
143.3
142.5
137.2
135.8
134.8
133.8
131.1
129.2
129.1
128.6
128.0
127.8
127.0
123.3
123.2
122.1
121.6
114.2
110.5
83.1
78.4
77.3
77.2
77.0
76.7
55.4
54.2
35.2
31.0
17.5
f1 (ppm)

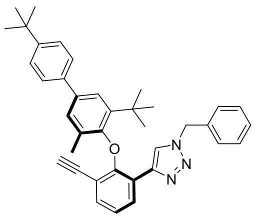

3ar

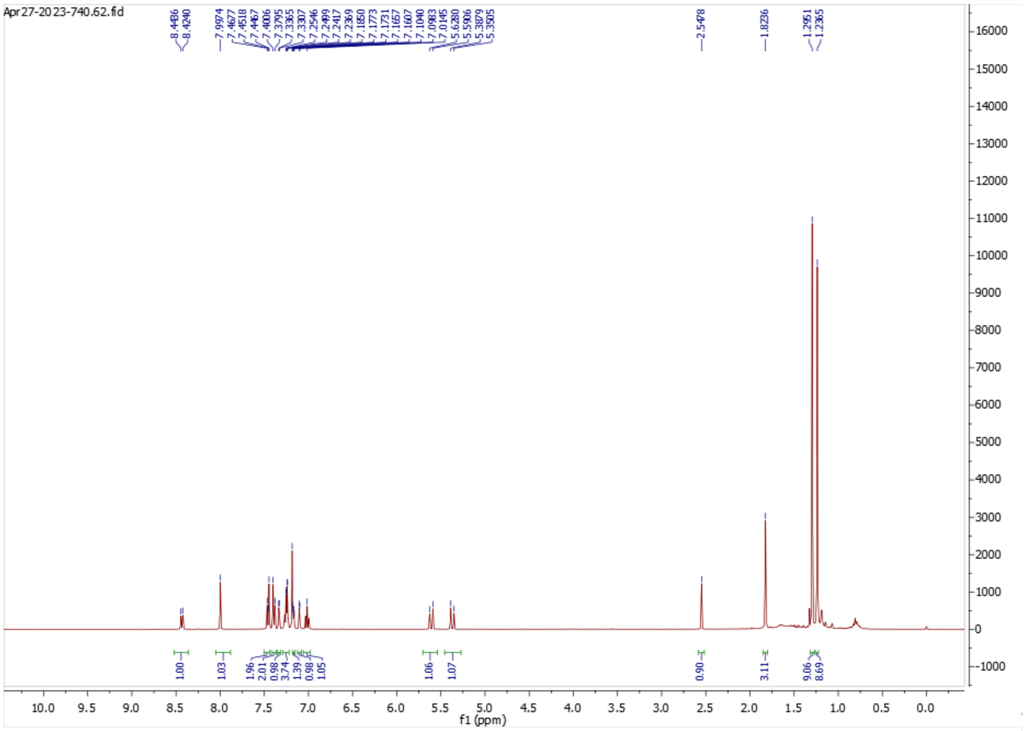
Apr27-2023-740.62.fid
8.4436
8.4240
7.9974
7.4677
7.4518
7.4467
7.4006
7.3795
7.3365
7.3307
7.2546
7.2499
7.2417
7.2369
7.1850
7.1773
7.1731
7.1657
7.1607
7.1040
7.0983
7.0145
5.6280
5.5906
5.3879
5.3505
2.5478
1.8236
1.2951
1.2365
1.00
1.03
1.96
2.01
0.98
3.74
1.39
0.98
1.05
1.06
1.07
0.90
3.11
9.06
8.69
f1 (ppm)

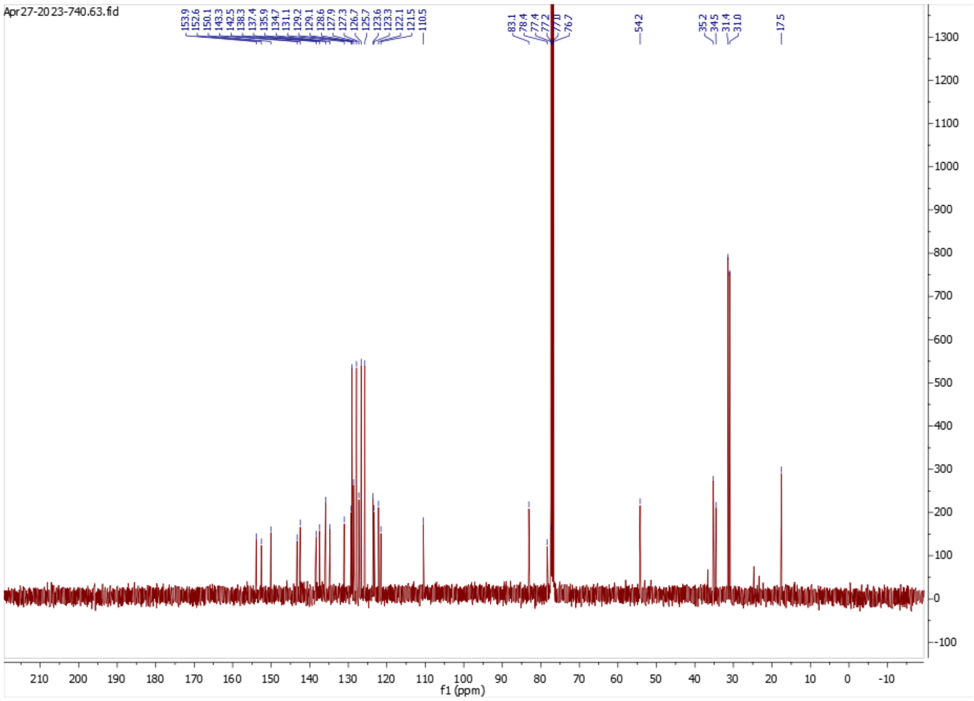
Apr27-2023-740.63.fid
153.9
152.6
150.1
143.3
142.5
138.3
137.4
135.9
134.7
131.1
129.2
129.1
128.6
127.9
127.3
126.7
125.7
123.6
123.3
122.1
121.5
110.5
83.1
78.4
77.4
77.2
77.0
76.7
54.2
35.2
34.5
31.4
31.0
17.5
f1 (ppm)

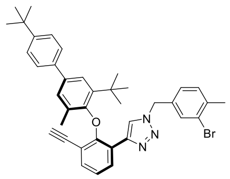
O
N
N
N
Br

3as

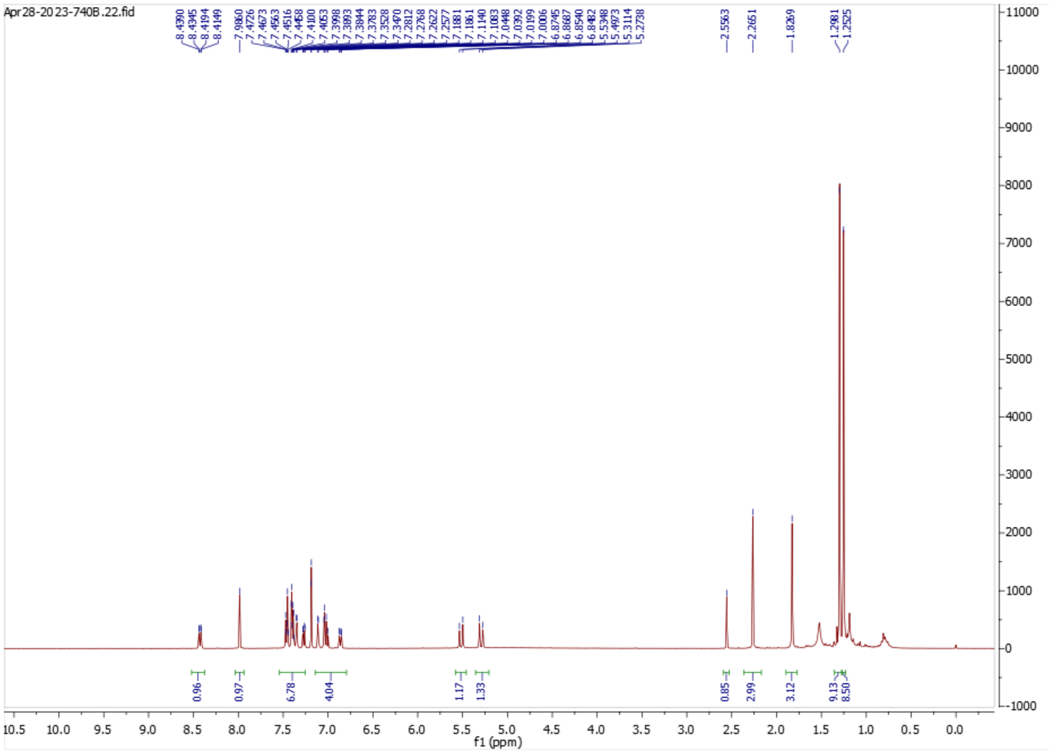
Apr28-2023-740B.22.fid
8.4390
8.4345
8.4194
8.4149
7.9860
7.4726
7.4673
7.4563
7.4516
7.4458
7.4100
7.4053
7.3998
7.3893
7.3844
7.3783
7.3528
7.3470
7.2812
7.2768
7.2622
7.2577
7.1881
7.1861
7.1140
7.1083
7.0448
7.0392
7.0199
7.0006
6.8745
6.8687
6.8540
6.8482
5.5348
5.4973
5.3114
5.2738
2.5563
2.2651
1.8269
1.2981
1.2525
0.96
0.97
6.78
4.04
1.17
1.33
0.85
2.99
3.12
9.13
8.50
f1 (ppm)

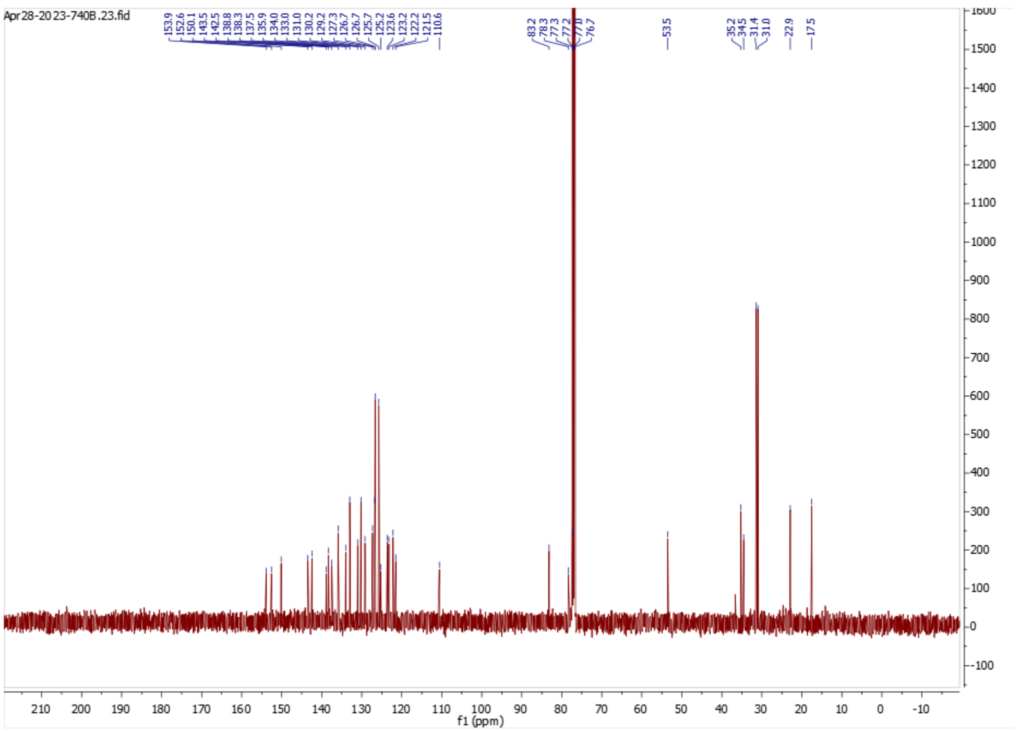
Apr28-2023-740B.23.fid
153.9
152.6
150.1
143.5
142.5
138.8
138.3
137.5
135.9
134.0
133.0
131.0
130.2
129.2
127.3
126.7
126.7
125.7
125.2
123.6
123.2
122.2
121.5
110.6
83.2
78.3
77.3
77.2
77.0
76.7
53.5
35.2
34.5
31.4
31.0
22.9
17.5
f1 (ppm)

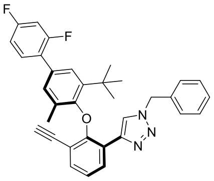
F
F
O
N
N
N

3at

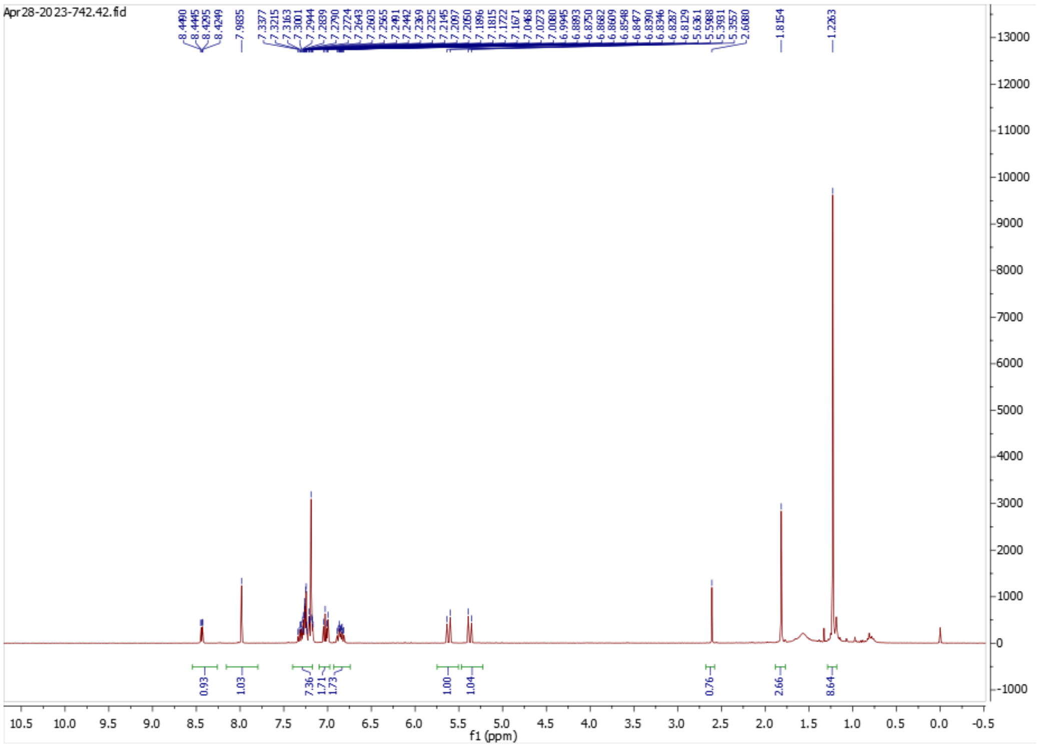
Apr28-2023-742.42.fid
f1 (ppm)

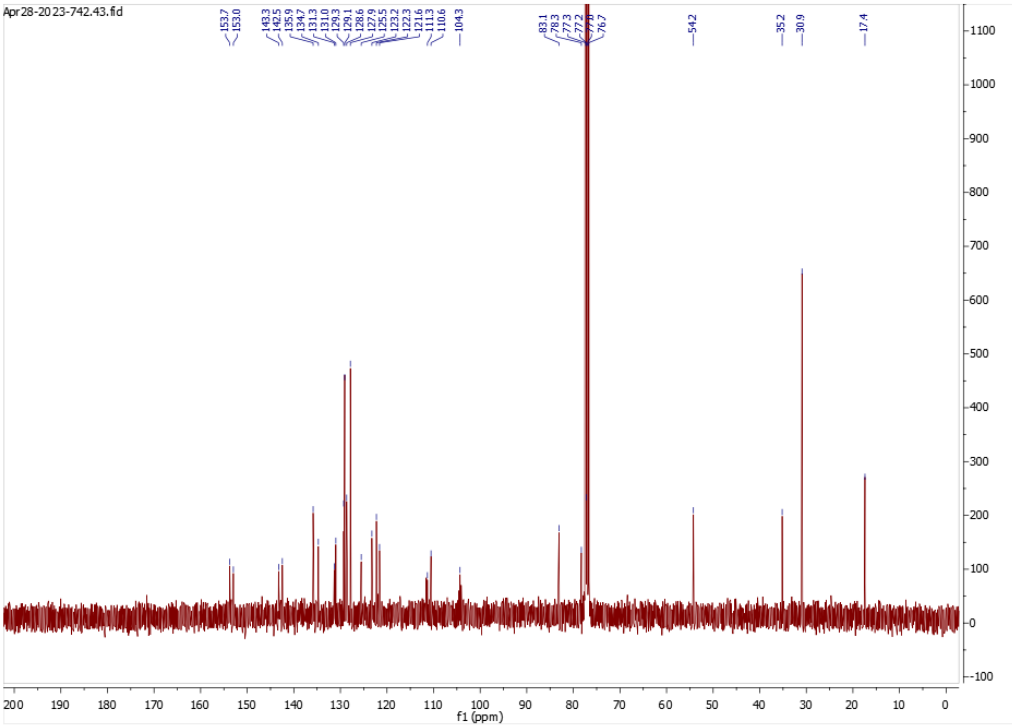
Apr28-2023-742.43.fid
f1 (ppm)

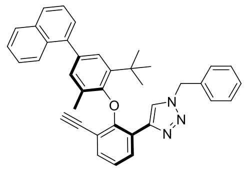

3at

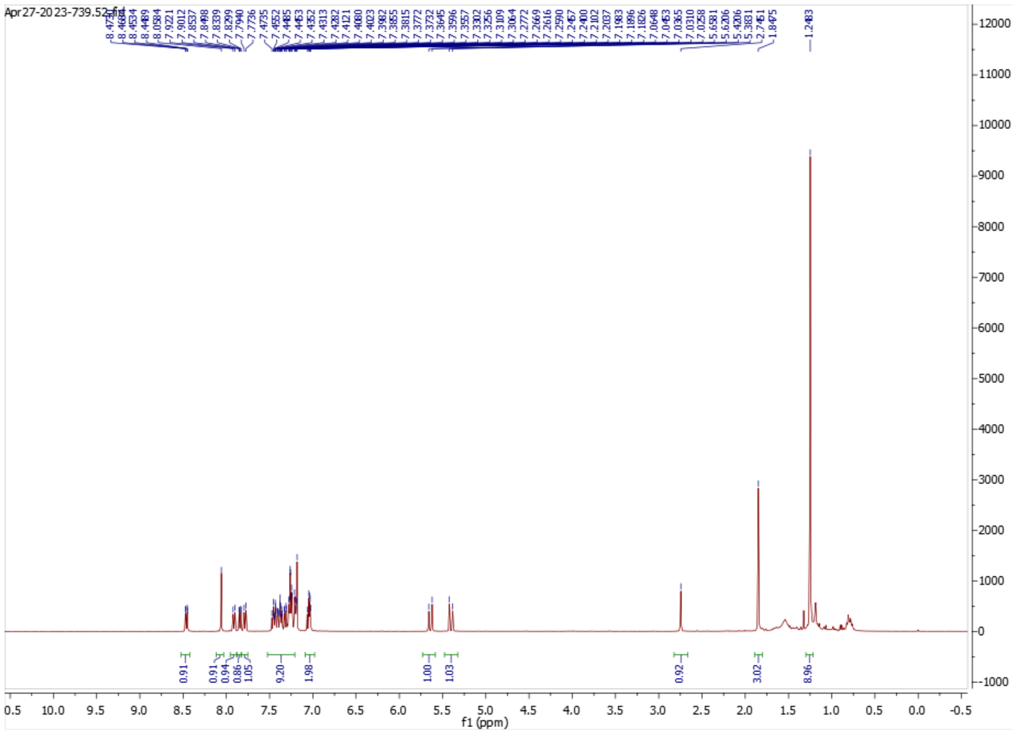

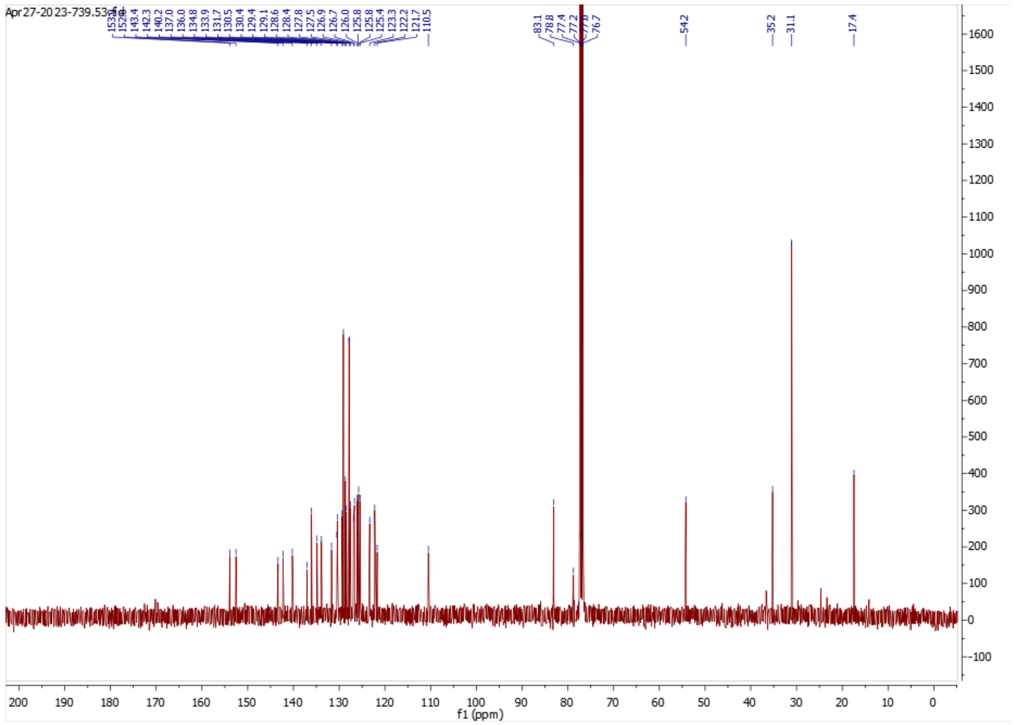

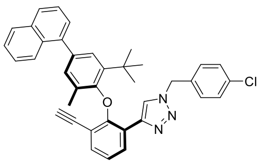

3at

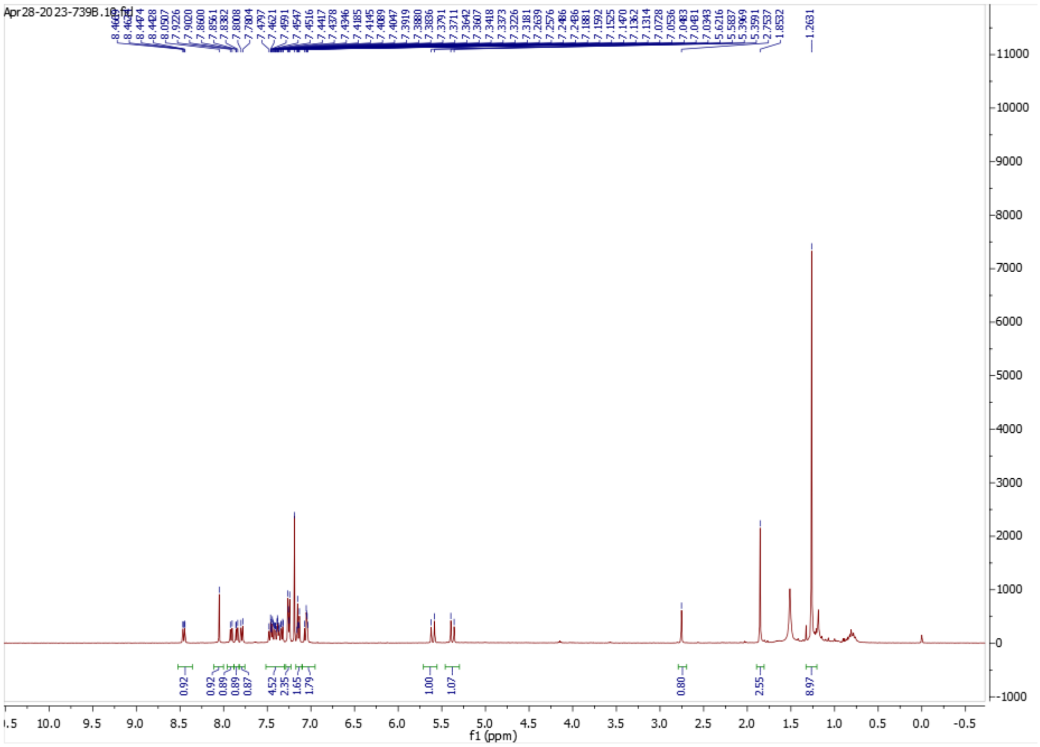

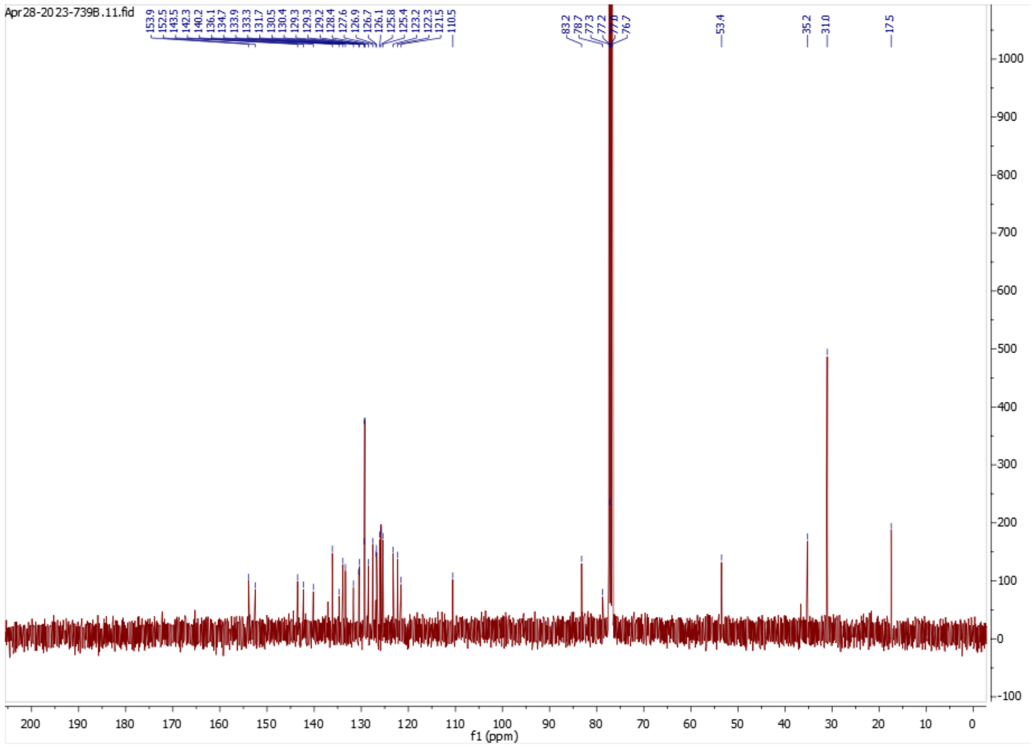

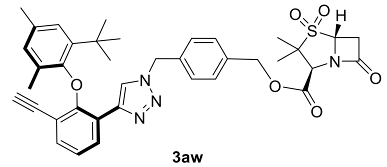
O
O
H
S
N
O
O
O
N
N
N
O

3aw

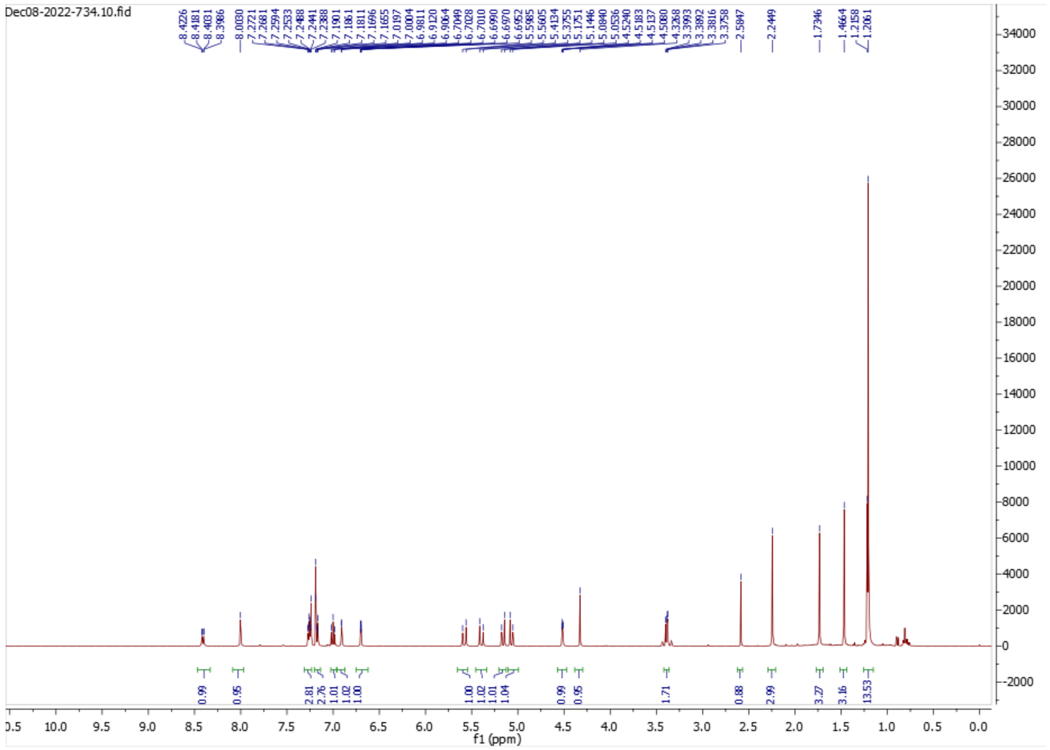
Dec08-2022-734.10.fid
f1 (ppm)

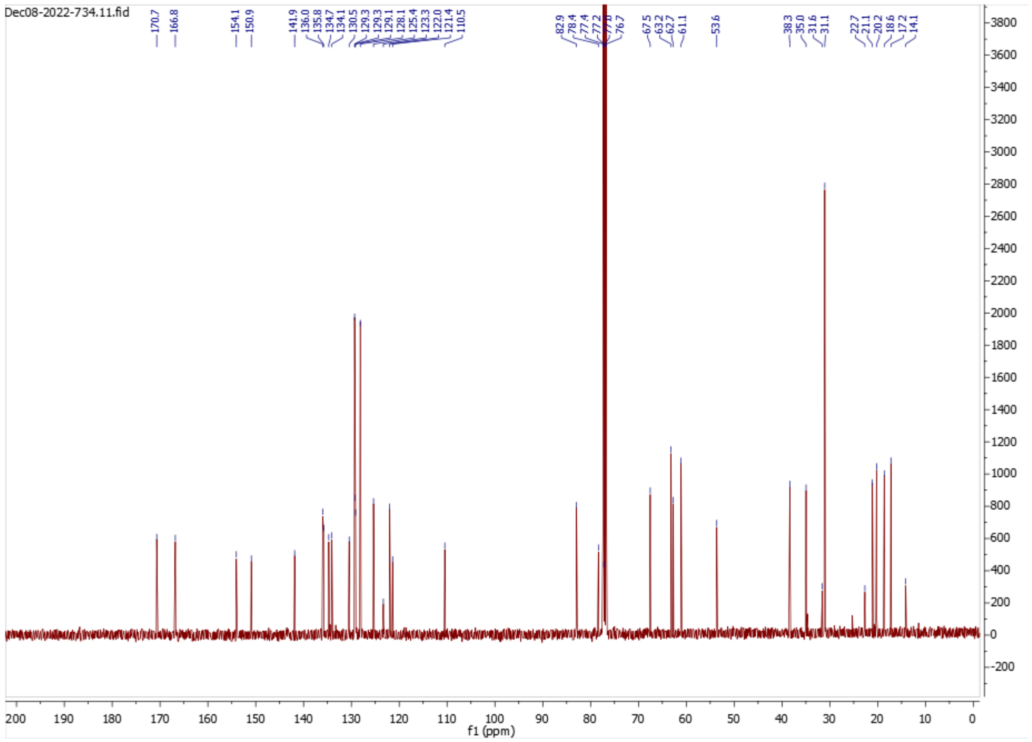
Dec08-2022-734.11.fid
170.7
166.8
154.1
150.9
141.9
136.0
135.8
134.7
134.1
130.5
129.3
129.3
128.1
125.4
123.3
122.0
121.4
110.5
82.9
78.4
77.4
77.2
77.0
76.7
67.5
63.2
62.7
61.1
53.6
38.3
35.0
31.6
31.1
22.7
21.1
20.2
18.6
17.2
14.1
f1 (ppm)

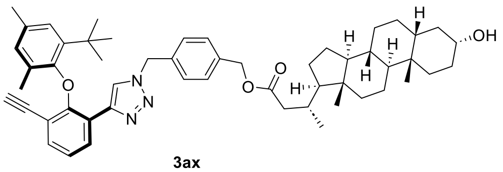

3ax

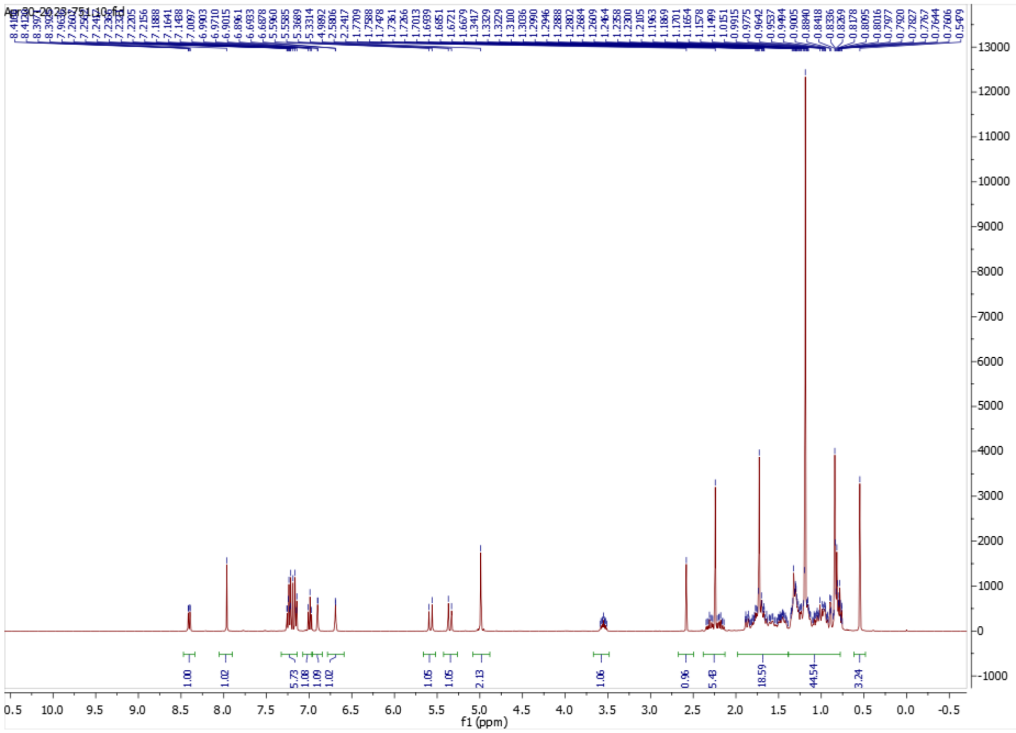

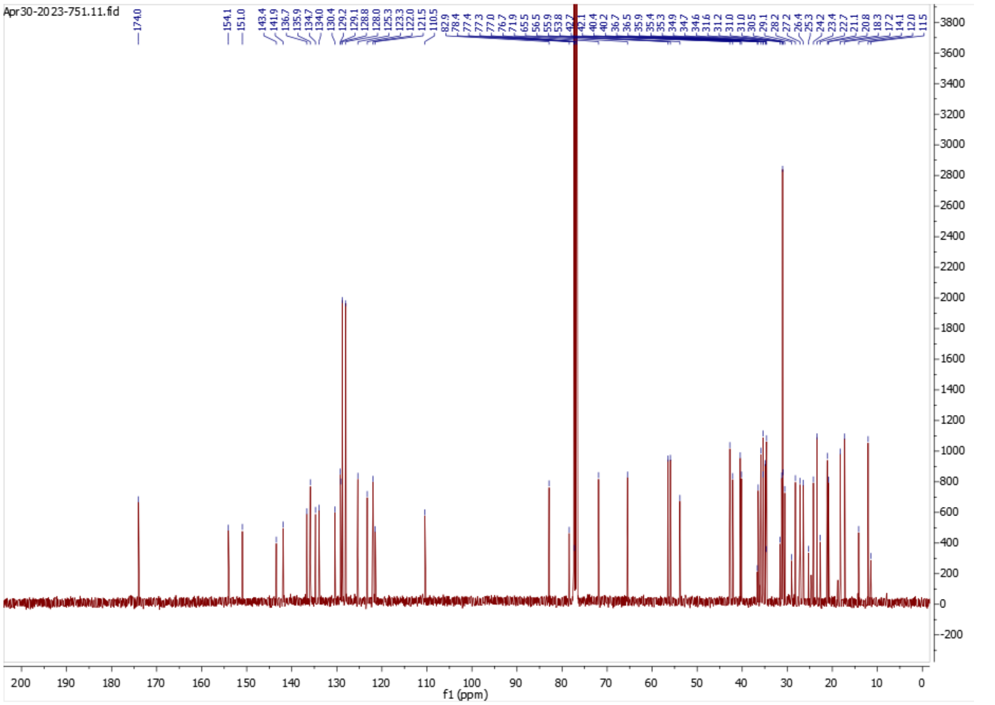

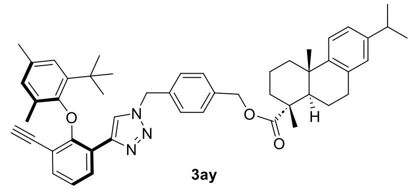

3ay

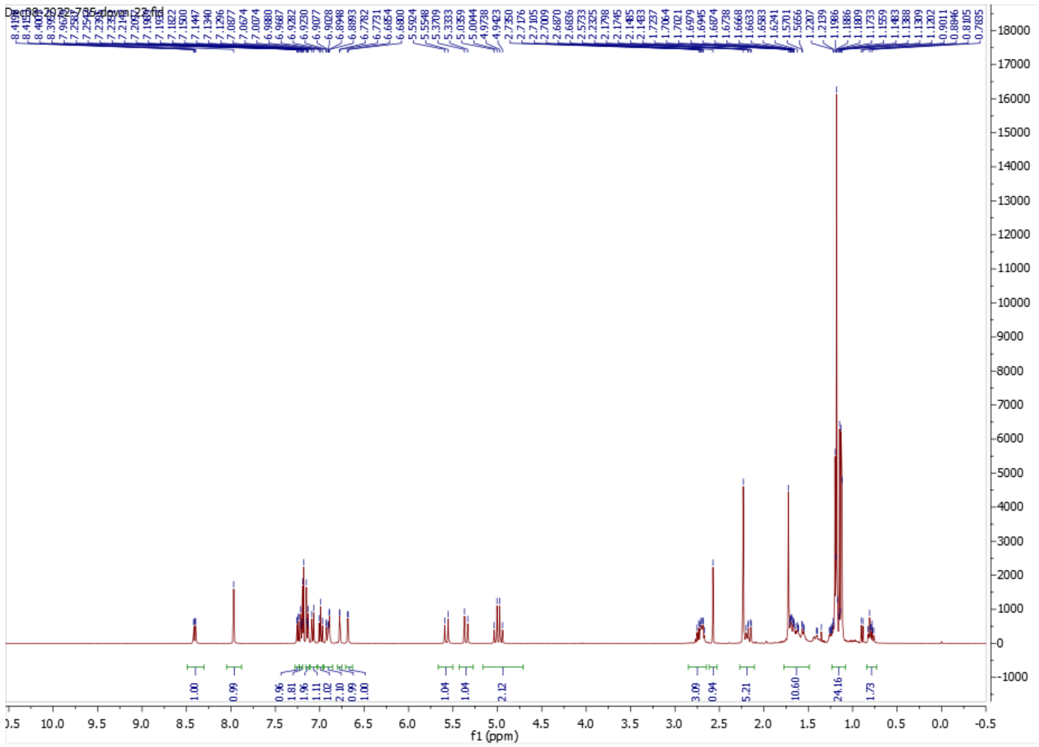

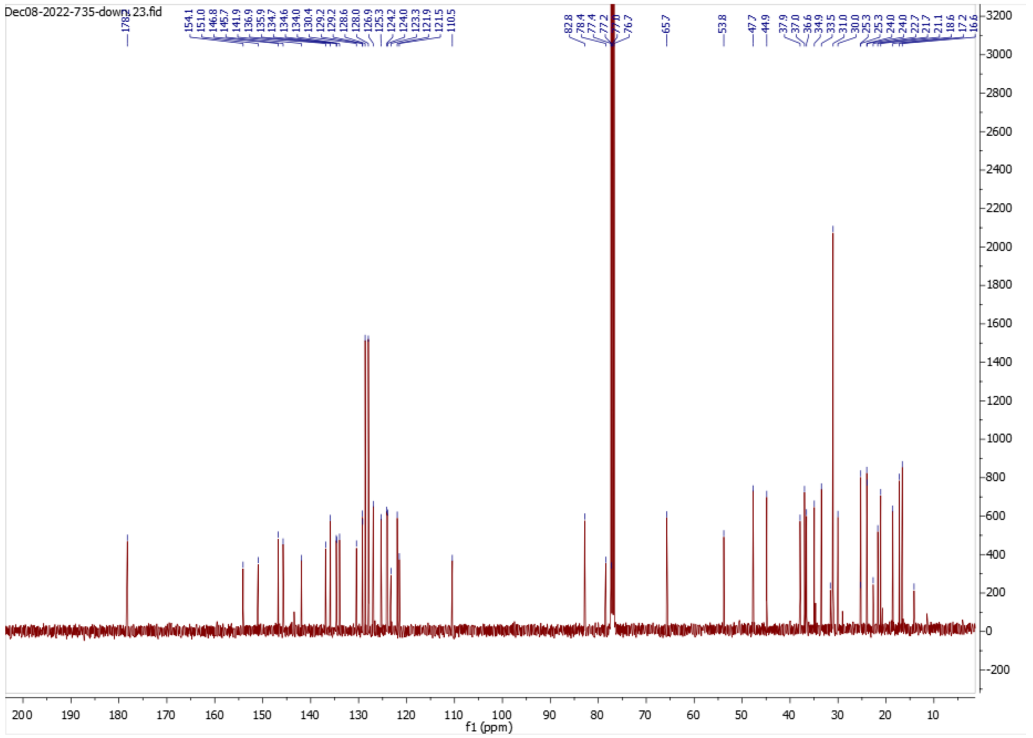

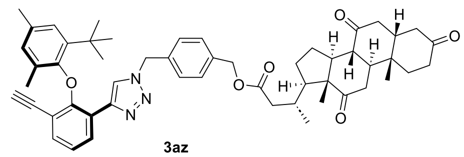

3az

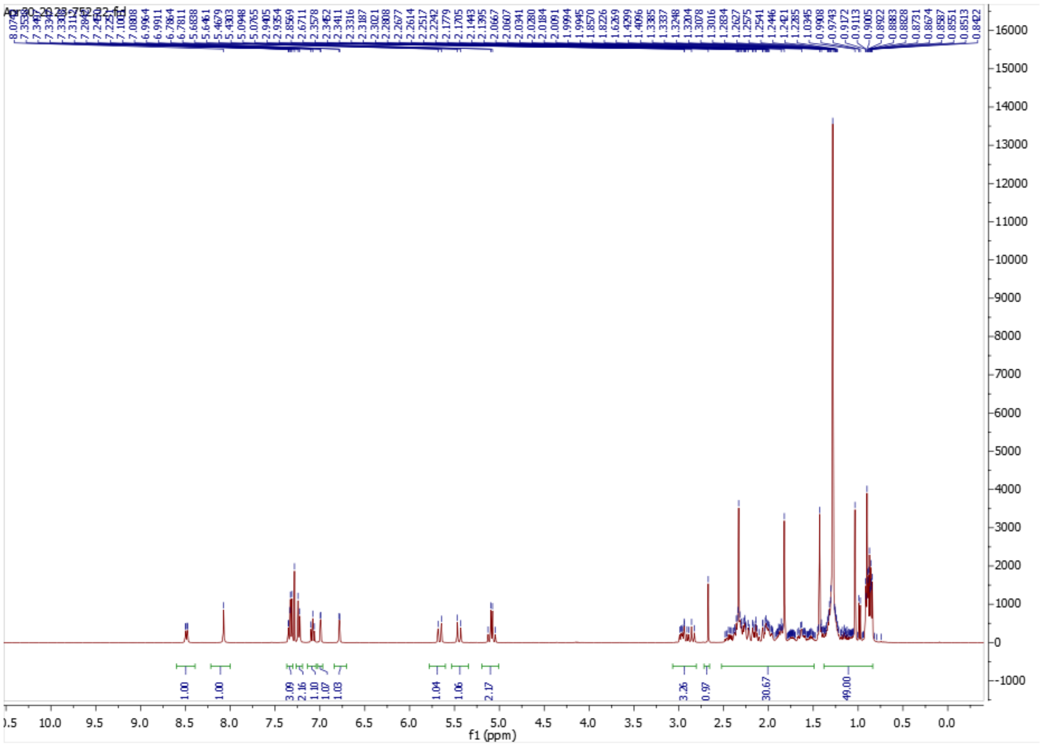

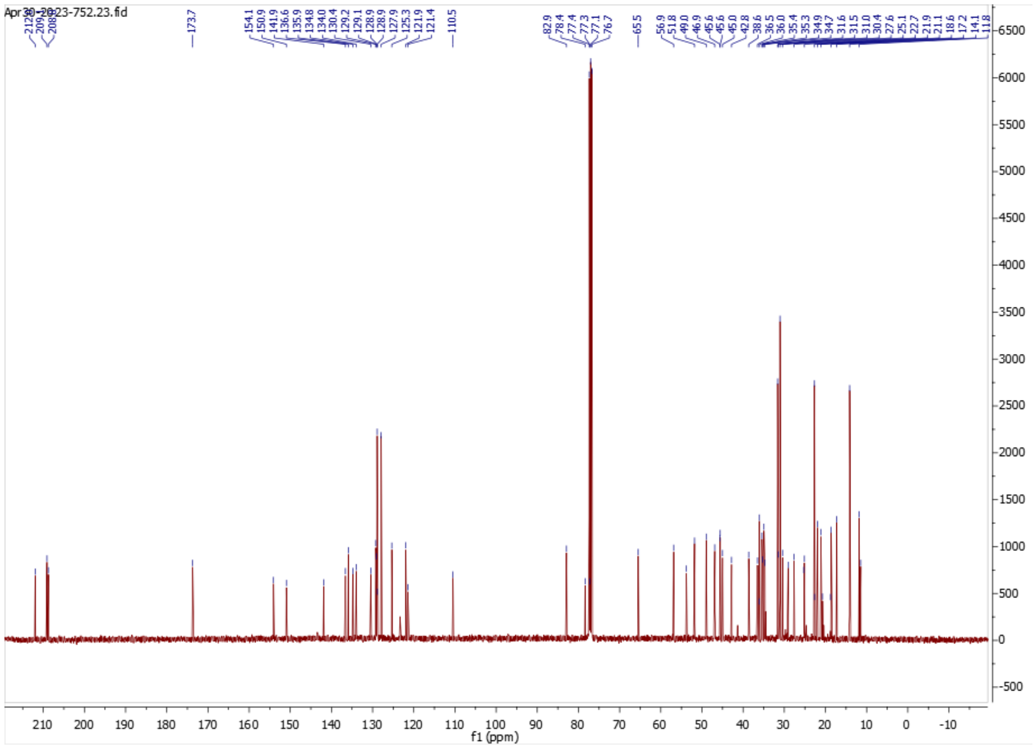

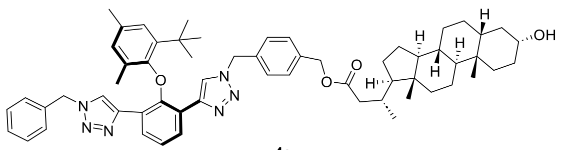

4a

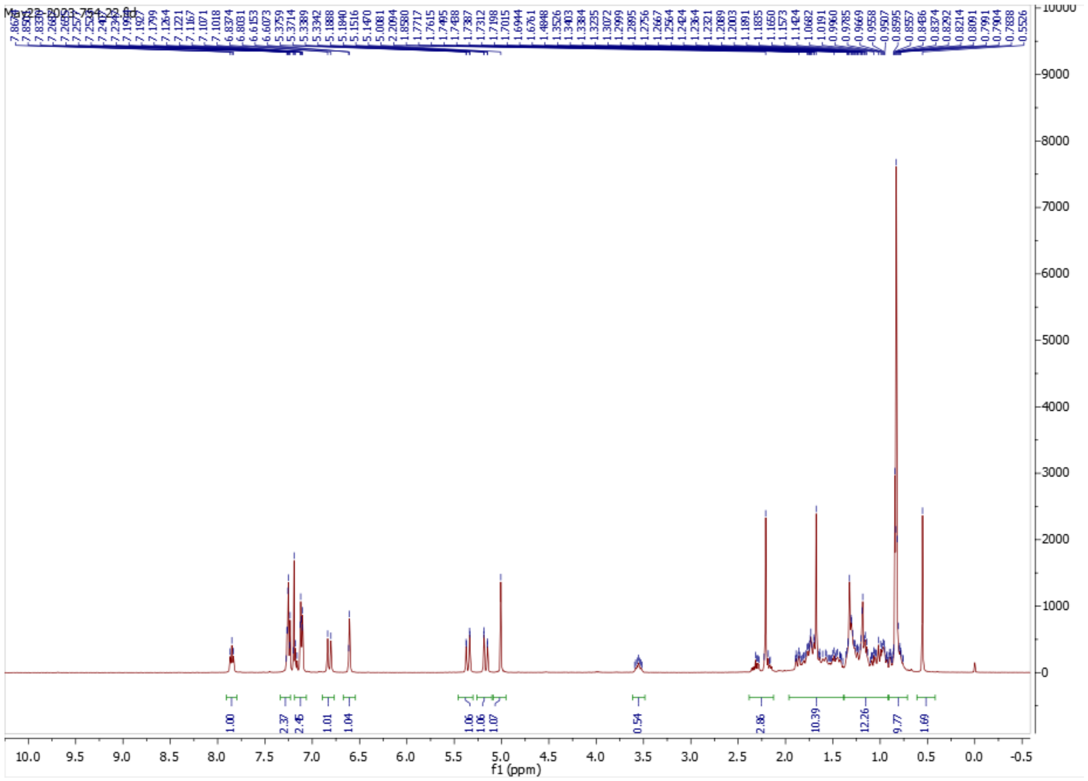

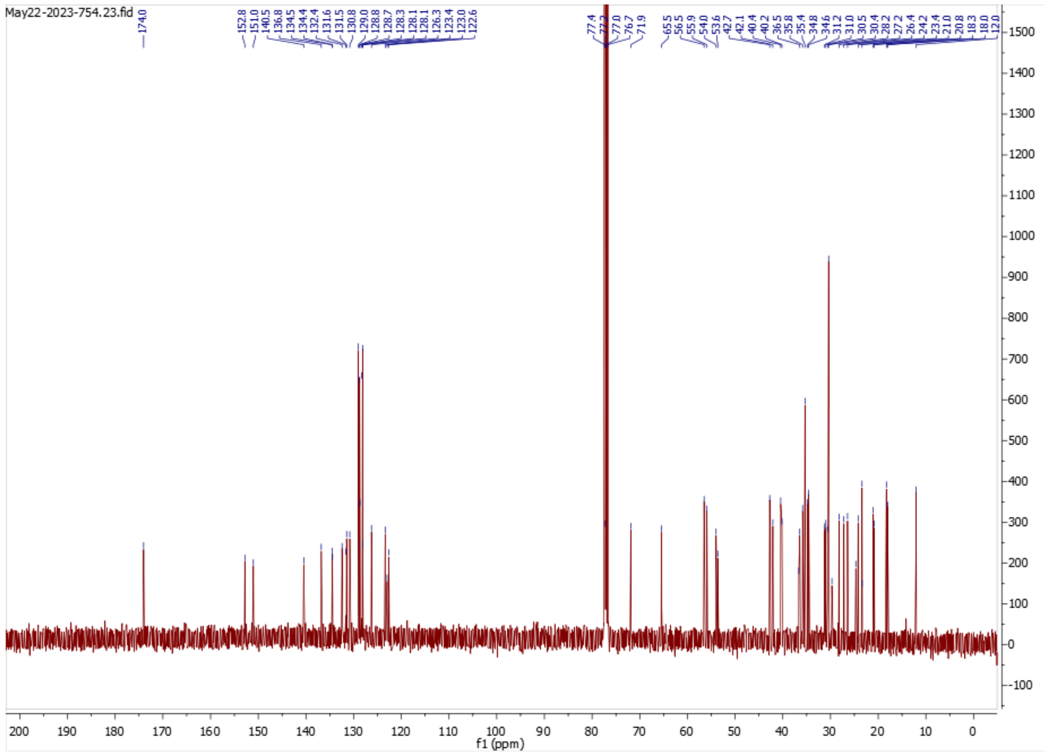

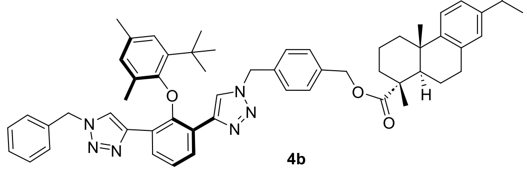

4b

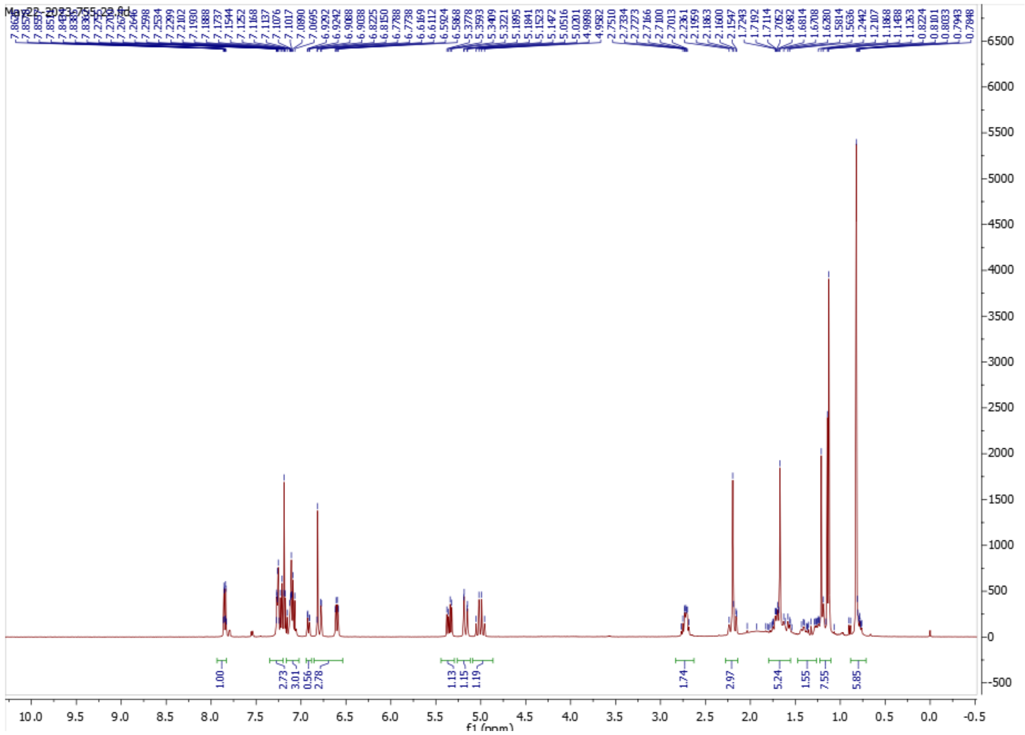

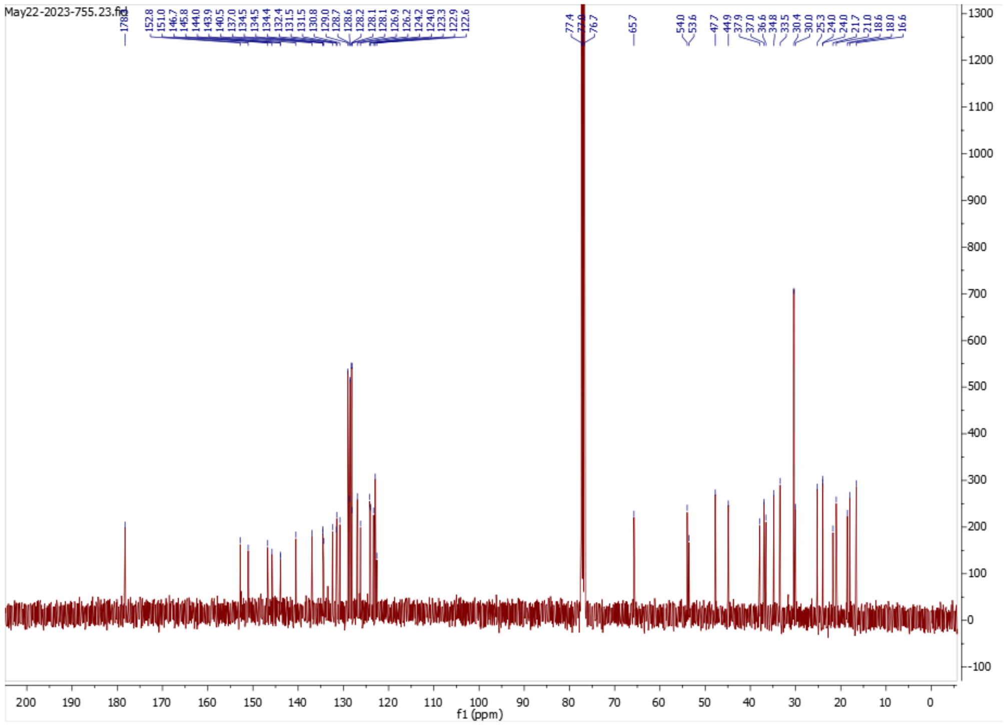

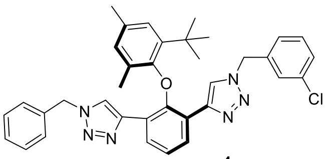

4c

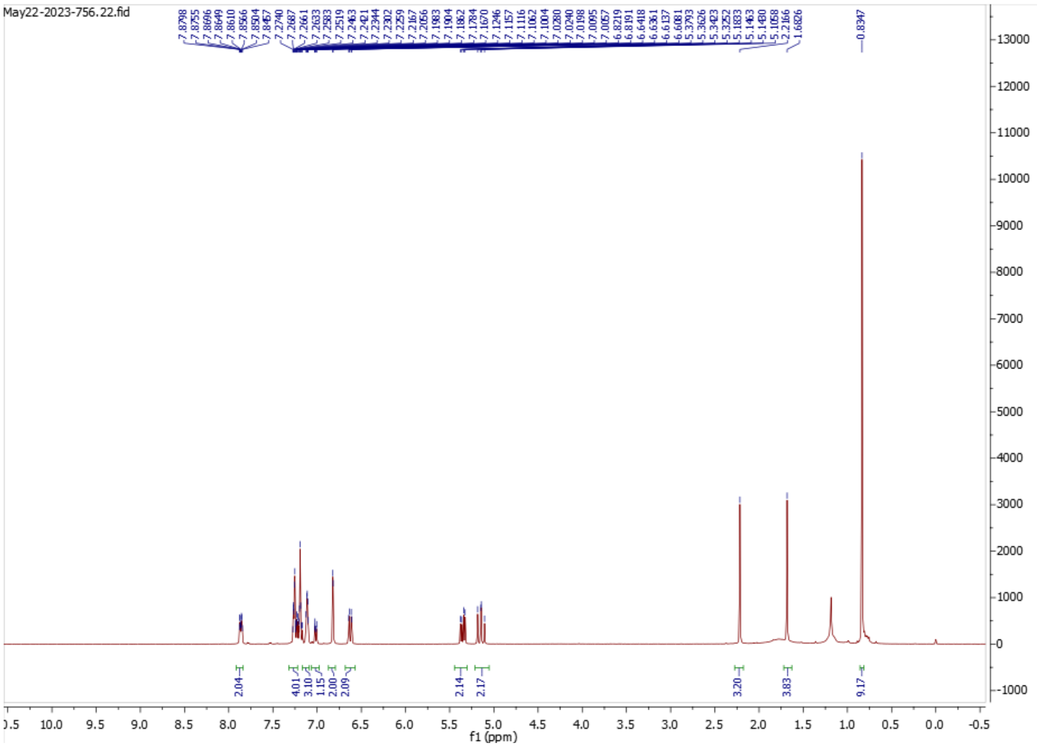

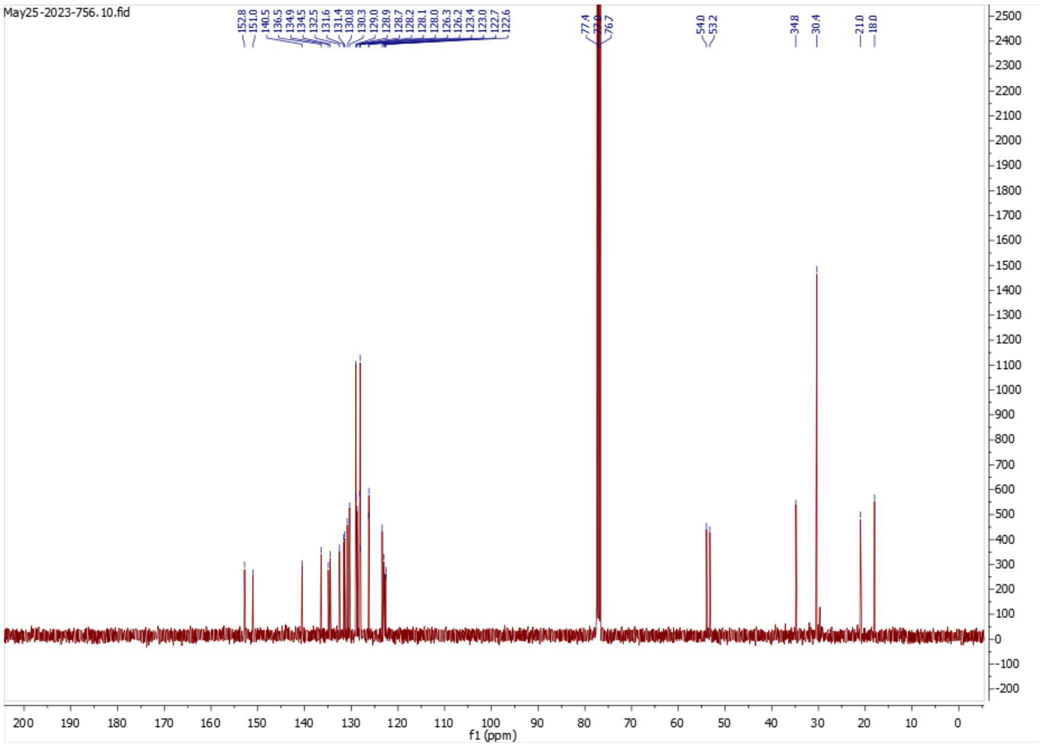

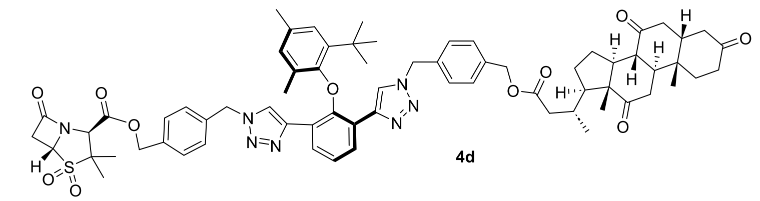

4d

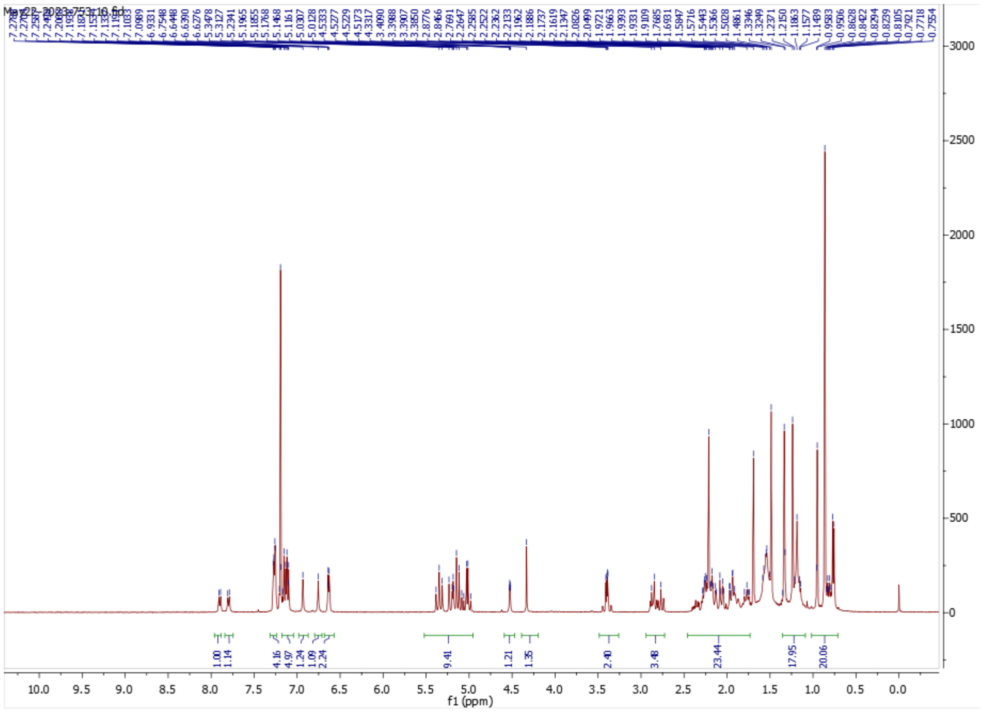

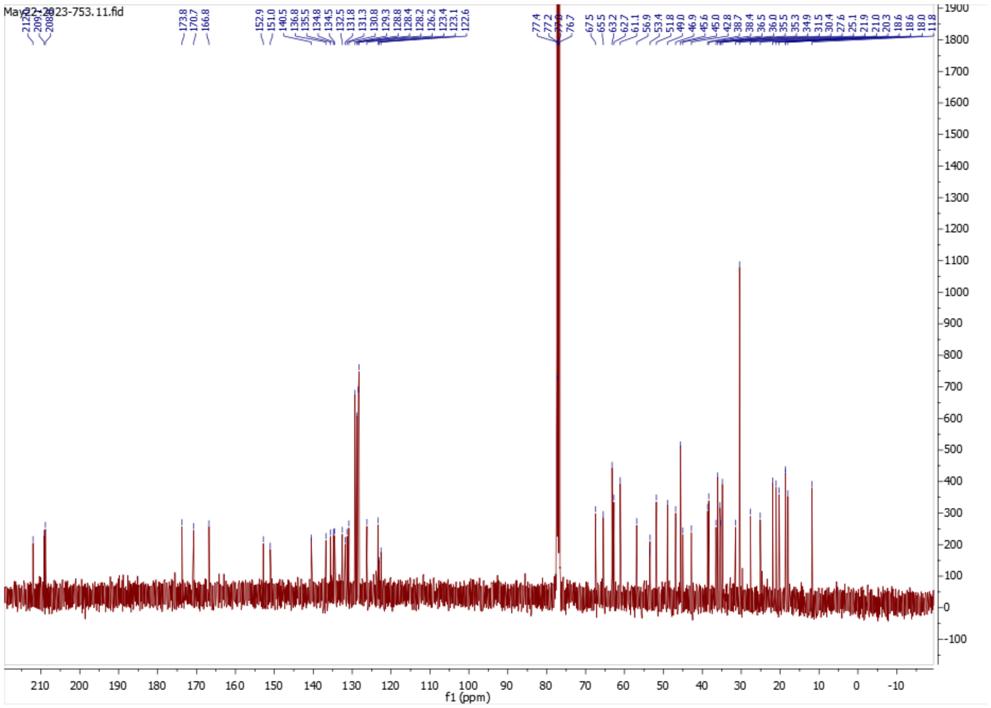

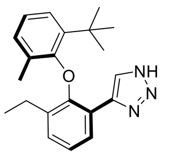
O
NH
N
N

5a

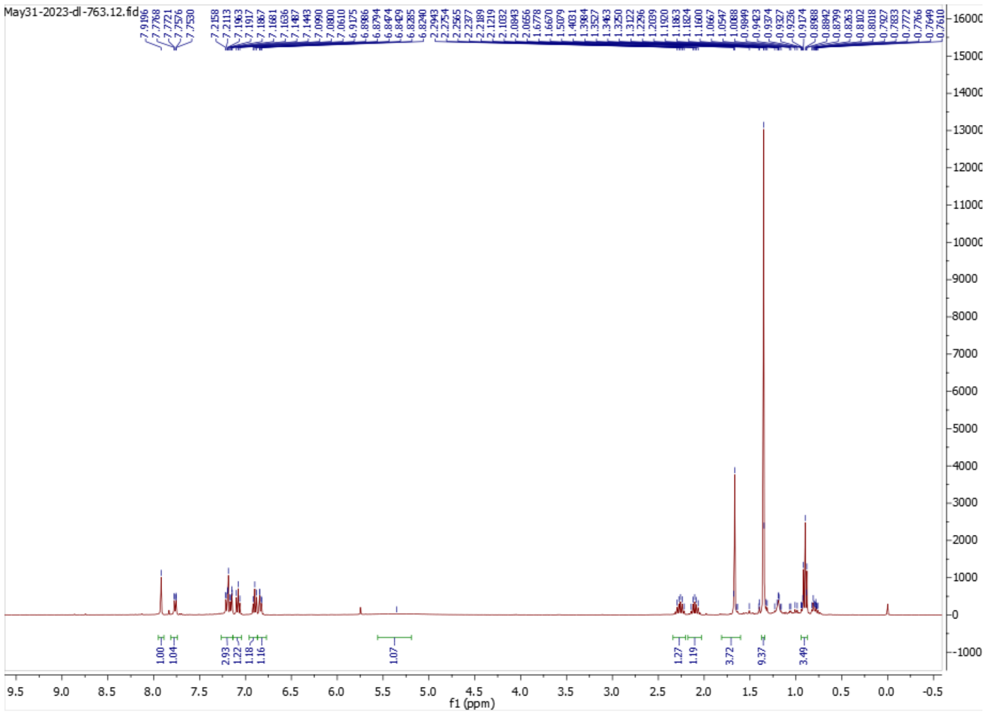
May31-2023-dl-763.12.fid
f1 (ppm)

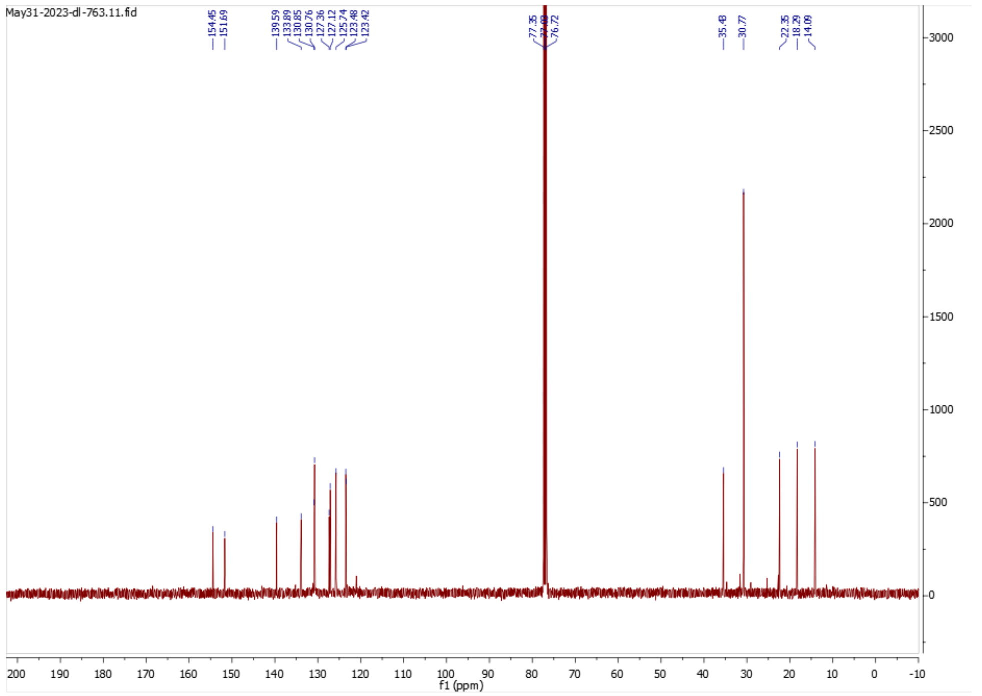
May31-2023-dl-763.11.fid
f1 (ppm)

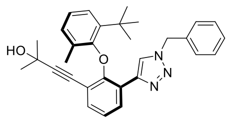

5b

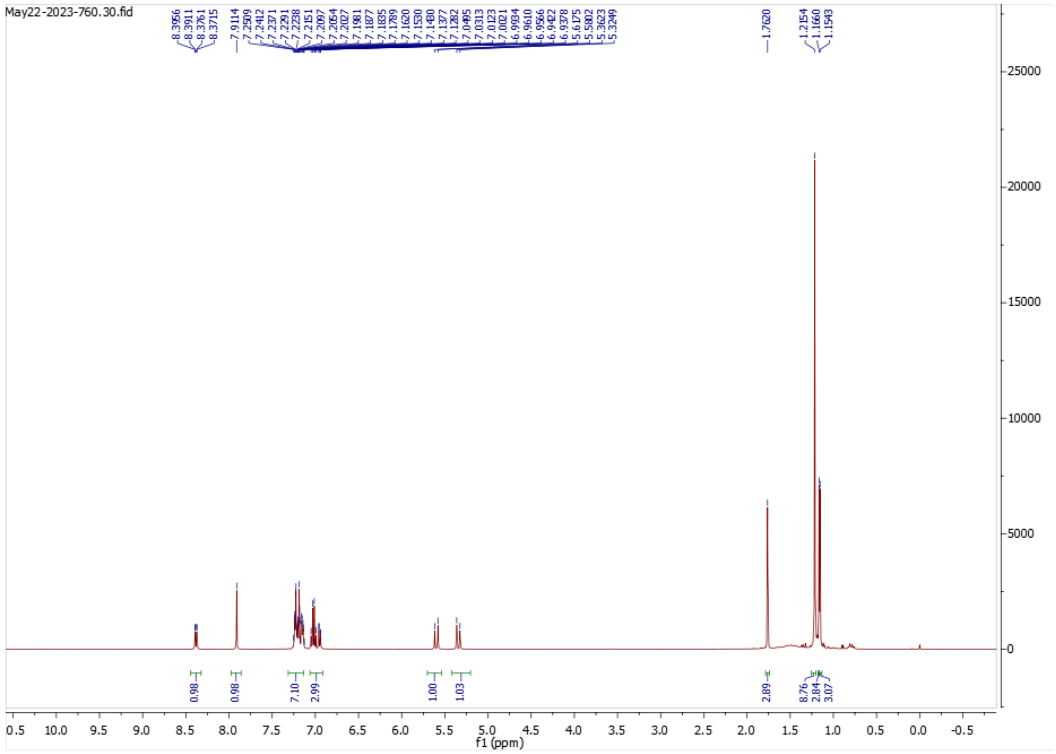

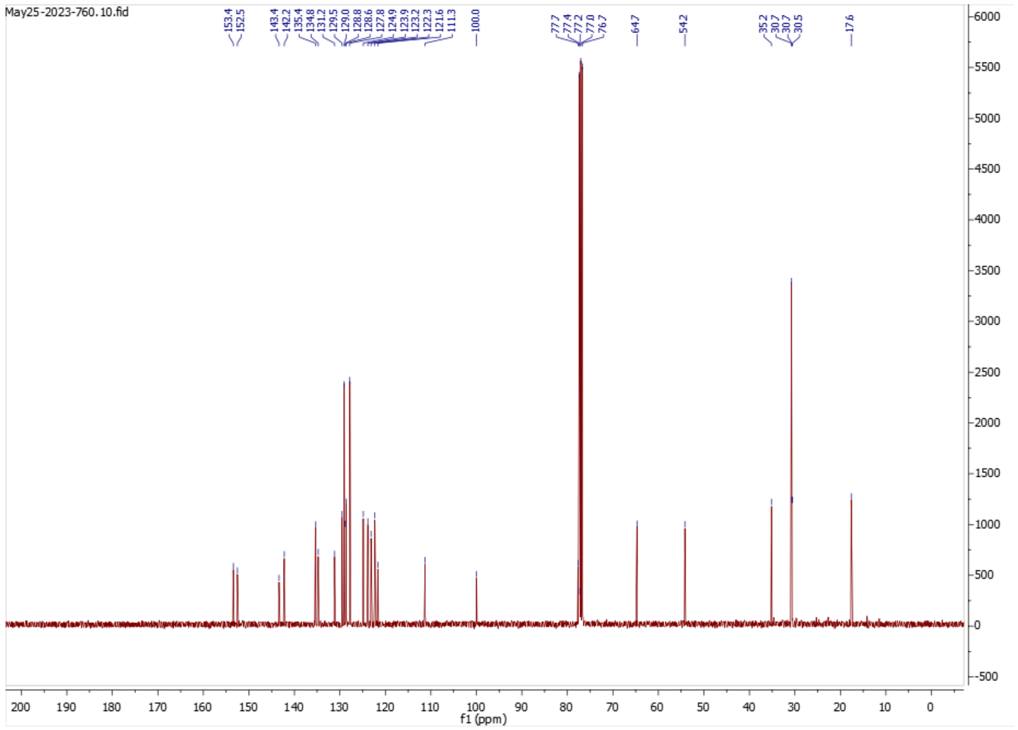

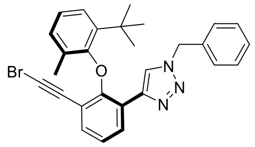
Br
O
N
N
N

5c

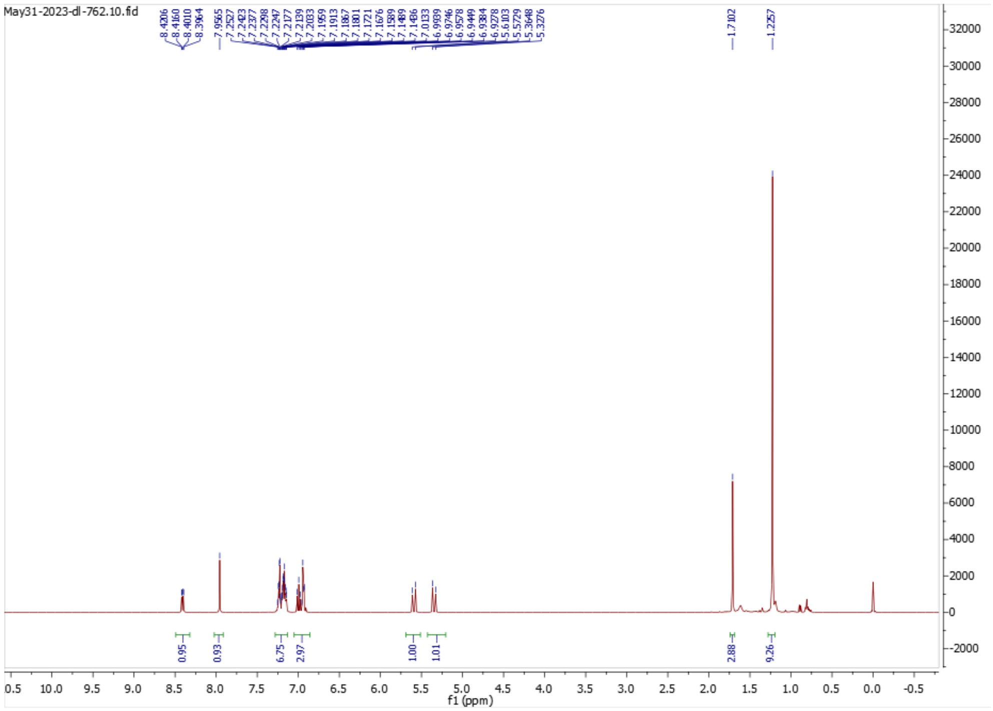
May31-2023-dl-762.10.fid
f1 (ppm)

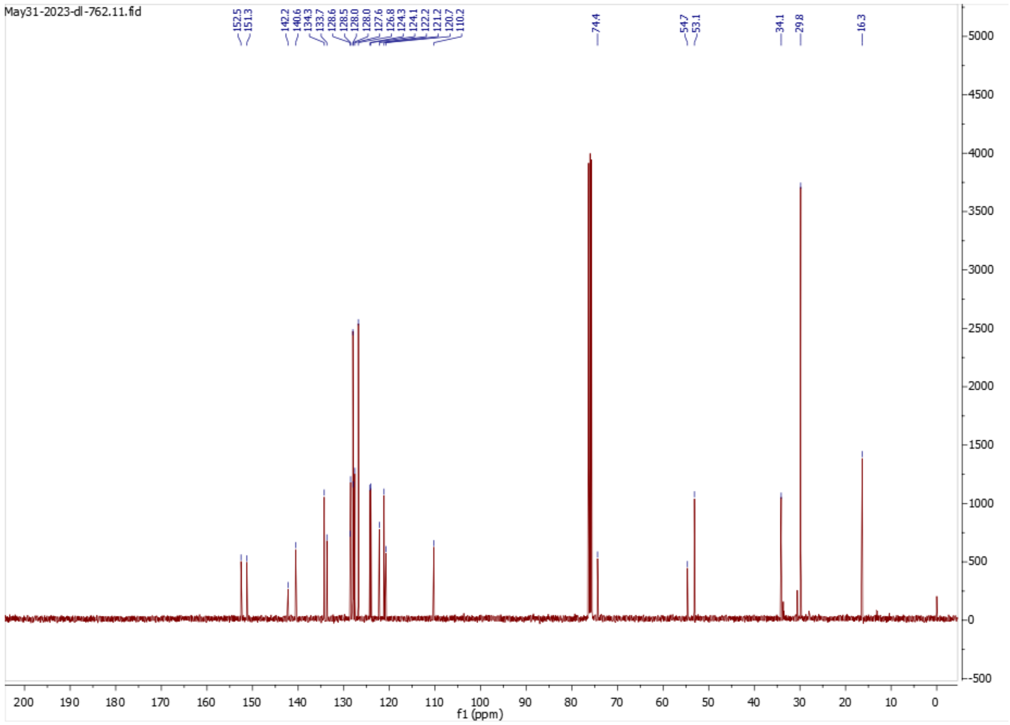
May31-2023-dl-762.11.fid
f1 (ppm)

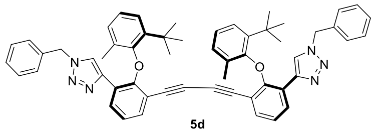

5d

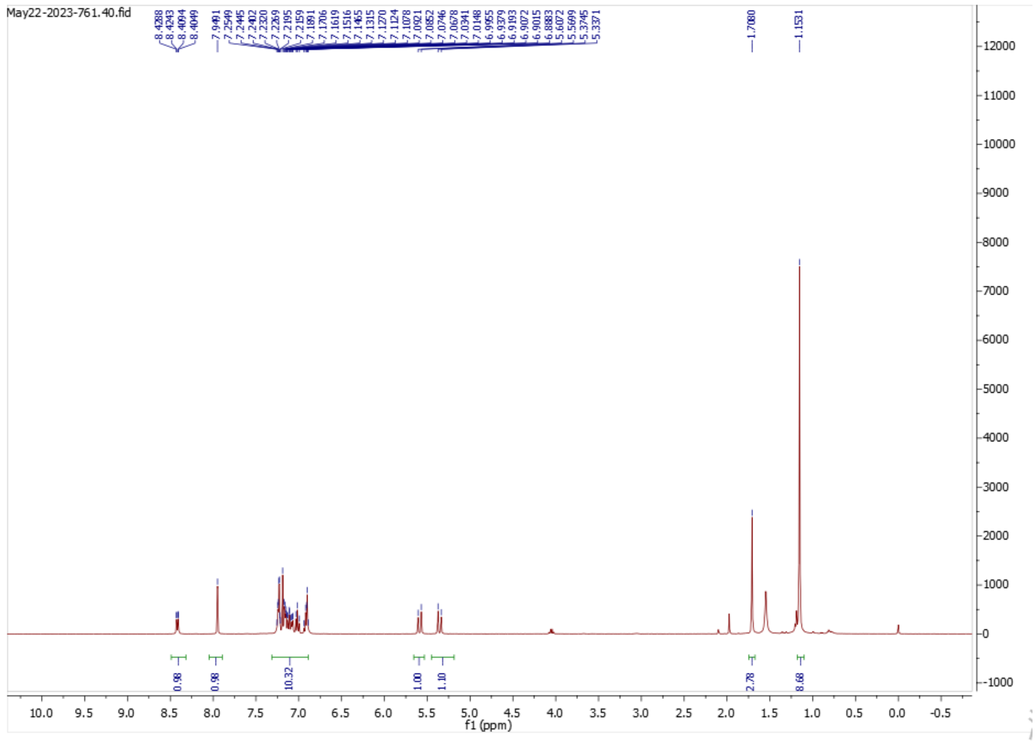

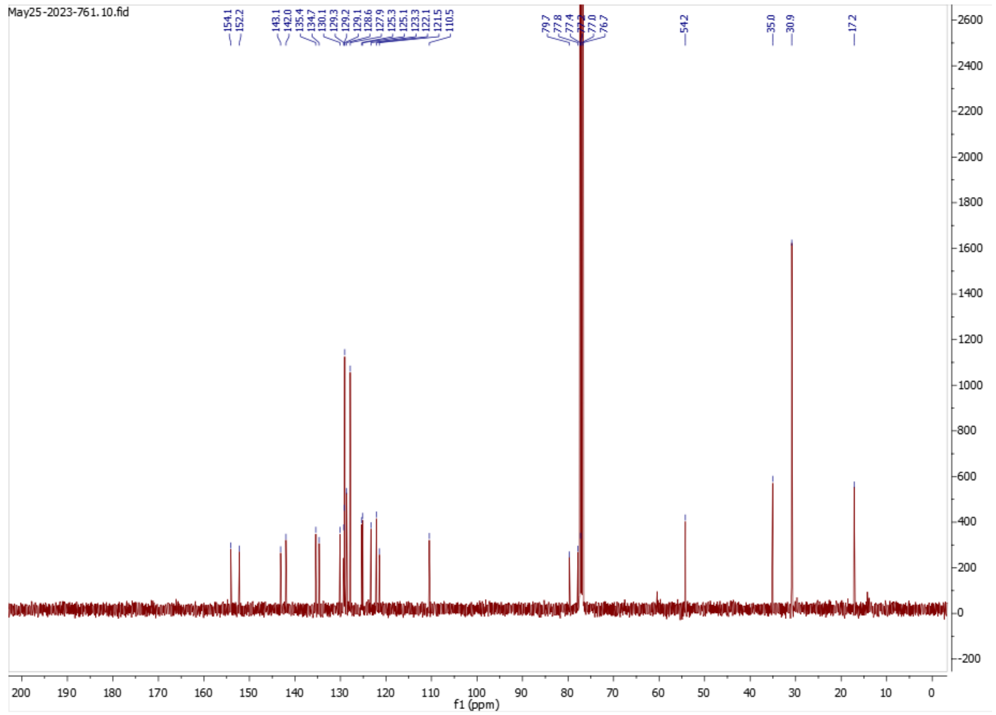

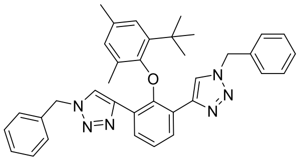

O
N
N
N
N
N
N

3a'

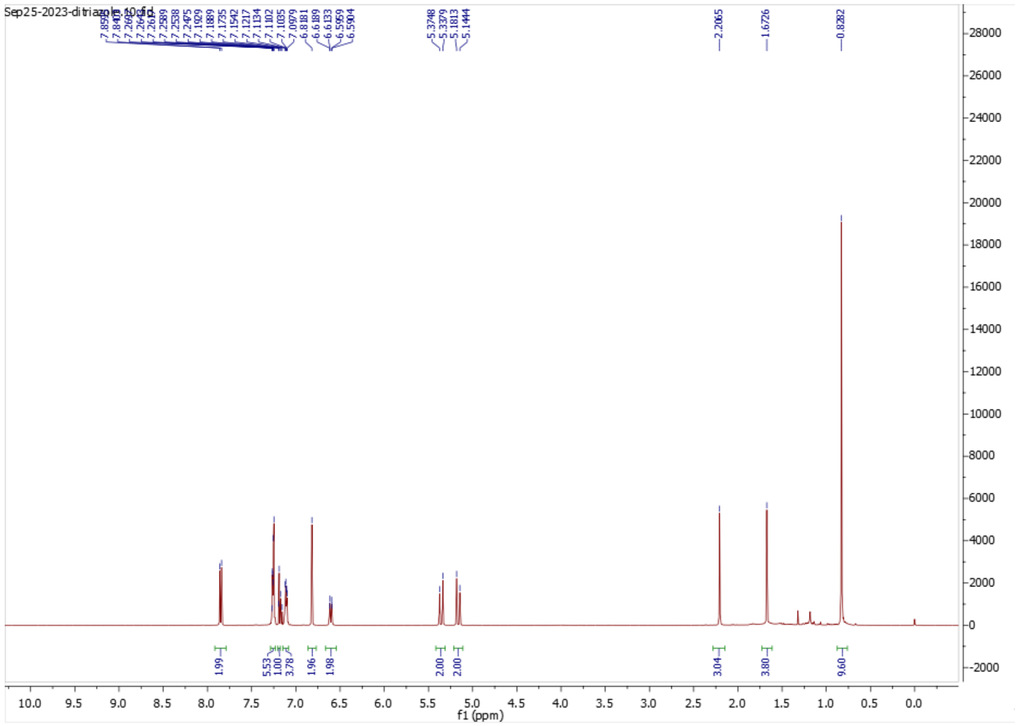

Sep25-2023-ditriazole.10.fid
6.8181
6.6189
6.6133
6.5959
6.5904
5.3748
5.3379
5.1813
5.1444
2.2065
1.6726
0.8282
1.99
5.53
1.00
3.78
1.96
1.98
2.00
2.00
3.04
3.80
9.60
f1 (ppm)

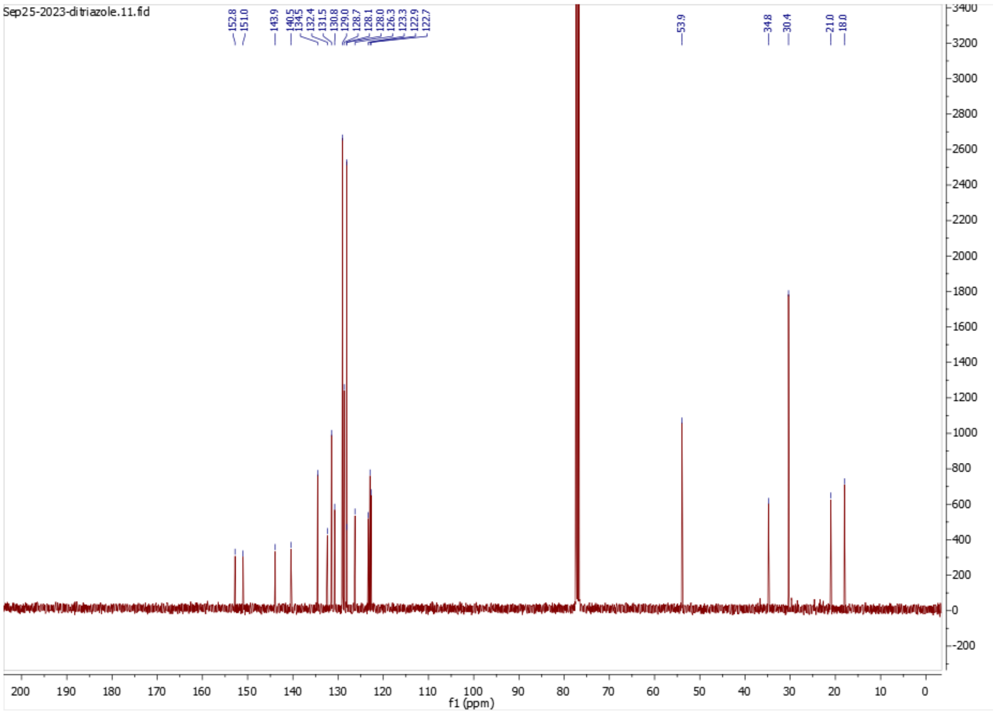

Sep25-2023-ditriazole.11.fid
152.8
151.0
143.9
140.5
134.5
132.4
131.5
130.8
129.0
128.7
128.1
128.0
126.3
123.3
122.9
122.7
53.9
34.8
30.4
21.0
18.0
f1 (ppm)

10. References

(1). Yuan, B.; Page, A.; Worrall, C. P.; Escalettes, F.; Willies, S. C.; McDouall, J. J. W.; Turner, N. J.; Clayden, J. Biocatalytic Desymmetrization of an Atropisomer with both an Enantioselective Oxidase and Ketoreductases. *Angew. Chem., Int. Ed.* **2010,** *49*, 7010-7013.
(2). Dai, L.; Liu, Y.; Xu, Q.; Wang, M.; Zhu, Q.; Yu, P.; Zhong, G.; Zeng, X. A Dynamic Kinetic Resolution Approach to Axially Chiral Diaryl Ethers by Catalytic Atroposelective Transfer Hydrogenation. *Angew. Chem., Int. Ed.* **2023,** *62*, e202216534.
(3). Bao, H.; Chen, Y.; Yang, X. Catalytic Asymmetric Synthesis of Axially Chiral Diaryl Ethers through Enantioselective Desymmetrization. *Angew. Chem., Int. Ed.* **2023,** *62*, e202300481.
(4). Shee, S.; Shree Ranganathappa, S.; Gadhave, M. S.; Gogoi, R.; Biju, A. T. Enantioselective Synthesis of C−O Axially Chiral Diaryl Ethers by NHC-Catalyzed Atroposelective Desymmetrization. *Angew. Chem., Int. Ed.* **2023,** *62*, e202311709.
(5). Zhou, B.-A.; Li, X.-N.; Zhang, C.-L.; Wang, Z.-X.; Ye, S. Enantioselective Synthesis of Axially Chiral Diaryl Ethers via NHC Catalyzed Desymmetrization and Following Resolution. *Angew. Chem., Int. Ed.* **2024,** *63*, e202314228.
(6). Zeng, L.; Li, J.; Cui, S. Rhodium-Catalyzed Atroposelective Click Cycloaddition of Azides and Alkynes. *Angew. Chem., Int. Ed.* **2022,** *61*, e202205037.
